# Flexibility of the TRMT10C N‑Terminal Domain Helix Explored by Molecular Dynamics Simulations

**DOI:** 10.1021/acs.jpcb.6c03249

**Published:** 2026-10-07

**Authors:** Gia Linh Hoang, Giulia Rossetti, Carine Tisné, Paolo Carloni

**Affiliations:** † Institute for Neuroscience and Medicine (INM-9), 196554 Forschungszentrum Jülich, 52425 Jülich, Germany; ‡ Department of Neurology, Medical Faculty, RWTH Aachen University, 52074 Aachen, Germany; § Jülich Supercomputing Centre (JSC), Forschungszentrum Jülich, 52425 Jülich, Germany; ∥ Université Paris Cité, CNRS, Institut de Biologie Physico-Chimique (IBPC), Microbial gene expression unit, 75005 Paris, France

## Abstract

The human mitochondrial tRNA methyltransferase TRMT10C contains a 41-residue long positively charged α-helix in its N-terminal domain, interacting with tRNAs from the anticodon to the T-loops. Here, we investigated the flexibility of this N-terminal long α-helix using comparative molecular dynamics simulations in explicit solvent with three different force fields/water model combinations, alongside two helices of identical length but different sequences: a polyalanine helix and an AEEEKRK repeat helix. We also expanded our simulations to three additional helices belonging to the Trm10 family. Five independent 1-μs simulations were performed for each system. The human TRMT10C helix turned out to display heterogeneous dynamics, with a stable α-helical segment (residues 132–139) embedded within more flexible regions and this trend was also observed in the human Trm10 family members. This relatively rigid segment is more hydrophobic, whereas the remaining portion is enriched in charged residues and shows higher conformational variability. The rigid region corresponds to the portion interacting with the cavity formed by tRNA and TRMT10C N-terminal domain. Additional simulations using alternative force-field and water-model combinations supported the robustness of the overall flexibility ranking for the AEEEKRK repeat helix and the TRMT10C helix, but it gave force-field-dependent results for the structural dynamics of the polyalanine helix. Simulations of the N-terminal helices of other TRMT10C homologues revealed similar flexibility profiles, suggesting that the observed trends are generalizable across the family. Based on our results, we suggest that the human TRMT10C helix is tuned to combine localized rigidity to provide localized structural support that helps present the fragile mitochondrial tRNA to the maturation enzymes with overall flexibility for conformational adaptation, facilitating tRNA positioning and efficient catalysis.

## Graphical abstract

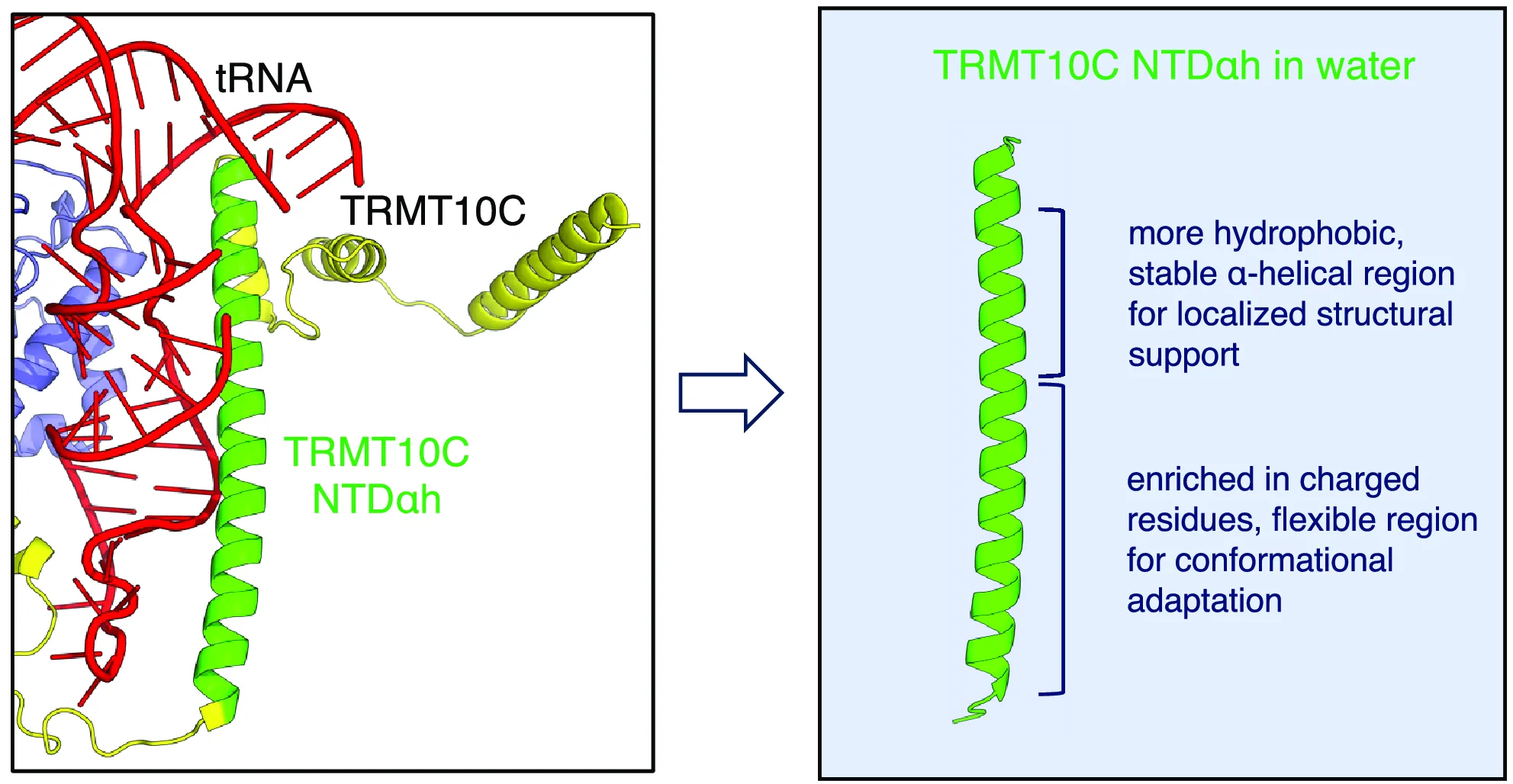

## Introduction

The human mitochondrial tRNA methyltransferase 10C (TRMT10C) (Figure 1a), which belongs to the Trm10 family of tRNA methyltransferases (MTases) found in archaea and eukaryotes, catalyzes the m^1^A9 and m^1^G9 modifications in human mitochondrial tRNAs, using S-adenosyl-l-methionine (SAM) as the methyl donor cofactor.^1–3^ Several TRMT10C mutations, including R181L, R182L, and T272A, are associated with a variety of diseases including lactic acidosis, hypotonia, feeding difficulties, deafness, and liver dysfunction.^4,5^

**Figure 1:**
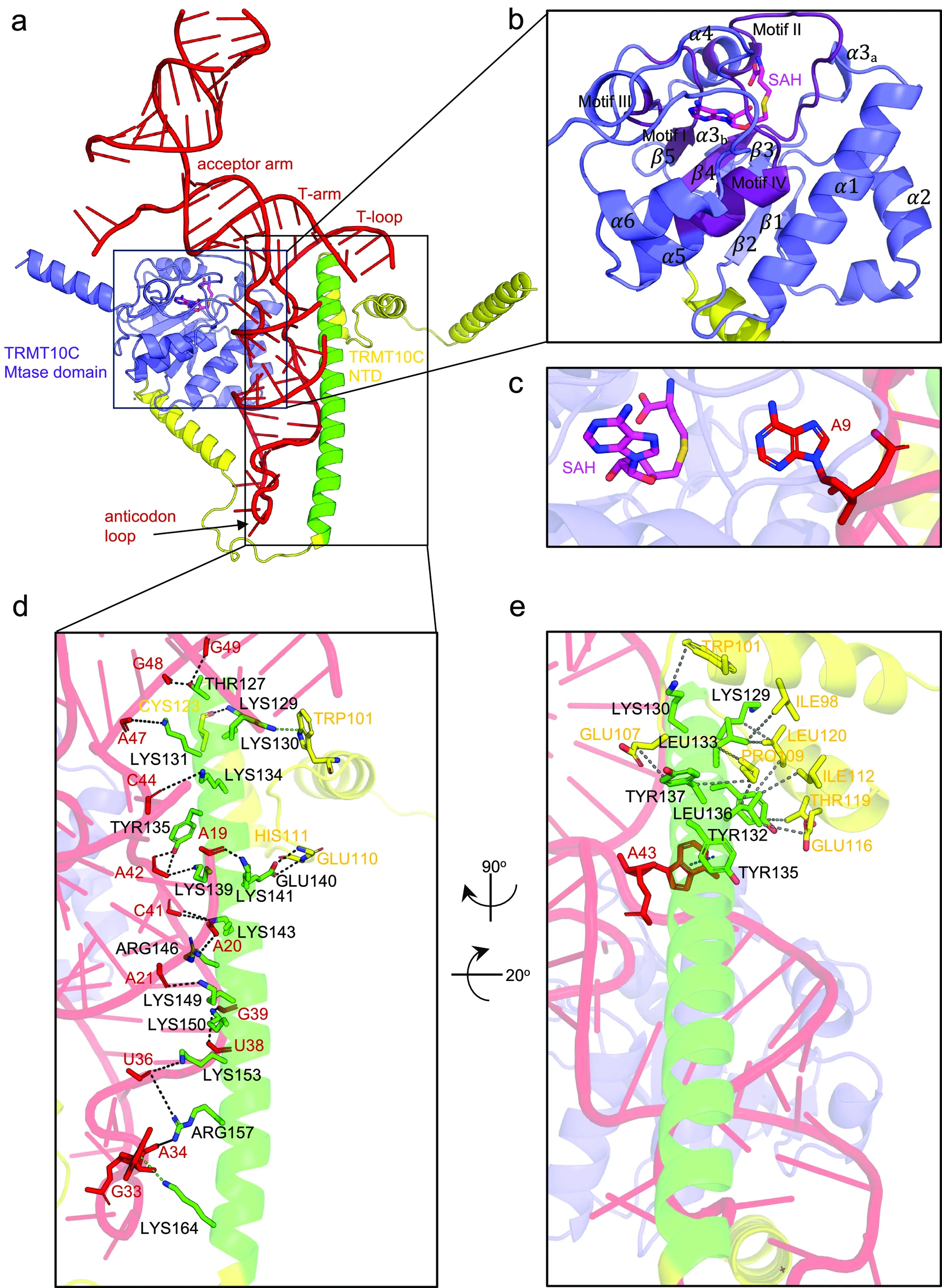
(a) Human tRNA/TRMT10C complex (PDBid 8CBK^4^) in cartoon representation. tRNA is colored in red; TRMT10C MTase domain is colored in slate blue; TRMT10C N-terminal domain (NTD) is colored in yellow, except for **NTDαh** (residues 126–166), which is colored in green. (b) SPOUT fold of the TRMT10C MTase domain consists of a central β-sheet flanked on both sides by α-helices. Four conserved motifs I–IV around the binding pocket are highlighted in purple. Some parts of the complex are removed for clarity. (c) A9 (in red) “flipped out” from the tRNA and SAH (in magenta). (d) Hydrogen bonds/salt bridges and π-cation intermolecular interactions involving **NTDαh**, represented by black and green dashed lines, respectively. (e) π-stacking (blue) and hydrophobic (gray) intermolecular interactions involving the same helix.

In TRMT10C, the so-called *SpoU–TrmD* (SPOUT, class IV of SAM-dependent MTases) fold of the protein consists of a central β-sheet flanked on both sides by α-helices, as well as four conserved motifs I–IV around the SAM-binding pocket^2,6^ (Figure 1b). Thus, far, TRMT10C is the only member of the Trm10 family that requires a partner to be active: the protein SDR5C1, belonging to the short chain dehydrogenase/reductase family of proteins.^1,7^ The *N* ^1^-methylation of purine is prevalent in human mitochondrial tRNAs, occurring on 19 tRNAs out of 22,^8^ ensuring the stabilization of the L-shaped conformation of mitochondrial tRNAs.^9^ In the complex, A/G9 is “flipped out” from the tRNA body (Figure 1c) for methylation: The tRNA backbone around positions 8–10 bends 30–60°, creating a kink that exposes the nucleobase.^4^

Besides this tRNA MTase activity, the TRMT10C/SDR5C1 subcomplex is involved in mitochondrial tRNA maturation steps essential for mitochondrial translation, i.e., 5′-leader processing by PRORP (protein-only RNase P catalytic subunit), 3′-trailer processing by ELAC2 (zinc phosphodiesterase ELAC protein 2), and 3′-CCA addition by TRNT1 (tRNA nucleotidyltransferase 1).^3,4^ The tRNA methyltransferase complex TRMT10C/SDR5C1 compensates for the mitochondrial pre-tRNA structural alteration to present the pre-tRNA to maturation enzymes.^4^

A long helix in the N-terminal domain (NTD) of TRMT10C establishes interactions from the anticodon to the T-loops of the L-shaped pre-tRNA (Figure 1a).^4,9^ This arrangement exposes the acceptor and T arms of the mt-pre-tRNA, particularly the T loop, for interactions with the maturation enzymes.^4,9^ Comparisons with predicted structures of Trm10-family enzymes in complex with tRNAs confirm that their NTDs adopt a similar fold in eukaryotes.^9^ Compared with archaeal Trm10 enzymes, the TRMT10C NTD has an entirely different three-dimensional fold. Both archaeal Trm10 proteins and TRMT10C use their NTDs during substrate recognition, but the interacting residues are completely different.^10^

This solvent-exposed NTD α-helix (**NTDαh**) consists of 41 residues (residues 126–166), is highly positively charged (+11) with as many as 15 basic residues (37%, Chart 1). Some of the latter interact with the negatively charged phosphate backbone of the pre-tRNA,^4,10^ while the others interact with the other residues of TRMT10C NTD or they are exposed to solvent (Figure 1d,e). This helix is required for the binding of pre-tRNAs and its deletion abolishes the MTase activity of TRMT10C.^4^ There are currently no disease-causing mutations that have been definitively mapped to the NTD helix. However, recent functional mutagenesis studies^11^ have shown that the NTD helix is essential for activity. Several mutations have been shown in vitro to affect function. These mutations include: (i) mutations of seven Lys to Ala at positions 130, 131, 139, 141, 143, 149, and 150, which severely impair the cleavage of mtRNase P­(∼650 to 850 fold) and methylation activity (∼100-fold); (ii) the N126A and K129A substitutions, which decrease the cleavage activity of mtRNase P (6–11-fold) and methylation activity (∼10-fold). This suggests that this region contributes to proper tRNA positioning.

**Chart 1:**
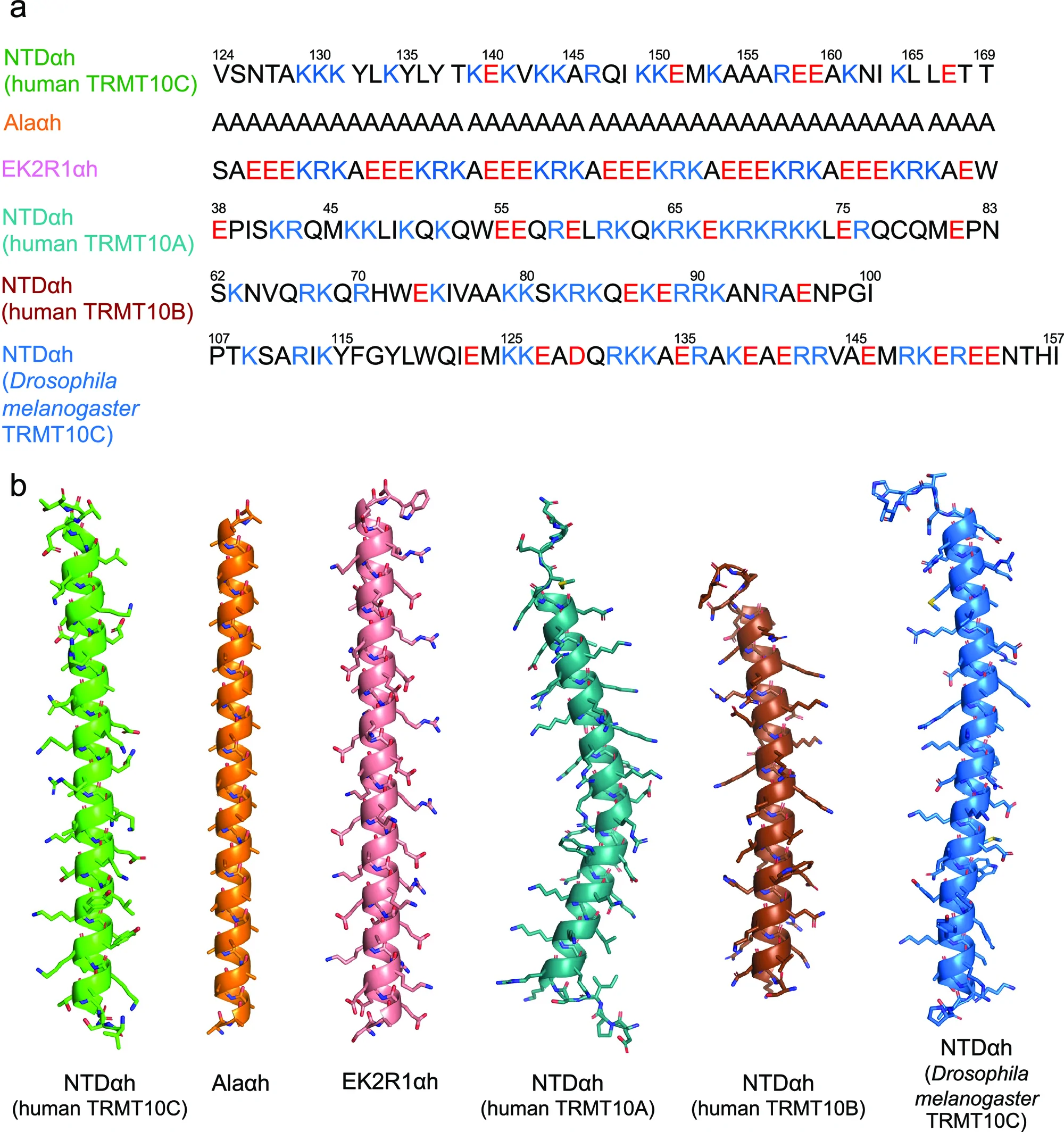
(a) Sequences of the human TRMT10C **NTDαh**, **Alaαh**, **EK2R1αh**, human TRMT10A NTDαh, human TRMT10B NTDαh, and *Drosophila melanogaster* TRMT10C NTDαh used in our simulations. The positively and negatively charged residues are indicated by blue and red letters, respectively. (b) Starting structures of those helices

**NTDαh** flexibility could be important for the function of the enzyme, as it could assist the proper position of the different tRNAs for catalysis by guiding the tRNA into a correct orientation toward SAM and/or by facilitating A/G9 flipping. To gain insights on the structural dynamics of the helix, we conducted a comparative molecular dynamics (MD) study of the helix in explicit solvent, alongside two helices of identical length but distinct sequences. The simulations were based on the Amber14SB force field^12^ with TIP3P water model^13^ and Joung-Cheatham TIP3P ion parameters^14^ (Amber14SB+TIP3P hereafter). First, we compared our results with those of the relatively rigid AEEEKRK repeat helix (**EK2R1αh**)^15^ (Chart 1). This stems from the Glu and Lys/Arg salt bridges in its AEEEKRK repeats which stabilize the backbone and limit solvent-induced distortions.^15,16^ Second, we examined a polyalanine helix (Alaαh, Chart 1) as a control case, providing a reference for the behavior of a helix with a uniform, uncharged sequence composition. To assess the robustness of the results to changes in the force-field model, we performed additional simulations for **NTDαh, Alaαh** and **EK2R1αh** using two other force fields, namely: (i) The Amber14SB force field,^12^ along with the OPC water model^17^ and the monovalent ion parameters developed by Sengupta et al.^18^ (Amber14SB+OPC hereafter); This force field provides us with information on the effect of changing the water potential. (ii) The CHARMM36m force field, along with the CHARMM-modified TIP3P water model and the CHARMM36 ion parameters^19^ (CHARMM+TIP3P hereafter). Finally, to assess whether the observed trends are generalizable across the family, we expanded our data set to include helical analyses of additional Trm10 homologues. Specifically, we performed MD simulations as above for three additional helices belonging to the Trm10 family: the human TRMT10A NTDαh, the human TRMT10B NTDαh, and the *Drosophila melanogaster* TRMT10C NTDαh (Chart 1).

## Computational Details

In this study, we focus on human TRMT10C NTDαh (**NTDαh** hereafter) in solution. The helix was extracted from the complex of the human mitochondrial RNase P with pre-tRNA^His‑Ser^, composed of TRMT10C­(SAH)/SDR5C1­(NADH)/pre-tRNA^His‑Ser^/PRORP_D479A_ (PDBid 8CBK^4^). To prevent artificial distortions of the terminal residues of the helices in solution due to truncation, we included a few residues of the protein to the termini (see Chart 1), for a total of 46 residues. The other two helices were made with the same length. They were built by “Peptide helix builder in J­(S)­mol”.^20^ The C-terminal amide NHCH3 (NME) and N-terminal acetyl COCH3 (ACE) capped the helices. Maestro (Schrodinger)^21^ was used to add the hydrogen atoms. The three helices were placed at the center of a 11.5 × 11.5 × 11.5 nm^3^ water box. Na^+^ ions and Cl^–^ were added to neutralize these systems and to achieve a concentration of 50 mM. Such concentration was selected to provide a low-to-moderate salt environment consistent with experimentally characterized conditions for human mitochondrial RNase P-related complexes. In particular, 50 mM NaCl has been used for the functional reconstitution of the human mitochondrial RNase P complex containing TRMT10C, SDR5C1, and PRORP.^22^ More recently, the TRMT10C/SDR5C1 complex was also assembled in 50 mM NaCl for structural characterization of the mitochondrial RNase Z complex.^23^ We therefore chose 50 mM ionic strength as a biologically and experimentally motivated condition while retaining appreciable electrostatic interactions, which are particularly relevant for the conformational dynamics of the charged TRMT10C N-terminal helix and its interactions with RNA.

In addition to human **NTDαh**, we performed simulations of NTDαh segments from other members of the Trm10 family, namely human TRMT10A (41 residues, from 40 to 80), human TRMT10B (37 residues, from 64 to 100), and *Drosophila melanogaster* TRMT10C (46 residues, from 109 to 154). As experimental structures for these regions are not available, we used AlphaFold-predicted models^24,25^ as starting structures. The corresponding UniProt IDs are Q8TBZ6, Q6PF06, and Q7JUX9, respectively. The confidence scores of the AlphaFold predictions for these structures turned out to be from high (90 > pLDDT > 70) to very high (pLDDT > 90). Following the setup used for human **NTDαh**, we included a few additional protein residues at both termini (Chart 1) and capped the termini with NME and ACE. We also used the same water-box dimensions and ion concentration as for the human TRMT10C NTDαh, except for human TRMT10B NTDαh, for which a smaller water box (10.5 × 10.5 × 10.5 nm^3^) was used because this helix is shorter than any other simulated here.

We ran simulations on all helices using the Amber14SB force field,^12^ TIP3P water model,^13^ and Joung-Cheatham TIP3P parameters^14^ for the helix, water molecules, and the ions, respectively. We called this combination Amber14SB+TIP3P.

The **NTDαh** and **EK2R1αh** helices are unusually long, highly charged α-helices. We therefore chose a slightly longer cutoff for nonbonded interactions (1.2 nm) to accurately treat the real-space electrostatics involving the numerous charged side chains, instead of 1.0 nm as usually set for the AMBER14SB force field.^12^ The same cutoff was also used for van der Waals interactions. For long-range electrostatic interactions, we employed the Particle Mesh Ewald (PME) method^26,27^ with grid spacing of 1.6 Å. The equations of motion were solved using the leapfrog algorithm with the integration time step of 2 fs. The LINCS algorithm^28^ was used to constrain all bonds involving hydrogen atoms.

Starting from the solvated system (after adding ions), the minimization steps were performed using steepest descent^29^ followed by conjugate gradient^30^ algorithms. Next, the system was heated gradually from 5 to 298 K during 5 ns using a simulated annealing procedure. The system was then equilibrated for 1 ns at the reference temperature 298 K and pressure 1 bar (NPT ensemble), using Velocity Rescale (V-rescale) temperature coupling^31^ and C-rescale pressure coupling.^32^ In the annealing and equilibrating steps, harmonic restraints with the spring constant of 240 kcal mol^–1^ nm^–2^ were applied to all non-hydrogen atoms of the helix along all three coordinates. These restraints were released in the production run afterward. The production run was performed at the same reference temperature and pressure as the previous step, using the Nose-Hoover thermostat^33–35^ and Parrinello–Rahman barostat.^36^ For the Nose-Hoover thermostat and V-scale thermostat, the time constant was set to 0.1 ps. For the barostat, isotropic pressure coupling was used with a time constant of 2 ps and the water’s isothermal compressibility of 4.5 × 10^–5^ bar^–1^.

For each system, we performed five independent 1000 ns production simulations with each replica initiated from a different set of random initial velocities. We discarded the first 20 ns of each replica in the trajectory analysis. The GROMACS/2024.3 code^37^ was used to perform all MD simulations and analyze trajectories.

For **NTDαh**, **Alaαh**, and **EK2R1αh**, we performed additional MD simulations in exactly the same conditions as above but using the following force fields: (i) the Amber14SB force field,^12^ along with the OPC water model^17^ and the monovalent ion parameters developed by Sengupta et al.^18^ (Amber14SB+OPC); (ii) the CHARMM36m force field, along with the CHARMM-modified TIP3P water model and the CHARMM36 ion parameters^19^ (CHARMM+TIP3P).

The representative structure of each helix is the central structure of the largest cluster obtained from cluster analysis in GROMACS.^37^ The solvent-accessible surface area (SASA) analysis was performed by gmx sasa in GROMACS.^37^ In the secondary structure analysis, the secondary structure was assigned using the STRIDE algorithm via the VMD Timeline plugin.^38^ The residue-wise bending angles were analyzed by Bendix.^39^ The representative structures of the helices in the bending-angle analysis were colored by an RGB heatmap color scheme from the Bendix^39^ plugin in VMD.^40^ The flexibility of the backbone of the helices was quantified by the Protein Angular Dispersion (PAD) analysis using the T-pad tool.^41^ The higher the value of the PAD of a backbone unit,^41^ the greater its flexibility.^41^ We also performed principal component analysis (PCA) and salt-bridge analysis on MD trajectories of all helices.

Besides Table 1 and Figures 1–6, additional results supporting this work can be found in the SI (Figures S1–S97 and Tables S1–S23). These include secondary structure analysis, bending-angle analysis, PAD analysis, SASA analysis, timeline of key observables, clustering analysis, PCA analysis, and salt-bridge analysis of all helices.

**Table 1:** Secondary Structure Composition over the Cumulative Trajectory of **NTDαh, Alaαh**, and **EK2R1αh** (with Amber14SB+TIP3P)^a^

	NTDαh	Alaαh	EK2R1αh
α-helix	78.18 (9.26)%	69.51 (14.36)%	87.86 (7.03)%
3–10 helix	3.25 (4.73)%	2.13 (3.77)%	1.00 (2.73)%
extended conformation	0.00%	0.08 (0.83)%	0.00%
isolated bridge	0.00%	0.14 (0.76)%	0.00%
turn	7.68 (6.96)%	11.86 (10.39)%	2.15 (4.75)%
coil	10.89 (5.02)%	16.28 (7.32)%	8.99 (3.99)%

a The average and standard deviation (in parentheses) of each composition across the trajectory are shown.

## Results and Discussion

Residue-level secondary structure analysis^38^ revealed marked differences in α-helical propensity and structural homogeneity among the three peptides (Figures 2 and S1–S3 and Table 1).

**NTDαh** retained an overall α-helical character, yet it showed a high heterogeneity along the sequence. The α-helical content drops (to ∼40–70%) in the regions around specific alanine residues (Ala145, Ala154–156), accompanied by substantial increases in turn and coil contributions, indicative of partial α-helical destabilization and segmental disorder. However, the region of residues 132–139 does show an α-helical content of ∼100%, suggesting localized rigidity (Figure 2a,d).

**Figure 2:**
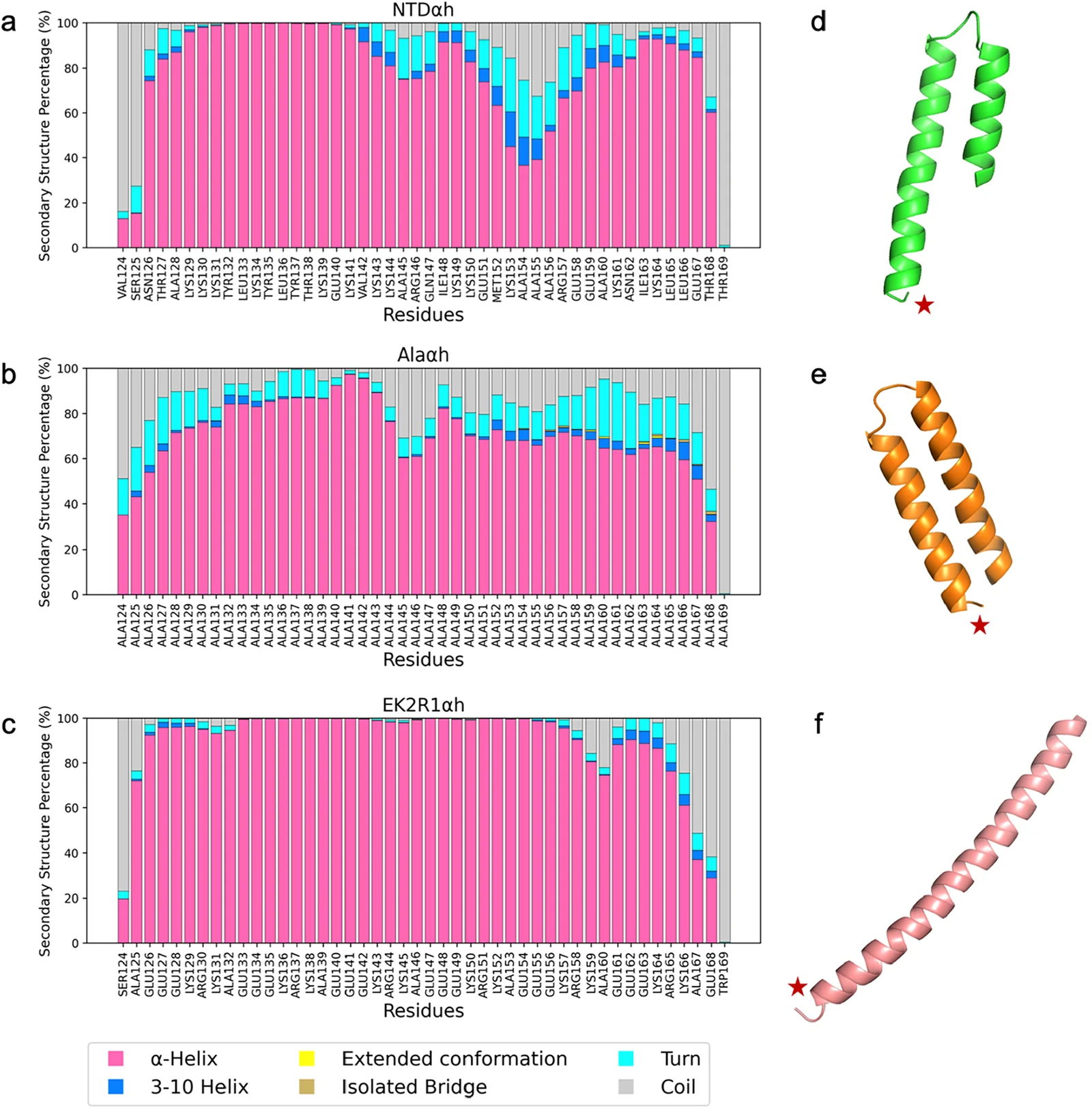
(a–c) Secondary structure composition per residue along the cumulative trajectories (with Amber14SB+TIP3P) of **NTDαh, Alaαh** and **EK2R1αh**, respectively. (d–f) Representative structure (the central structure of the largest cluster obtained from cluster analysis in GROMACS) of **NTDαh, Alaαh**, and **EK2R1αh**, respectively. The red star highlights the N-terminus.

**Alaαh** exhibited the lowest α-helical propensity, with the total α-helical composition over the cumulative trajectory around ∼70% (to be compared to **NTDαh** ∼80% and **EK2R1αh** ∼90%), respectively (Table 1). Along the sequence, except at the termini, the α-helical content is generally quite uniform, ranging between ∼60–80%, indicative of similar probabilities of forming turns/coils across all residues (Figure 2b,e).

**EK2R1αh** displayed a consistently high α-helical content across nearly its entire sequence, with most residues exhibiting α-helical content approaching ∼100%, with the exception of some residues close to the termini. This indicates a highly stable helical conformation (Figure 2c,f).

Collectively, these results demonstrate that sequence composition strongly modulates α-helix stability, with **EK2R1αh** exhibiting the highest structural order, followed by **NTDαh**, and **Alaαh** showing the greatest degree of conformational flexibility. **NTDαh** samples the broadest distribution of α-helical content during the simulations (Figure 2), indicating that it interconverts among conformations with different extents of helicity more frequently than the other two systems.

Next, we investigate the bending of the helices^39^ during the MD trajectories (Figures 3 and S4–S6).

**Figure 3:**
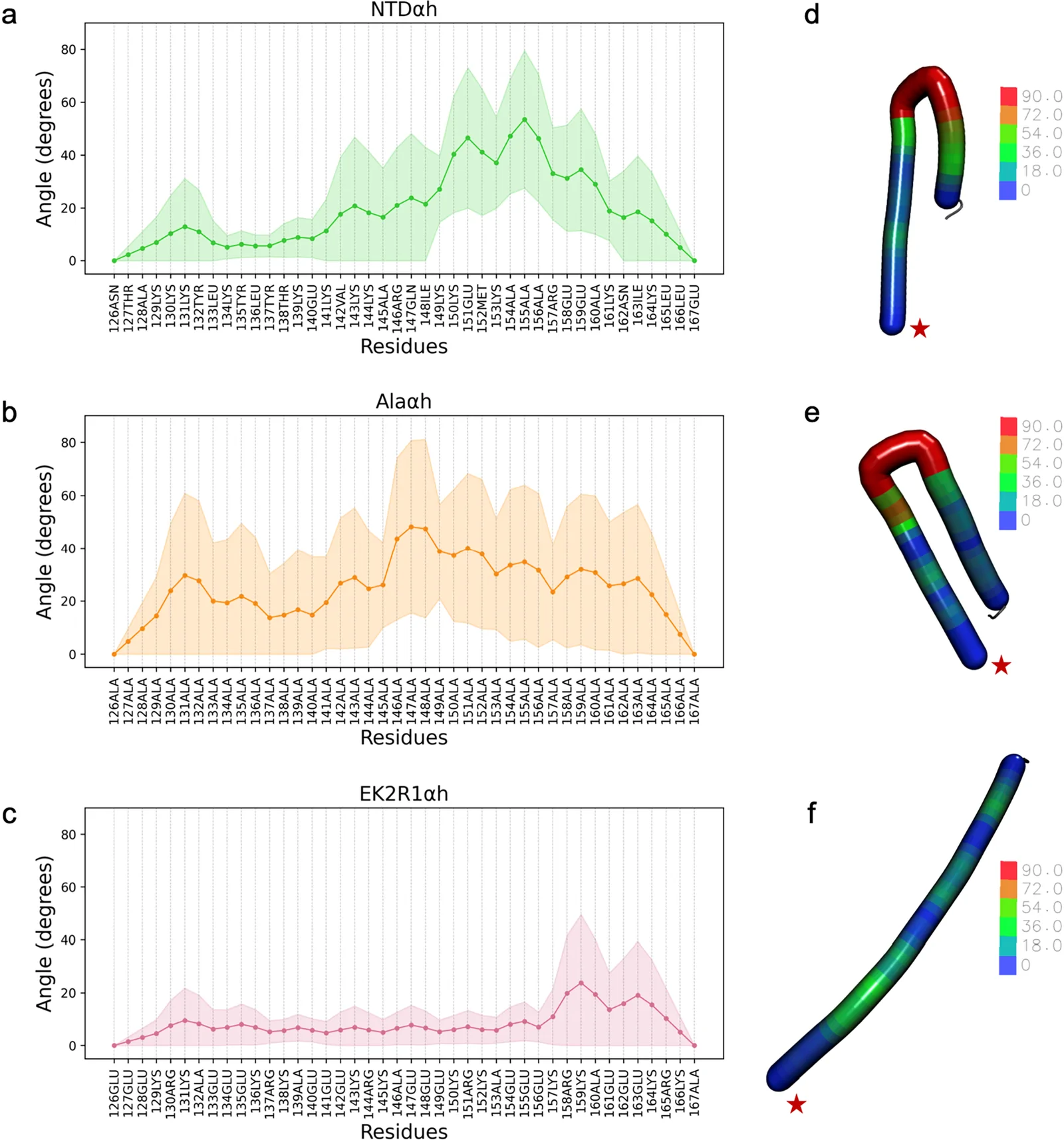
(a–c) Αverage bending angles, along with their standard deviations, along the cumulative trajectories (with Amber14SB+TIP3P) of **NTDαh, Alaαh**, and **EK2R1αh**, respectively. (d–f) Representative structures of the helices. The red color indicates the areas of highest helix axis angle, green indicates intermediate angle magnitudes, and blue indicates angles close to or at 0°. The red star highlights the N-terminus.

**NTDαh** exhibited a distinctly heterogeneous bending-angle distribution: Residues 132 to 139 show small average bending angles (∼5–15°) with minor fluctuations (Figure 3a). This corresponds to the residues with high composition of α-helix observed earlier (Figure 2a). Bending angles increase progressively toward the residues 150 to 160 and reach values of ∼40–60°, accompanied by broad fluctuations (Figure 3a). These regions of elevated curvature coincide with the reduced α-helical content and increased turn/coil contributions (Figure 2a). The cartoon in Figure 3d shows the bend around positions 150–160.

**Alaαh** displayed moderate average bending angles (∼20–50°) but with markedly large deviation distributed across the sequence (Figure 3b). This is consistent with its reduced α-helical stability and with its increased occurrence of turns (Figure 2b). Thus, the helix exhibits distortions and flexibility along its entire sequence, and it can bend at any position (Figure 3e is an example).

**EK2R1αh** showed low average bending angles throughout the sequence, often below ∼20° (Figure 3c). This minimal curvature aligns with its near-complete α-helical content and absence of significant coil or turn regions (Figure 2c). This is a straight helix (Figure 3f).

Next, we analyzed the flexibility of helices at the residue level. For this, we calculated the Protein Angular Dispersion (PAD)^41^ for each residue (Figures 4 and S7–S9). The PAD values correlate with flexibility: the higher the PAD value, the greater the flexibility.

**Figure 4:**
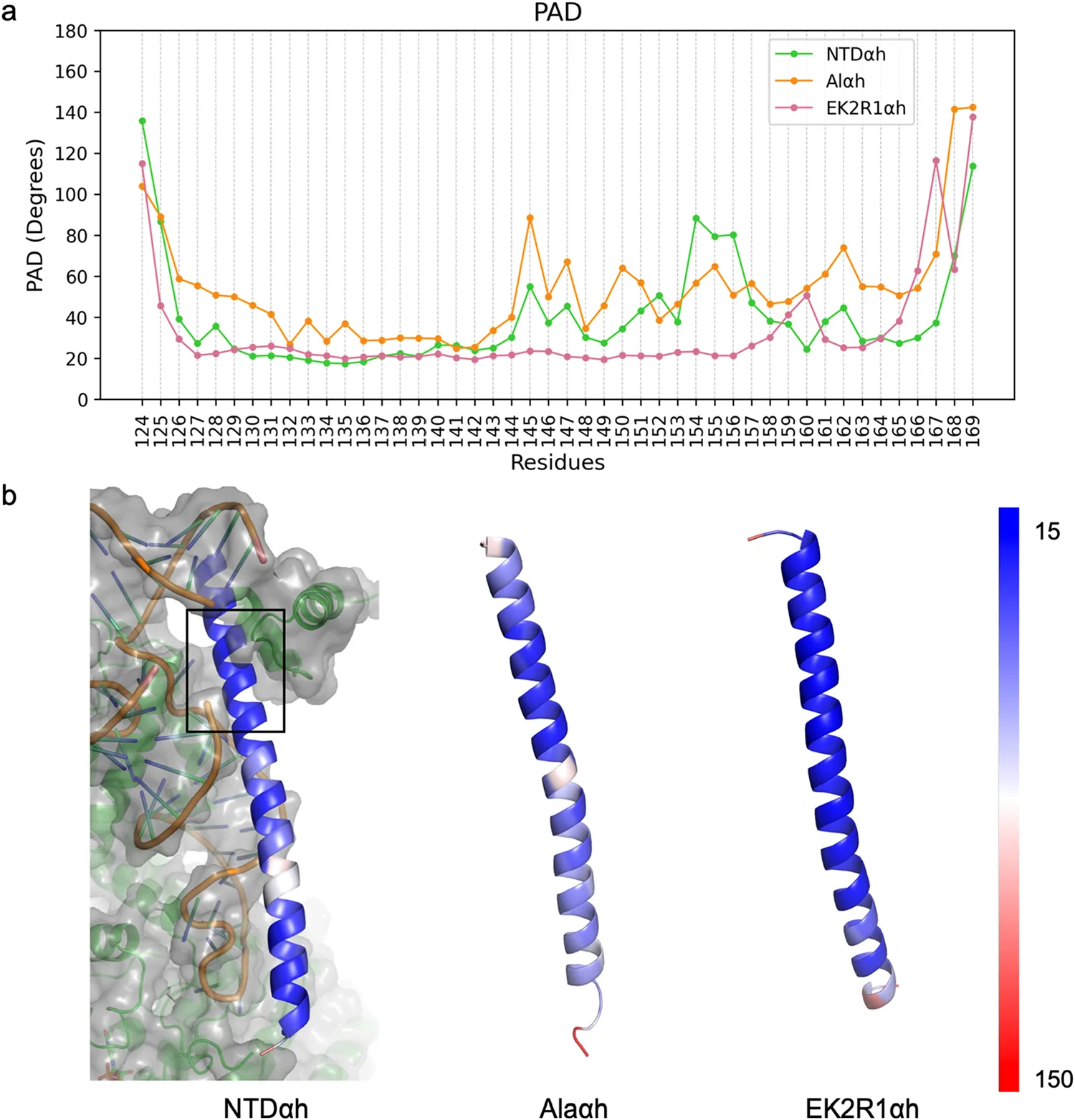
(a) PAD values calculated for each residue over the cumulative trajectories (with Amber14SB+TIP3P) of the three helices **NTDαh, Alaαh**, and **EK2R1αh**. The PAD values correlate with flexibility: higher is the PAD value, larger is the flexibility. (b) Latter are colored according to the PAD values. For **NTDαh**, the α-helical region (residues 132–139, indicated by the black outline) features low PAD values. Part of the complex surrounding **NTDαh** are shown as cartoon and surface representation. The figure was made using PyMOL.^42^

The flexibility of **NTDαh** regions with large bending angles and a high content of turns/coils is relatively high, as shown by a plot of the PAD values^41^ for each residue, calculated from the cumulative trajectories (Figure 4). In contrast, the region that does not bend during MD simulations (residues 132–139) features relatively low PAD values. **Alaαh** residues feature relatively large flexibility (Figure 4), as this helix can bend at any position while **EK2R1αh** residues exhibit relatively low flexibility except for the termini regions and for residues 157–161 (Figure 4). Note that this 98-residue helix^15^ was truncated to make it as long as **NTDαh**, thus, the terminal residues in our study are not the terminal residues of the helices and hence their large flexibility is most likely due to this truncation.

Taking our results together, we suggest that the increased α-helical content is associated with reduced bending and, as expected, enhanced structural rigidity; disruptions in helicity lead to increased curvature and conformational flexibility. We can identify two regions of the helices: bending region, which features high flexibility, high turns/coils composition and large bending angles, and α-helical region, which has low flexibility, ∼100% α-helical composition and small bending angles.

**Alaαh** contains only bending regions; **EK2R1αh** and **NTDαh** feature α-helical regions only from residues 133 to 154 and from 132 to 139, respectively. Strikingly, **NTDαh**’s α-helical region is the part that is accommodated into the “cavity” formed by the T-loop of the tRNA and the nearby part of TRMT10C NTD in the complex (Figures 1 and 4b): residues in this region form not only polar interactions (hydrogen bonds/salt bridges/π-cation) but also hydrophobic interactions with the TRMT10C NTD and the tRNA (Figure 1d,e), while the region from residues 142 to 166 forms only polar interactions with the tRNA on one side and is exposed to water on the other side (Figure 1d,e).

The solvent-accessible surface area (SASA) per residue was also investigated (Figures 5 and S10–S12). EK2R1αh’s significant exposure to the solvent (SASA ∼0.5–1.5 nm^2^, Figure 5a) stabilizes the predominantly hydrophilic sequence of the helix shown by low hydrophobicity values in Figure 5b, as those of the highly charged AEEEKRK repeated sequence (Figure 6). This stable structure is demonstrated by the low SASA fluctuations (standard deviations). **Alaαh** shows relatively low and uniform solvent exposure (SASA values ∼0.5–0.8 nm^2^, Figure 5a). This is caused by its uniformly hydrophobic sequence (Figure 5b): the complete lack of charged residues (Figure 6) favors tight packing. However, the SASA fluctuations are high, indicating a flexible structure in solution. **NTDαh** showed the highest variability in SASA, with residue-specific fluctuations, driven by its heterogeneity in charge distribution and hydrophobicity profile. The regions with high SASA average values and fluctuations (with peaks in SASA at Arg146, Lys153, and Arg157) correspond to the regions enriched in polar or charged residues (Figures 5 and 6). These are bending regions. The regions with lower SASA values and small fluctuations are associated with more hydrophobic segments (Figures 5 and 6), especially from residue 132 to 139, that also corresponds to the rigid α-helical region of **NTDαh**. Thus, the SASA profiles are strongly modulated by the underlying sequence properties. **EK2R1αh** combines electrostatic patterning and hydrophilicity to form a rigid yet solvent-accessible helix. **Alaαh** adopts a more compact, less exposed conformation and high fluctuations driven by hydrophobicity. In **NTDαh**, its mixed sequence composition leads to the heterogeneous solvent exposure and fluctuations consistent with its flexibility. In conclusion, the SASA profiles of **NTDαh** and **EK2R1αh** appear to reflect the distinct flexibilities of the two helices. **NTDαh** displays heterogeneous solvent exposure along its length (Figure 5a), consistent with its nonuniform flexibility in solution (Figure 4). In contrast, **EK2R1αh** exhibits relatively small variations in SASA along the helix (Figure 5a), consistent with its greater overall rigidity (Chart 1 and Figure 4).

**Figure 5:**
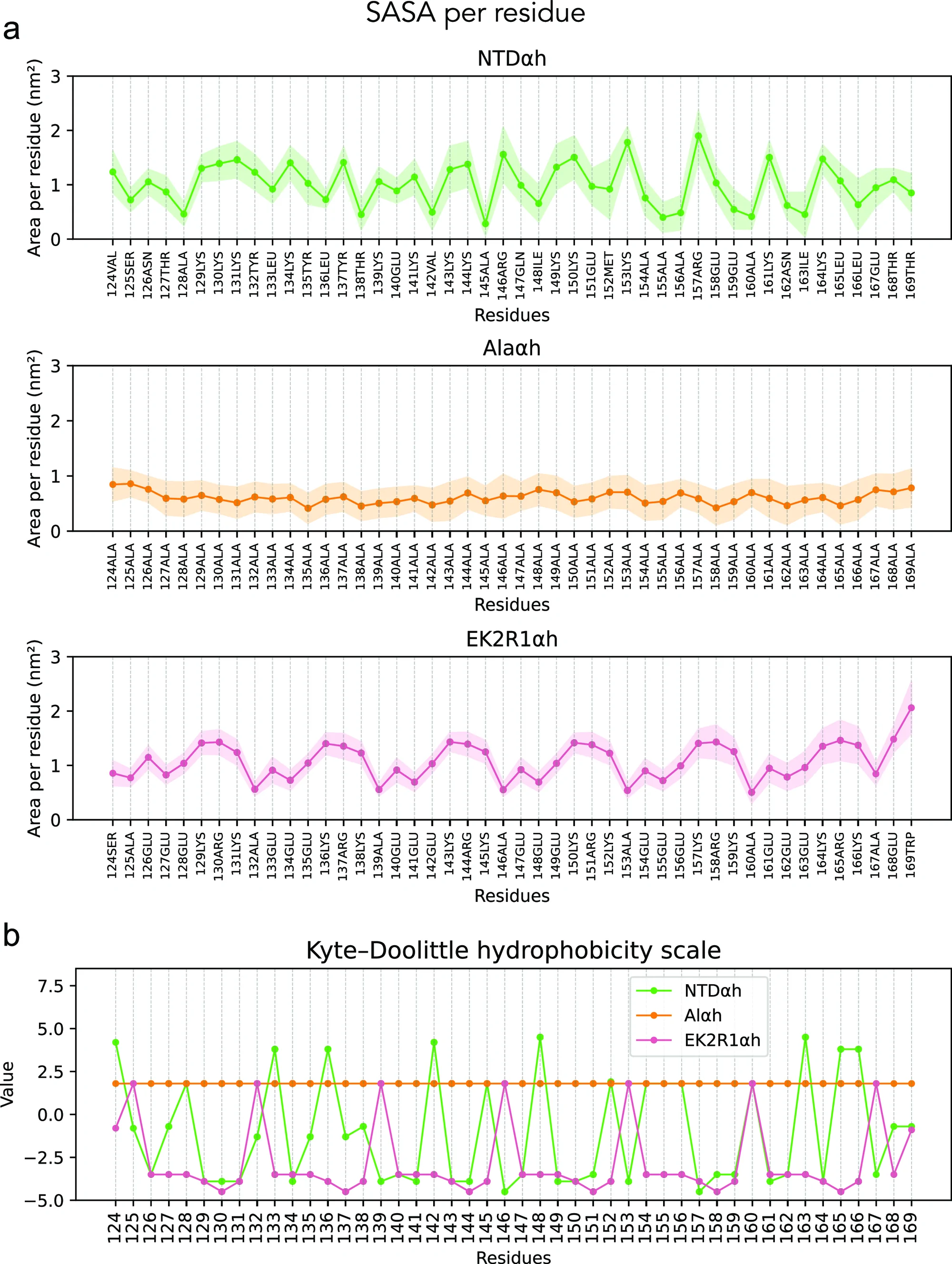
(a) Average and standard deviation of the solvent-accessible surface areas per residue over the cumulative trajectories (with Amber14SB+TIP3P) of **NTDαh, Alaαh**, and **EK2R1αh**. (b) Hydrophobicity of each residue (using the Kyte-Doolittle scale^43^) of **NTDαh, Alaαh**, and **EK2R1αh**.

**Figure 6:**
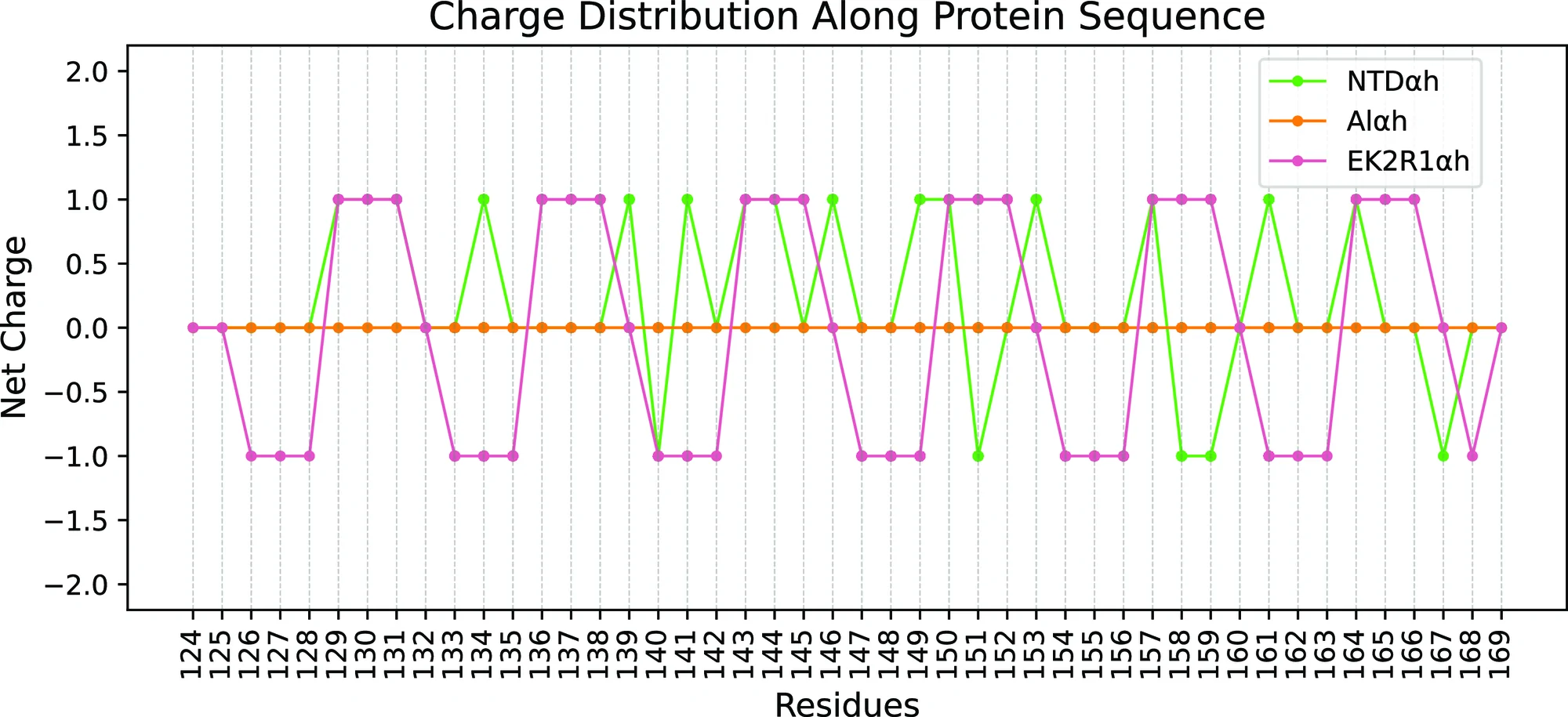
Charge distribution along the sequences of **NTDαh, Alaαh**, and **EK2R1αh**. The total charge of each residue is shown.

A clustering analysis reveals distinct conformational landscapes for each helix (see SI, Figures S13–S15 and Tables S1–S3). **EK2R1αh** is characterized by a single dominant cluster accounting for 73.6% of the trajectory, indicating a highly stable conformational ensemble. In contrast, **Alaαh** exhibits the greatest conformational heterogeneity, with the two largest clusters account for only 20.3% and 10.6% of the trajectory, respectively, while all remaining clusters each contribute less than 5%, reflecting the absence of a predominant structure. **NTDαh** displays intermediate behavior: Its three most populated clusters represent 34.6%, 11.0%, and 11.0% of the trajectory, together accounting for 56.6% of the sampled conformations, whereas all remaining clusters each have populations below 10%. Overall, the clustering analysis suggests that **EK2R1αh** is the most conformationally rigid system, as evidenced by the predominance of a single structural state, whereas **Alaαh** is the most flexible, sampling a broad and diverse conformational ensemble without a dominant structure. **NTDαh** exhibits intermediate conformational plasticity. These findings are fully consistent with the results of the secondary-structure, bending-angle, PAD and SASA analyses.

A principal component analysis (PCA) on MD trajectories of all helices allowed us to construct free energy landscapes along the first two principal components (see SI, Figures S16–S21). In **EK2R1αh**, we find only one basin present in the free energy landscape and its representative conformation as a straight helix. This suggests that **EK2R1αh** has high structural stability and retains its α-helical structure during almost all the simulation time. On the other hand, **Alaαh** shows a much more scattered free energy landscape. This means that this helix forms many different conformations as a result of high flexibility. The free energy landscape of **NTDαh** also shows few basins but they are less scattered than the ones of **Alaαh**, indicating its flexibility is higher than **EK2R1αh** but lower than **Alaαh**, consistently with other analyses.

MD simulations with Amber14SB+OPC yielded very similar structural features and conformational fluctuations to those observed with Amber14SB+TIP3P (see SI, Figures S22–S46 and Tables S4–S7). Specifically, **NTDαh** remained a stable α-helical segment embedded between flexible regions, **EK2R1αh** was a highly stable helix with low flexibility, and **Alaαh** was highly flexible. Thus, the relative ranking of flexibility across the three helices was preserved. We conclude that changing the water potential does not largely affect our results.

MD simulations with CHARMM36m+TIP3P yielded similar structural characteristics and fluctuations for **NTDαh** and **EK2R1αh**. In contrast, **Alaαh** exhibited a higher helical content than in the other two simulations, reaching 85%, compared with 70% with Amber14SB+TIP3P and 60% with Amber14SB+OPC (see SI, Figures S47–S71 and Tables S8–S11). Thus, the trend observed using Amber14SB+OPC and Amber14SB+TIP3P is maintained for **NTDαh** and **EK2R1αh** but not for **Alaαh**.

Similar flexibility profiles of **NTDαh** were observed for the human Trm10 family members TRMT10A and TRMT10B (see SI, Figures S72–S97 and Tables S12–S15). Thus, this expanded analysis supports the conservation of the observed flexibility trends across the family and provides further evidence for a robust relationship between complex function and helical flexibility.

## Conclusions

We have presented a comparative molecular dynamics study on different α-helical peptides in aqueous solution, revealing how sequence composition governs helix stability, bending, flexibility, and solvent exposure. The objective of the present study is to isolate the intrinsic conformational properties encoded by the helix sequence itself. By comparing the native helix with polyA and polyE/K/R variants under identical conditions, we sought to disentangle sequence-dependent effects from those arising from the surrounding molecular environment of the mtRNase P complex.

As expected, in all our analyses of the helices, the residues featuring high helical content show lower flexibility and low bending angles than residues forming random coils. Decorating the helix with a repeated AEEEKRK sequence makes **EK2R1αh** markedly more rigid with all the force fields analyzed here. The helix remains nearly fully α-helical with minimal bending, except for a localized region around residues 157–161. This reduced flexibility is accompanied by low SASA fluctuations, indicating a stable and well-defined structure in aqueous solution. The oppositely charged residues promote intrahelical salt-bridge formation (Table S16) and stabilize the helical structure (while in **NTDαh**, there are fewer salt bridges, Tables S17–S23). Together, these features result in a rigid, straight helix with minimal conformational heterogeneity.

With all the force fields analyzed here, **NTDαh** samples a broader distribution of α-helical content during the simulations than the other two helices, indicating that it interconverts among conformations with different content of helicity more frequently than the other two systems. Bending occurs at multiple positions along the sequence, but not in all. The SASA fluctuations vary along the sequence. These features are caused by the helix’s mixed sequence composition, including alternating hydrophobic and charged residues. Notably, the region spanning residues 132–139 exhibits reduced bending and enhanced rigidity. This localized rigidity within an otherwise flexible **NTDαh** (which was also observed in other human Trm10 family members TRMT10A/B) may have functional implications: the short, stable α-helical segment is evolutionarily optimized to facilitate molecular recognition, particularly in binding to the cavity formed by the T-loop of tRNA and the N-terminal domain of TRMT10C. Such interactions would require a balance between global flexibility - allowing conformational adaptation - and local rigidity - ensuring precise structural complementarity. In this way, TRMT10C may rearrange easily upon conformational changes associated with tRNA binding and release, and with the different human mitochondrial tRNA sequences it modifies.

**Alaαh** exhibits more contrasting results. In the Amber14SB+OPC- and Amber14SB+TIP3P-based MD simulations, it is a structure with moderate and relatively uniformly distributed flexibility, consistent with its homogeneous composition and absence of charged residues. Bending occurs across the sequence without a strong positional preference, consistent with its homogeneous composition and absence of charged residues. However, in the CHARMM36m+TIP3P-based MD simulations, the helix appears to be less flexible and with a higher helical content.

Neither experimental nor computational studies of polyalanine helices as long as the one investigated here have been reported. The longest helices investigated contained less than half of the residues. So, we cannot establish which force field reproduces the behavior of the helix in solution. Specifically, to date, the longest polyalanine helices studied contain only Ala residues and were investigated computationally using the AMBER and CHARMM force fields, including two of the force fields employed in our study.^44^ Table S24 shows that the helical content obtained with the AMBER14SB+TIP3P and CHARMM36m+TIP3P force fields used here is lower than that observed in our simulations. This discrepancy may arise from the substantial difference in peptide length: the polyalanine helix investigated in the present study contains as many as 46 residues.

Given its homogeneous composition, the full-Ala helix is expected to display relatively uniform conformational behavior along its length. In particular, because Alaαh lacks charged residues and the alternating hydrophobic/charged pattern characteristic of **NTDαh**, bending and flexibility should not be strongly localized to specific sequence positions. This is indeed what we observe: **Alaαh** shows moderate flexibility and bending distributed across essentially the entire sequence, whereas **NTDαh** exhibits a heterogeneous profile, with a relatively rigid segment around residues 132–139 and more flexible, strongly bending regions toward residues 150–160. Thus, removal of sequence heterogeneity should produce a more spatially homogeneous dynamical behavior. The simulations support this interpretation, although the absolute degree of flexibility of **Alaαh** is force-field dependent.

In conclusion, we have characterized the intrinsic conformational landscape of the isolated TRMT10C N-terminal helix. Although interactions within the complete mtRNase P complex are expected to modulate this landscape, our results demonstrate that the helix sequence itself encodes substantial conformational plasticity, which may be important for tRNA recognition. Simulations of the complete TRMT10C–tRNA complex will therefore be important to establish how these intrinsic properties contribute to tRNA recognition and catalysis.

## Supplementary Material



## Data Availability

The raw data supporting all the findings of this study are available within the article. Data including force field parameters, starting structures, topology files, mdp files for MD simulations as well as the MD trajectories can be found in the Zenodo repository: 10.5281/zenodo.19882122.

## Notes

Timeline of key observables, Clustering analysis and PCA-based free energy analysis of NTDαh, Alaαh, EK2R1αh (Amber14SB+TIP3P-based MD). AMBER14SB + OPC-based MD simulations of NTDαh, Alaαh, EK2R1αh. CHARMM + TIP3P-based MD simulations of NTDαh, Alaαh, EK2R1αh. NTDαh helices from the Trm10 family using Amber14SB + TIP3P-based MD. Salt-bridge analysis. Molecular dynamics study of the 21-residue polyalanine peptide (PDF)

## Notes

We dedicate this work to Prof. Michael L. Klein, who gave seminal contributions to the statistical–mechanical development of simulation methods that support the modern free-energy landscape description of biomolecular dynamics.

## Abbreviations

MTases: methyltransferases
RNase: ribonuclease
TRMT10C: tRNA methyltransferase 10C
SDR5C1: short-chain dehydrogenase/reductase family 5C member 1
PRORP: protein-only RNase P catalytic subunit
ELAC2: zinc phosphodiesterase ELAC protein 2
TRNT1: tRNA nucleotidyltransferase 1
SAM: S-adenosyl-l-methionine
SAH: S-adenosylhomocysteine
NTD: N-terminal domain
PAD: protein angular dispersion
SASA: solvent-accessible surface area
MD: molecular dynamics
