# Supplementary material for: Flexibility of the TRMT10C N‑Terminal Domain Helix Explored by Molecular Dynamics Simulations
Source: J Phys Chem B. 2026 Oct 7;130(42):11103–14. doi: 10.1021/acs.jpcb.6c03249 (PMC13700100; doi:10.1021/acs.jpcb.6c03249)
Supplement: Supplementary file 1 [file jp6c03249_si_001.pdf]

Supporting Information

Flexibility of the TRMT10C N-terminal domain helix explored by molecular dynamics simulations

*Gia Linh Hoang[1], Giulia Rossetti[1,2,3], Carine Tisne[4], Paolo Carloni[1]**

[1] Institute for Neuroscience and Medicine (INM-9), Forschungszentrum Jülich, 52425 Jülich, Germany.

[2] Department of Neurology, Medical Faculty, RWTH Aachen University, 52074 Aachen, Germany.

[3] Jülich Supercomputing Centre (JSC), Forschungszentrum Jülich, 52425 Jülich, Germany.

[4] Université Paris Cité, CNRS, Institut de Biologie Physico-Chimique (IBPC), 75005 Paris, France.

1. Timeline of key observables (Amber14SB+TIP3P-based MD)

1.1. α-helical content

The α-helical content of **NTDαh** in the Amber14SB+TIP3P-based MD does not change significantly during the dynamics, except for replica 3, where the α helix content decreases up to 30% (Fig.S1). That of **Alaαh** decreases up to 20-50% during the dynamics (Fig. S2). That of **EK2R1αh** does not vary significantly (Fig. S3).

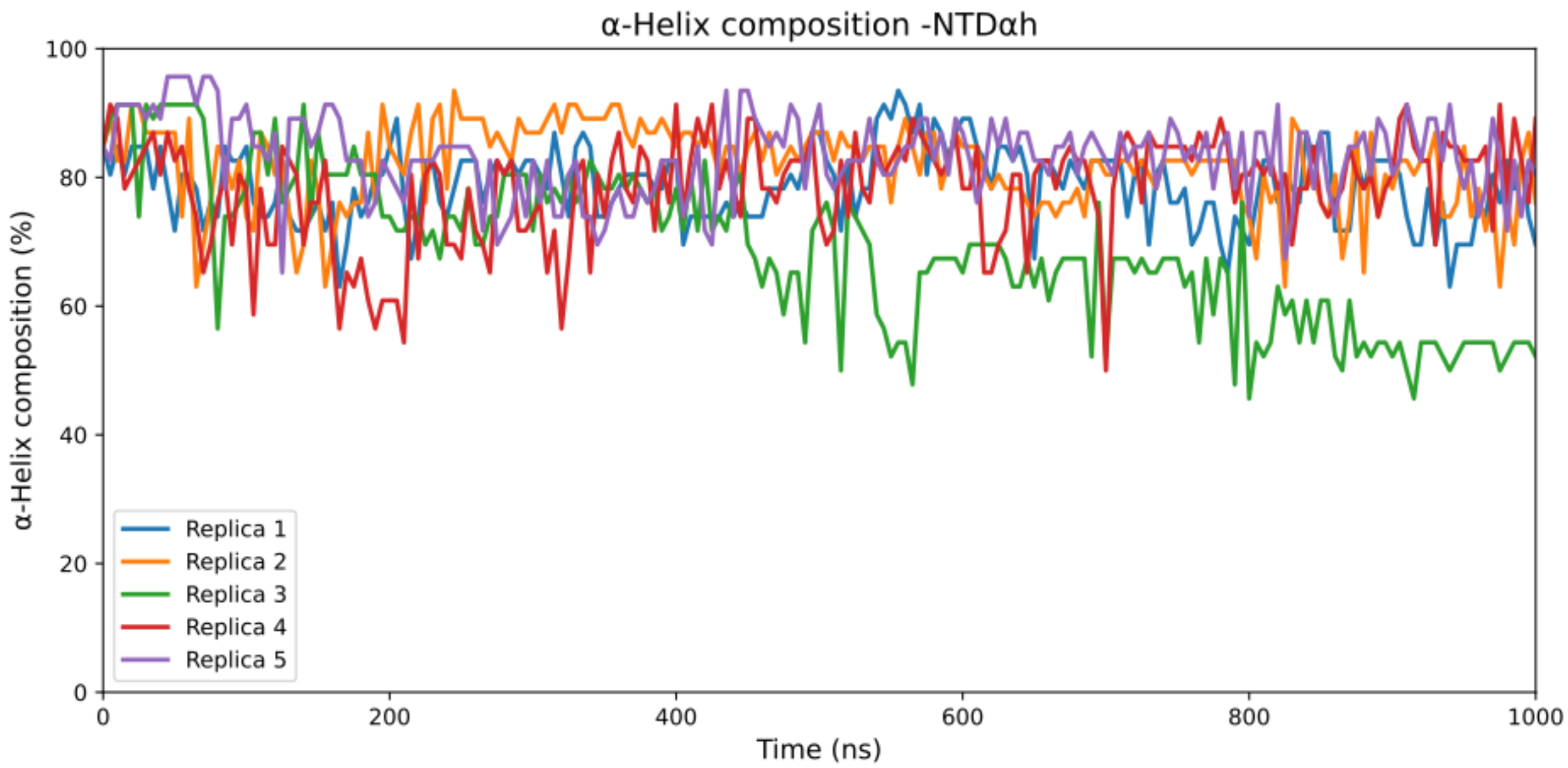

Figure S1. α-helix content of **NTDαh** in the Amber14SB+TIP3P-based MD (5 replicas), plotted as a function of simulated time.

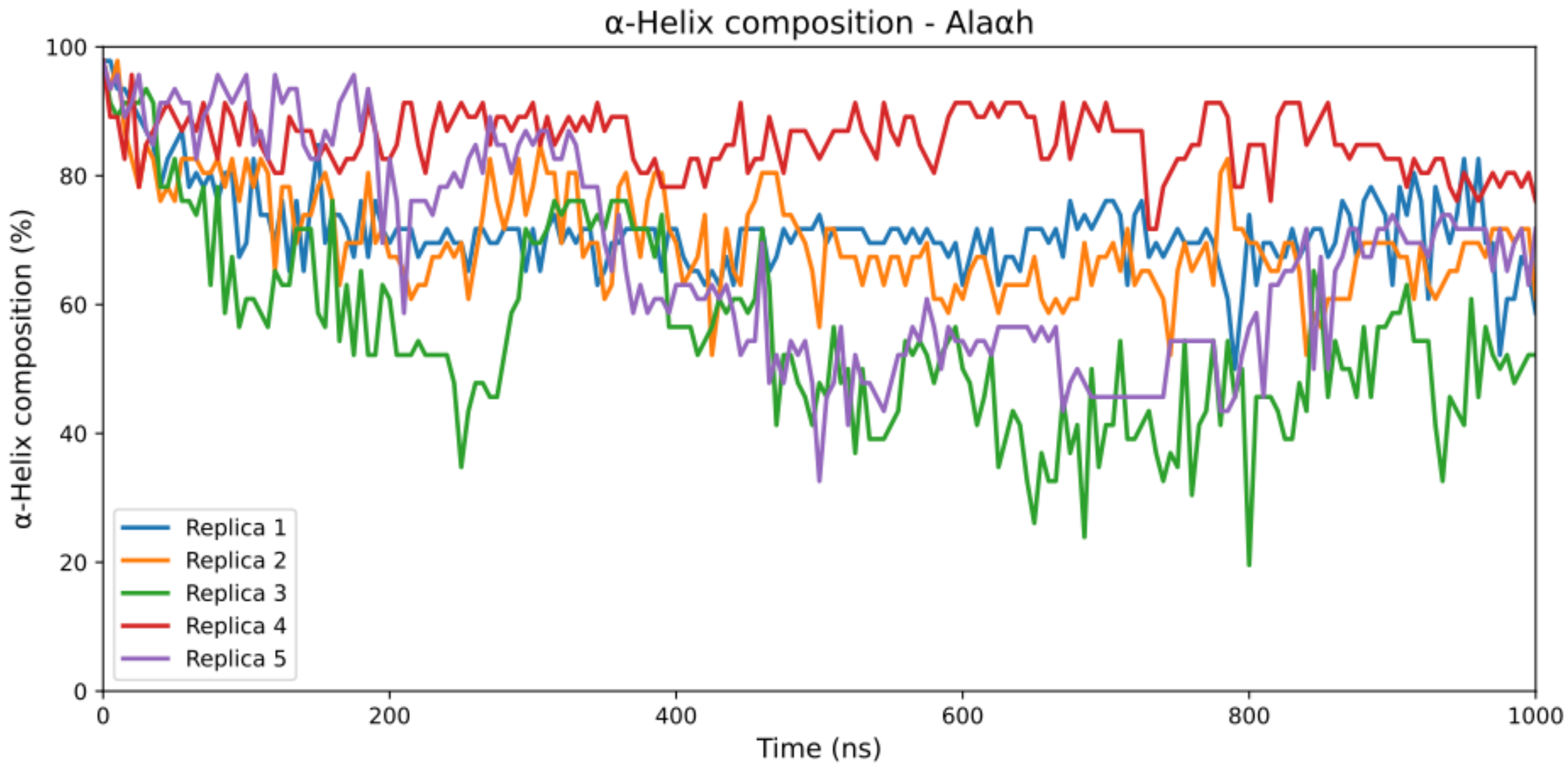

Figure S2. Same as Fig. S1 but for **Alaαh.**

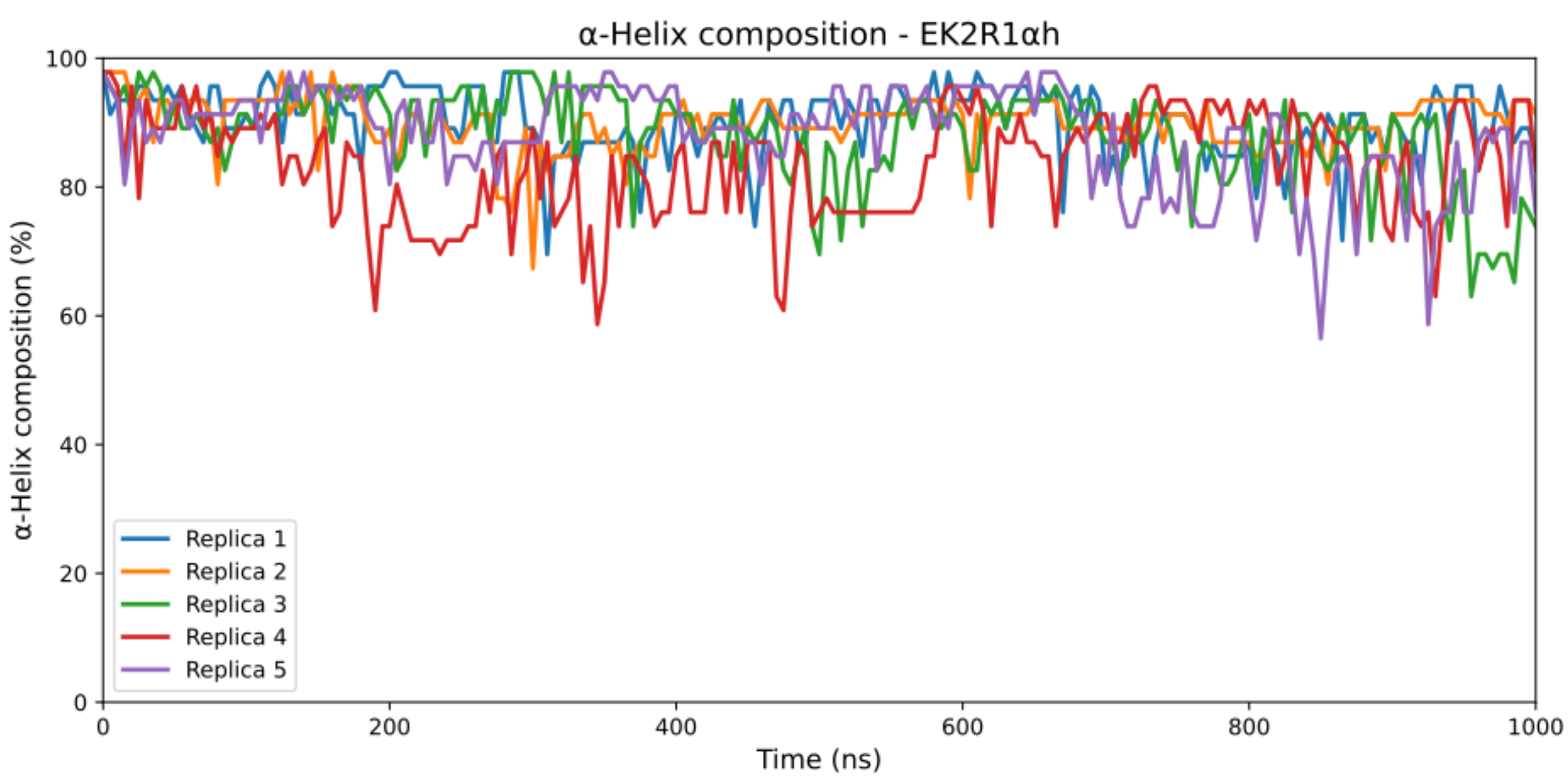

Figure S3. Same as Fig. S1 but for **EK2R1αh**.

1.2. Bending angles

The bending angles of each residue are calculated using the Bendix plugin[1] in VMD[2].

In the Amber14SB+TIP3P-based MD simulations of **NTDαh**, the bending angles increase for several residues. However, this was not the case for residues 132 to 139, which remained helical

(Fig.S4). **Alaαh** bends in many different positions (Fig.S5), while **EK2R1αh** exhibits small bending angles during the simulations (Fig.S6).

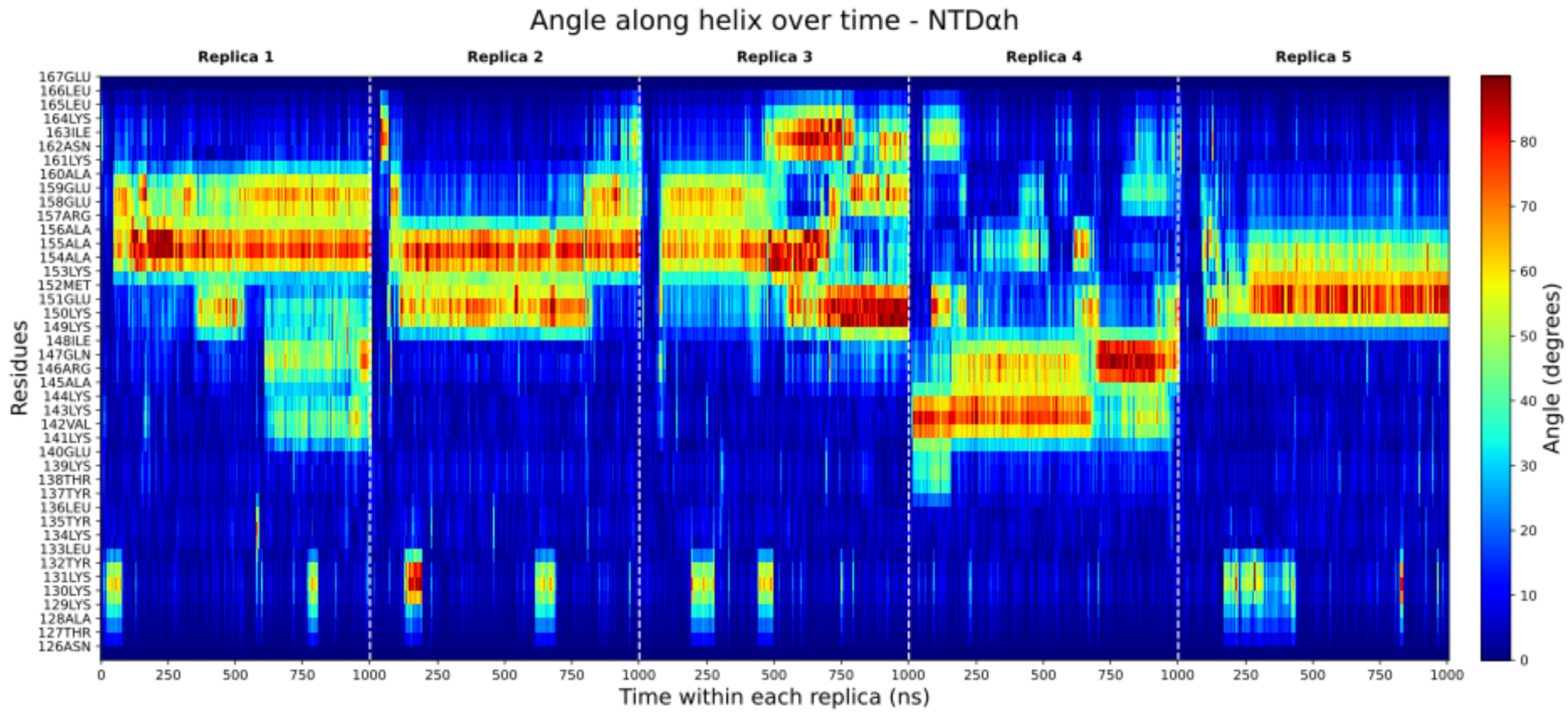

Figure S4. Bending angles for each residue of **NTDαh** in the Amber14SB+TIP3P-based MD simulations of 5 replicas, plotted as a function of simulated time.

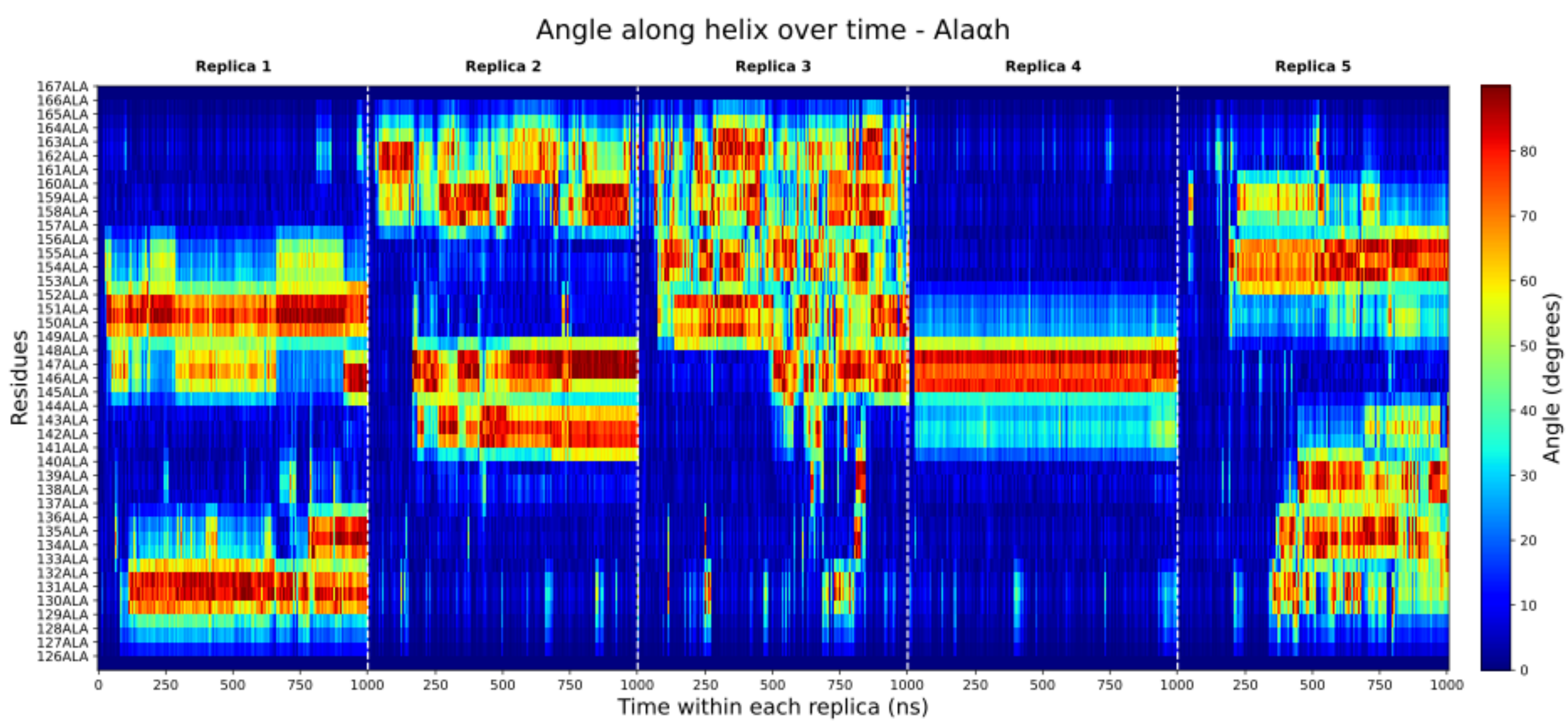

Figure S5. Same as Fig. S4 but for **Alaαh.**

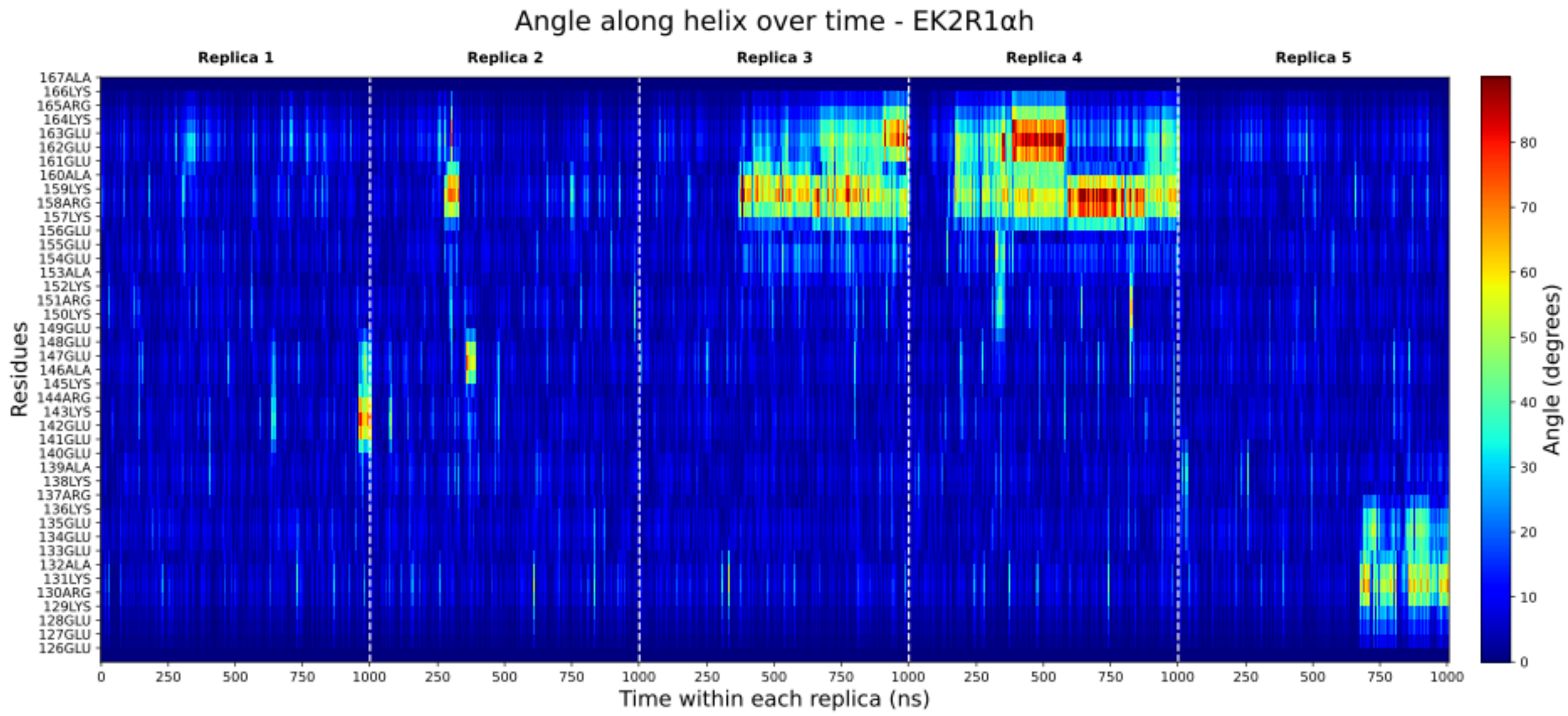

Figure S6. Same as Fig. S4 but for **EK2R1αh.**

1.3. PAD

A PAD value is a measure of the local fluctuation of the backbone torsion angles: the larger the value, the larger the fluctuations.
The timeline of PAD was calculated using 100-ns sliding windows with a 10-ns step, yielding one PAD value every 10 ns. Each PAD value was assigned to the center of its corresponding window.

In the Amber14SB+TIP3P-based MD, the PAD values of most **NTDαh'**s residues increase with simulated time. This is not the case for residues 132 to 139, which remain helical during the simulations (Fig.S7). The PAD values increase for the residues of **Alaαh** (Fig.S8) while the values for most residues of **EK2R1αh** do not change (Fig. S9).

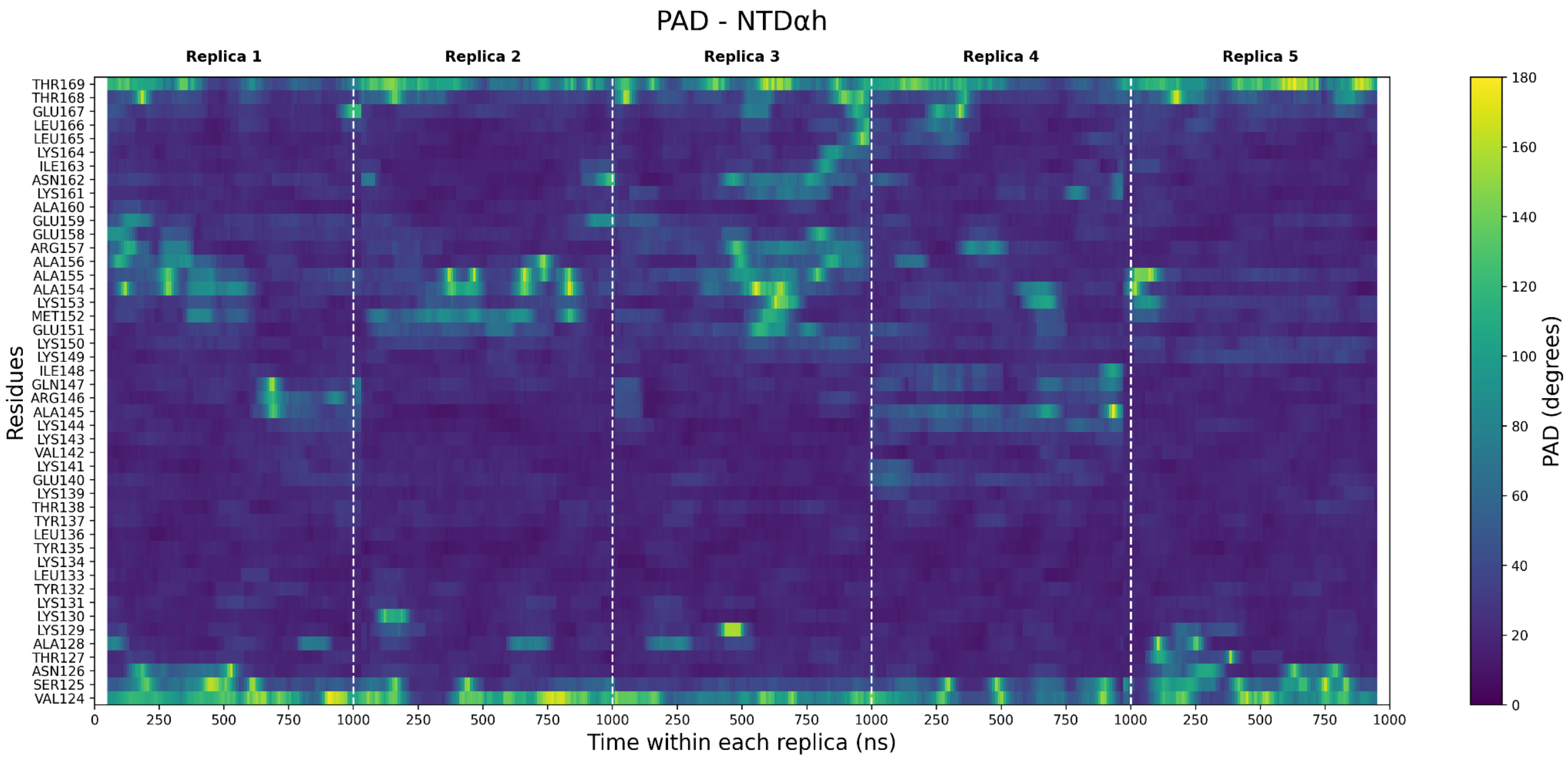

Figure S7. PAD value of each residue in the Amber14SB+TIP3P-based MD of **NTDαh,** plotted as a function of simulated time (5 replicas).

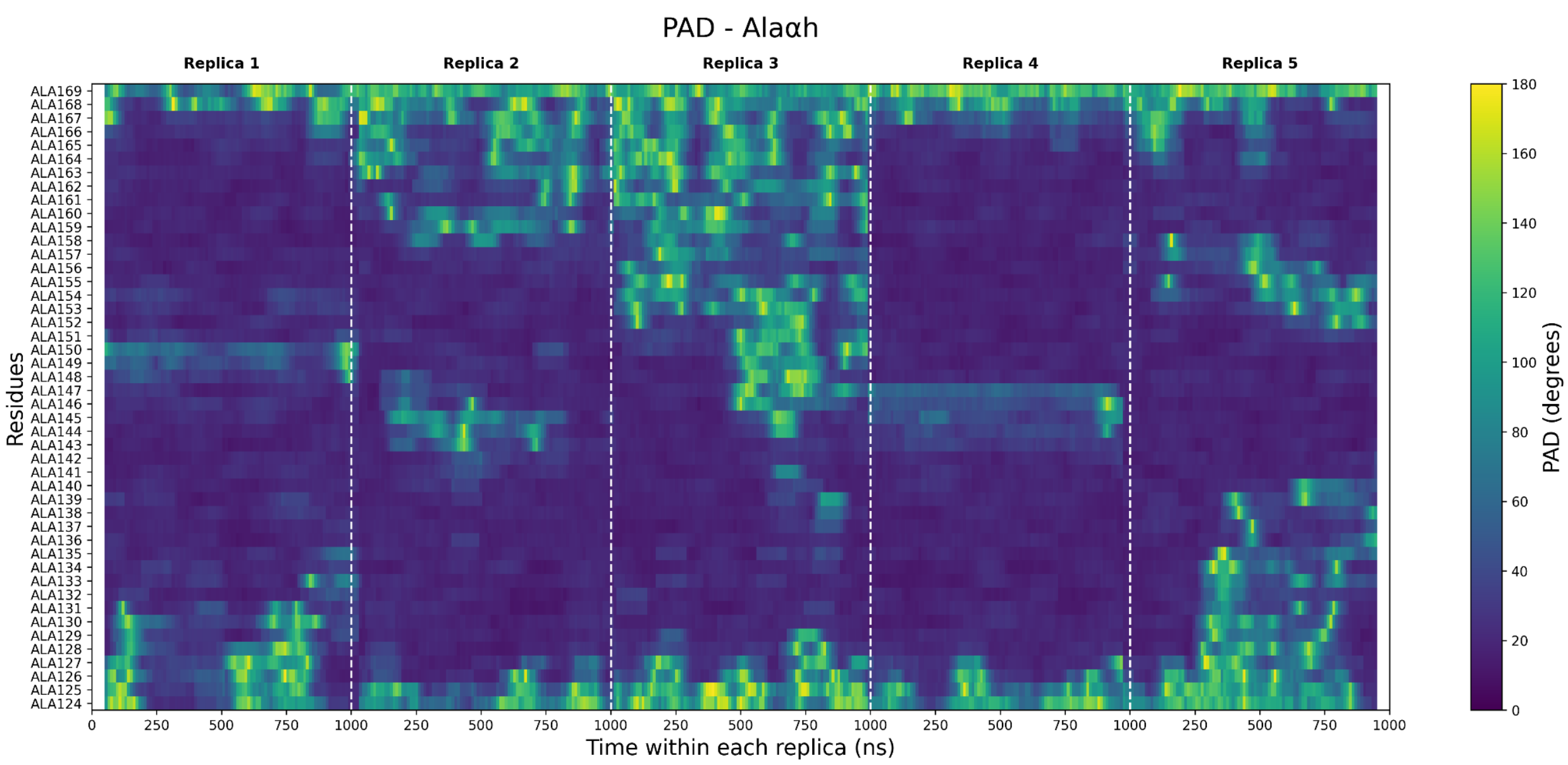

Figure S8. Same as Fig. S7 but for **Alaαh**.

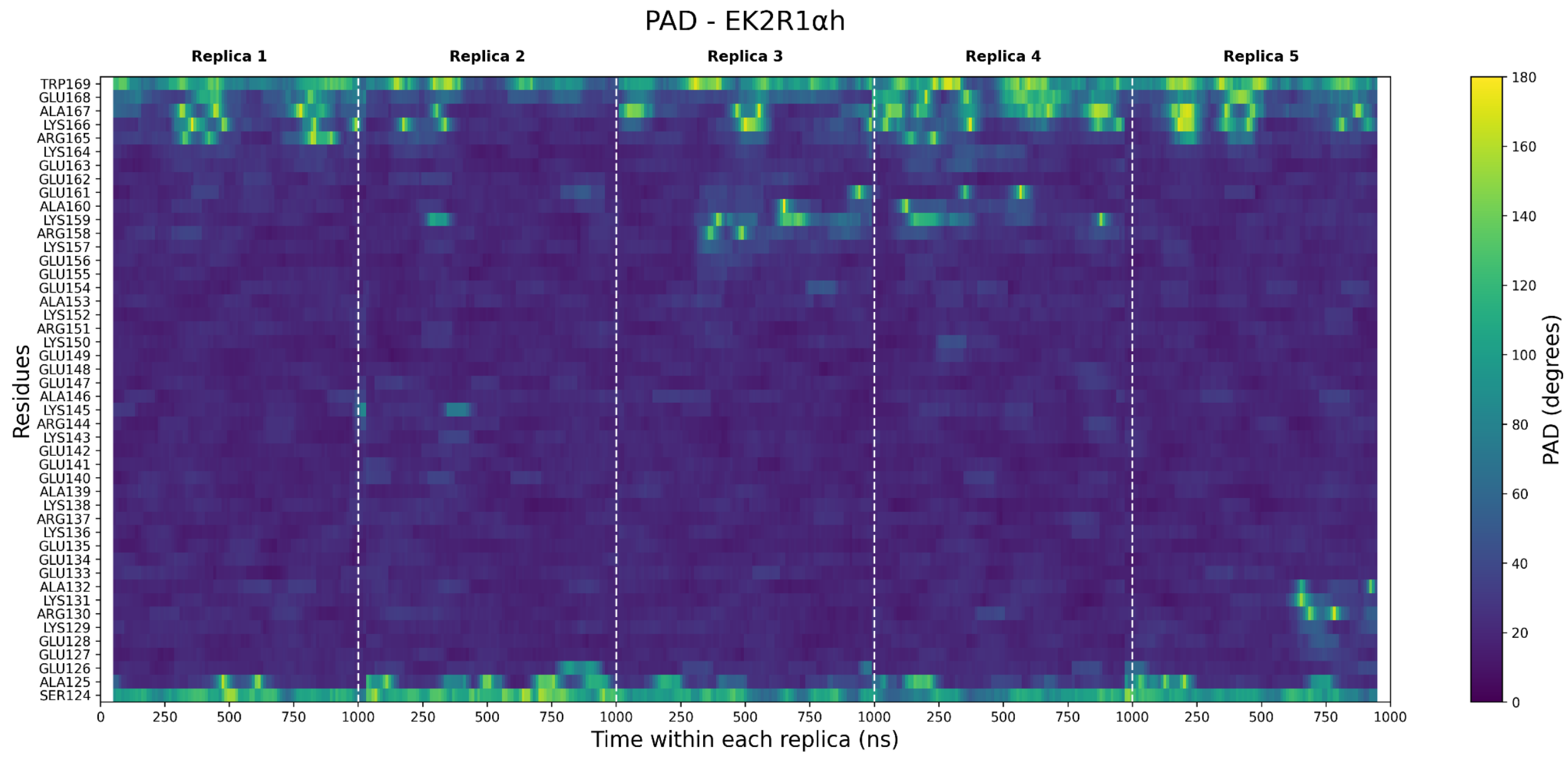

Figure S9. Same as Fig. S7 but for **EK2R1αh**.

1.4. SASA

In the Amber14SB+TIP3P-based MD of **NTDαh**, the SASA values of most residues decrease with simulated time. However, this is not the case for residues 132 to 139 (Fig.S10). The **Alaαh** residues' SASA decreases (Fig.S11), while that of **EK2R1αh** does not vary significantly (Fig.S12).

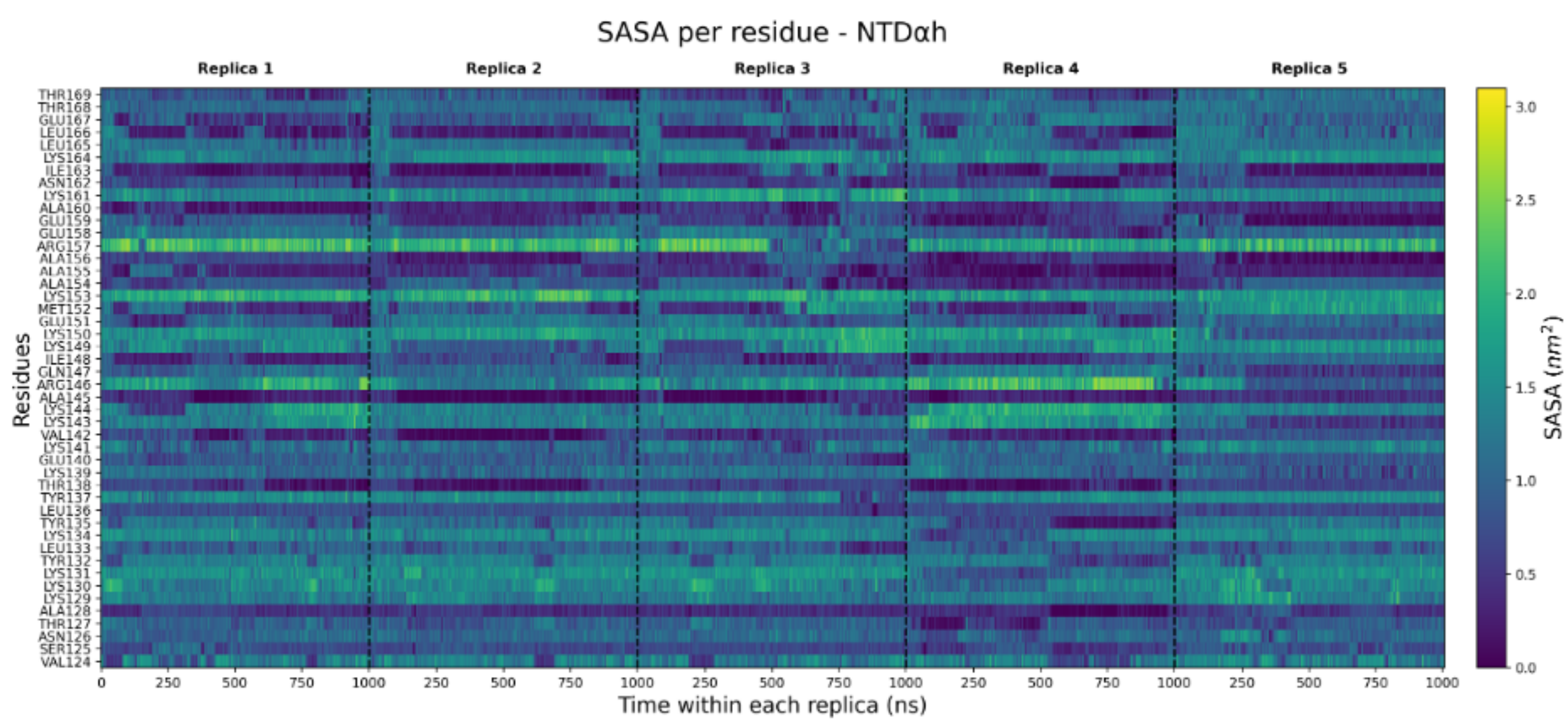

Figure S10. SASA of each residue in the Amber14SB+TIP3P-based MD of **NTDαh** (5 replicas) plotted as a function of simulated time.

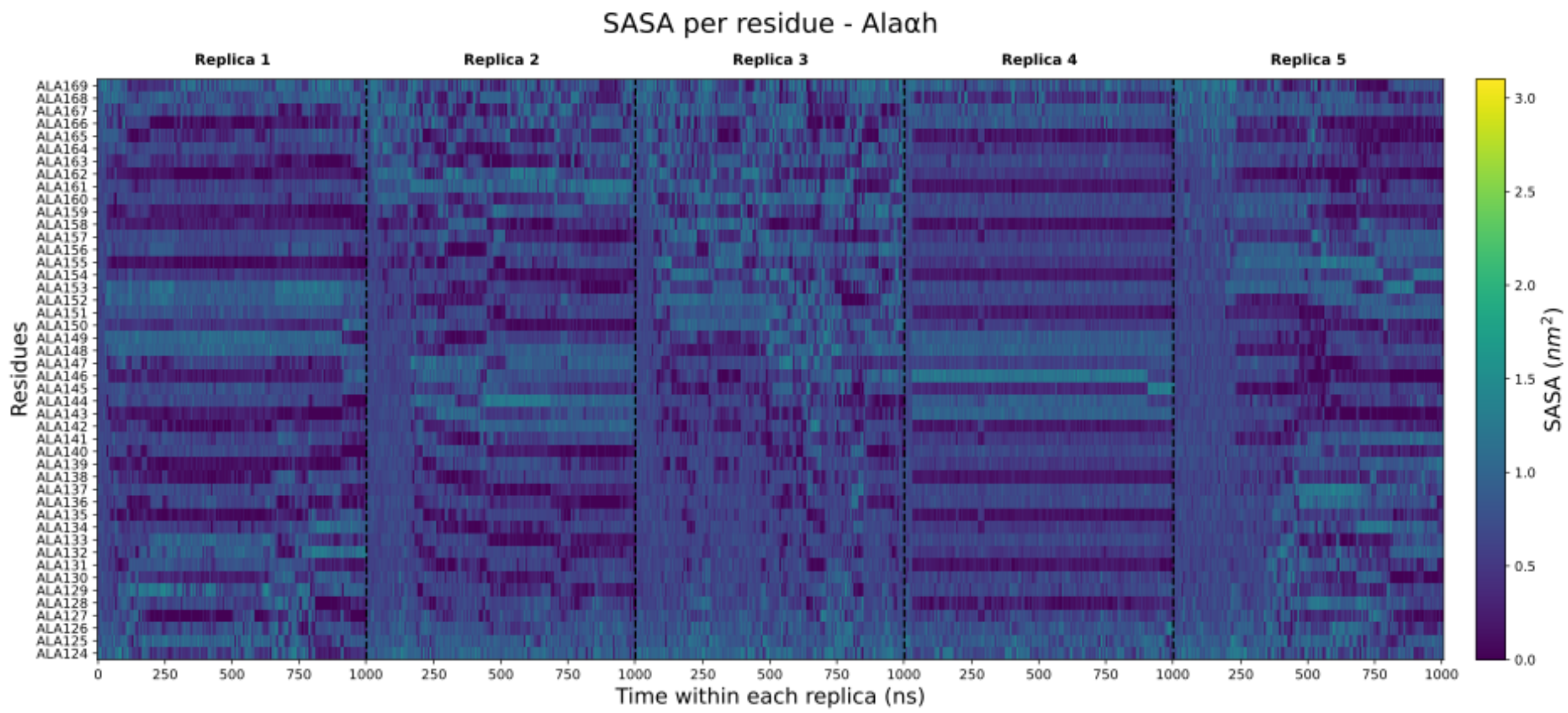

Figure S11. Same as Fig. S10 but for **Alaαh**.

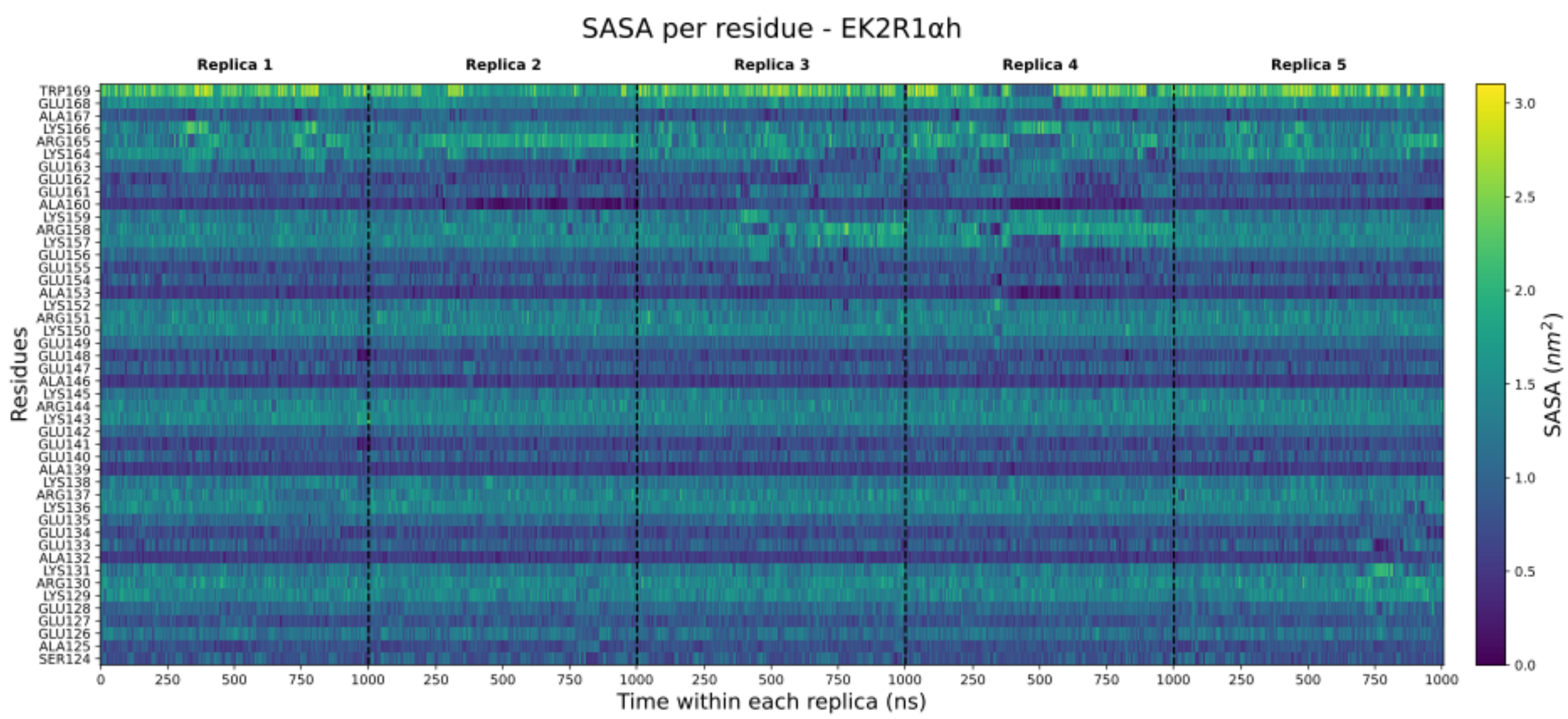

Figure S12. Same as Fig. S10 but for **EK2R1αh**.

2. Clustering analysis (Amber14SB+TIP3P-based MD)

The clustering analysis was performed on the MD trajectories of all helices using the GROMACS implementation of the GROMOS algorithm[3] with an RMSD cutoff of 0.4 nm.

Cluster	Population
1	73.6%
2	11.2%
3	6.0%
Other (15 clusters)	9.2%

Table S1. Cluster populations from the Amber14SB+TIP3P-based MD of **EK2R1αh**.

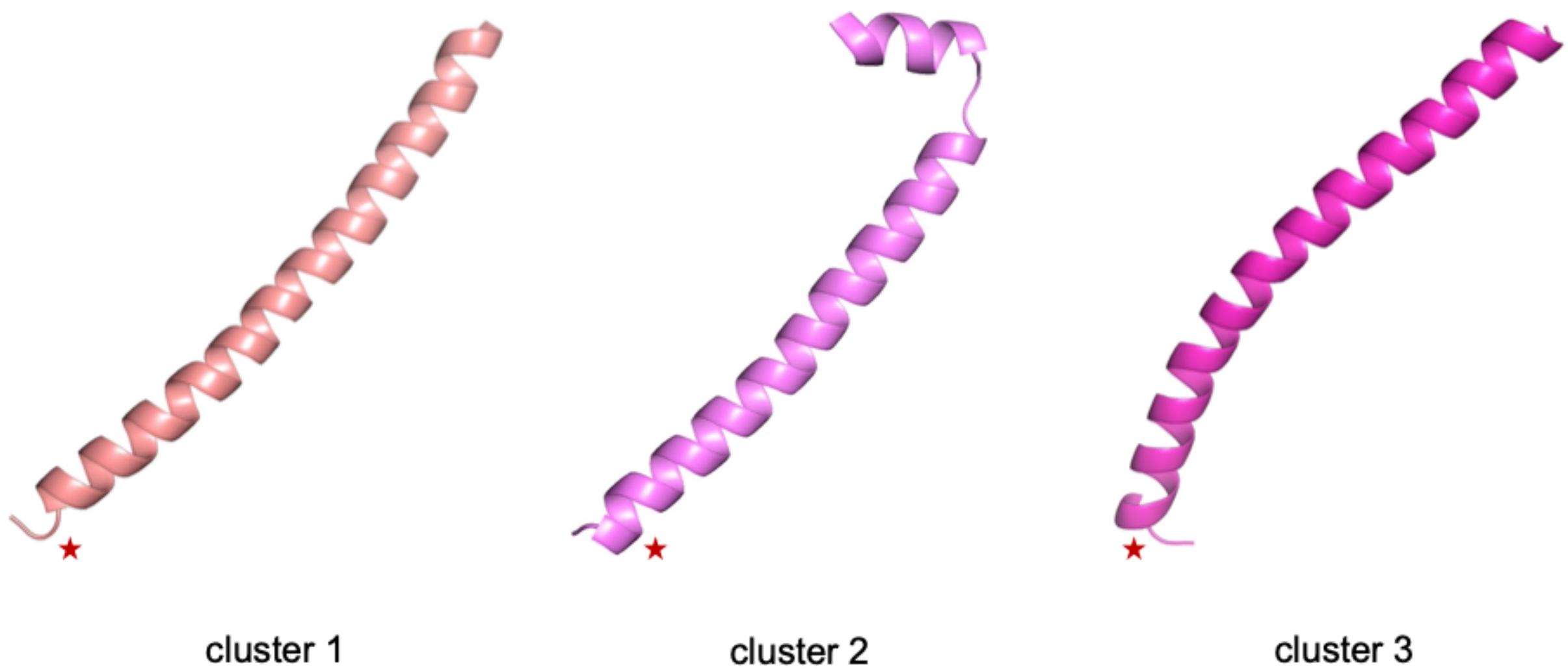

Figure S13. Representative structure of each cluster from the Amber14SB+TIP3P-based MD of **EK2R1αh**. The red star highlights the N-terminus.

Cluster	Population
1	20.3%
2	10.6%
3	4.9%
4	4.6%
5	4.4%
6	4.3%
7	4.1%
8	2.7%
9	2.4%
10	2.3%
Other (141 clusters)	39.4%

Table S2. Same as Tab. S1 but for **Alaαh.**

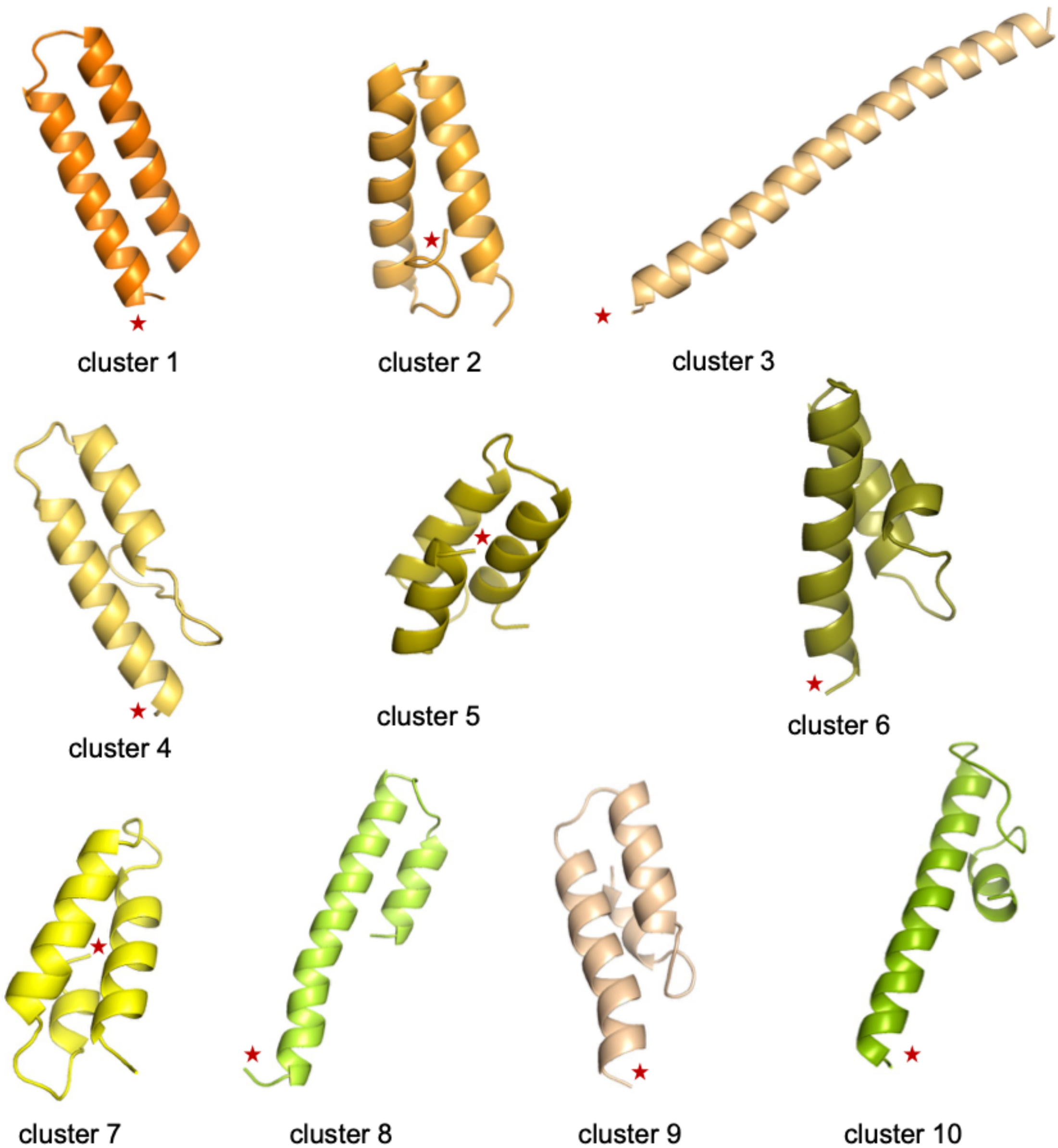

Figure S14. Same as Fig. S13 but for **Alaαh**.

Cluster	Population
1	34.6%
2	11.0%
3	11.0%

4	8.3%
5	7.3%
6	6.7%
7	4.7%
8	3.9%
Other (31 clusters)	12.5%

Table S3. Same as Tab. S1 but for **NTDαh**.

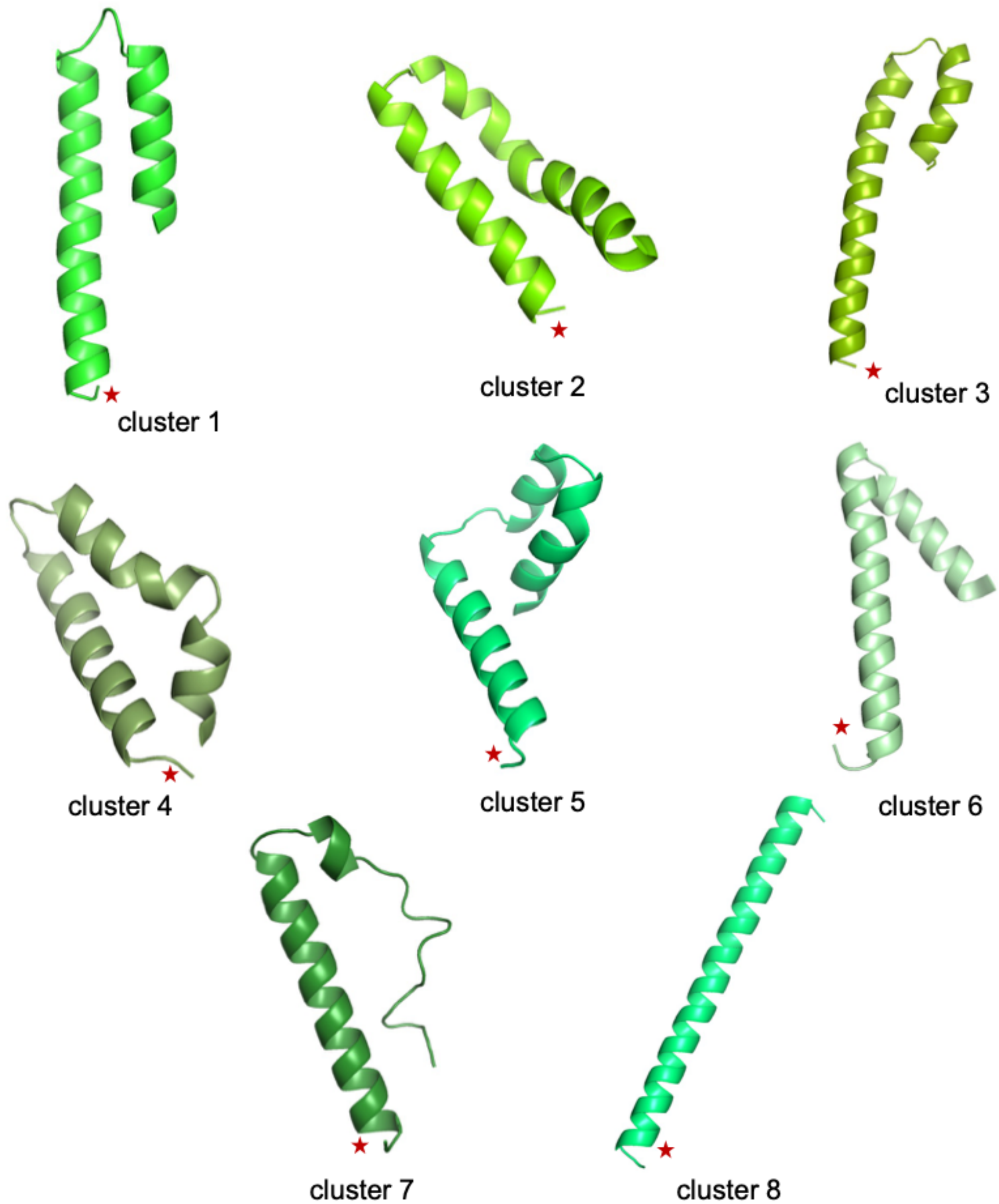

Figure S15. Same as Fig. S13 but for **NTDαh**.

3. PCA - based free energy analysis (Amber14SB+TIP3P-based MD)

Principal component analysis (PCA) was performed on the MD trajectories of all helices using GROMACS[4]. The trajectories were projected onto the first two principal components (PC1 and PC2), which capture the dominant collective motions of the system. A two-dimensional probability distribution was then calculated from the PC1–PC2 projections and converted into the corresponding free-energy landscape according to $\Delta G = -kT \ln P + C$.

From the resulting free-energy landscapes, highly populated conformational basins corresponding to local free-energy minima were identified. Frames within each selected basin were extracted from the corresponding trajectory and clustered using the GROMOS algorithm[3], as implemented in GROMACS, with a 0.4 nm clustering cutoff. For each basin, the representative conformation, defined as the centroid of the corresponding cluster, was reported.

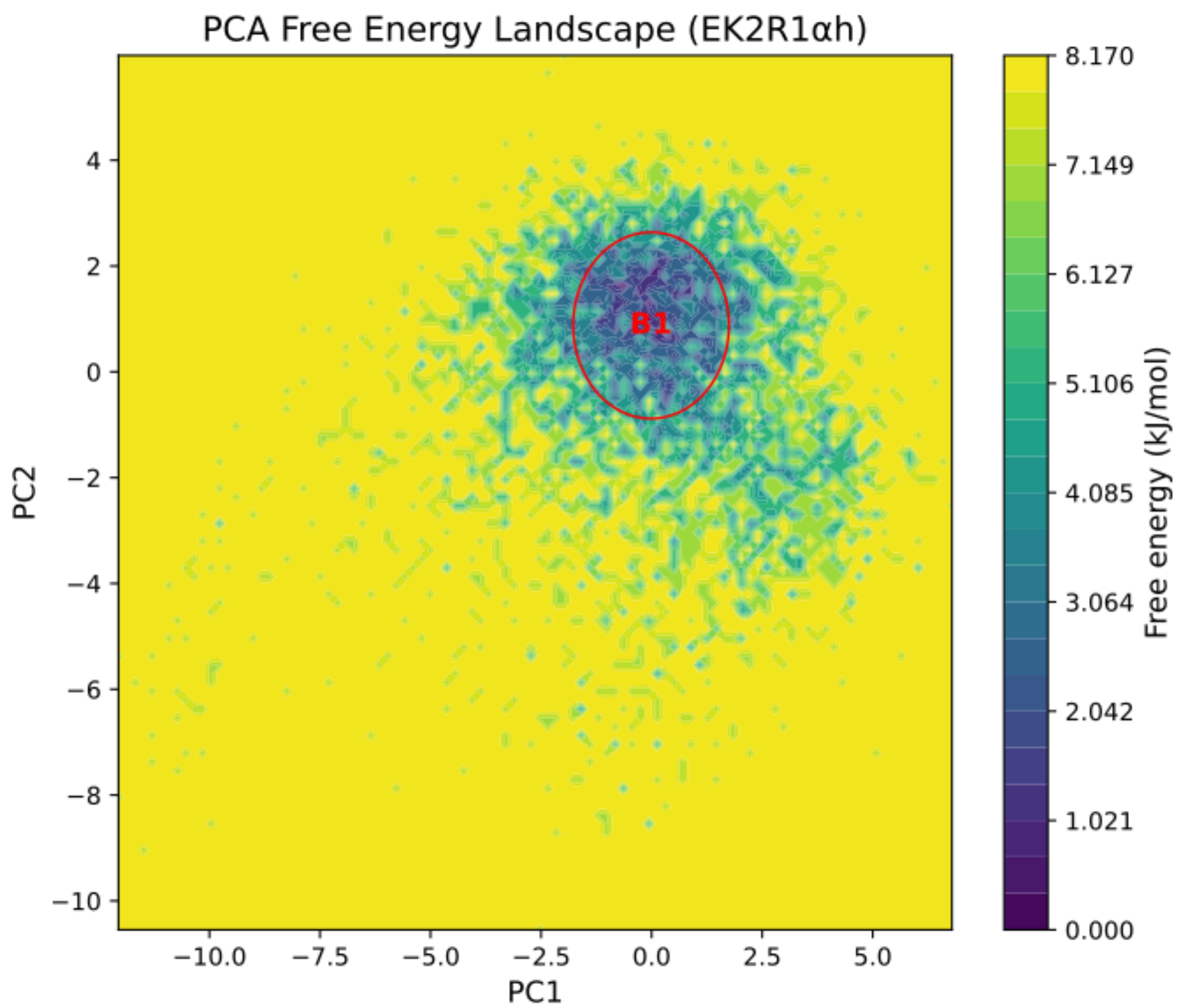

Figure S16. PCA relative free energy landscape from the Amber14SB+TIP3P-based MD of **EK2R1αh**. The minimum is set to zero. The free energy is plotted as a function of the first two principal components (PC1 and PC2). The basin B1 is marked with the red circles.

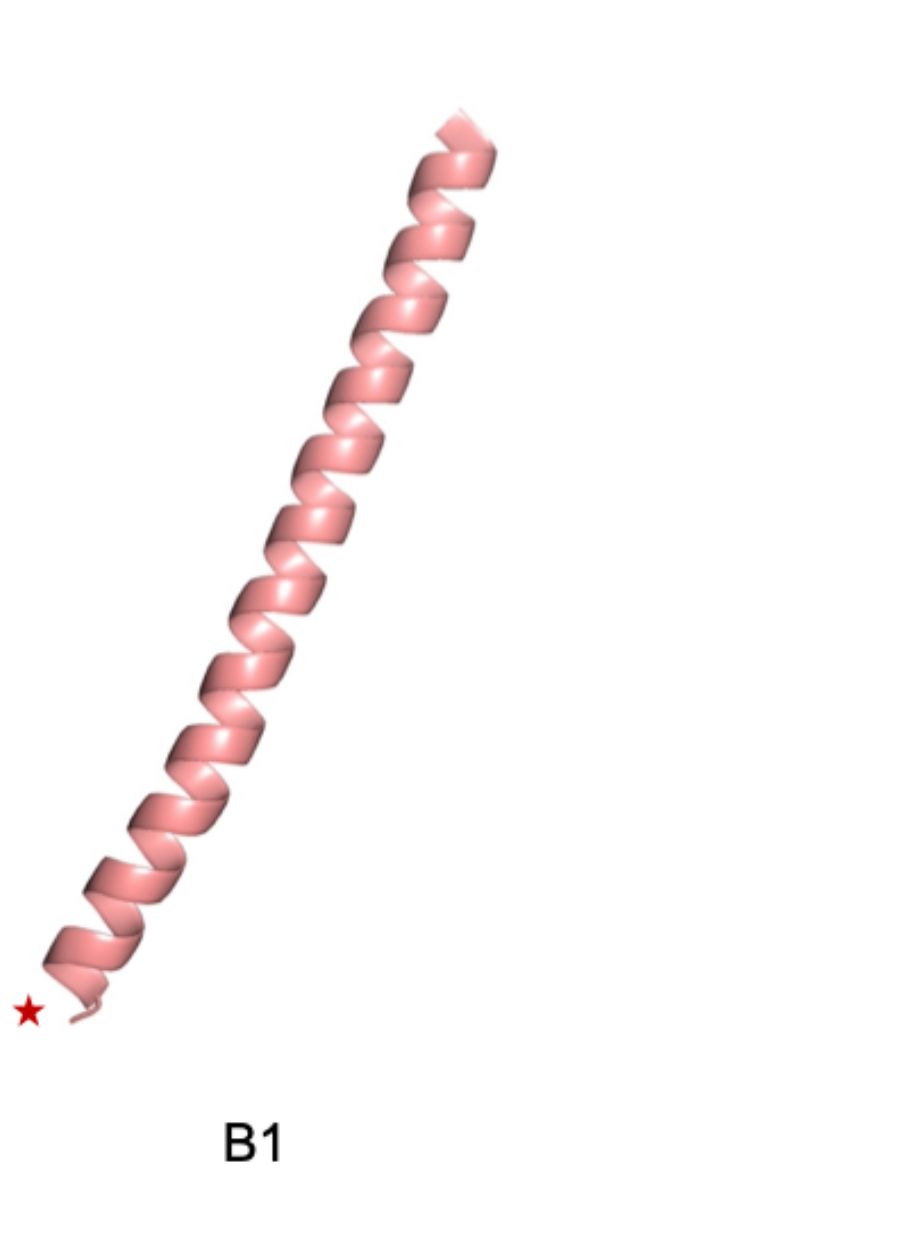

Figure S17. Representative structures from a clustering analysis of the basin 1 in Fig. S16. The red star highlights the N-terminus.

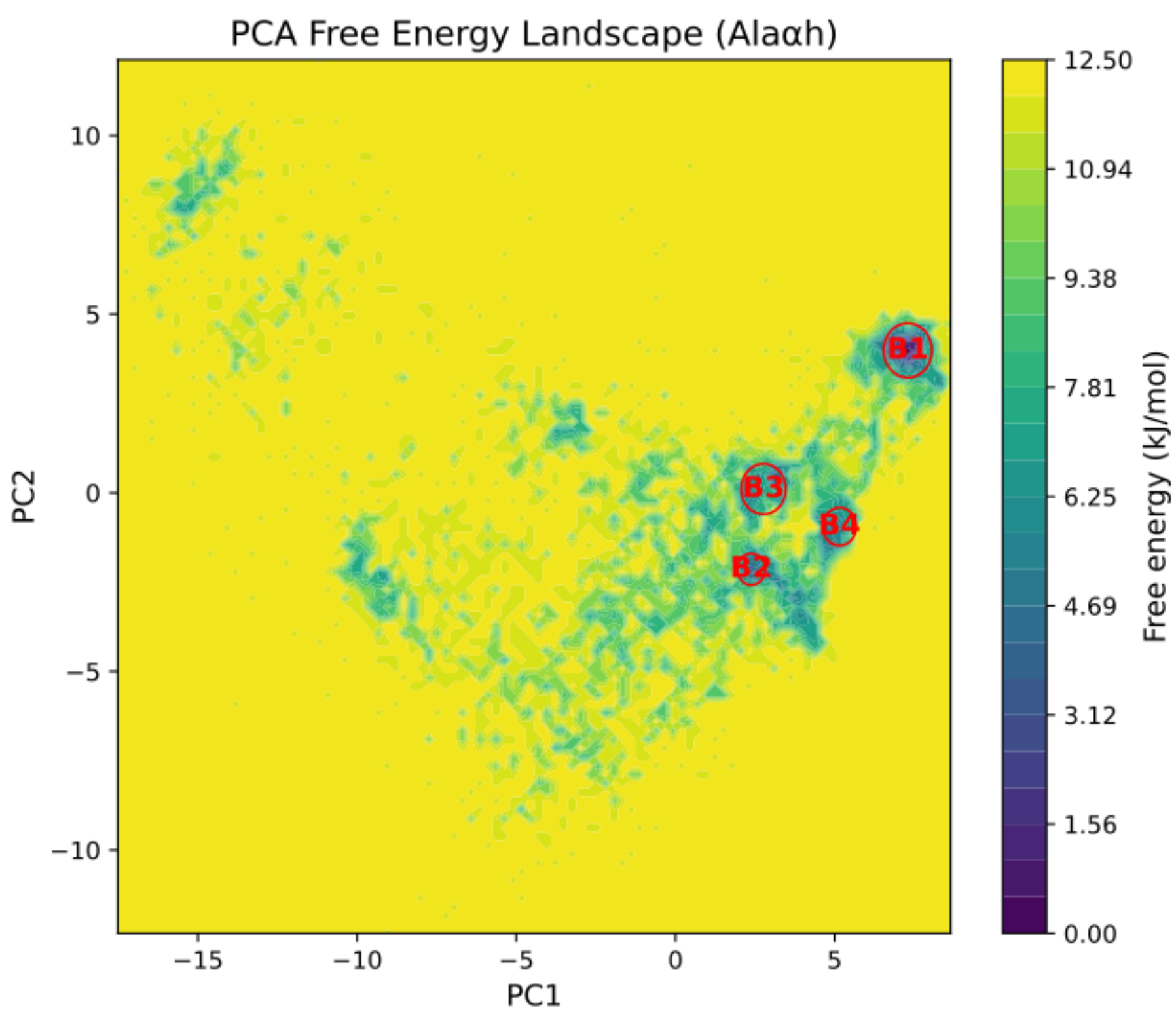

Figure S18. Same as Fig. S16 but for **Alaαh**.

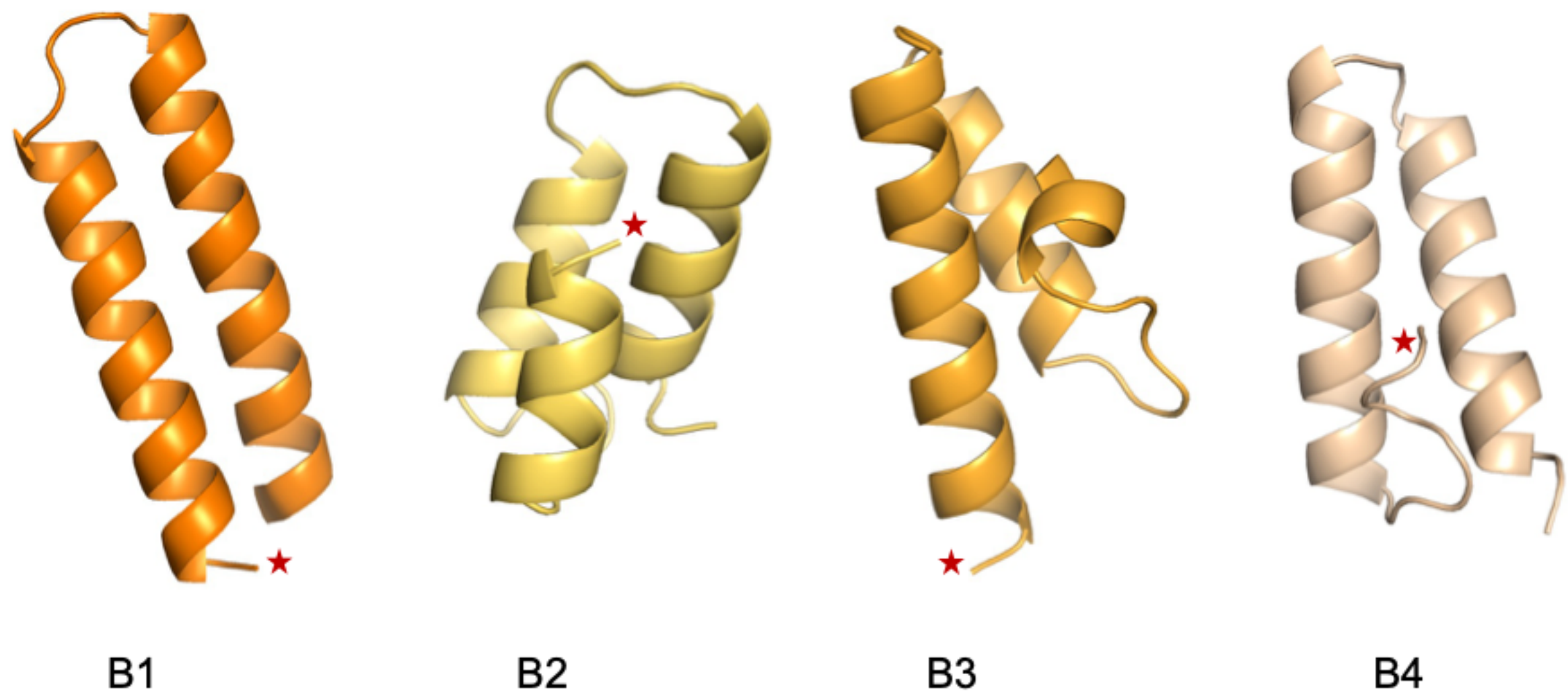

Figure S19. Representative structures from a clustering analysis of the basins in Fig. S18.

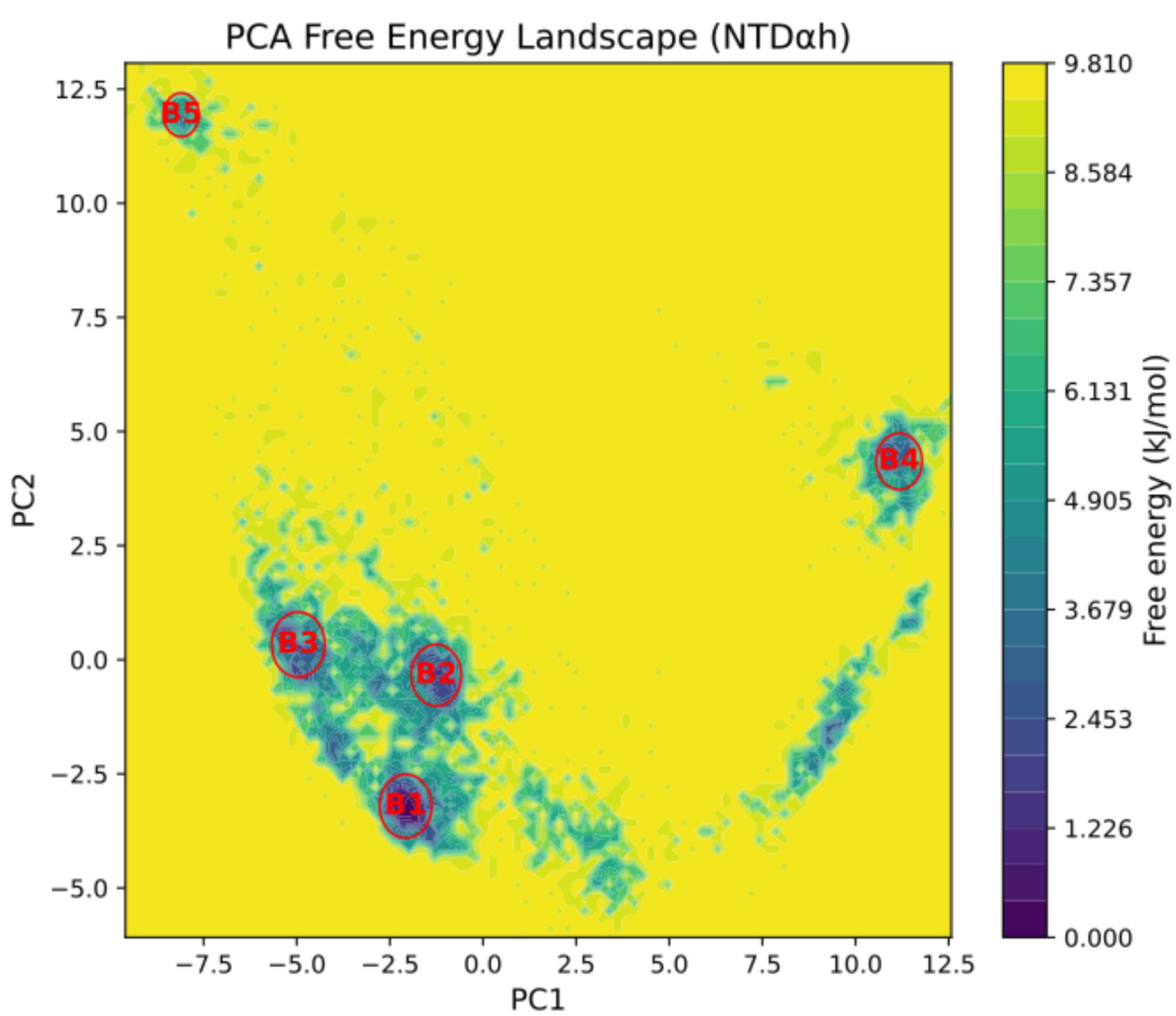

Figure S20. Same as Fig. S16 but for **NTDαh**.

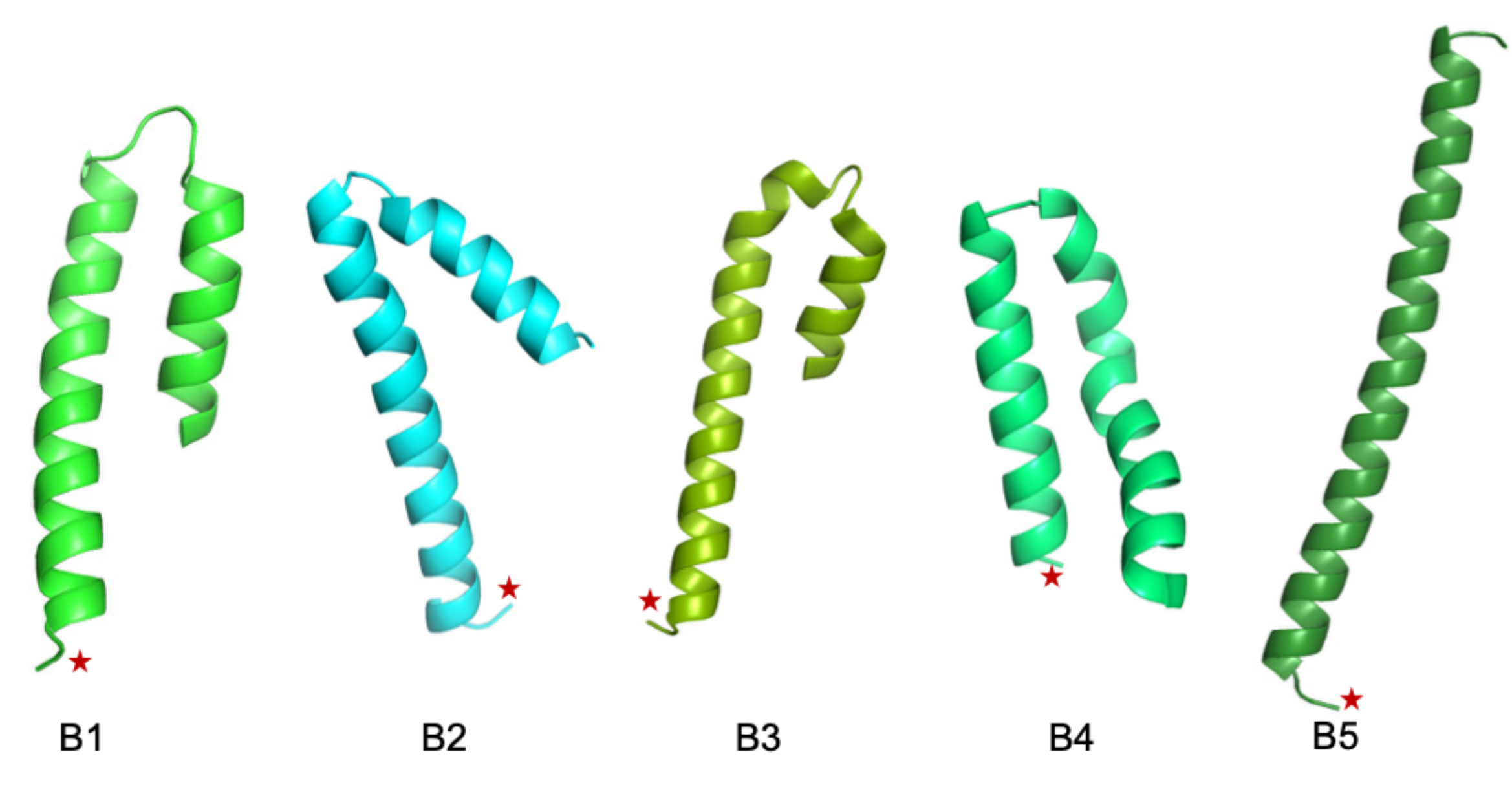

Figure S21. Representative structures from a clustering analysis of the basins in Fig. S20.

4. AMBER14SB+OPC- based MD simulations of NTDαh, Alaαh, EK2R1αh.

4.1 Secondary structure analysis

In our Amber14SB[5]+OPC[6]-based MD simulations (Fig.S22-S25, Tab.S4), we observe the following: (i) **NTDαh** displays a region with high α-helical content (residues 134 to 146, >95% content) (Fig.S22a,d), as seen in the Amber14SB[5]+TIP3P[7]-based MD (Fig.2, main text), although this region is smaller in the latter simulations (residues 132 to 139). (ii) Almost half of the residues of **Alaαh** exhibit α-helical content <50%. Only 5 residues feature 95% or more α-helical content (Fig.S22b,e). (iii) Almost the entire sequence of **EK2R1αh** shows a high α-helical content (80% or more, Fig.S22c,f).

The overall ranking of α-helical composition is the same as that of the Amber14SB+TIP3P-based MD simulations (Tab.1, main text) for the Amber14SB+OPC-based MD (**EK2R1αh** has the highest α-helical content (~80%), followed by **NTDαh** (~70%) and then **Alaαh** (~60%) (Tab.S4).

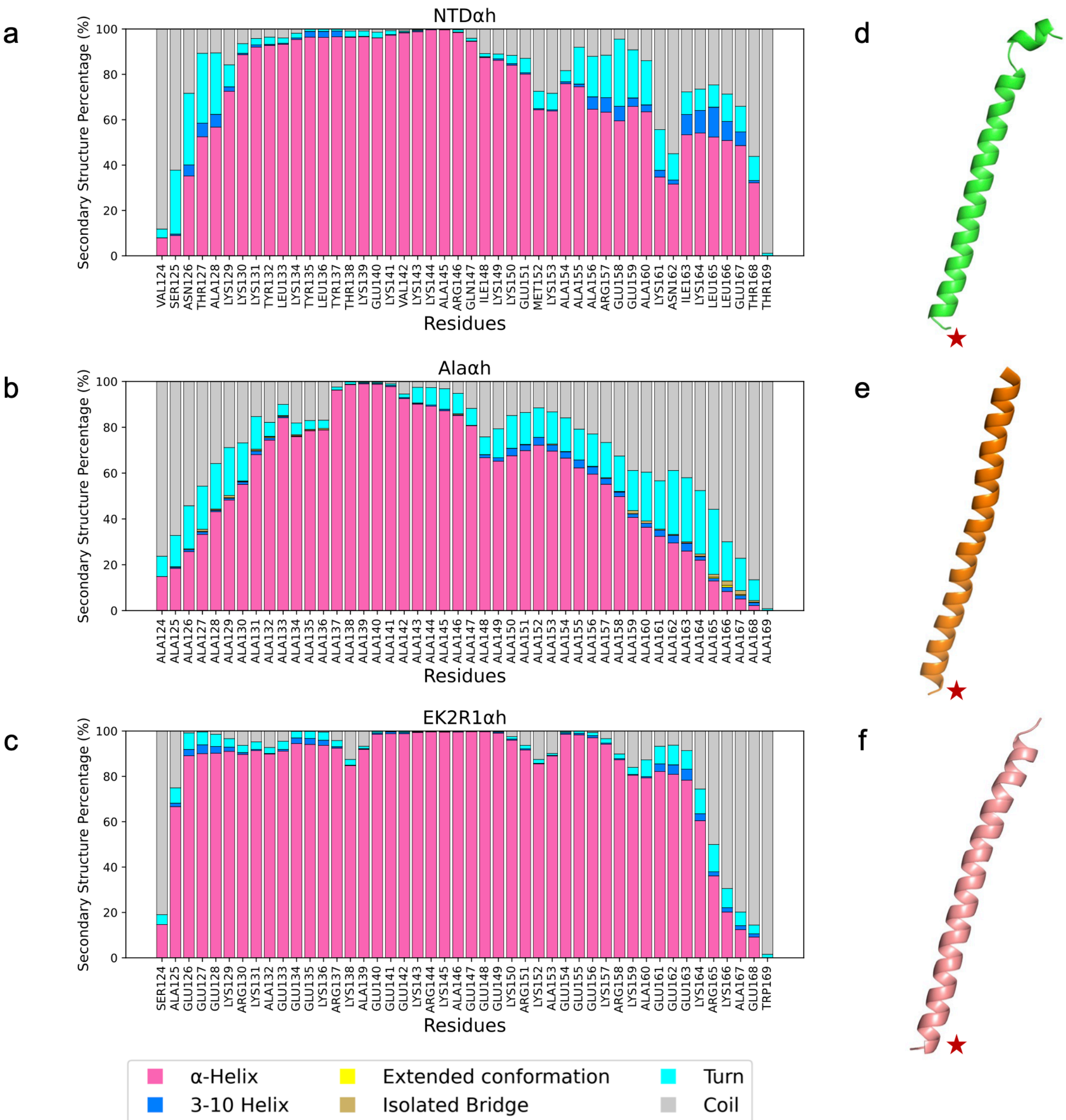

Figure S22. Amber14SB+OPC-based MD of **NTDαh**, **Alaαh** and **EK2R1αh**, **a-c** Secondary structure composition per residue. **d-f** Corresponding representative structures, defined as the central structure of the largest cluster obtained from cluster analysis in GROMACS. The red star highlights the N-terminus.

	NTDαh	Alaαh	EK2R1αh
α-Helix	70.70 (12.07) %	57.26 (20.12) %	80.97 (11.80) %
3-10 Helix	2.50 (4.07) %	1.32 (3.15) %	1.21 (3.06) %
Extended conformation	0.00 (0.00) %	0.10 (1.12) %	0.00 (0.00) %
Isolated Bridge	0.00 (0.14) %	0.39 (1.33) %	0.00 (0.00) %
Turn	8.95 (8.08) %	12.22 (11.12) %	3.36 (6.10) %
Coil	17.85 (9.35) %	28.70 (13.84) %	14.46 (7.34) %

Table S4. Secondary structure composition over the cumulative trajectory of Amber14SB+OPC-based MD of **NTDαh, Alaαh, EK2R1αh.** The average and standard deviation (in parentheses) of each composition across the trajectory are shown.

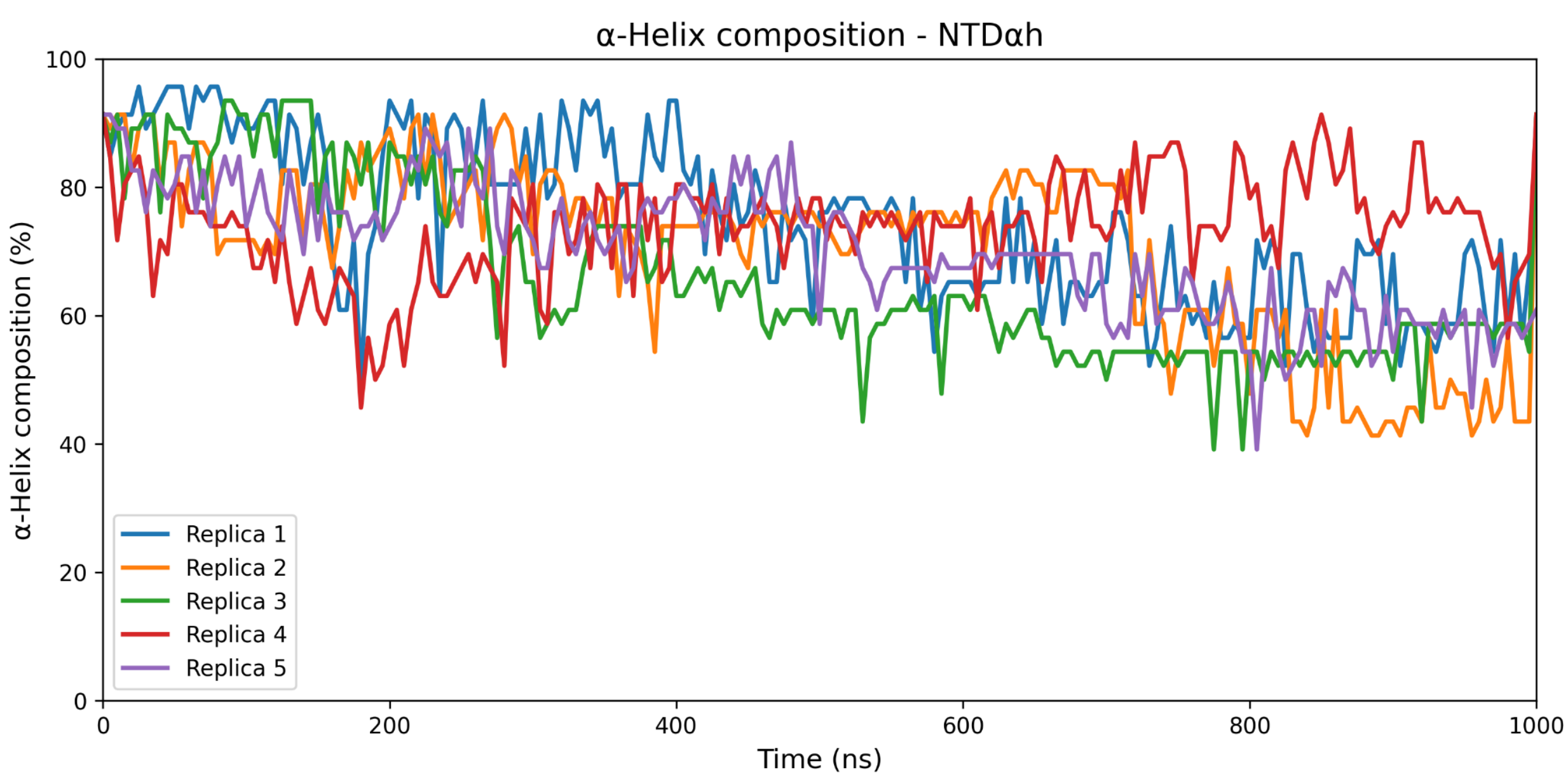

Figure S23. α-helix content of **NTDαh** in the Amber14SB+ OPC -based MD (5 replicas), plotted as a function of simulated time.

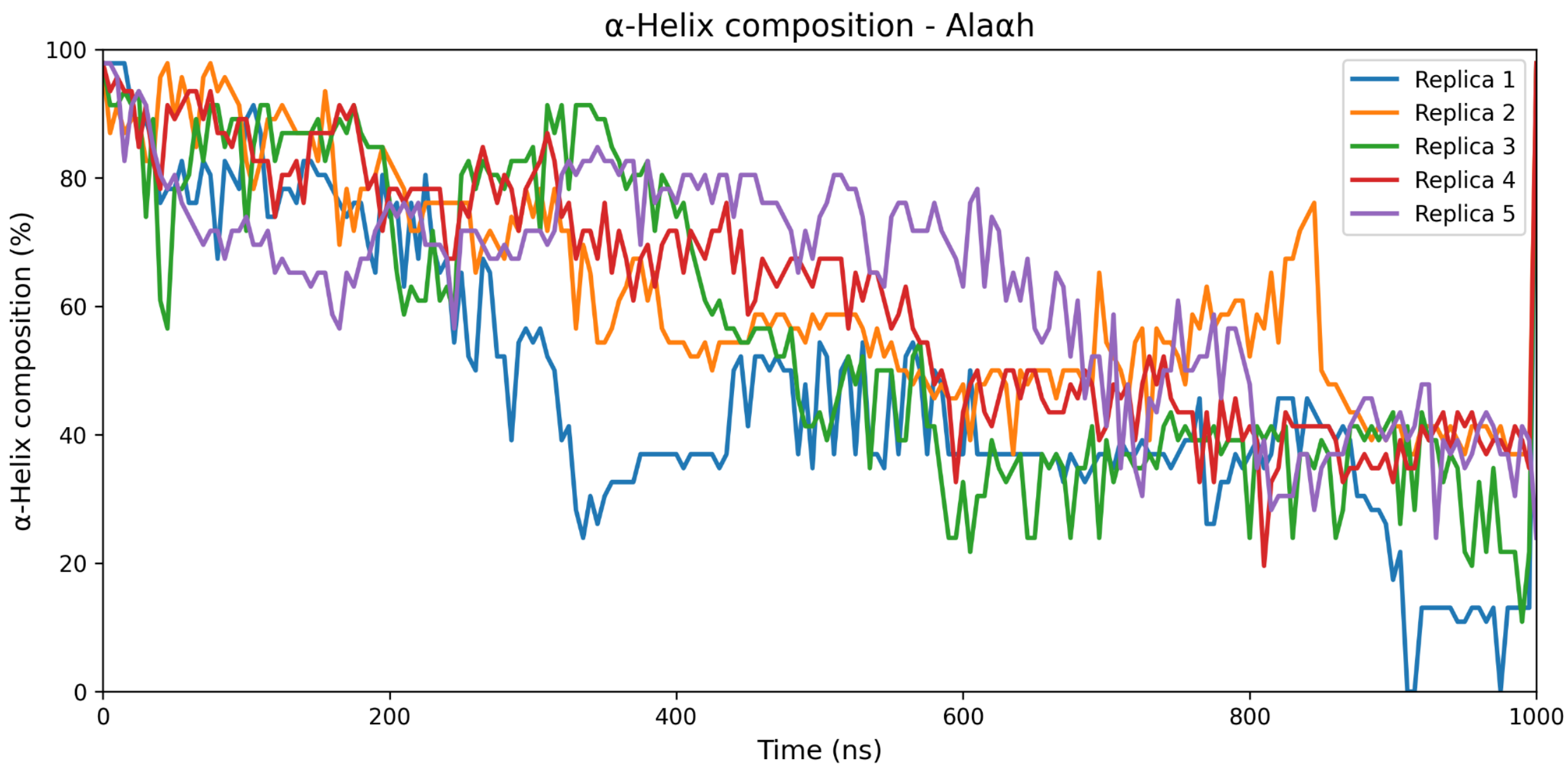

Figure S24. Same as Fig. S23 but for the Amber14SB+OPC-based MD of **Alaαh**.

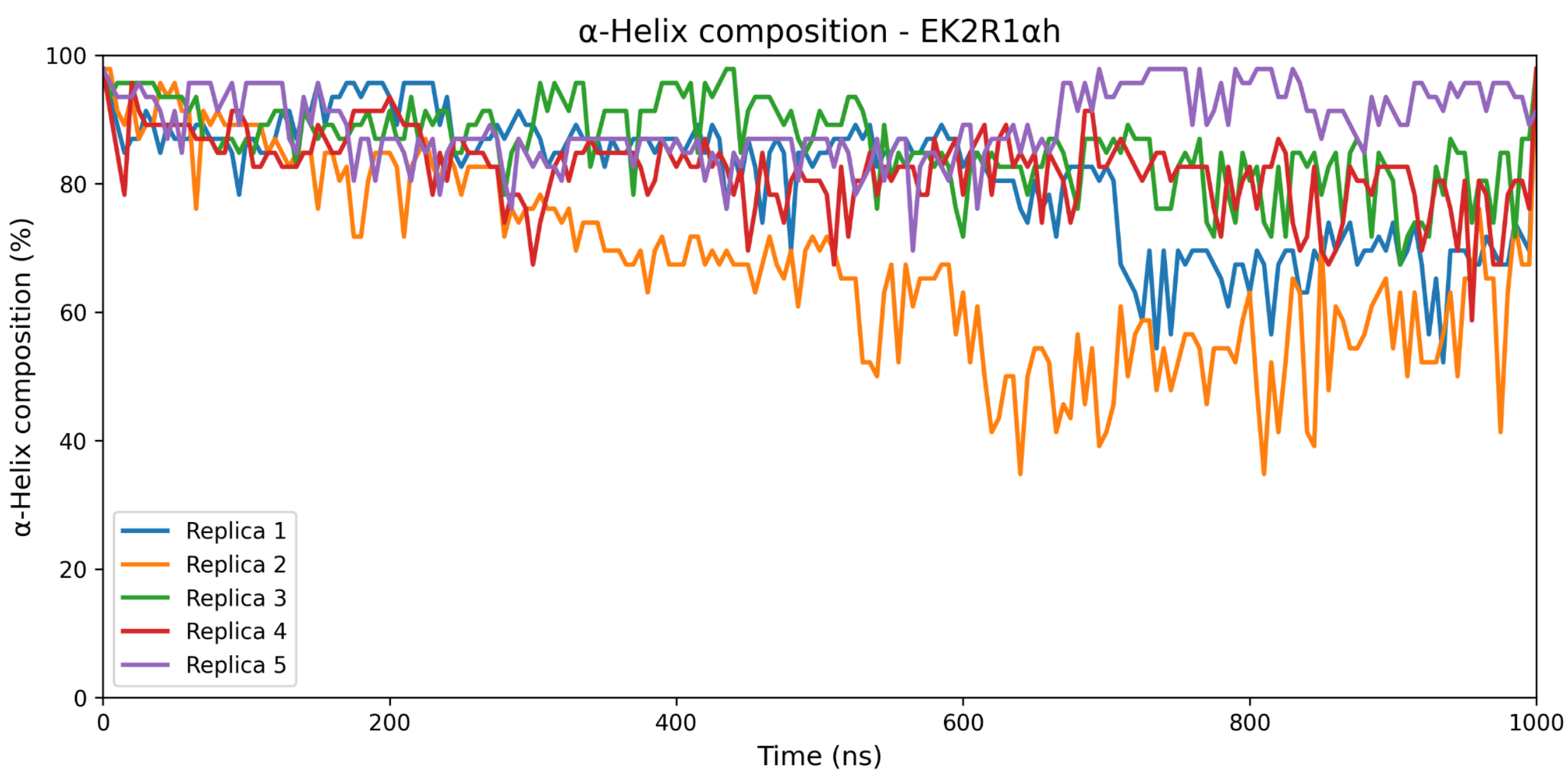

Figure S25. Same as Fig. S23 but for the Amber14SB+OPC-based MD of **EK2R1αh**.

4.2. Bending angles

In general, smaller fluctuations of the bending angles are associated with high helical content.

The bending angle distributions in the Amber14SB+OPC MD simulations (Figs.S26-S29) of the three systems are not too dissimilar from those of Amber14SB+TIP3P. In particular, **NTDαh** features a heterogeneous distribution: (i) relatively small average bending angles (~5-15°) with small fluctuations in the region from the first few residues of the N-terminus to the middle of the sequence, (ii) relatively large bending angles (~30-60°) with large fluctuations from the middle of the sequence to the C-terminus (Fig.S26a,d). **Alaαh** exhibits large fluctuations for most of the residues (Fig.S26b,e). **EK2R1αh** has small average bending angles with small fluctuations (Fig.S26c,f).

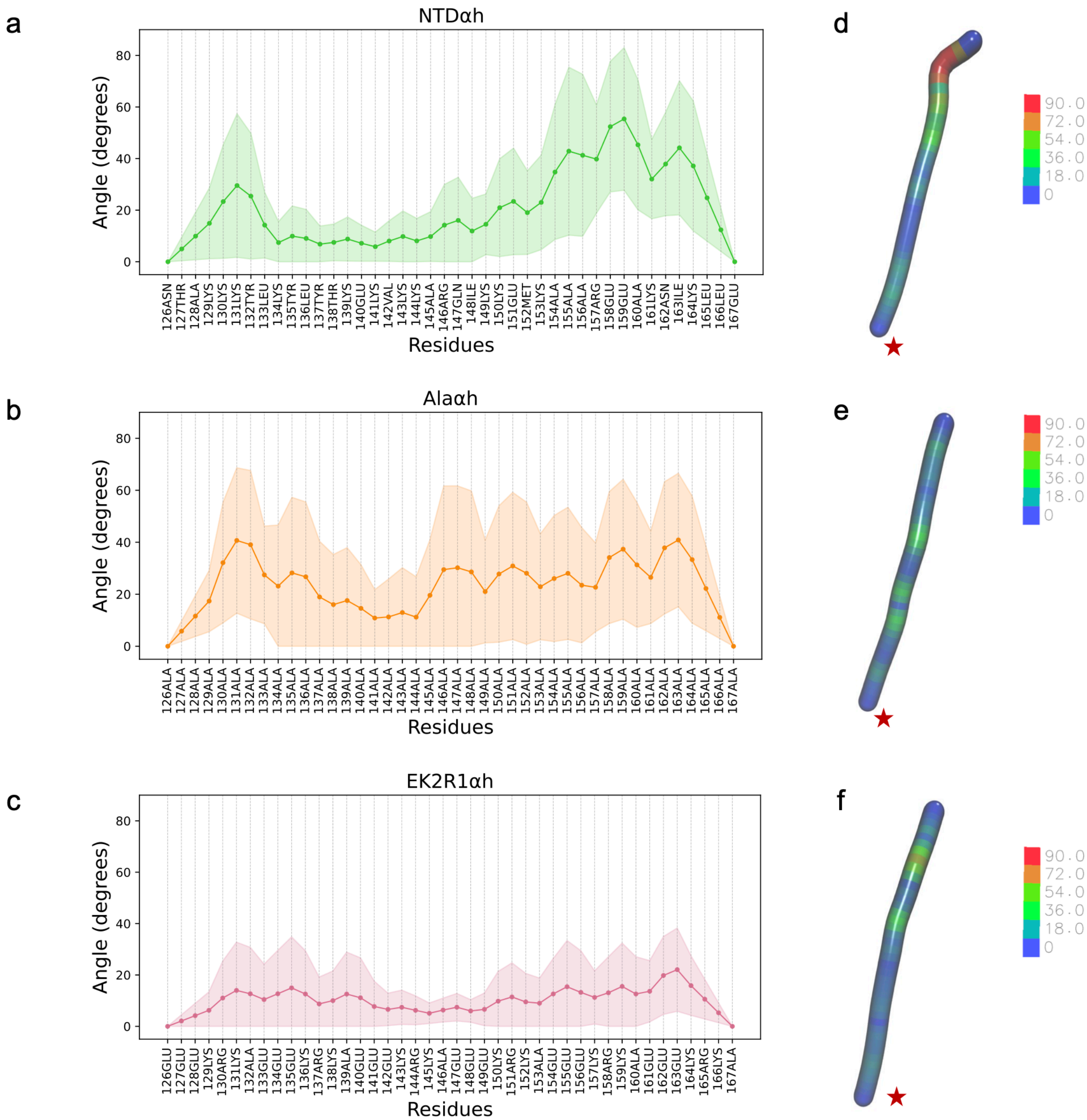

Figure S26. Amber14SB+OPC-based MD of **NTDαh, Alaαh** and **EK2R1αh**. **a-c** Average bending angles, along with their standard deviations, along the cumulative trajectories. **d-f** Representative structures of the helices. The red color indicates the areas of highest helix axis angle, green indicates intermediate angle magnitudes and blue indicates angles close to or at 0 degrees. The red star highlights the N-terminus.

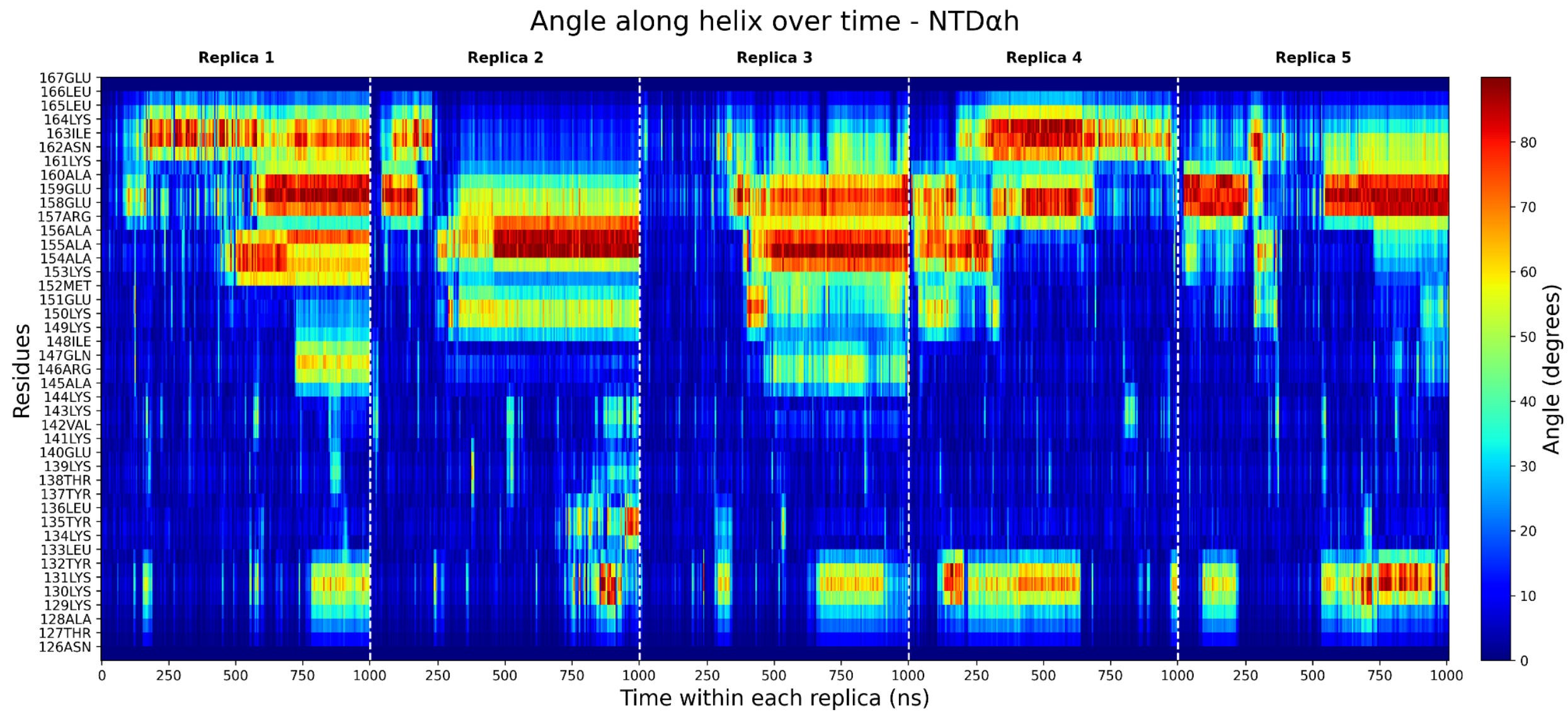

Figure S27. Bending angles for each residue of **NTDαh** in the Amber14SB+OPC-based MD simulations of 5 replicas, plotted as a function of simulated time.

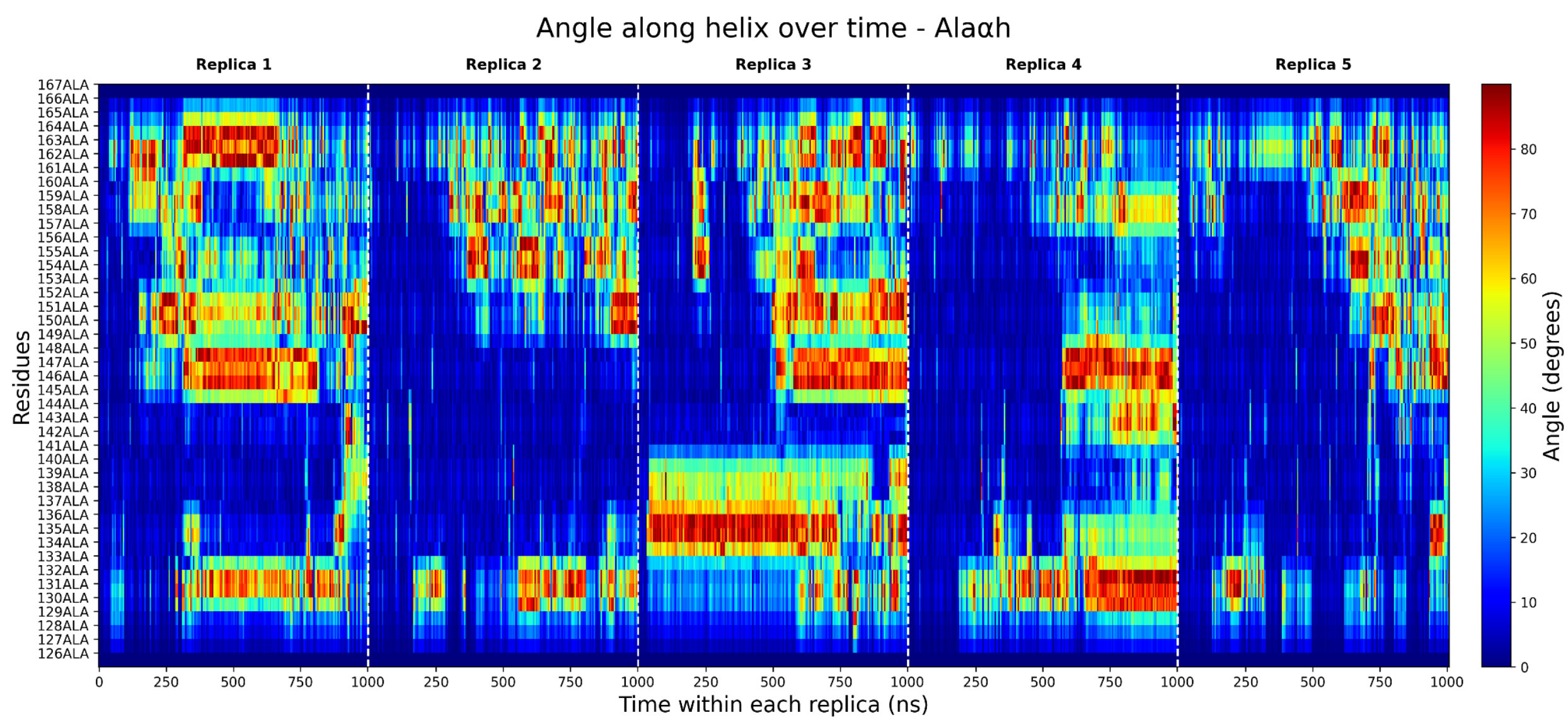

Figure S28. Same as Fig. S27 but for Amber14SB+OPC-based MD of **Alaαh**.

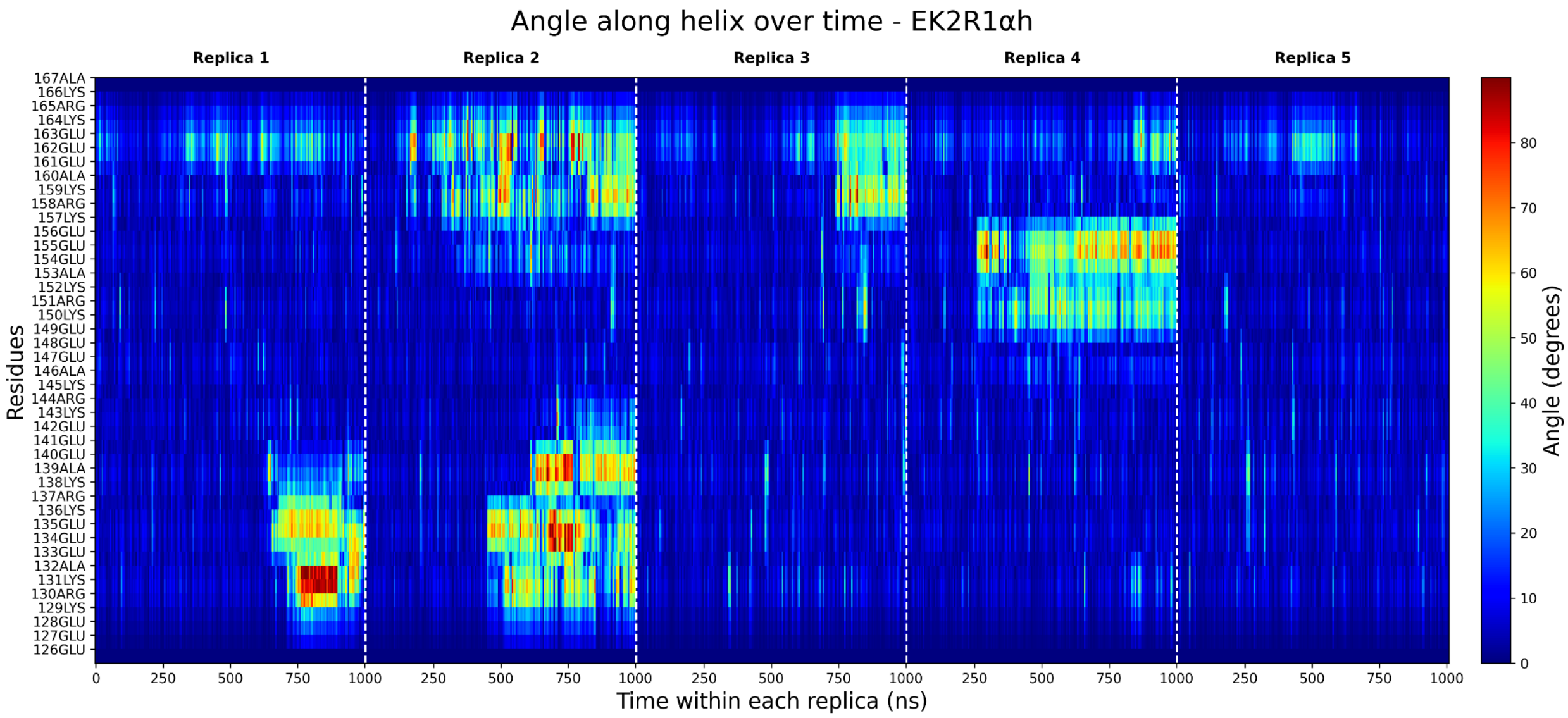

Figure S29. Same as Fig. S27 but for Amber14SB+OPC-based MD of **EK2R1αh**.

4.3. PAD analysis

As expected, the flexibility is high in turns and in coils, while it is low in the regions in helices (Fig.S30-S33).

The flexibility values of **NTDαh** and **EK2R1αh** along the sequence are similar to those observed with the Amber14SB+TIP3P-based MD (Fig.S30 and Fig.4a in the main text).

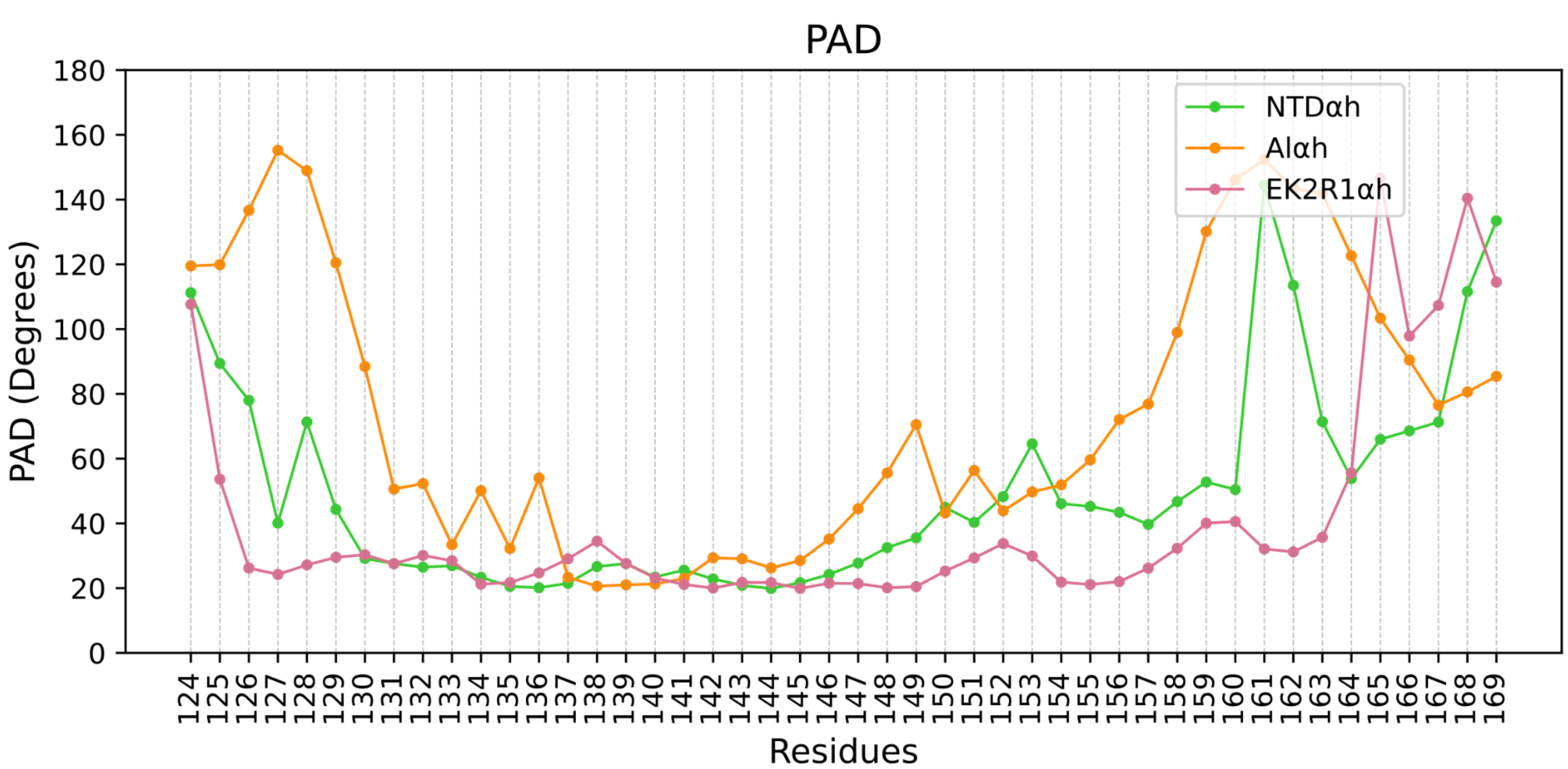

Figure S30. PAD values calculated for each residue from Amber14SB+OPC-based MD of **NTDαh**, **Alaαh** and **EK2R1αh**.

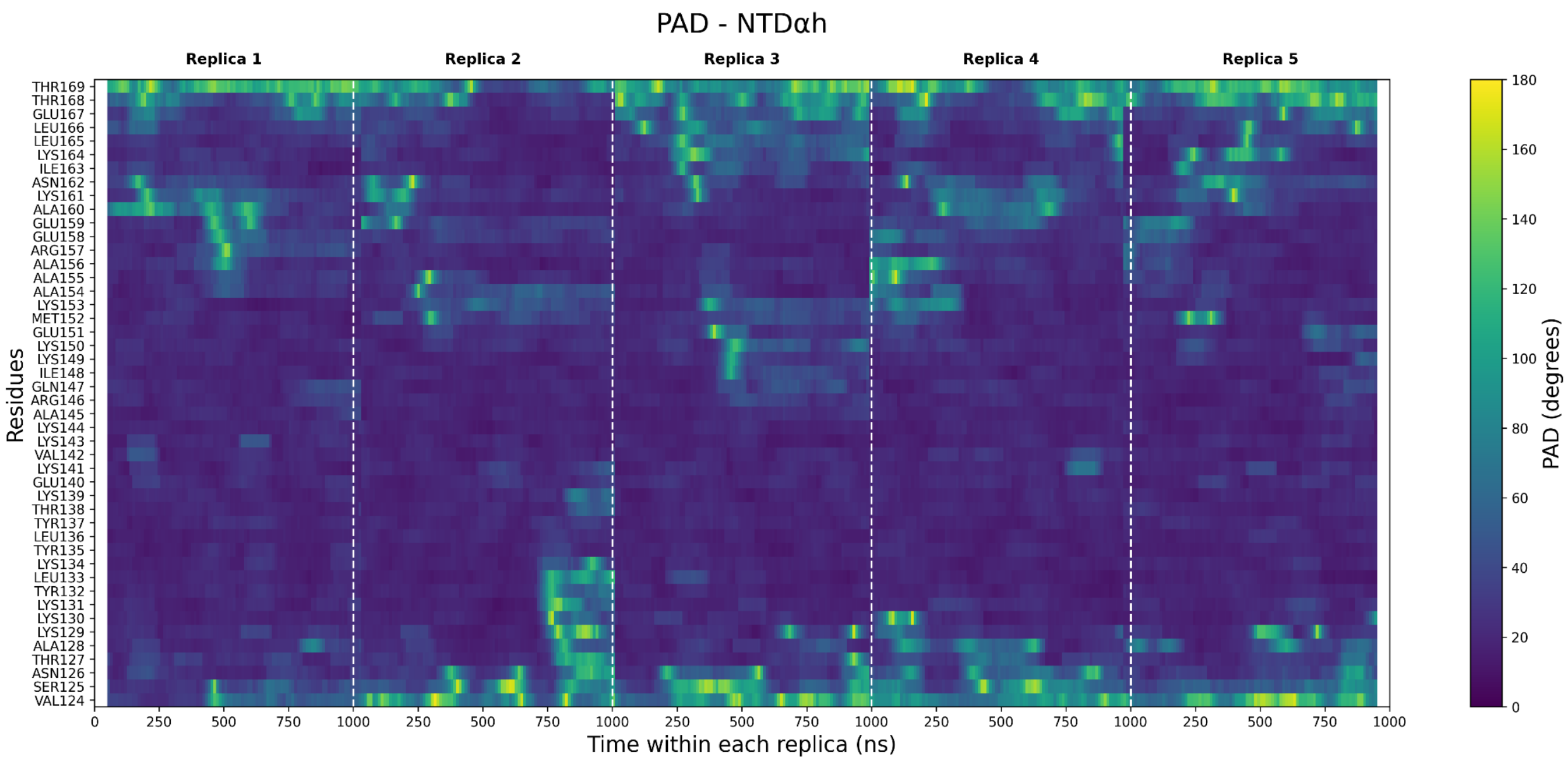

Figure S31. PAD value of each residue in the Amber14SB+OPC-based MD of **NTDαh,** plotted as a function of simulated time (5 replicas).

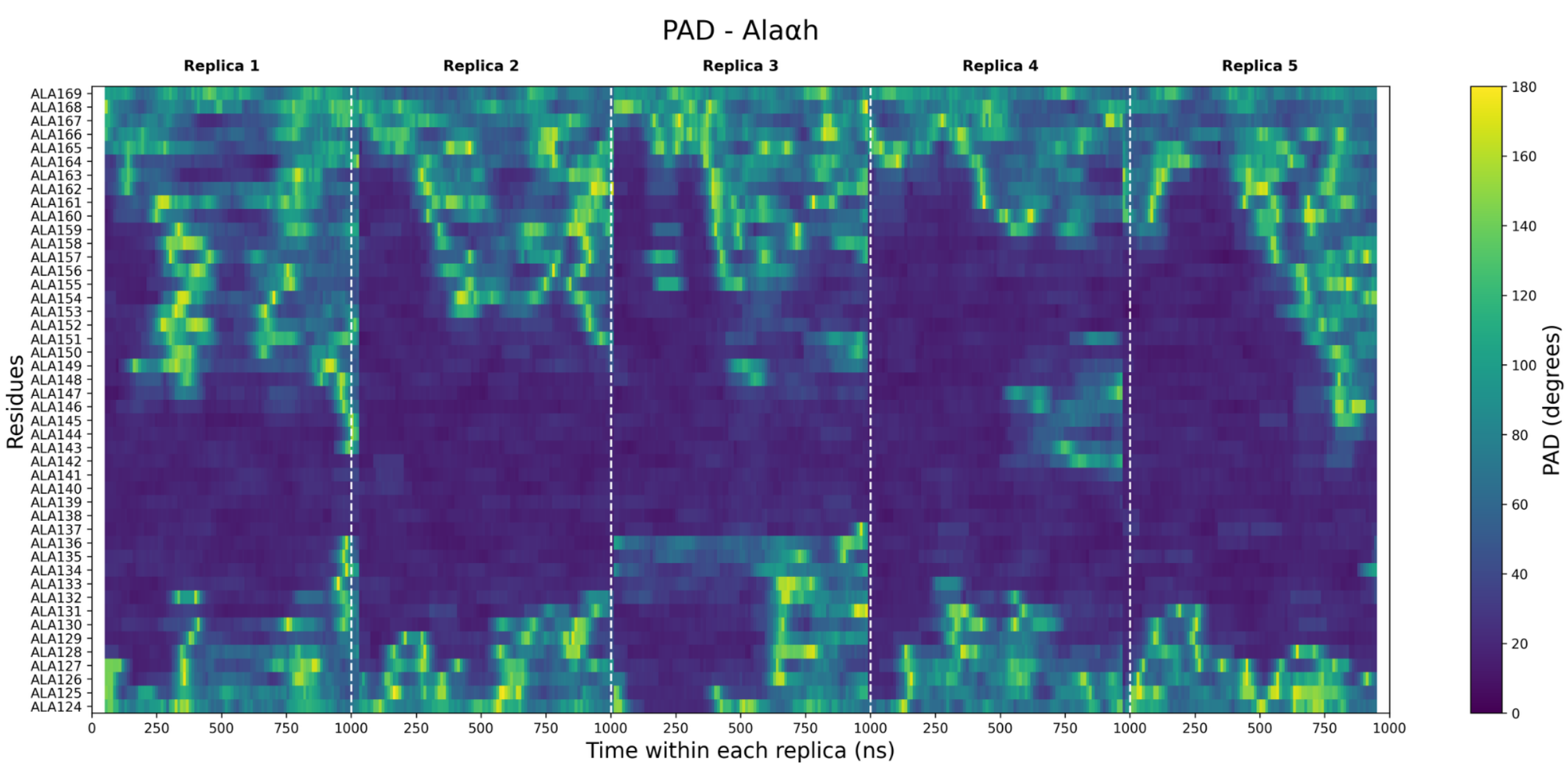

Figure S32. Same as Fig. S31 but for Amber14SB+OPC-based MD of **Alaαh**.

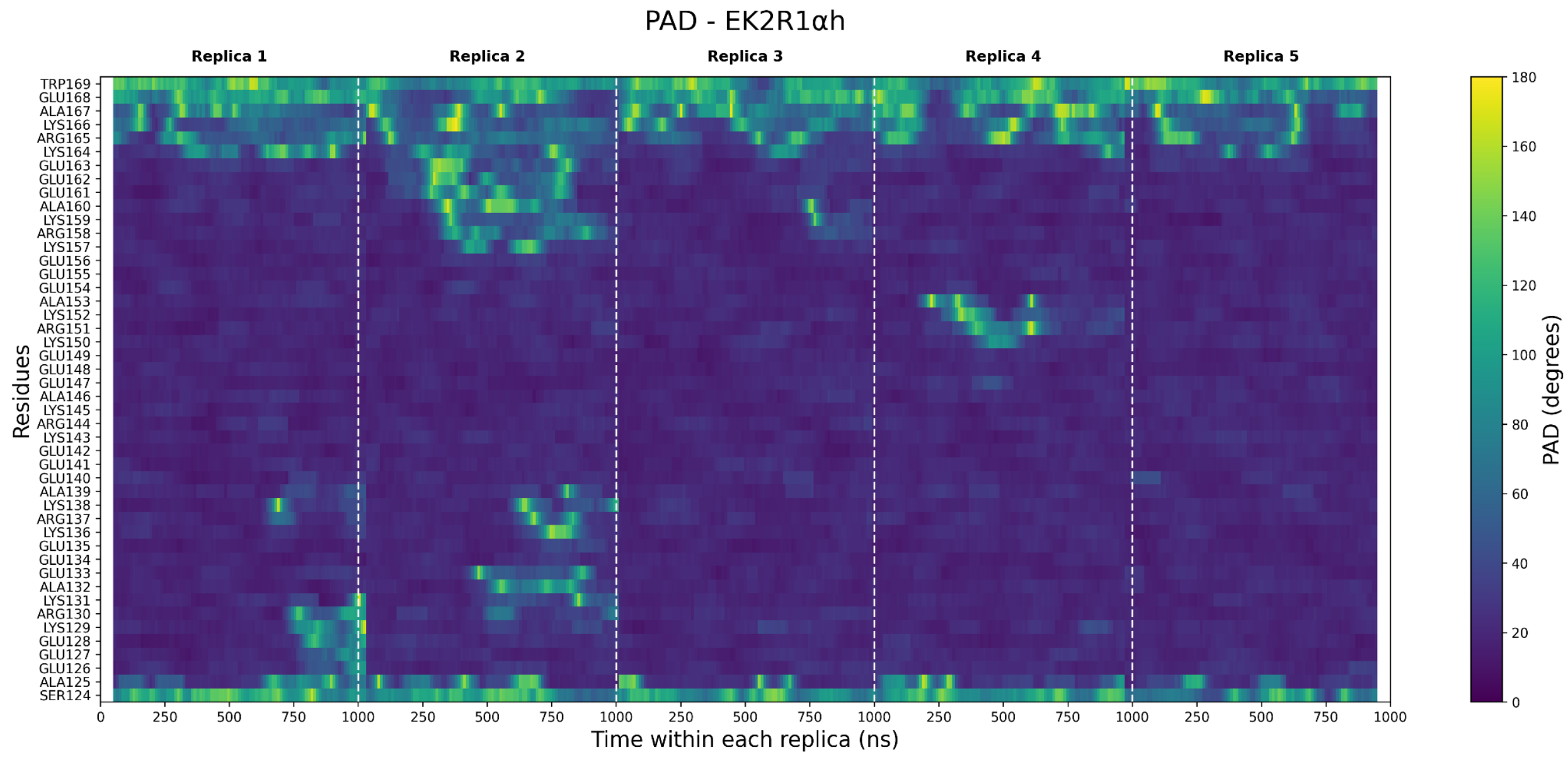

Figure S33. Same as Fig. S31 but for Amber14SB+OPC-based MD of **EK2R1αh**.

4.4. SASA analysis

The SASA values of the three systems are similar to those obtained using Amber14SB+TIP3P-based MD. As expected, the regions with low α-helical content and increased bending and flexibility have high average SASA values and fluctuations and vice versa (Fig.S34-S37).

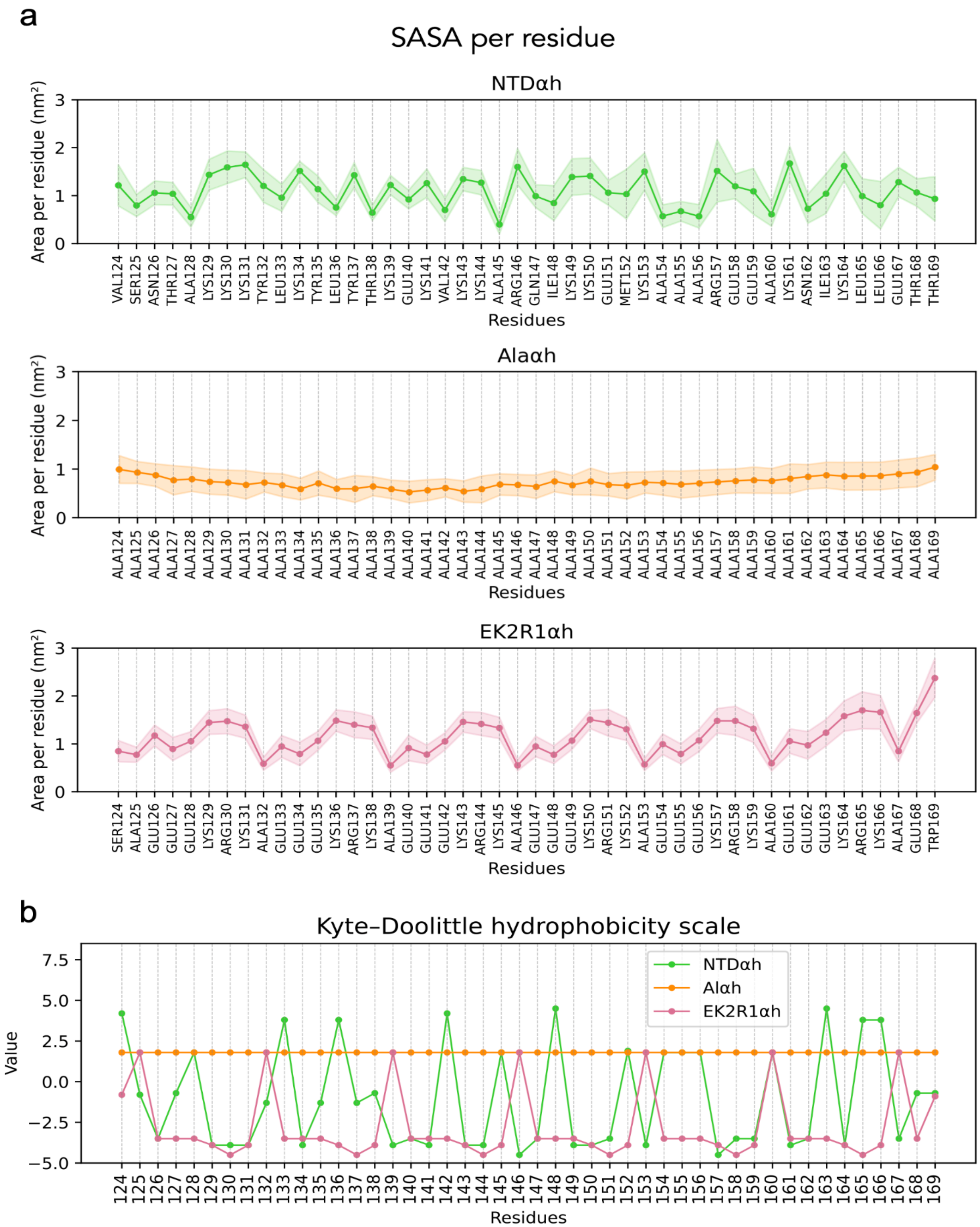

Figure S34. a Average SASA (with its standard deviation) per residue from Amber14SB+OPC-based MD of **NTDαh**, **Alaαh** and **EK2R1αh**. **b** Corresponding hydrophobicity of each residue based on the Kyte-Doolittle scale[8].

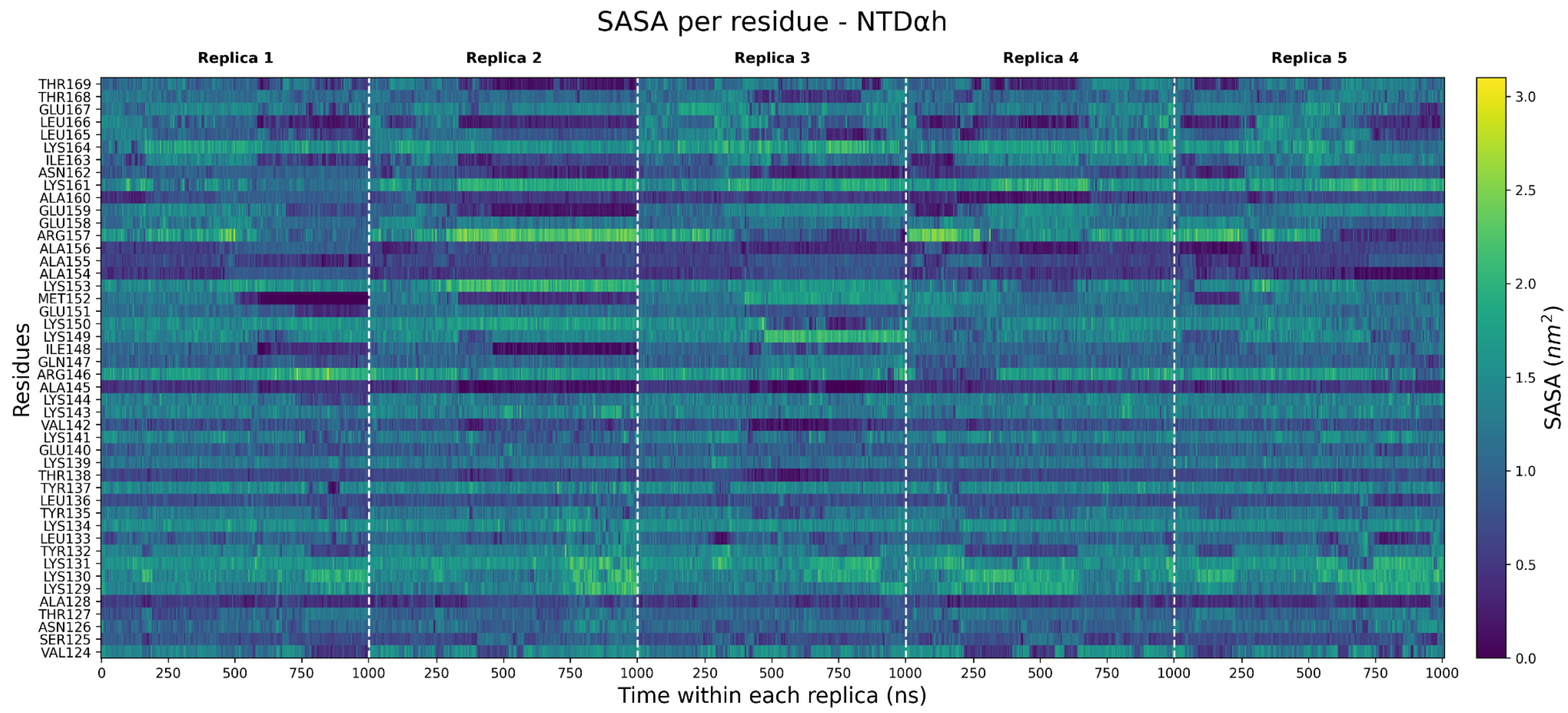

Figure S35. SASA of each residue in the Amber14SB+OPC-based MD of **NTDαh** (5 replicas) plotted as a function of simulated time.

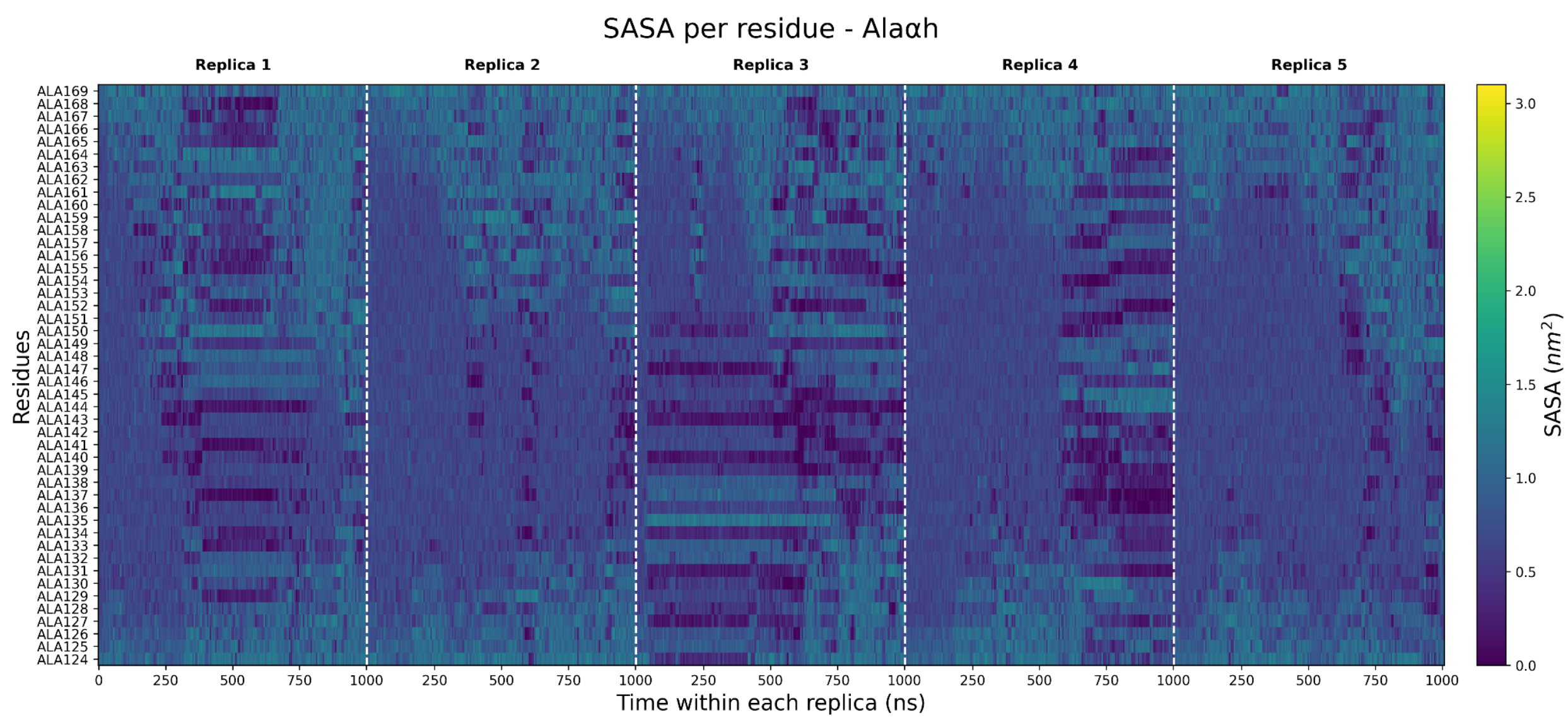

Figure S36. Same as Fig. S35 but for the Amber14SB+OPC-based MD of **Alaαh**.

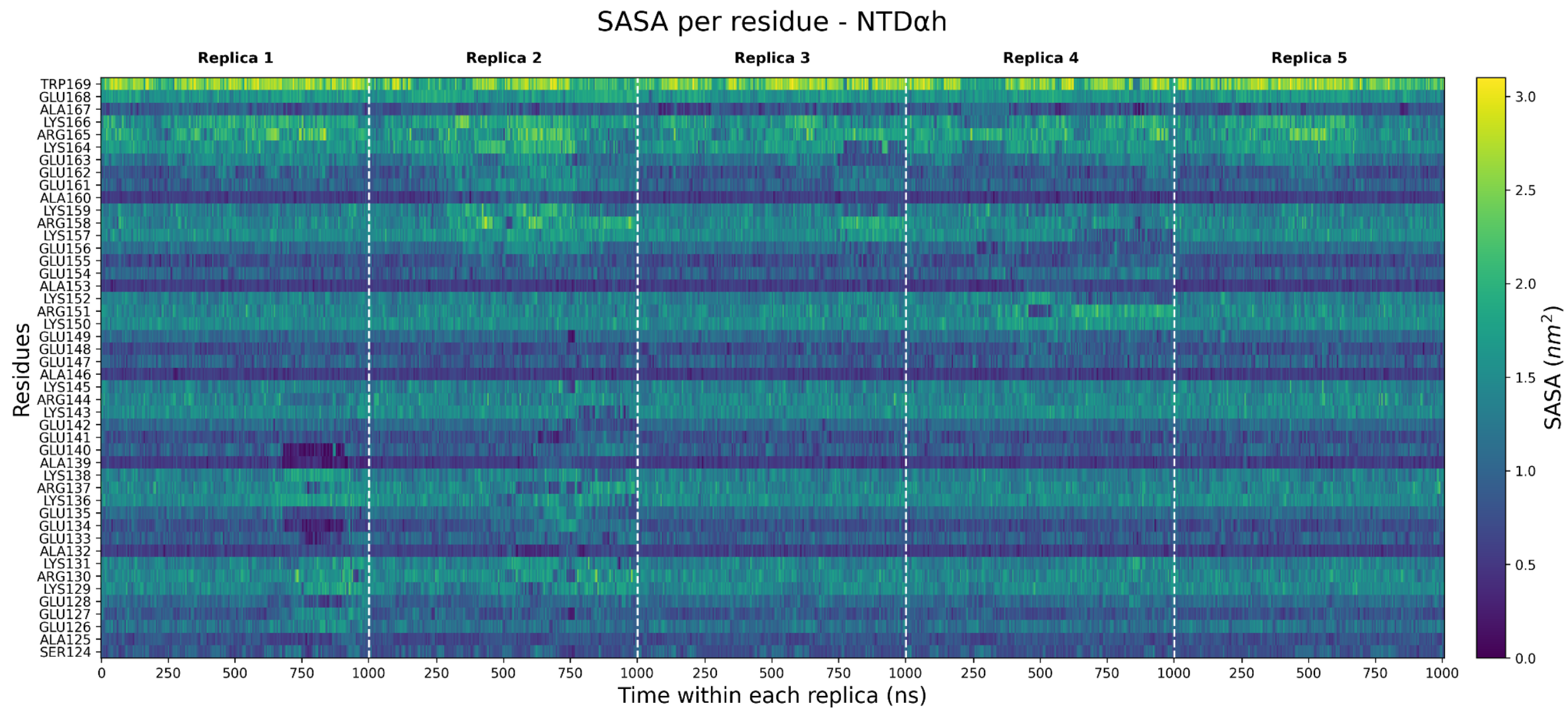

Figure S37. Same as Fig. S35 but for the Amber14SB+OPC-based MD of **EK2R1αh**.

4.5. Clustering analysis

The Amber14SB+OPC-based MD shows similar results (Tabs S5-S7 and Figs S38-S40) as those of Amber14SB+TIP3P-based MD: **EK2R1αh** has the smallest number of clusters and a dominant cluster accounting for ~58% of the trajectory. **Alaαh** has the largest number of clusters without a dominant conformation. **NTDαh** shows intermediate conformational diversity with 7 dominant clusters accounting for ~50% of the trajectory.

Cluster	Population
1	18.0%
2	9.6%
3	8.4%
4	6.9%
5	4.9%
6	4.7%
7	3.8%
Other (93 clusters)	43.7%

Table S5. Cluster populations from the Amber14SB+OPC-based MD of **NTDαh**.

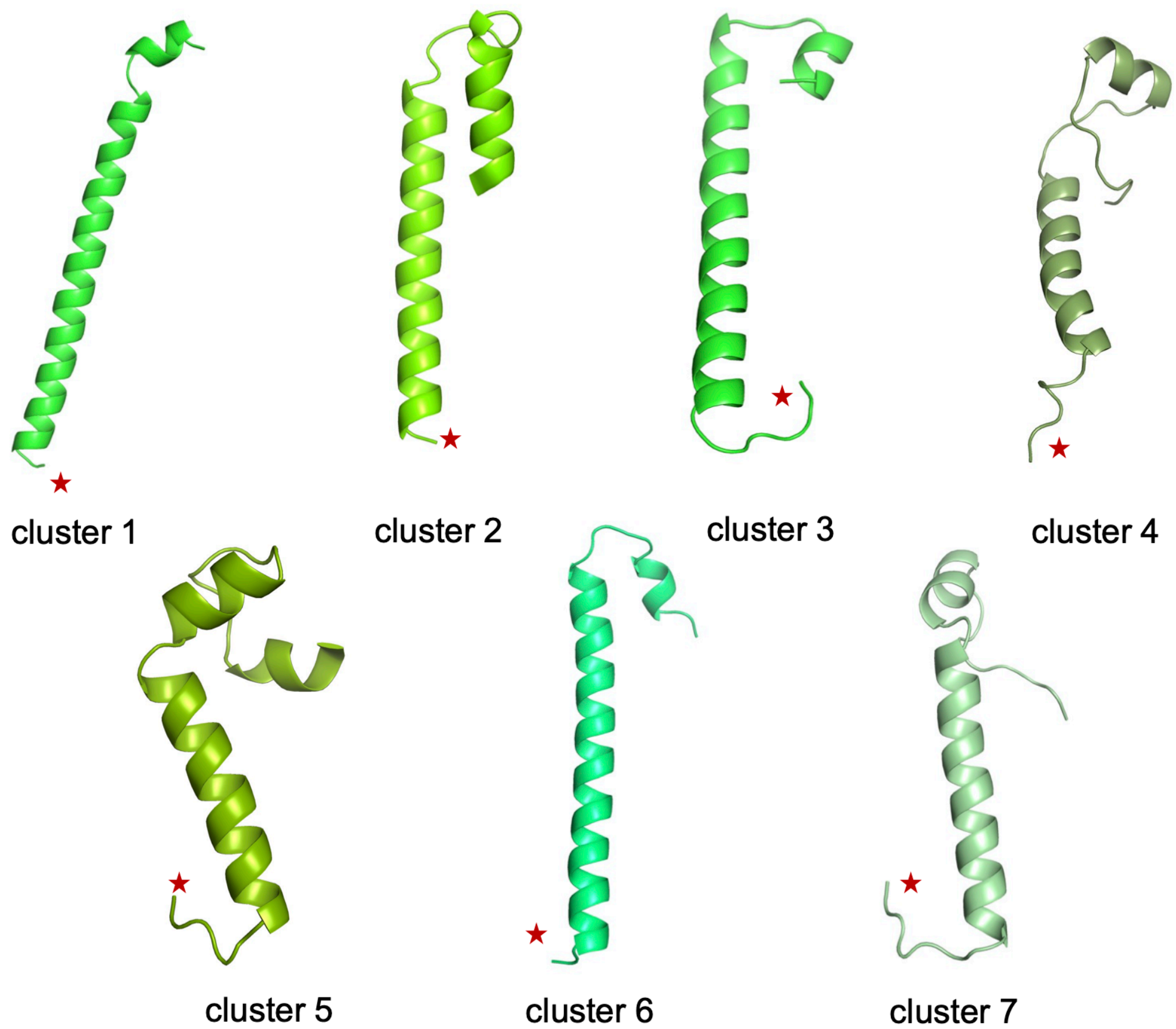

Figure S38. Representative structure of each cluster from the Amber14SB+OPC-based MD of **NTDαh**. The red star highlights the N-terminus.

Cluster	Population
1	10.4%
2	6.2%
3	4.9%
4	3.9%
5	3.3%
6	2.8%
Other (406 clusters)	68.5%

Table S6. Same as Tab. S5 but for the Amber14SB+OPC-based MD of **Alaαh.**

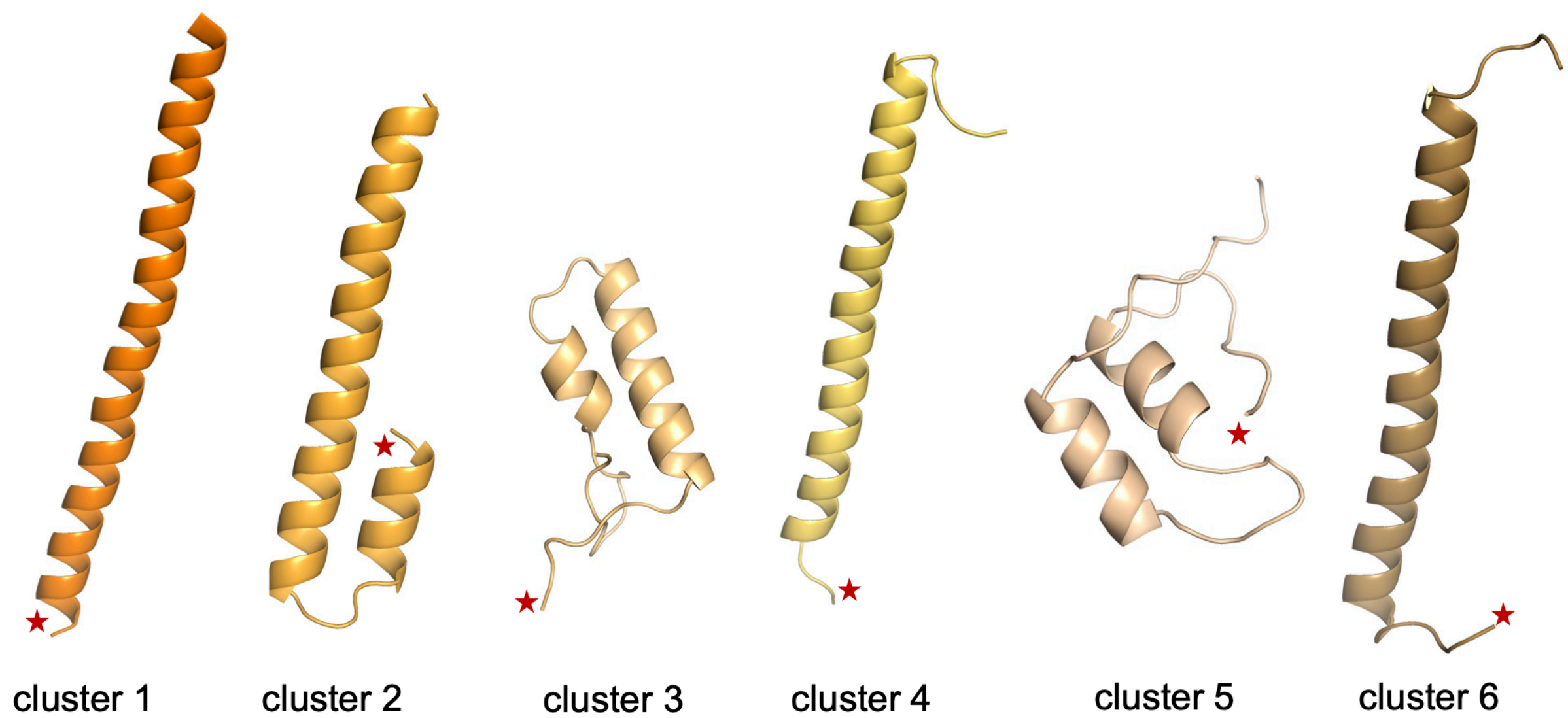

Figure S39. Same as Fig. S38 but for the Amber14SB+OPC-based MD of **Alaαh**.

Cluster	Population
1	58.2%
2	7.5%
3	4.2%
Other (84 clusters)	30.1%

Table S7. Same as Tab. S5 but for the Amber14SB+OPC-based MD of **EK2R1αh**.

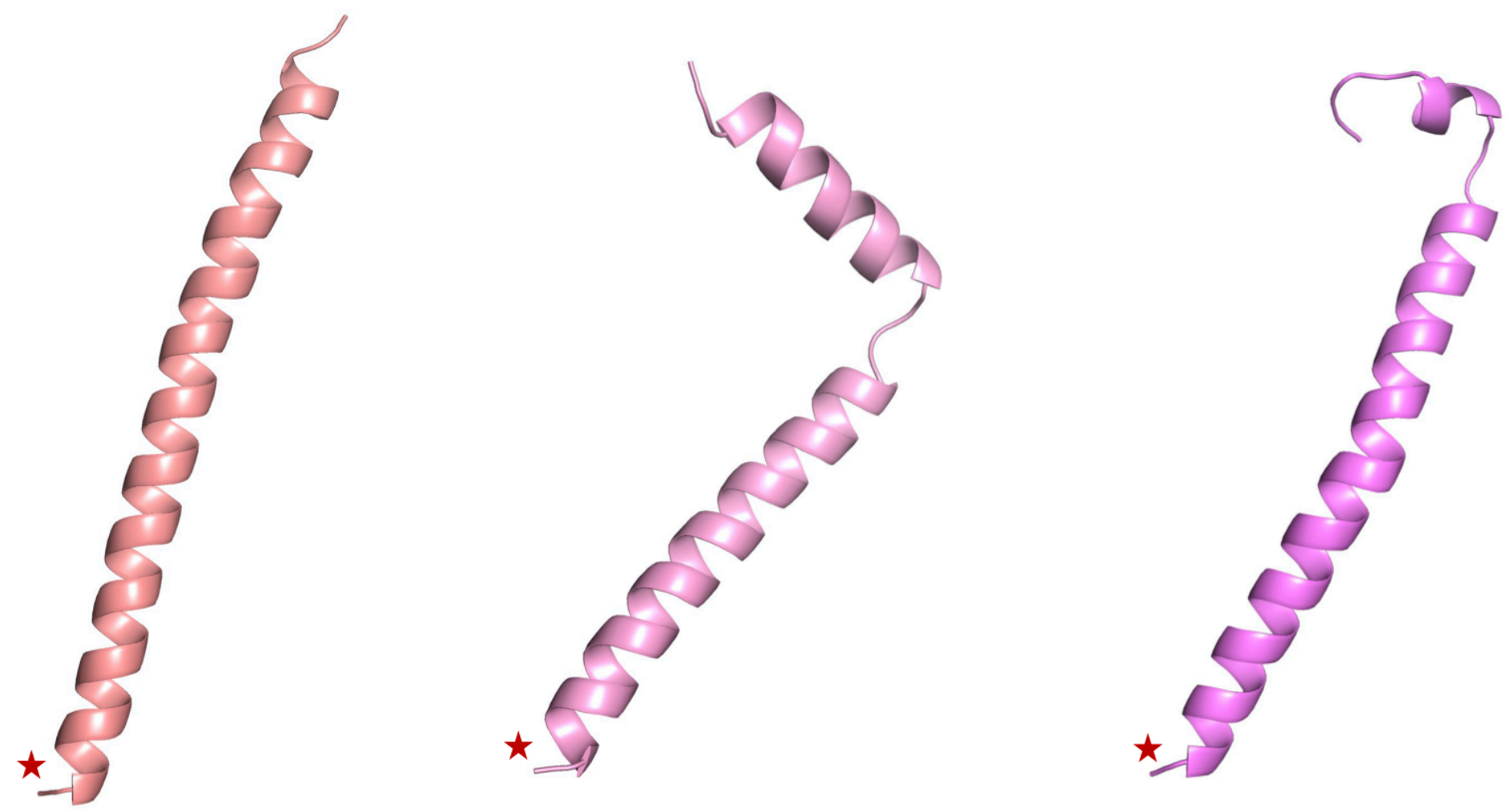

Figure S40. Same as Fig. S38 but for the Amber14SB+OPC-based MD of **EK2R1αh**.

4.6. PCA analysis

The same results obtained from the Amber14SB+TIP3P-based simulations (main text) were obtained with Amber14SB+OPC-based MD (Figs.S41-S46): **EK2R1αh** shows only one basin and its representative conformation is a straight helix, indicating it is a stable α-helical structure. **NTDαh** and **Alaαh** exhibit two main basins in more scattered free energy landscapes.

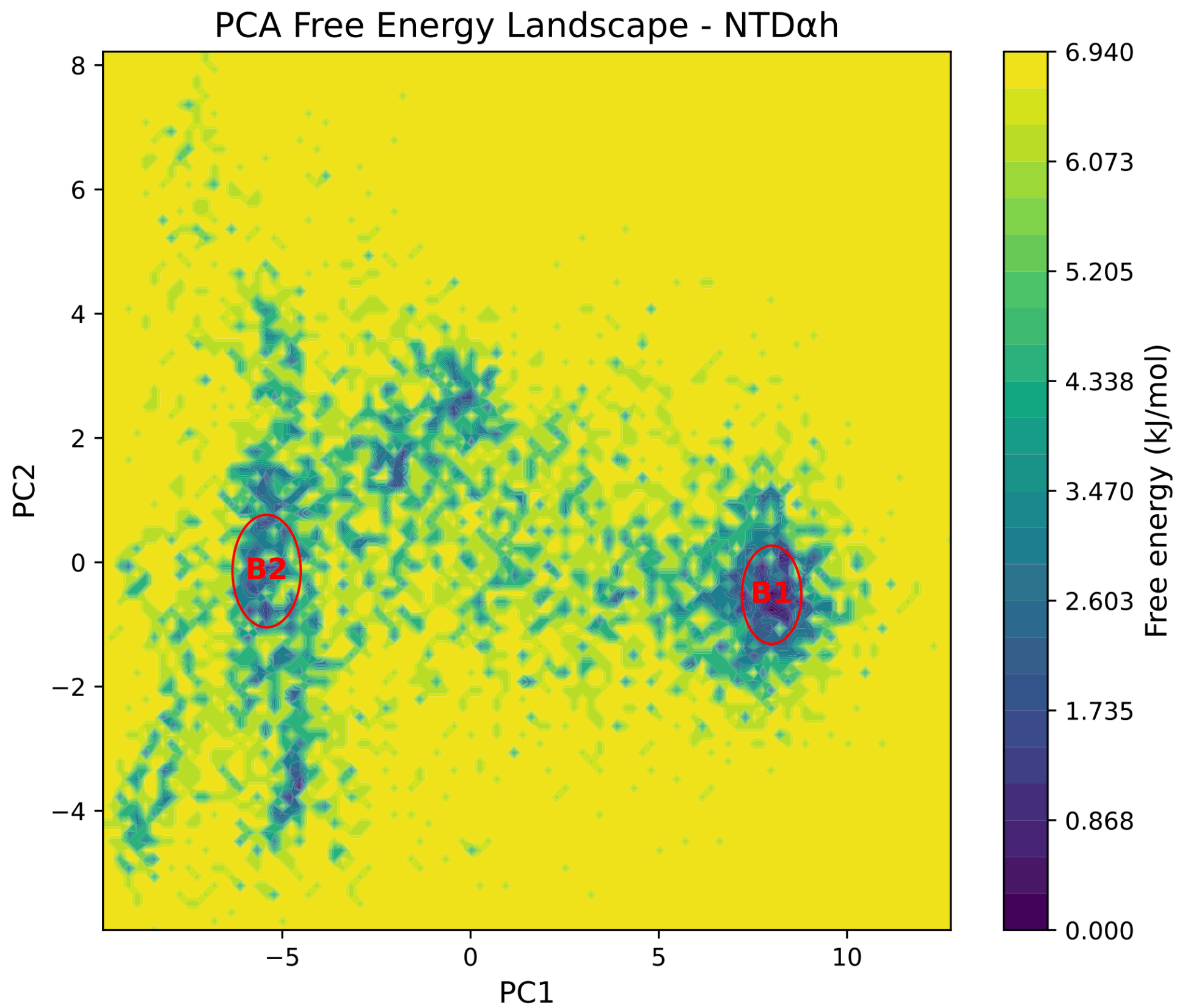

Figure S41. PCA relative free energy landscape from the Amber14SB+OPC-based MD of **NTDαh**. The minimum is set to zero. The free energy is plotted as a function of the first two principal components (PC1 and PC2). The basins B1-B2 is marked with the red circles.

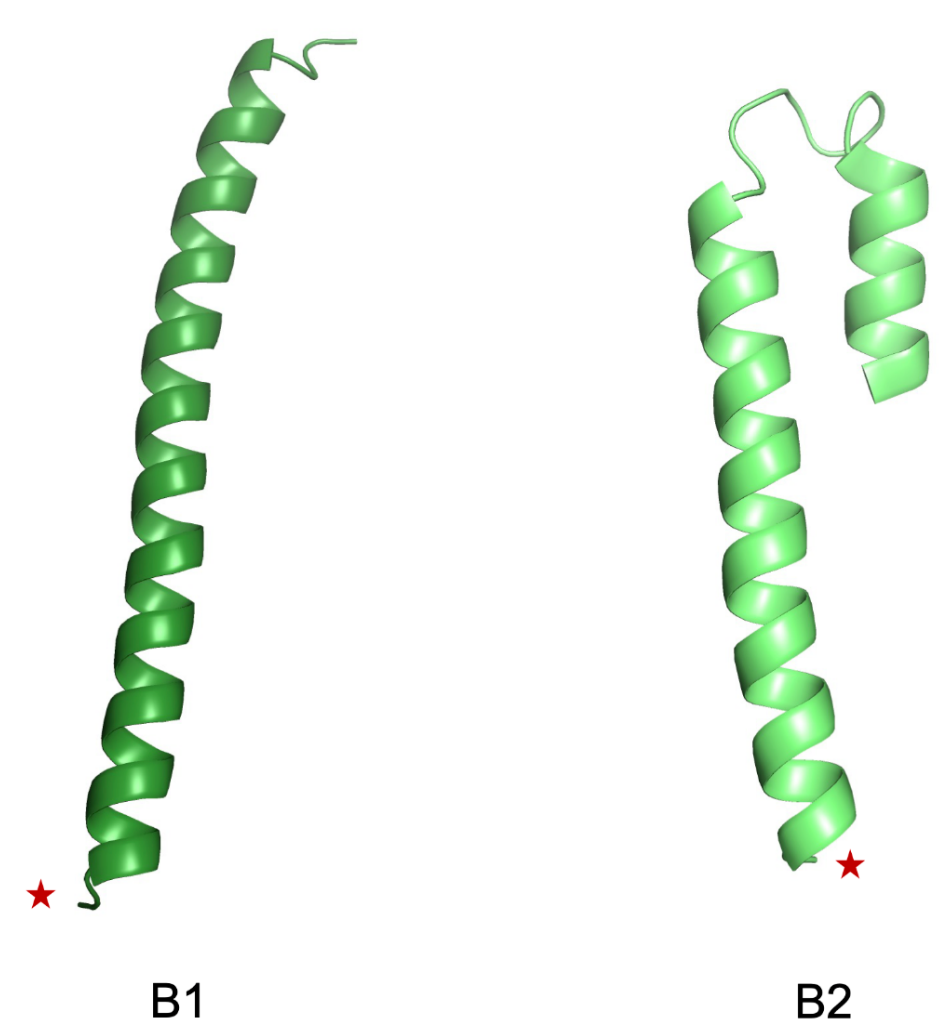

Figure S42. Representative structures from clustering analysis of the two basins in Fig.S41. The red star highlights the N-terminus.

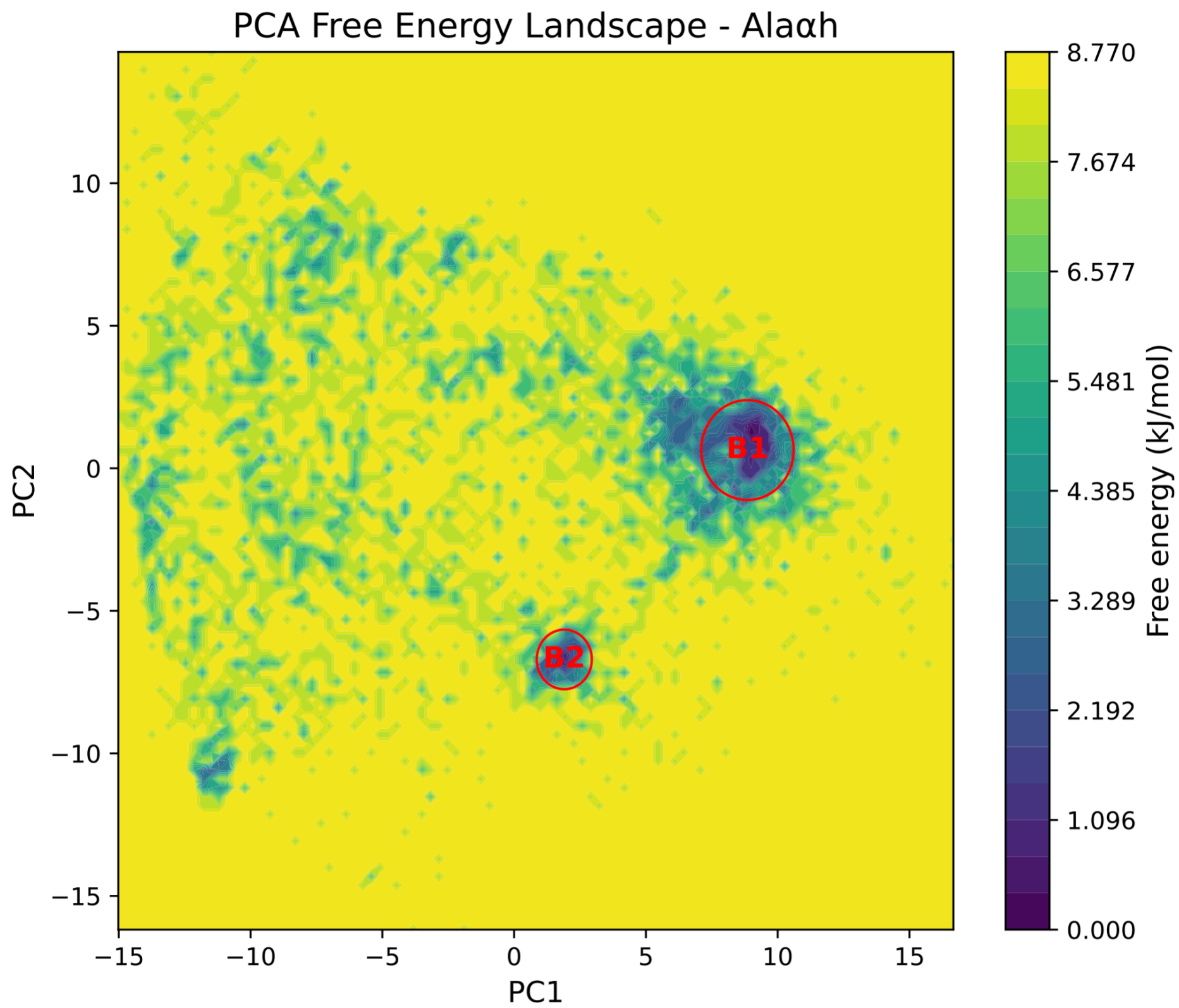

Figure S43. Same as Fig. S41 but for Amber14SB+OPC-based MD of **Alaαh.**

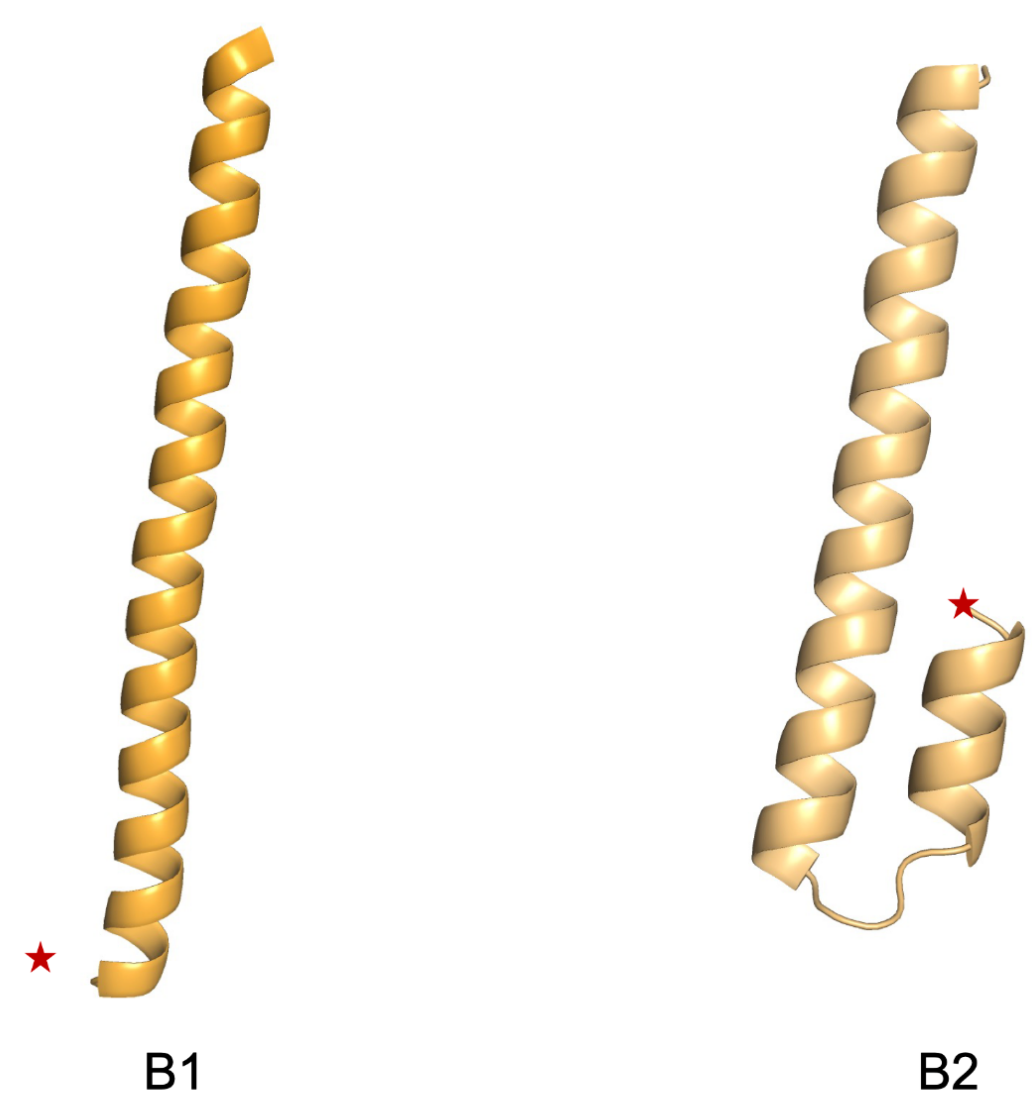

Figure S44. Representative structure from clustering analysis of two basins in Fig. S43. The red star highlights the N-terminus.

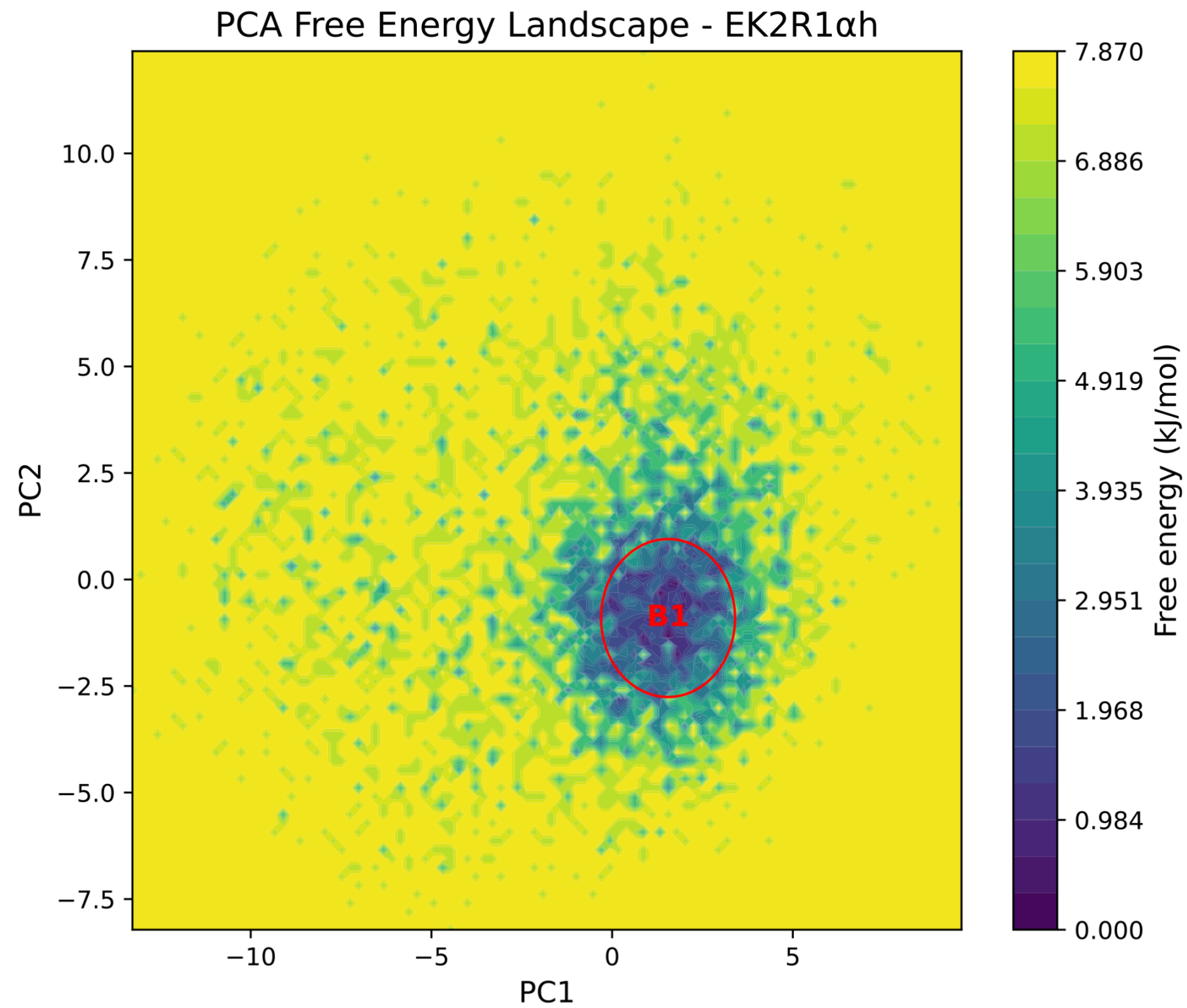

Figure S45. Same as Fig. S41 but for Amber14SB+OPC-based MD of **EK2R1αh**.

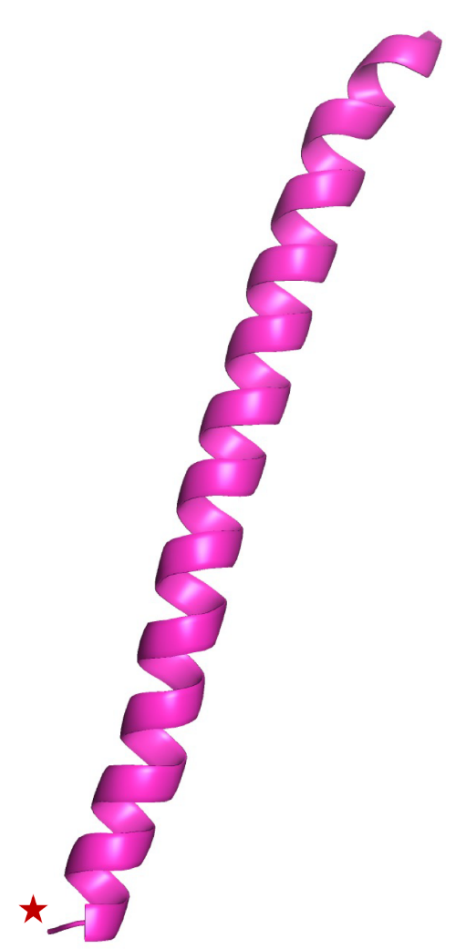

Figure S46. Representative structure from clustering analysis of basin 1 in Fig. S45. The red star highlights the N-terminus.

5. CHARMM+TIP3P - based MD simulations of NTDαh, Alaαh, EK2R1αh.

5.1 Secondary structure analysis

In our CHARMM+TIP3P[9]-based MD (Figs. S47-S50), we observe that: (i) **NTDαh** features a region with α-helical content of ~100% (from residues 132 to 139, see Fig.S47a,d); this region matches with that observed in Amber14SB+TIP3P-based MD (see Fig.2, main text). (ii) **EK2R1αh** features a region with high α-helical content (Fig.S47c,f). (iii) **Alaαh** exhibits slightly more helical conformation than that observed in the Amber14SB+TIP3P-based MD; the distribution of α-helical content does not vary along the sequence (Fig.S47b,e).

The overall ranking of α-helical composition is the same as that of the Amber14SB+OPC-based MD simulations for at least for **EK2R1αh** and **NTDαh** (helical content of ~85% and ~70%, respectively) for the CHARMM+TIP3P-based MD (Tab.S8), although in this case Alaαh has higher helical content (~85%).

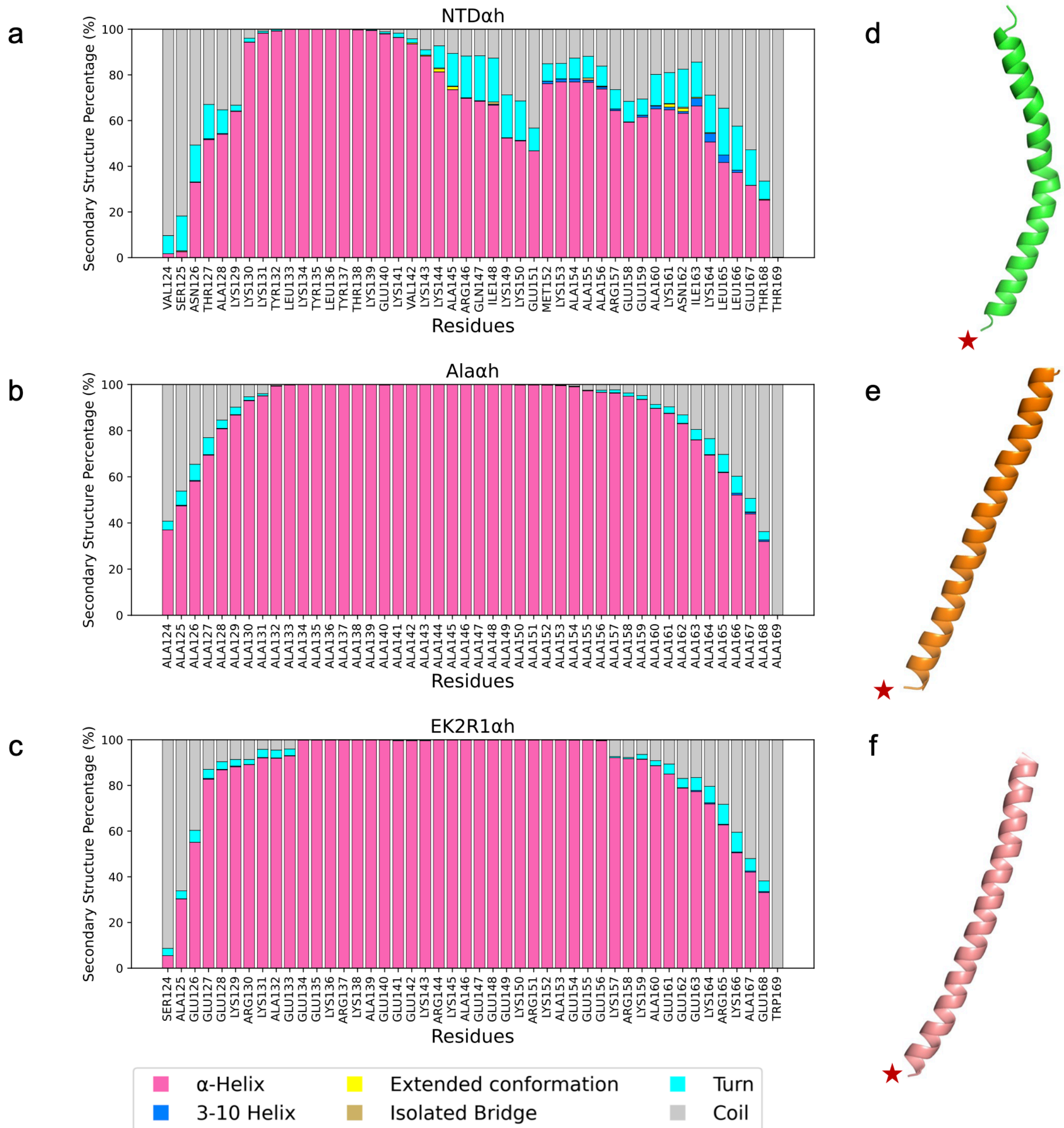

Figure S47. CHARMM+TIP3P -based MD of **NTDαh**, **Alaαh** and **EK2R1αh**, **a-c** Secondary structure composition per residue. **d-f** Corresponding representative structures, defined as the central structure of the largest cluster obtained from cluster analysis in GROMACS. The red star highlights the N-terminus.

	NTDαh	Alaαh	EK2R1αh
α-Helix	67.30 (19.94) %	85.62 (7.90) %	84.29 (8.80) %

3-10 Helix	0.53 (1.83) %	0.10 (0.84) %	0.10 (0.80) %
Extended conformation	0.17 (1.62) %	0.00 (0.00) %	0.00 (0.00) %
Isolated Bridge	0.11 (0.80) %	0.00 (0.14) %	0.00 (0.00) %
Turn	8.91 (9.69) %	1.82 (3.92) %	1.96 (4.30) %
Coil	22.98 (13.25) %	12.44 (7.17) %	13.65 (7.55) %

Table S8. Secondary structure composition over the cumulative trajectory of CHARMM+TIP3P -based MD of **NTDαh, Alaαh, EK2R1αh.** The average and standard deviation (in parentheses) of each composition across the trajectory are shown.

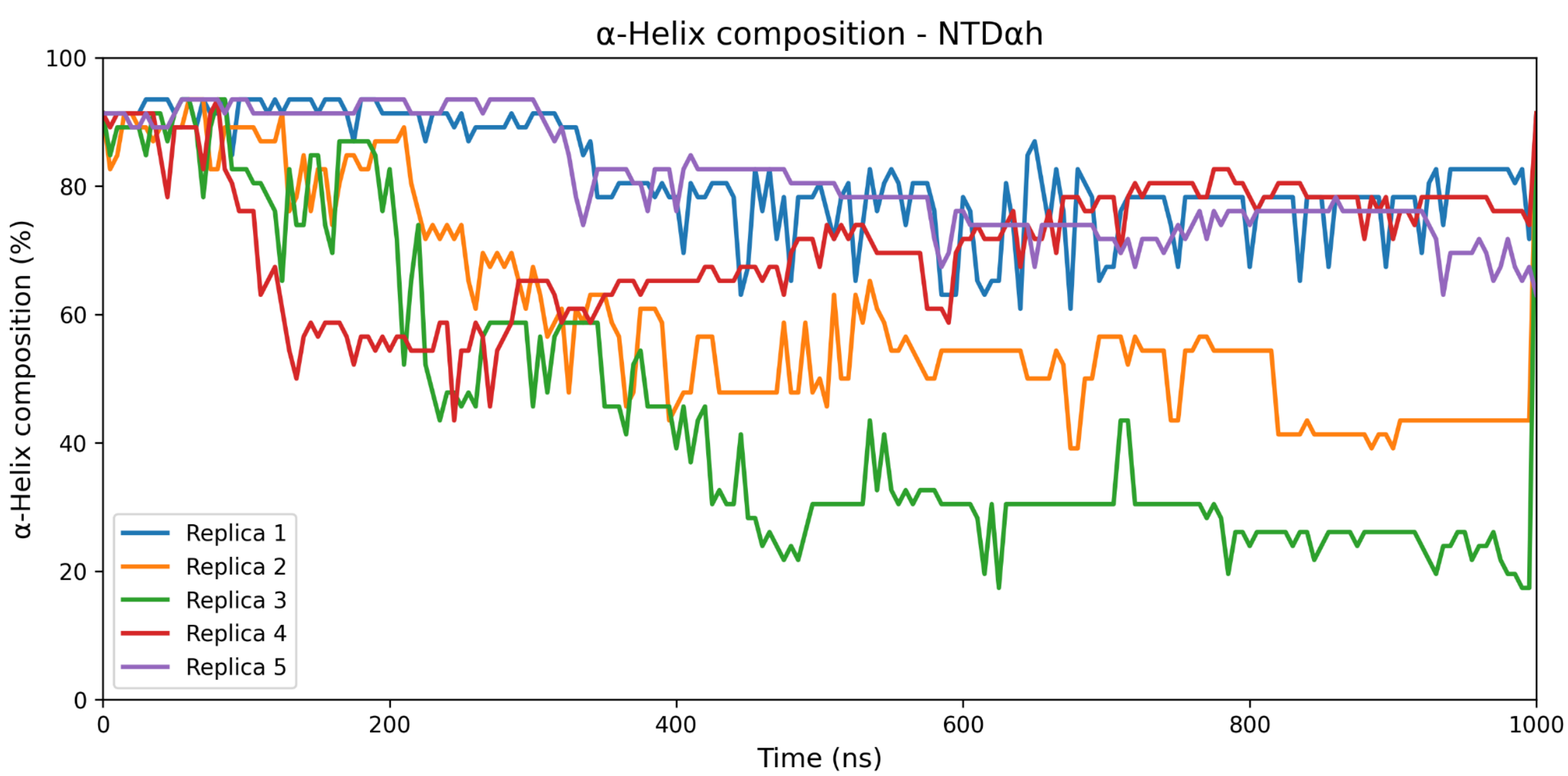

Figure S48. α-helix content of **NTDαh** in the CHARMM+TIP3P -based MD (5 replicas), plotted as a function of simulated time.

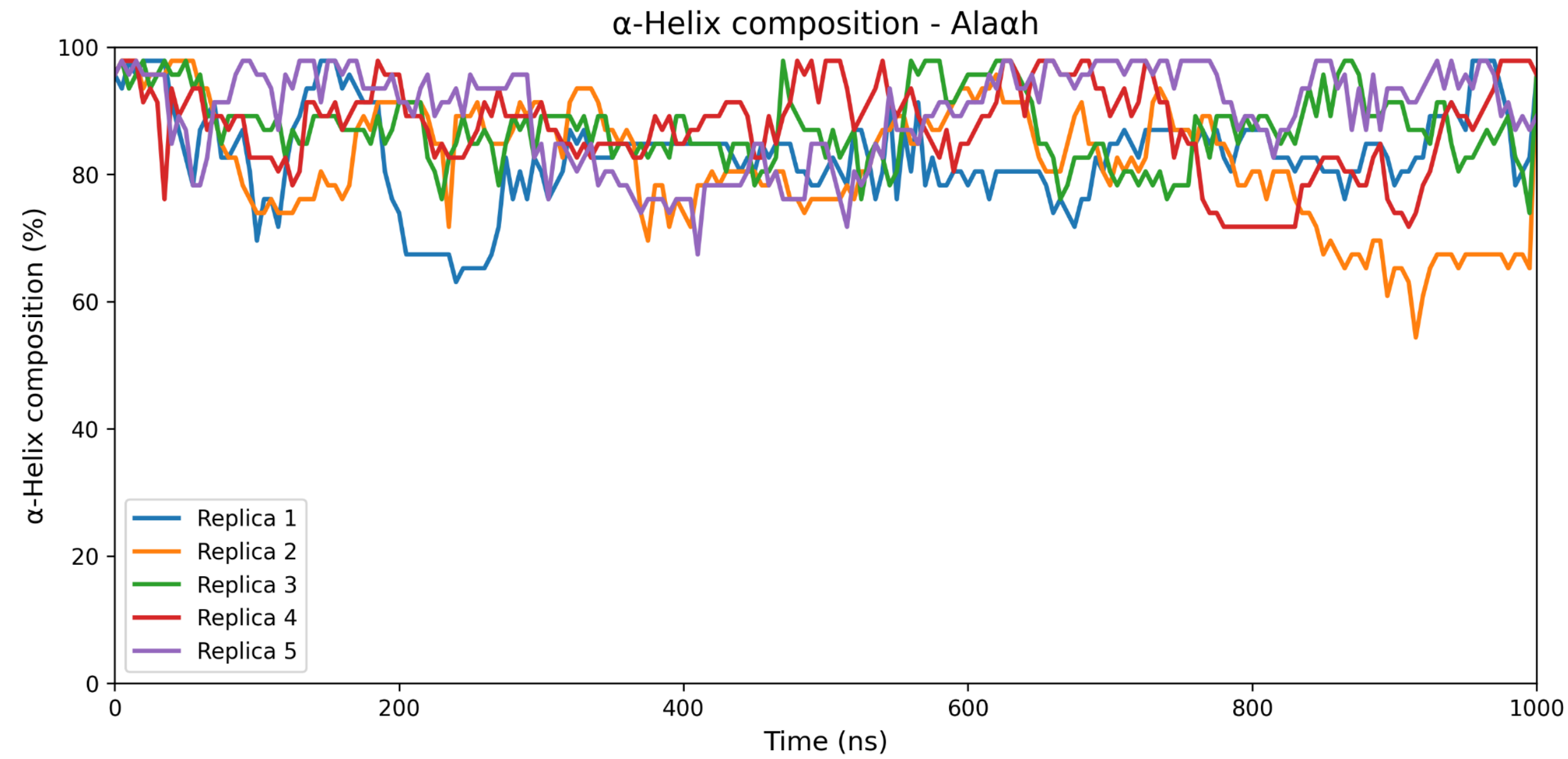

Figure S49. Same as Fig. S48 but for the CHARMM+TIP3P-based MD of **Alaαh**.

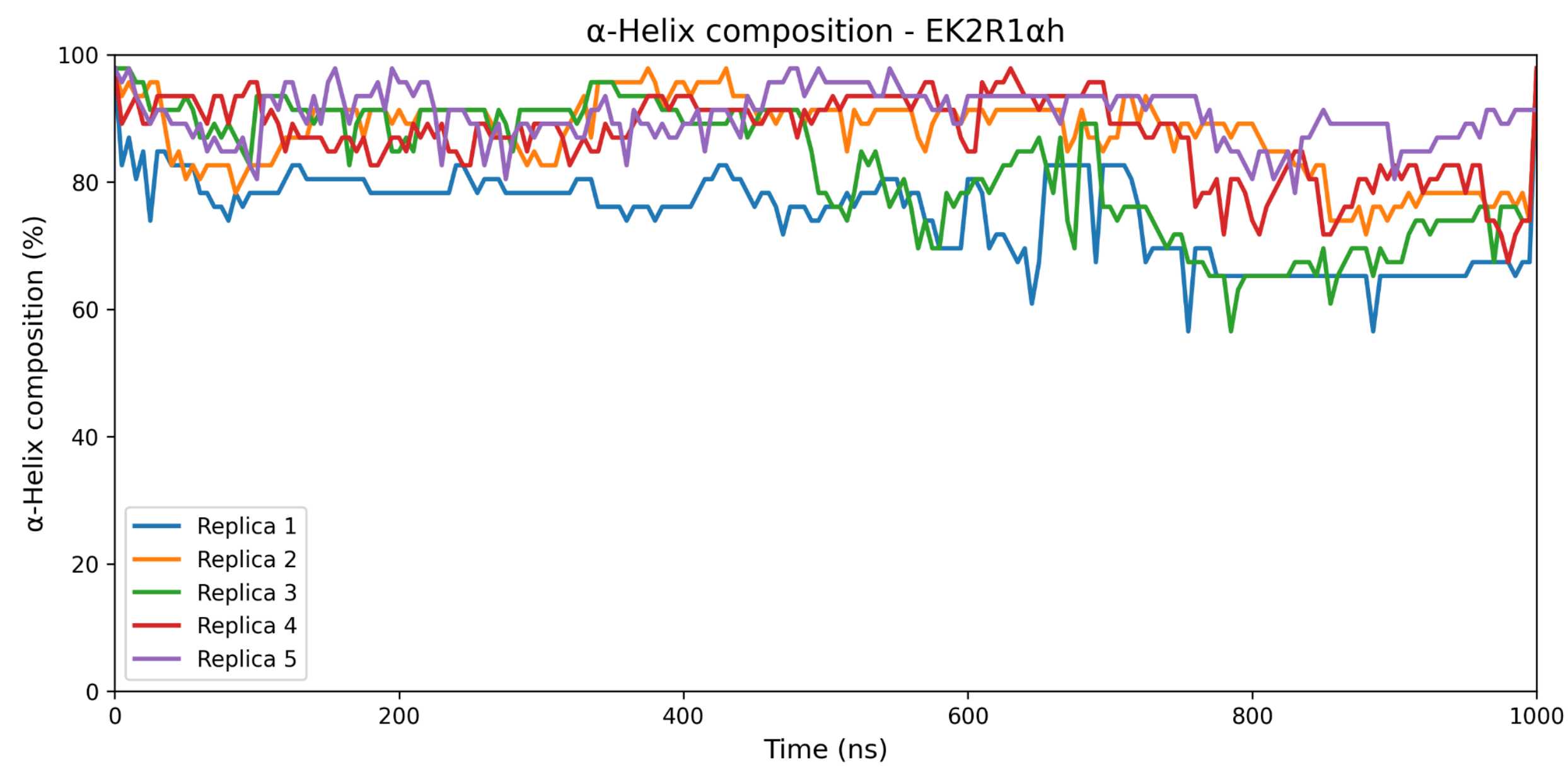

Figure S50. Same as Fig. S48 but for the CHARMM+TIP3P-based MD of **EK2R1αh**.

5.2. Bending angles

The results for CHARMM+TIP3P-based MD (Figs.S51-S54) are not too dissimilar from those above for **NTDαh** and **EK2R1αh** (Fig.S51a,d,c,f); **Alaαh** instead features low average bending angles throughout the sequence (Fig.S51b,e), similar to **EK2R1αh**.

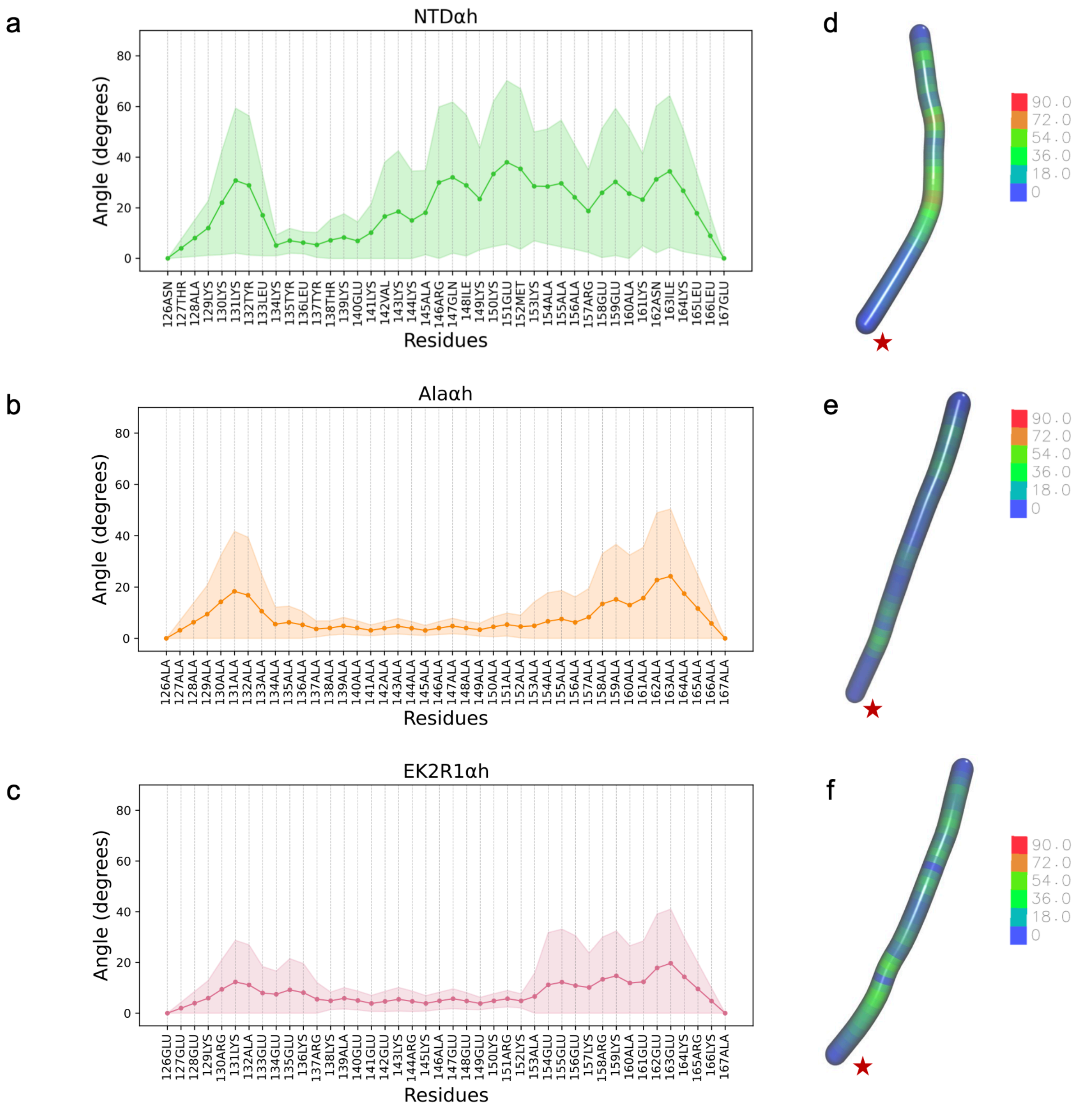

Figure S51. CHARMM+TIP3P -based MD of **NTDαh, Alaαh** and **EK2R1αh**. **a-c** Average bending angles, along with their standard deviations, along the cumulative trajectories. **d-f** Representative structures of the helices. The red color indicates the areas of highest helix axis angle, green indicates intermediate angle magnitudes and blue indicates angles close to or at 0 degrees. The red star highlights the N-terminus.

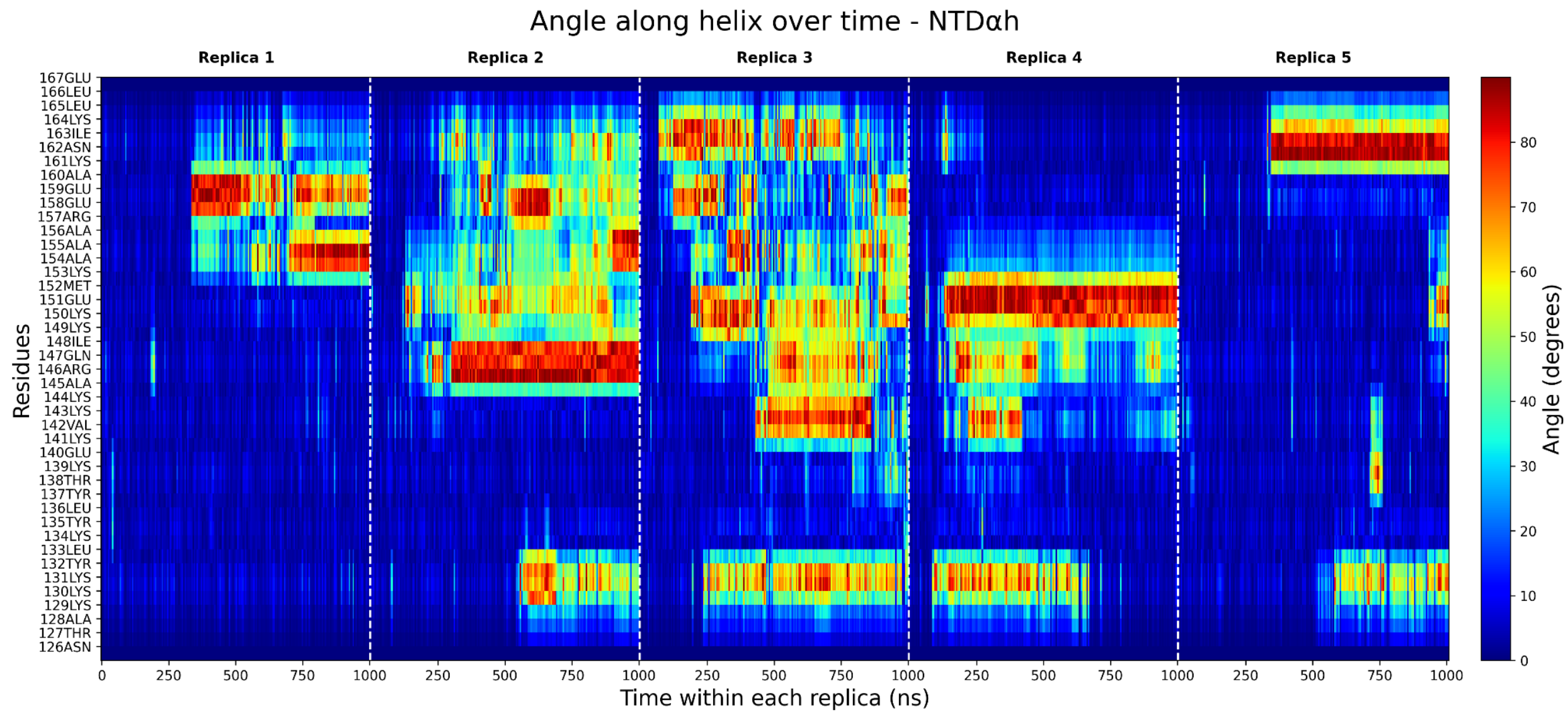

Figure S52. Bending angles for each residue of **NTDαh** in the CHARMM+TIP3P -based MD simulations of 5 replicas, plotted as a function of simulated time.

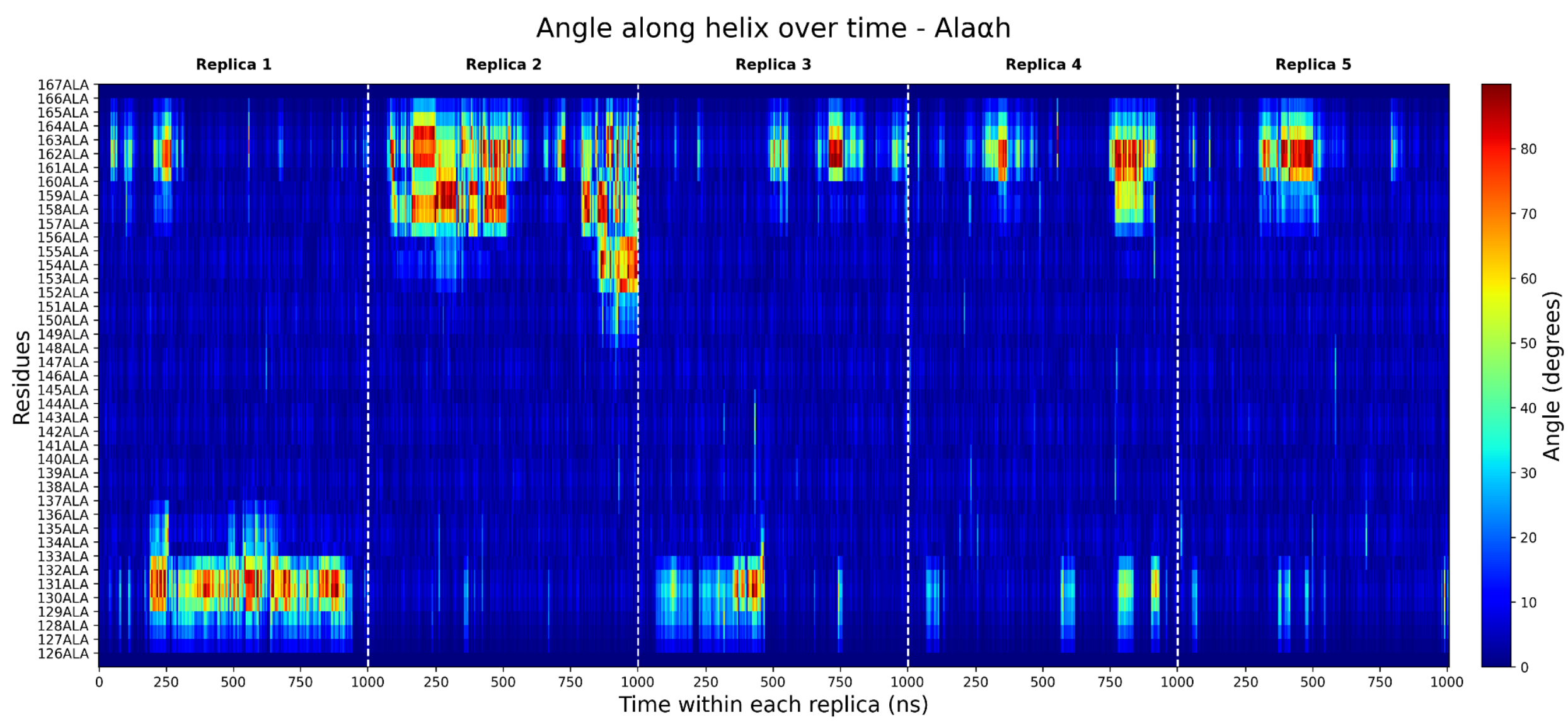

Figure S53. Same as Fig. S52 but for CHARMM+TIP3P-based MD of **Alaαh**.

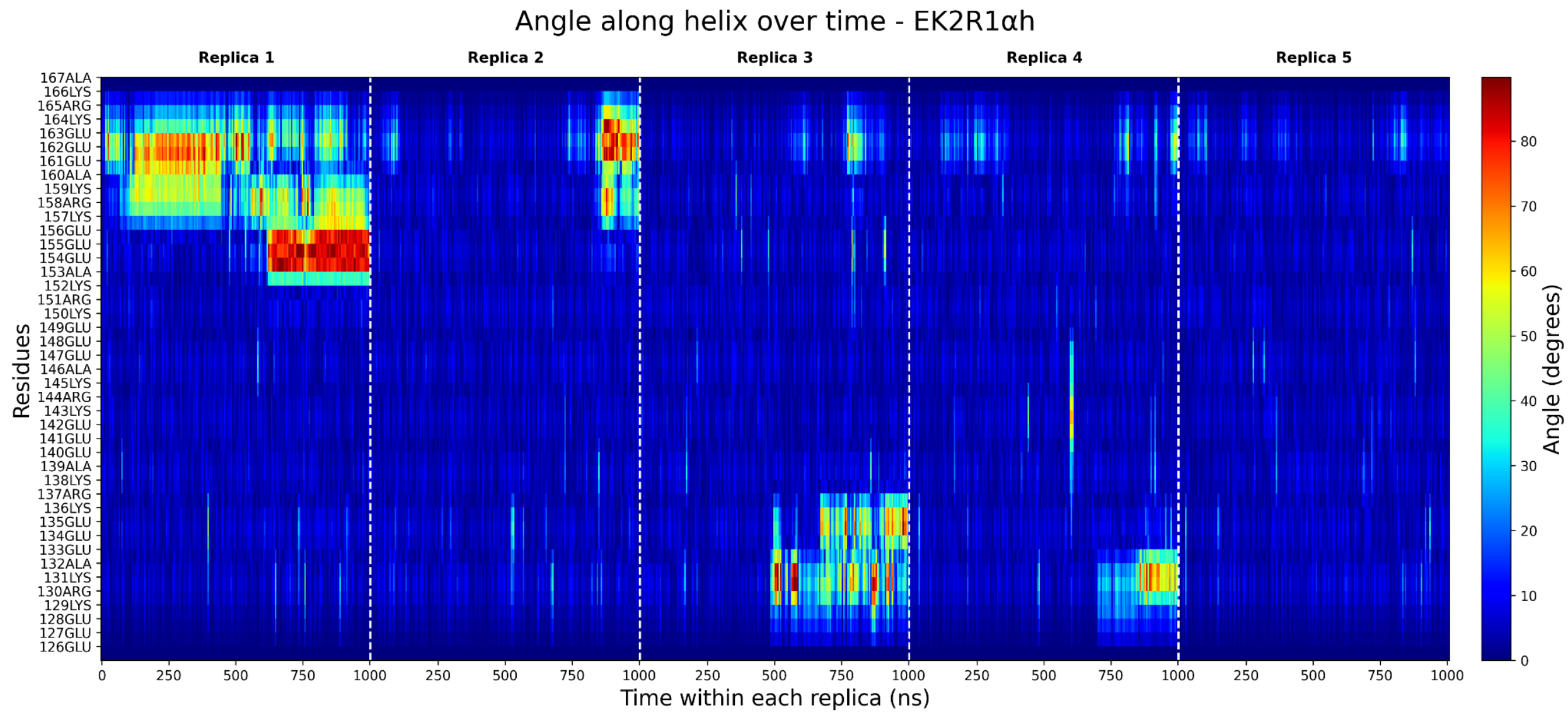

Figure S54. Same as Fig. S52 but for CHARMM+TIP3P-based MD of **EK2R1αh**.

5.3. PAD analysis

As expected, the flexibility is high in turns and in coils, while it is low in the regions in helices (Fig.S55-S58).

The flexibility values of **NTDαh** and **EK2R1αh** along the sequence are similar to those observed with the Amber14SB+TIP3P-based MD (Fig.S55-S57 and Fig.4a in the main text); however, the values of **Alaαh** are similar only for the Amber14SB+OPC-based MD, while those in CHARMM+TIP3P-based MD are lower (Fig. S55, S58).

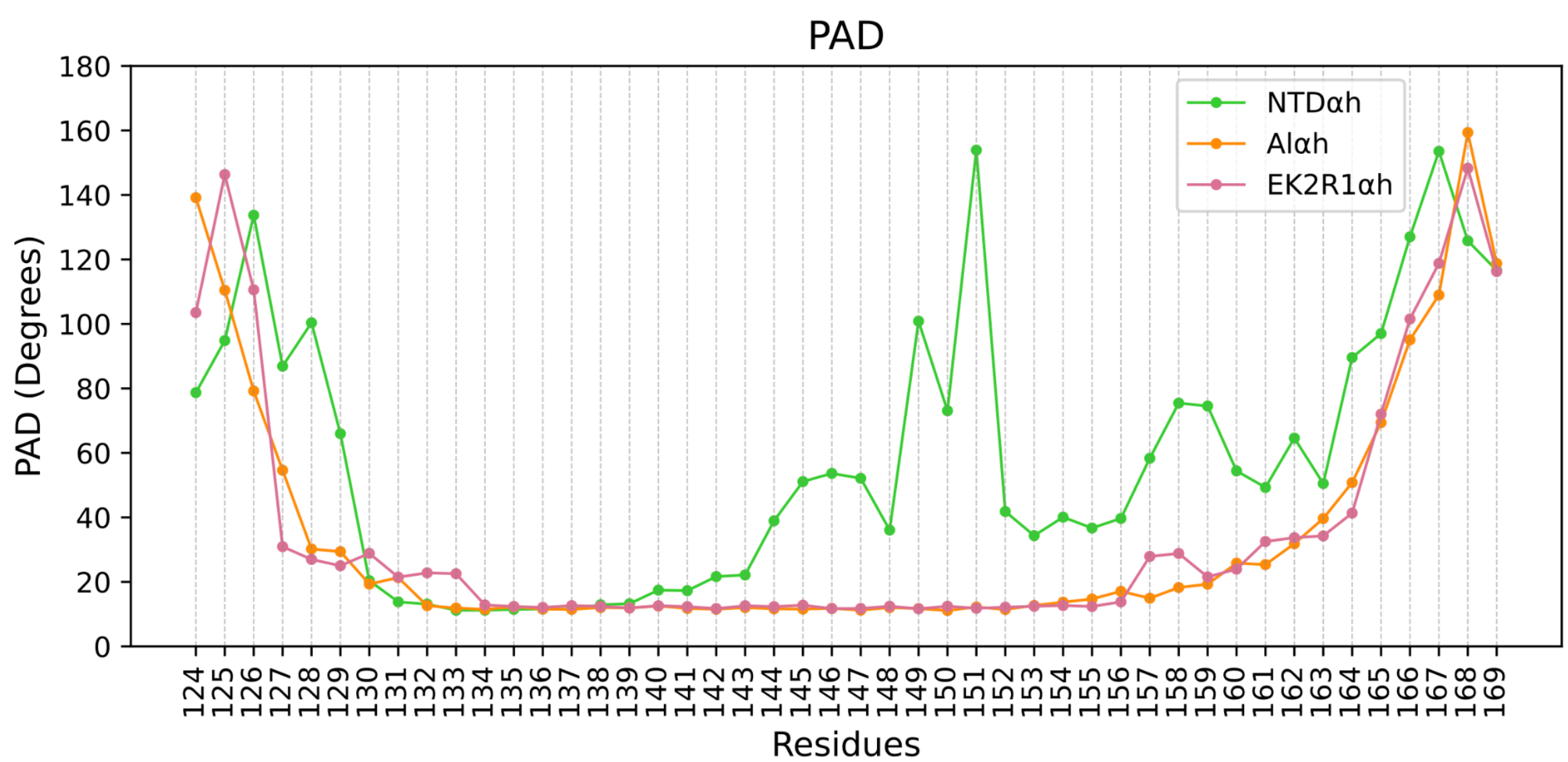

Figure S55. PAD values calculated for each residue from CHARMM+TIP3P -based MD of **NTDαh**, **Alaαh** and **EK2R1αh**.

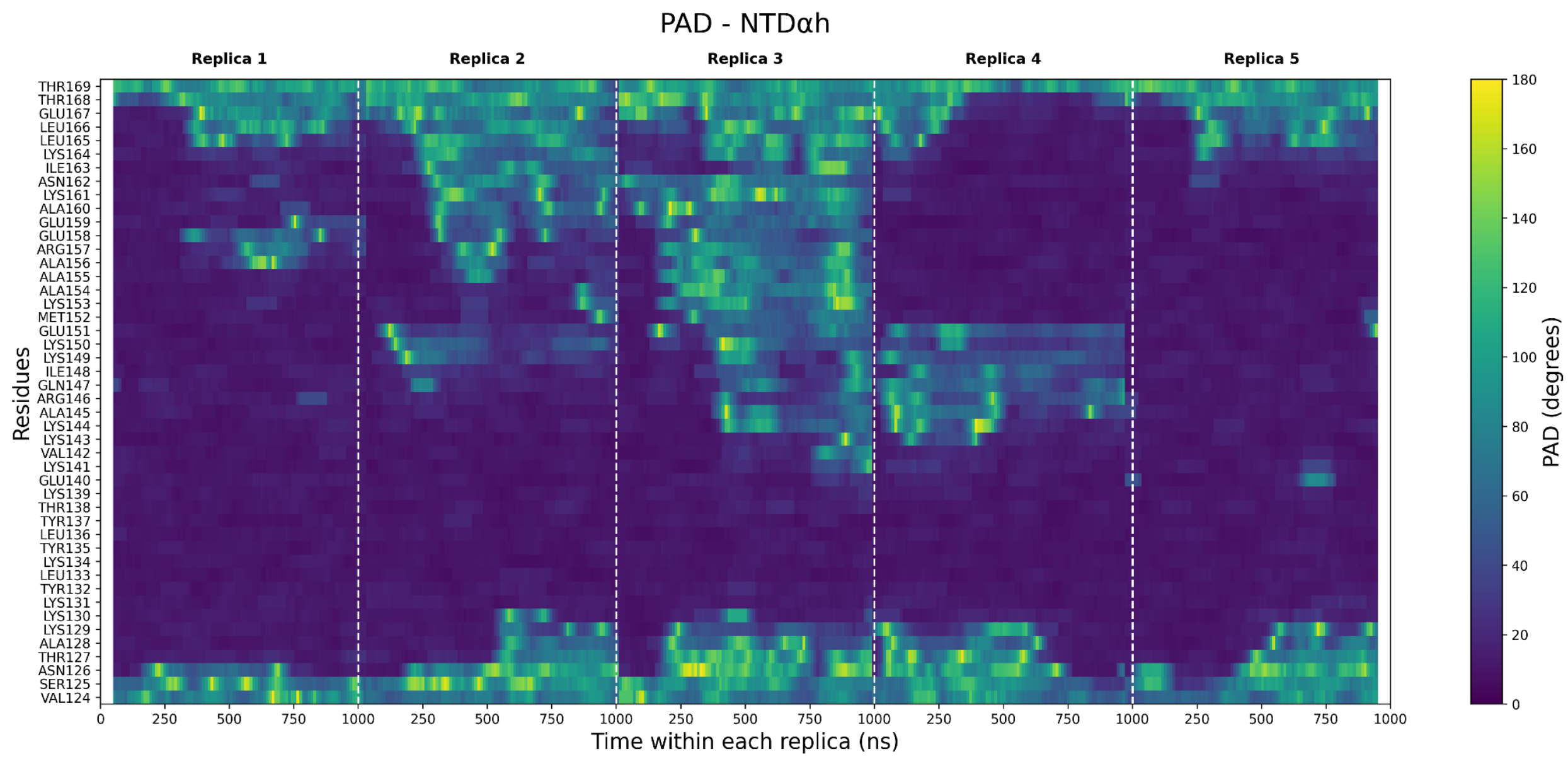

Figure S56. PAD value of each residue in the CHARMM+TIP3P -based MD of **NTDαh,** plotted as a function of simulated time (5 replicas).

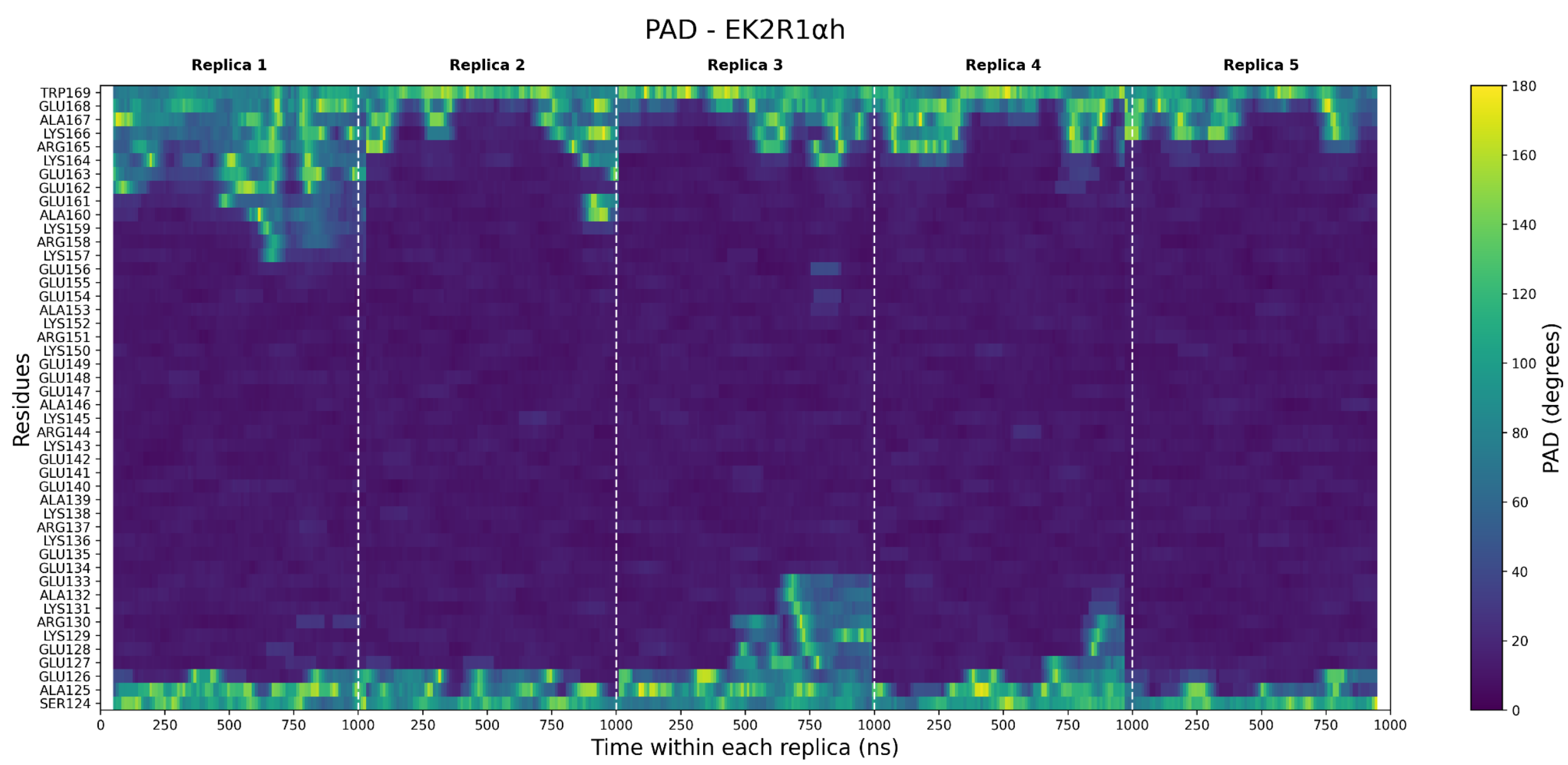

Figure S57. Same as Fig. S56 but for CHARMM+TIP3P-based MD of **EK2R1αh**.

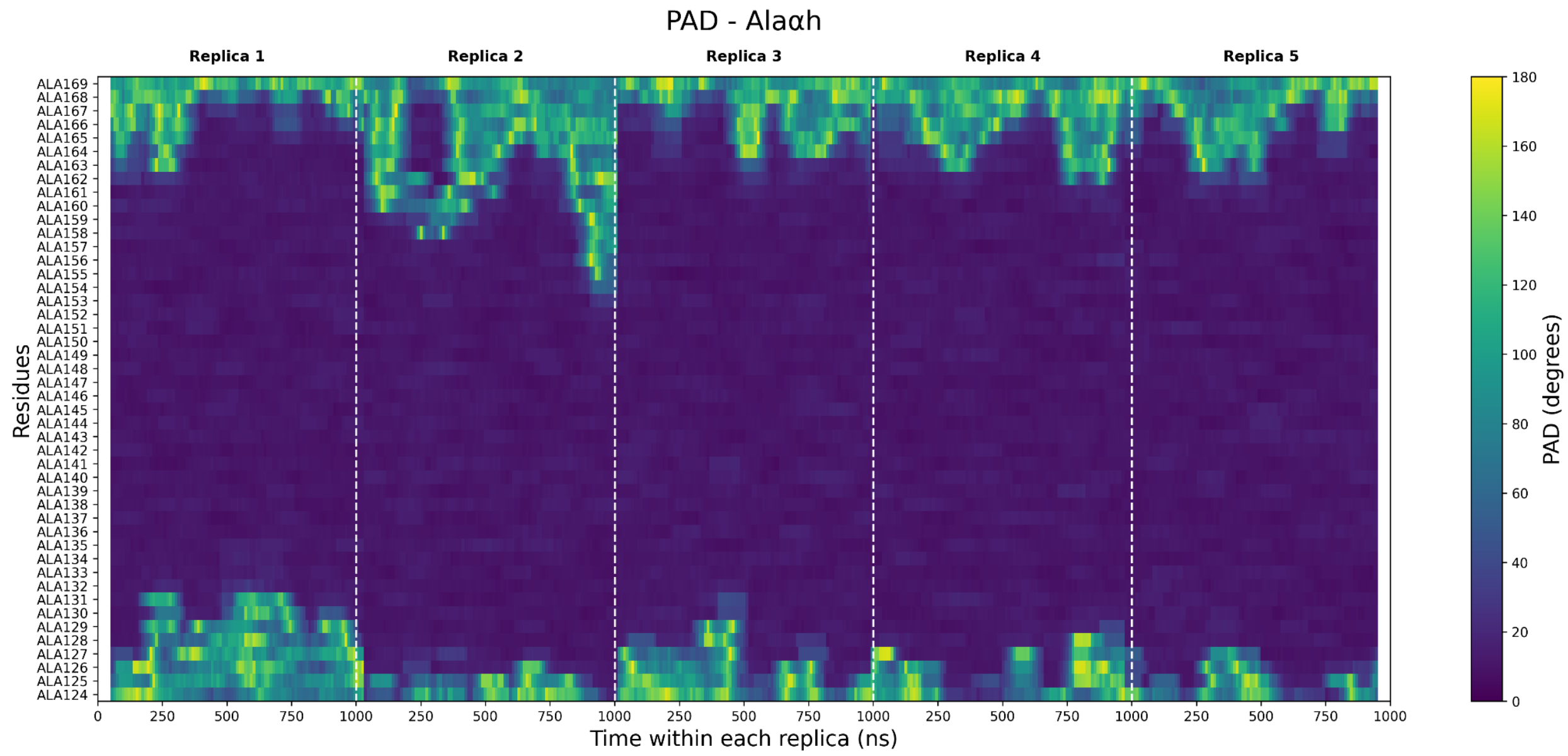

Figure S58. Same as Fig. S56 but for CHARMM+TIP3P-based MD of **Alaαh**.

5.4. SASA analysis

The SASA values of the three systems are similar to those obtained using Amber14SB+TIP3P-based MD. As expected, the regions with low α-helical content and increased bending and flexibility have high average SASA values and fluctuations and vice versa (Figs. S59-S62).

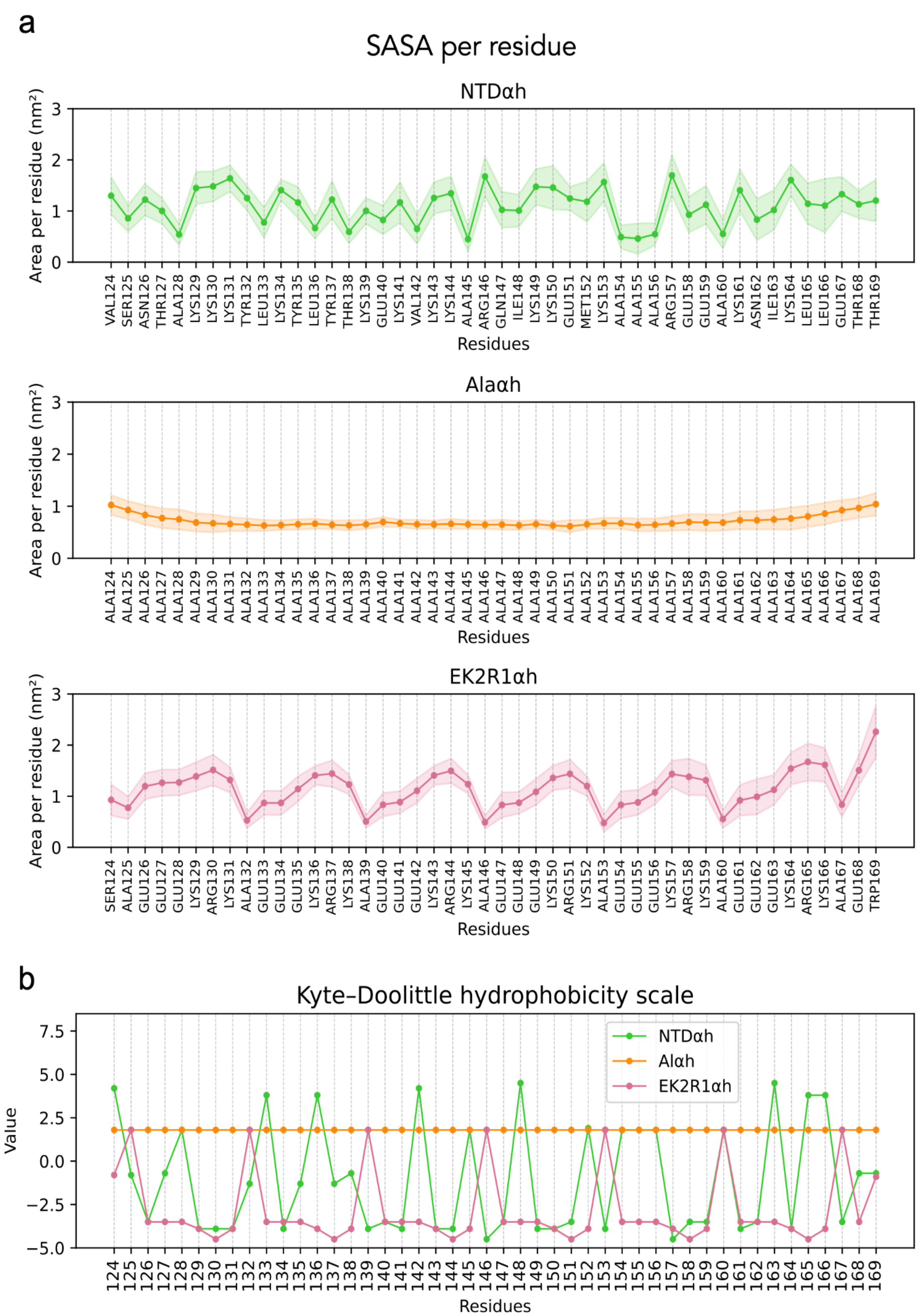

Figure S59. a Average SASA (with its standard deviation) per residue from CHARMM+TIP3P - based MD of **NTDαh**, **Alaαh** and **EK2R1αh**. **b** Corresponding hydrophobicity of each residue based on the Kyte-Doolittle scale[8].

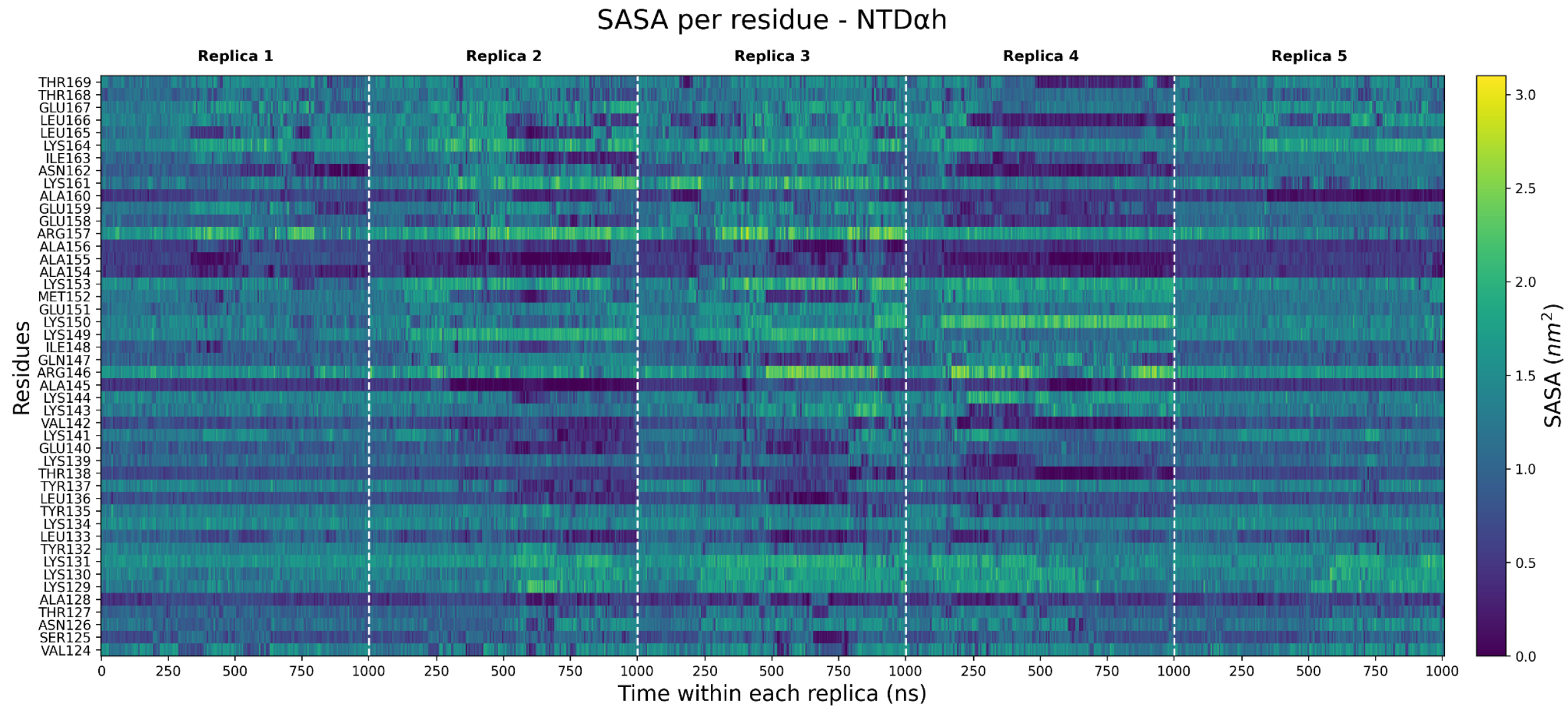

Figure S60. SASA of each residue in the CHARMM+TIP3P -based MD of **NTDαh** (5 replicas) plotted as a function of simulated time.

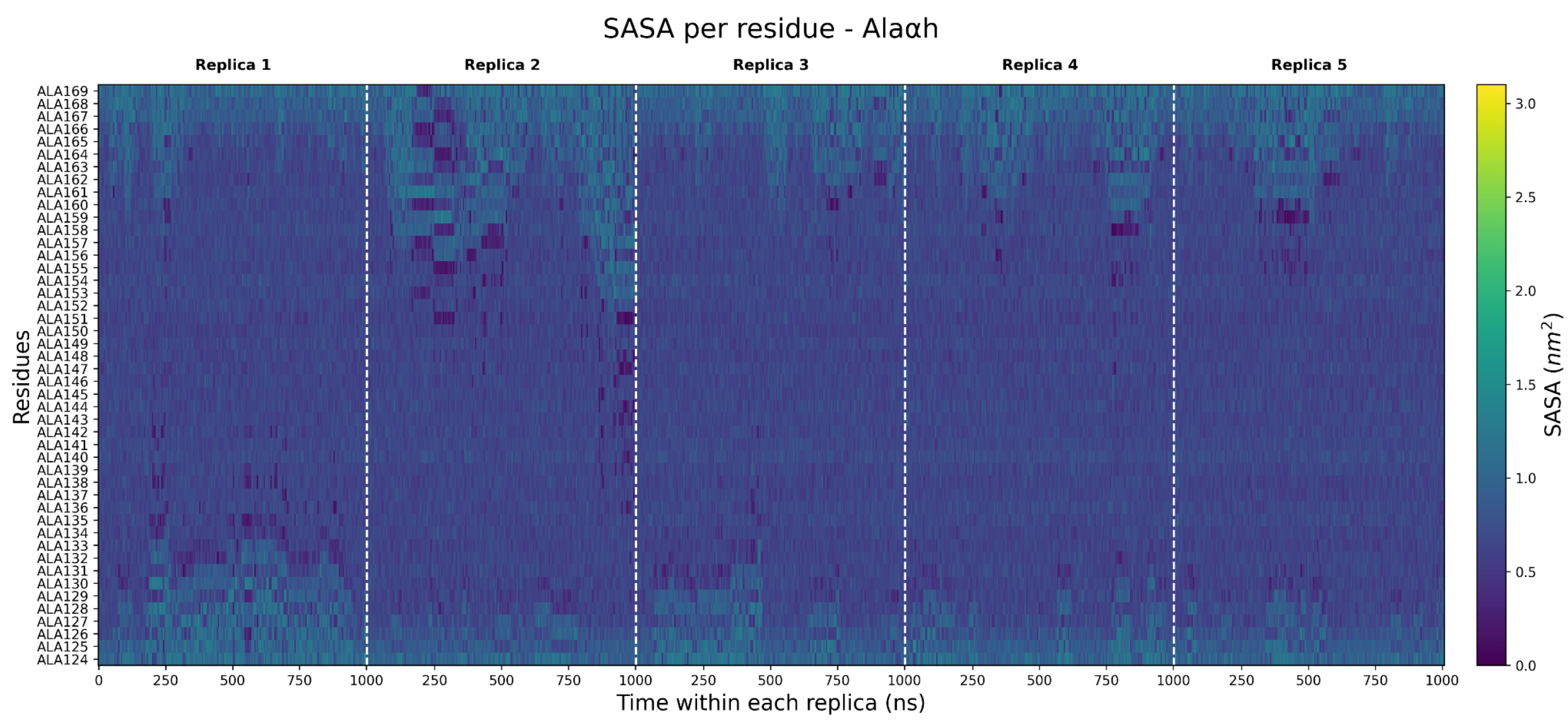

Figure S61. Same as Fig. S60 but for the CHARMM+TIP3P -based MD of **Alaαh**.

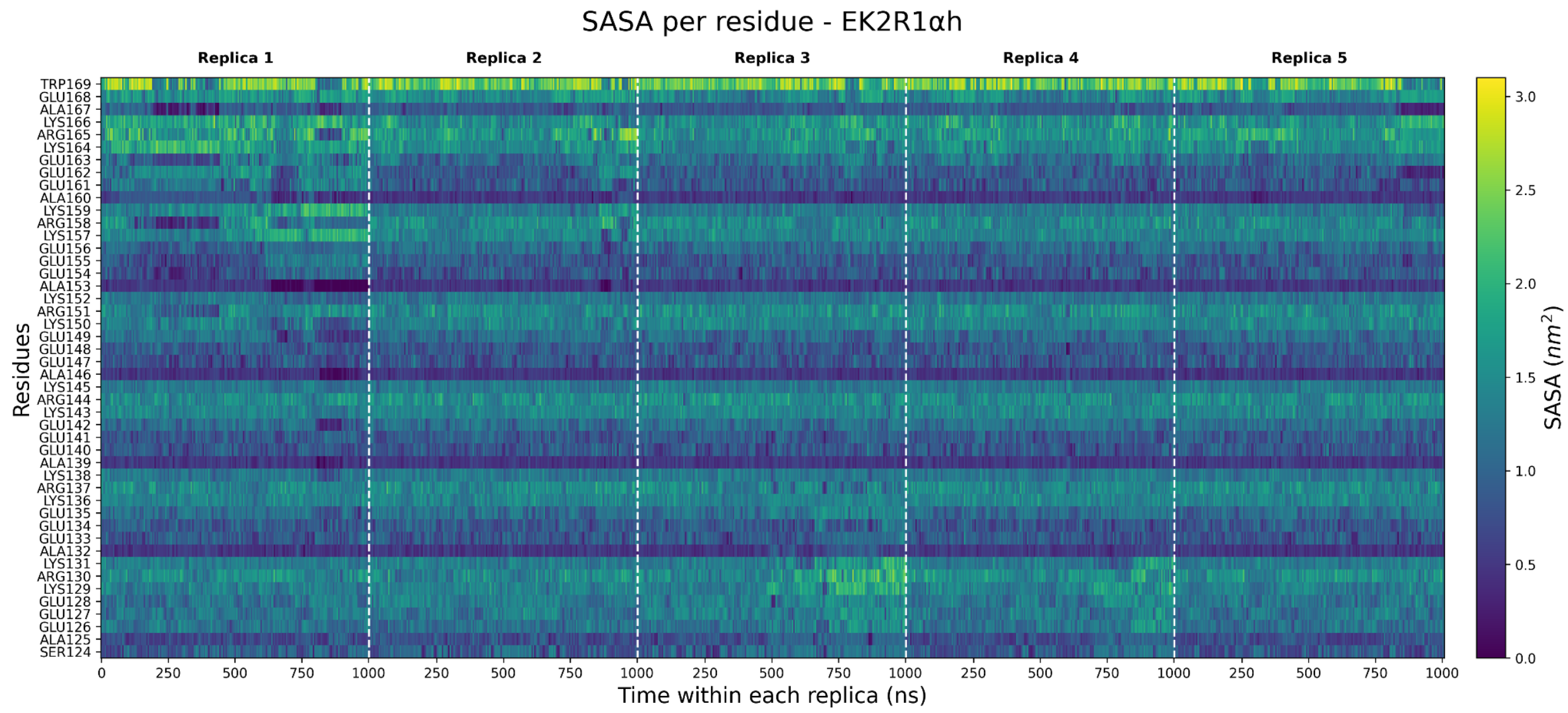

Figure S62. Same as Fig. S60 but for the CHARMM+TIP3P -based MD of **EK2R1αh**.

5.5. Clustering analysis

In the CHARMM+TIP3P-based MD (Tabs S9-S11 and Figs S63-S65), **NTDαh** still exhibits intermediate conformational diversity with 8 dominant clusters accounting for ~55% of the trajectory and **EK2R1αh** remains as a rigid system with a single dominant cluster accounting for ~68% of the trajectory. However, **Alaαh** features a dominant conformation.

Cluster	Population
1	20.9%
2	10.3%
3	5.5%
4	3.9%
5	3.9%
6	3.7%
7	3.7%
8	3.1%
Other (198 clusters)	45.0%

Table S9. Cluster populations from the CHARMM+TIP3P -based MD of **NTDαh**.

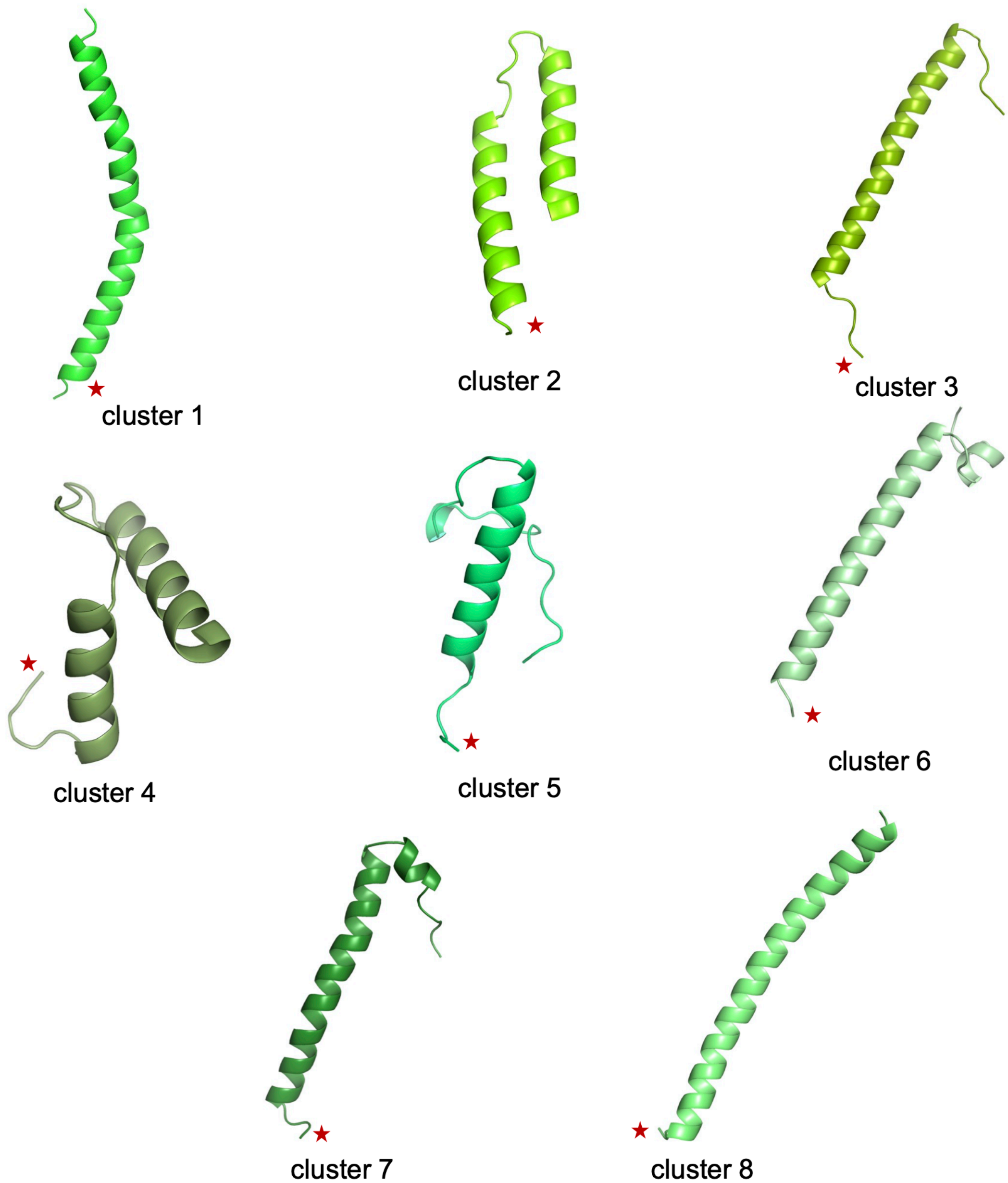

Figure S63. Representative structure of each cluster from the CHARMM+TIP3P -based MD of **NTDαh**. The red star highlights the N-terminus.

Cluster	Population
1	67.4%
2	8.0%
3	7.1%
4	3.8%
5	2.8%
Other (38 clusters)	10.9%

Table S10. Same as Tab. S9 but for the CHARMM+TIP3P-based MD of **Alaαh.**

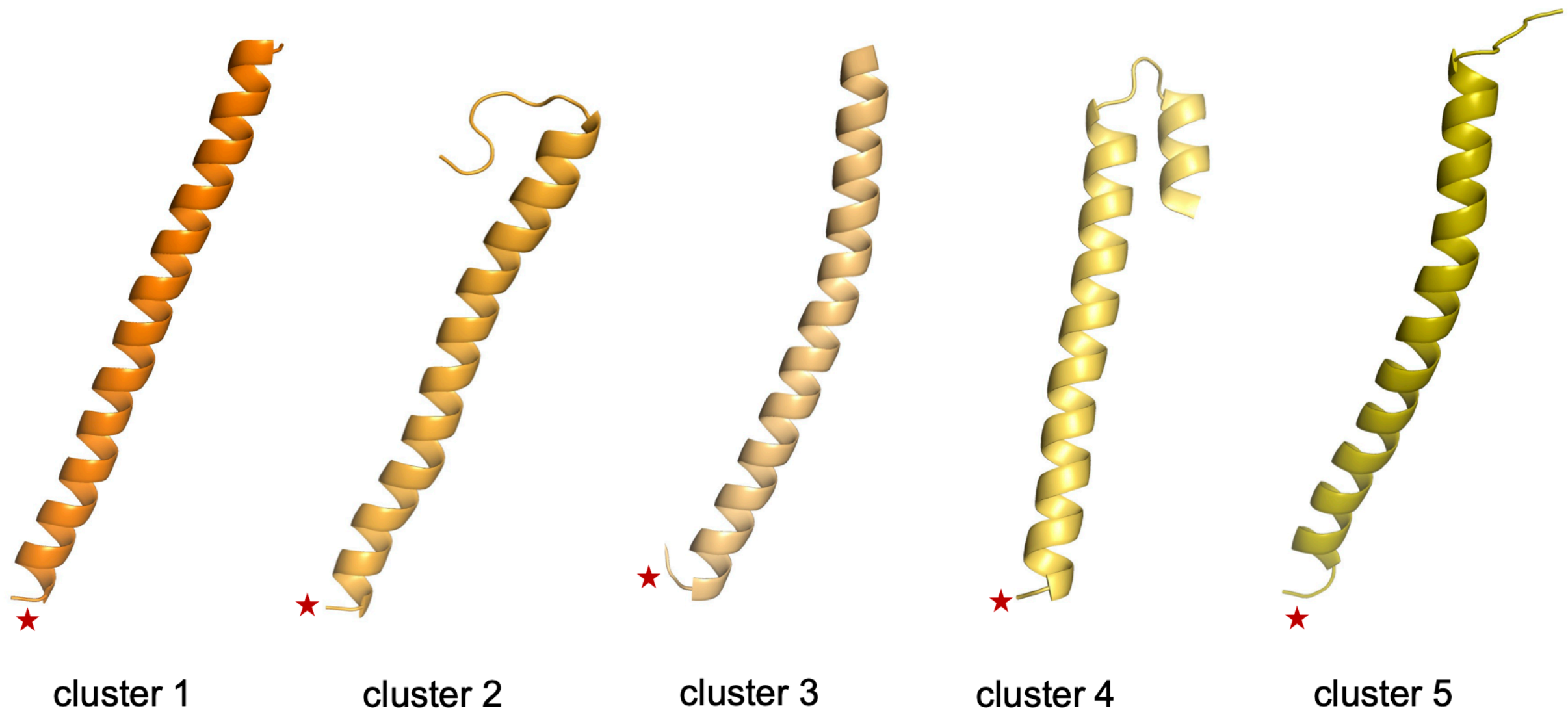

Figure S64. Same as Fig. S63 but for the CHARMM+TIP3P-based MD of **Alaαh.**

Cluster	Population
1	68.3%
2	8.5%
3	4.1%
4	3.8%
5	2.8%
Other (31 clusters)	12.5%

Table S11. Same as Tab. S9 but for the CHARMM+TIP3P-based MD of **EK2R1αh.**

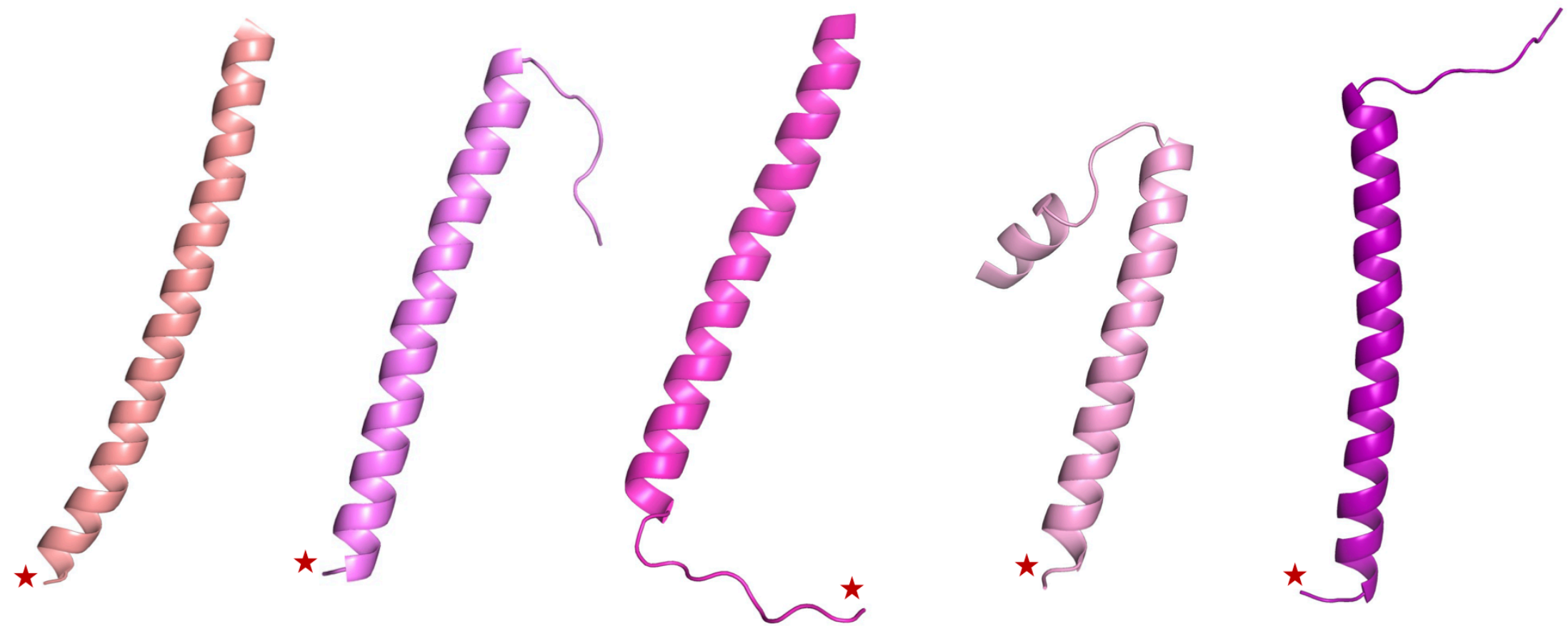

Figure S65. Same as Fig. S63 but for the CHARMM+TIP3P-based MD of **EK2R1αh.**

5.6. PCA analysis

In the CHARMM+TIP3P-based MD (Figs.S66-S71), the free energy landscape of **EK2R1αh** presents one basin with a straight helix as representative and **NTDαh** shows two basins with different conformations. However, **Alaαh** exhibits only one basin, indicating that it is a stable helix like **EK2R1αh**.

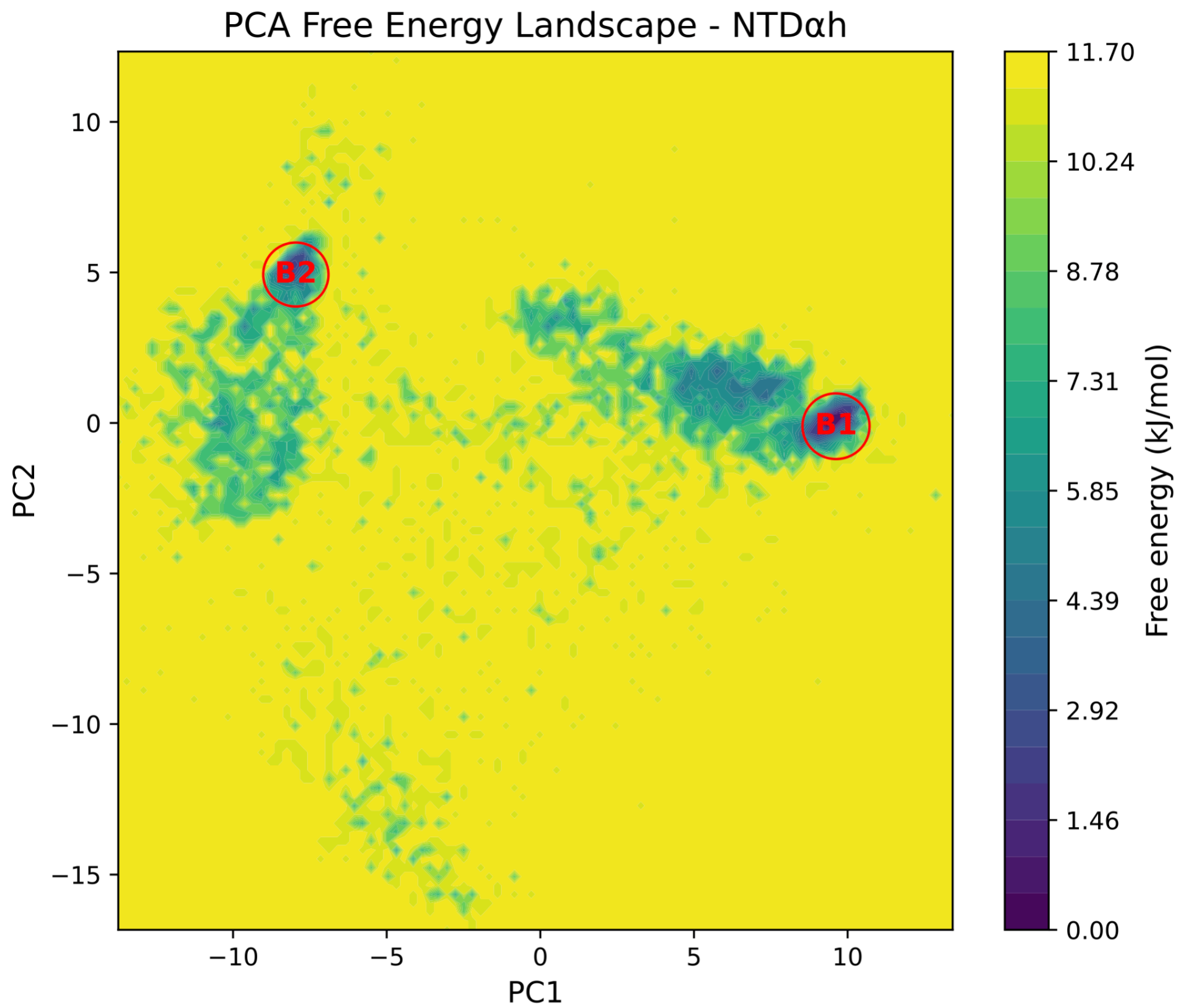

Figure S66. PCA relative free energy landscape from the CHARMM+TIP3P -based MD of **NTDαh**. The minimum is set to zero. The free energy is plotted as a function of the first two principal components (PC1 and PC2). The basins B1-B2 is marked with the red circles.

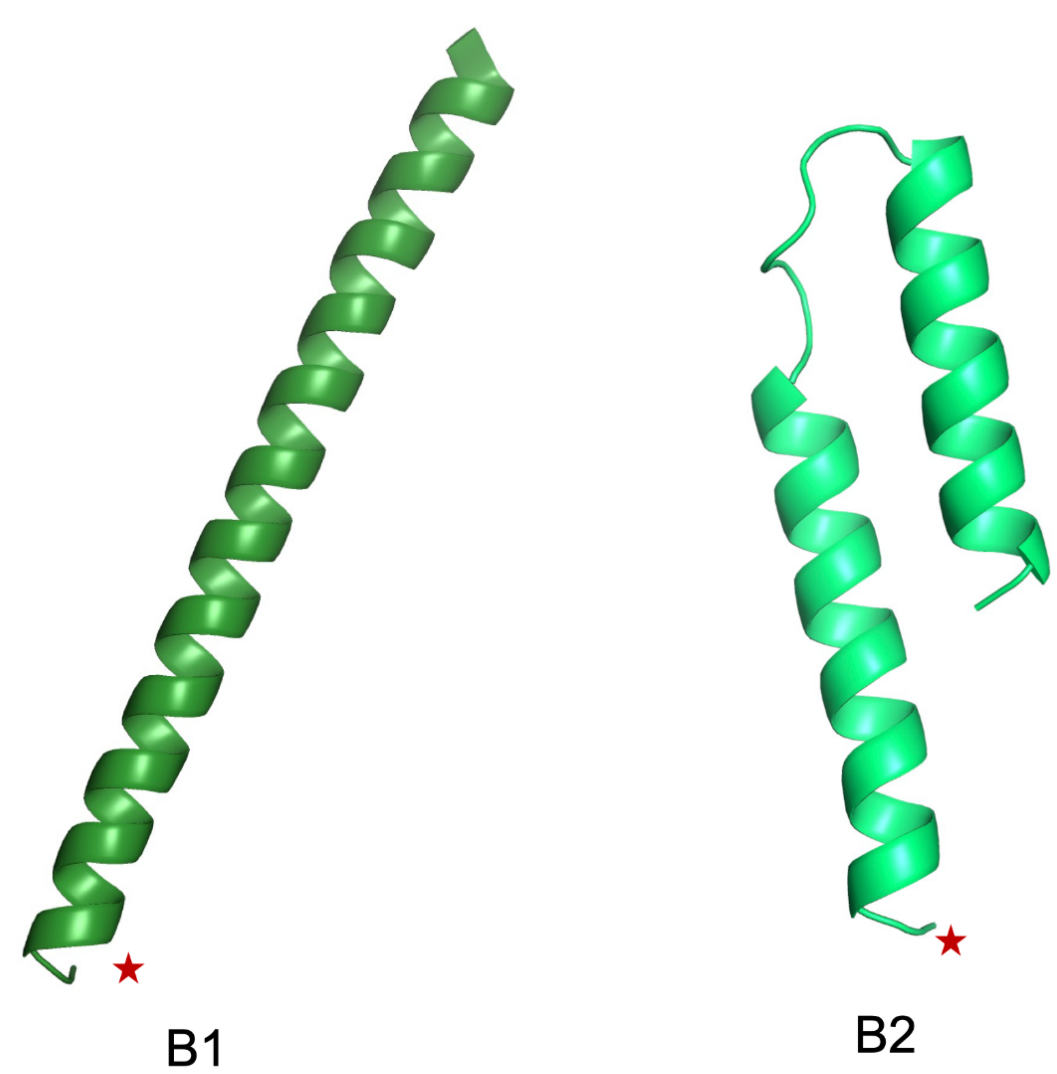

Figure S67. Representative structures from a clustering analysis of the basins 1-2 in Fig. S66. The red star highlights the N-terminus.

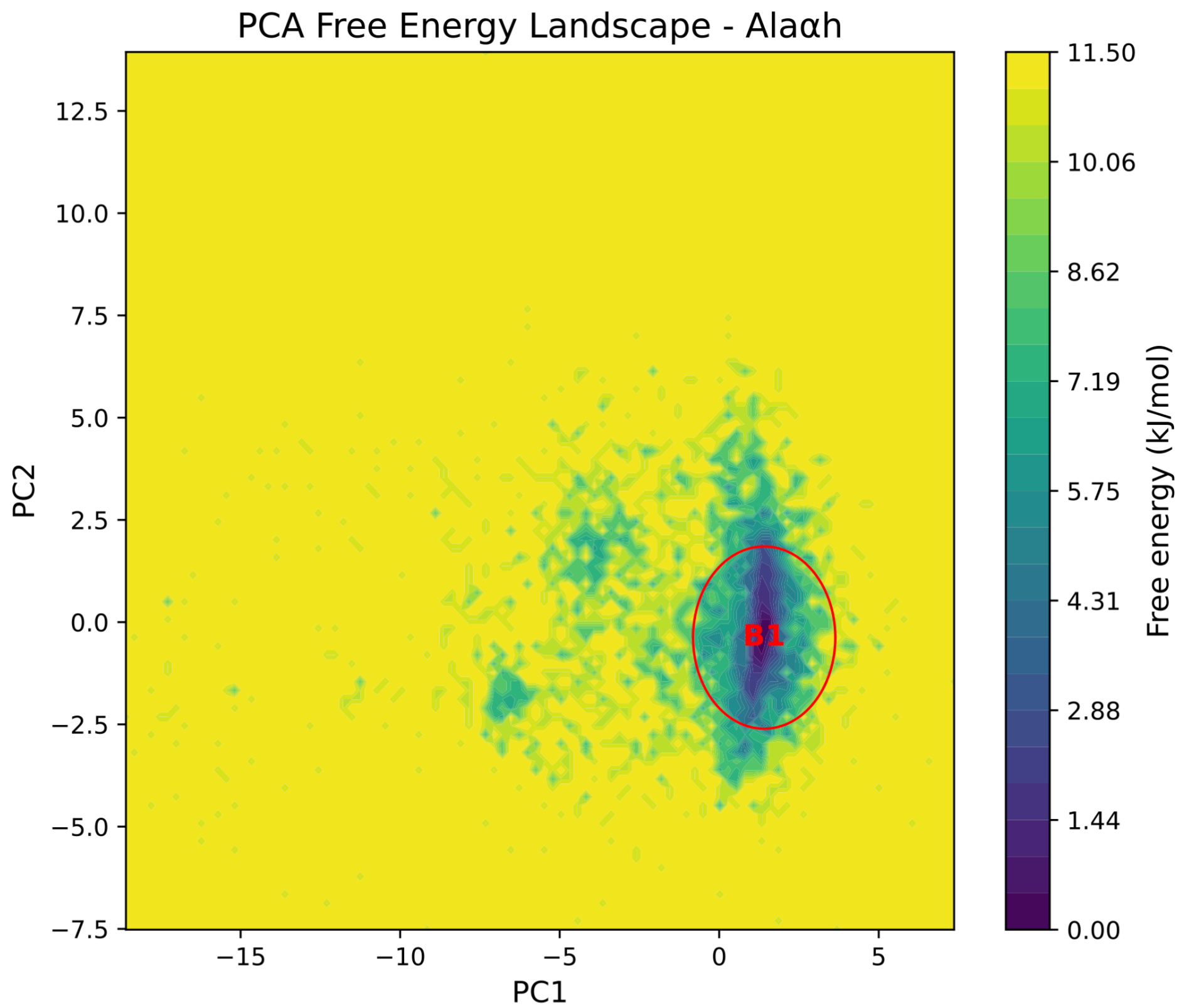

Figure S68. Same as Fig. S66 but for the CHARMM+TIP3P-based MD of **Alaαh**.

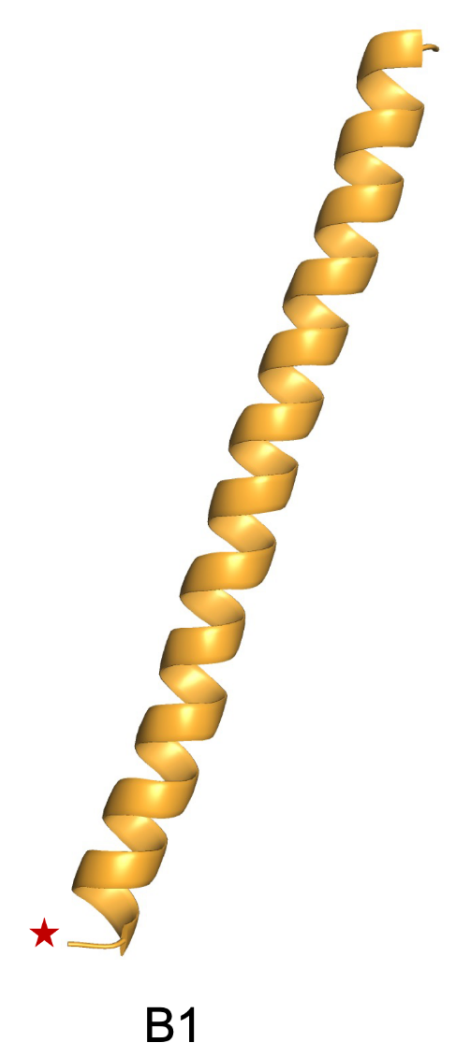

Figure S69. Representative structures from a clustering analysis of the basin 1 in Fig. S68. The red star highlights the N-terminus.

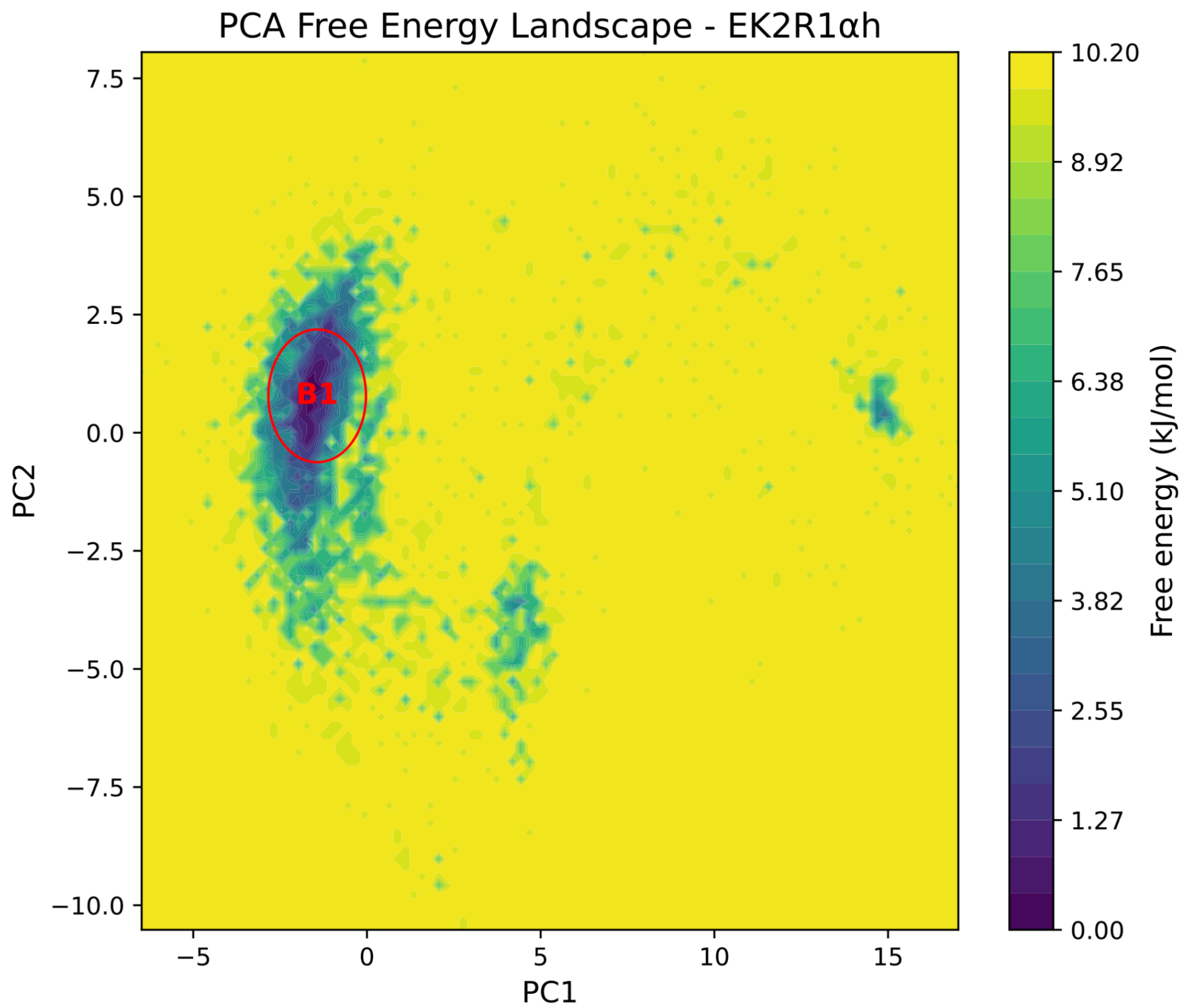

Figure S70. Same as Fig. S66 but for the CHARMM+TIP3P-based MD of **EK2R1αh.**

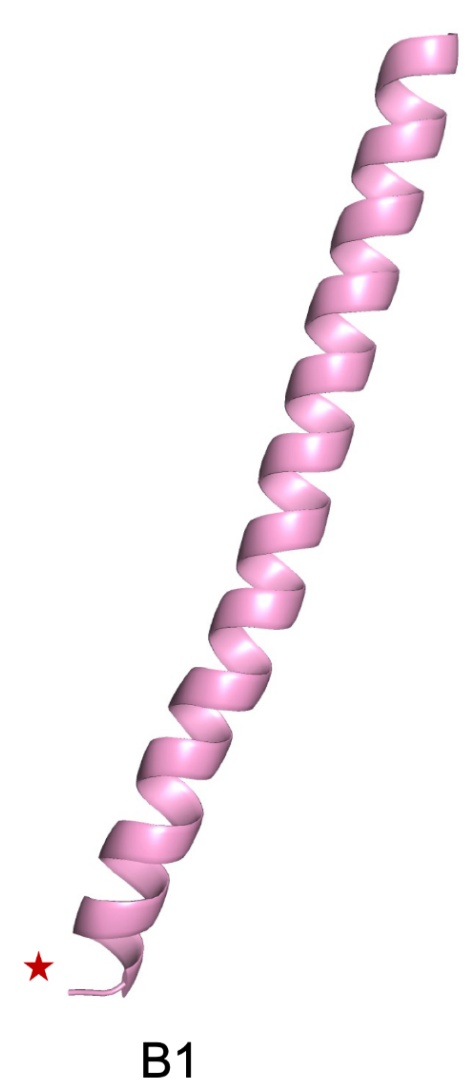

Figure S71. Representative structures from a clustering analysis of the basin 1 in Fig. S70. The red star highlights the N-terminus.

6. NTDαh helices from the Trm10 family using Amber14SB+TIP3P-based MD

6.1 Secondary structure analysis

We compared the secondary structure results of NTDαh in human TRMT10C (referred to as **NTDαh**) with three other NTDαh helices from the Trm10 family (human TRMT10A, human TRMT10B and *Drosophila melanogaster* TRMT10C). The three helices showed not too dissimilar helical distributions from those of **NTDαh** (Figs. S72-S75, Tab. S12). The first two helices feature a higher α-helical content from the first few residues from the N-terminus to the middle of the sequence, and a region of lower helical content from the middle of the sequence to the C-terminus (Fig.72a-c,e-g). *Drosophila melanogaster* TRMT10C NTDαh exhibits instead a higher α-helical content at the middle of the sequence and a lower content close to the termini (Fig.S72d,h). Overall, the α-helical composition in TRMT10C (~80%) is not too dissimilar from that in TRMT10A and TRMT10B (60-70%) (Tab.S12).

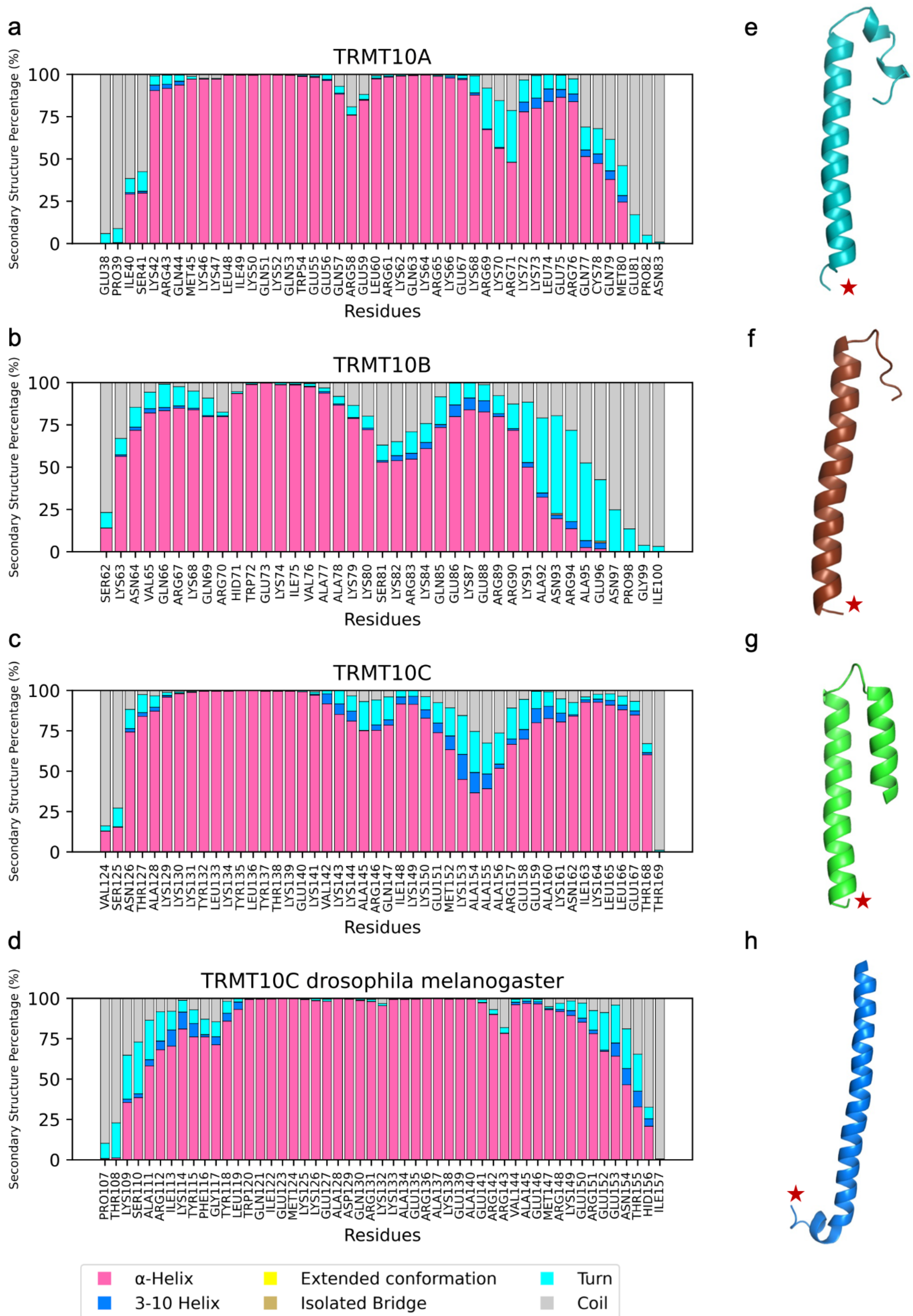

a
TRMT10A
Secondary Structure Percentage (%)
100
75
50
25
0
GLU38 PRO39 ILE40 SER41 LYS42 ARG43 GLN44 LYS45 MET45 LYS46 LYS47 LEU48 ILE49 LYS50 GLN51 LYS52 GLN53 TRP54 GLU55 GLU56 GLN57 ARG58 GLU59 LEU60 ARG61 LYS62 GLN63 LYS64 ARG65 LYS66 GLU67 LYS68 ARG69 LYS70 ARG71 LYS72 LYS73 LEU74 GLU75 ARG76 GLN77 CYS78 GLN79 MET80 GLU81 PRO82 ASN83
Residues
b
TRMT10B
Secondary Structure Percentage (%)
100
75
50
25
0
SER62 LYS63 ASN64 VAL65 GLN66 ARG67 LYS68 GLN69 ARG70 HID71 TRP72 GLU73 LYS74 ILE75 VAL76 ALA77 ALA78 LYS79 LYS80 SER81 LYS82 ARG83 LYS84 GLN85 GLU86 LYS87 GLU88 ARG89 ARG90 LYS91 ALA92 ASN93 ARG94 ALA95 GLU96 ASN97 PRO98 GLY99 ILE100
Residues
c
TRMT10C
Secondary Structure Percentage (%)
100
75
50
25
0
VAL124 SER125 ASN126 THR127 ALA128 LYS129 LYS130 LYS131 TYR132 LEU133 LYS134 TYR135 LEU136 TYR137 THR138 LYS139 GLU140 LYS141 VAL142 LYS143 LYS144 ALA145 ARG146 GLN147 ILE148 LYS149 LYS150 GLU151 MET152 LYS153 ALA154 ALA155 ALA156 ARG157 GLU158 GLU159 ALA160 LYS161 ASN162 ILE163 LYS164 LEU165 LEU166 GLU167 THR168 THR169
Residues
d
TRMT10C drosophila melanogaster
Secondary Structure Percentage (%)
100
75
50
25
0
PRO107 THR108 LYS109 SER110 ALA111 ARG112 ILE113 LYS114 TYR115 PHE116 GLY117 TYR118 LEU119 TRP120 GLN121 ILE122 GLU123 MET124 LYS125 LYS126 GLU127 ALA128 ASP129 GLN130 ARG131 LYS132 LYS133 ALA134 GLU135 ARG136 ALA137 LYS138 GLU139 ALA140 GLU141 ARG142 ARG143 VAL144 ALA145 GLU146 MET147 ARG148 LYS149 GLU150 ARG151 GLU152 GLU153 ASN154 THR155 HID156 ILE157
Residues
α-Helix
3-10 Helix
Extended conformation
Isolated Bridge
Turn
Coil
e
f
g
h

Figure S72. Amber14SB+TIP3P -based MD of TRMT10B NTDαh of TRMT10A/TRMT10B/TRMT10C and *drosophila melanogaster* TRMT10C (indicated in the figure). **a-d** Secondary structure composition per residue. **e-h** Corresponding representative structures, defined as the central structure of the largest cluster obtained from cluster analysis in GROMACS. The red star highlights the N-terminus.

	TRMT10A human	TRMT10B human	TRMT10C human	TRMT10C *drosophila melanogaster*
α-Helix	73.65 (7.93) %	60.72(14.06) %	78.18 (9.26) %	79.65 (7.70) %
3-10 Helix	1.45 (3.38) %	1.82 (4.02) %	3.25 (4.73) %	2.16 (3.59) %
Extended conformation	0.00 (0.00) %	0.00 (0.00) %	0.00 (0.00) %	0.00 (0.00) %
Isolated Bridge	0.00 (0.14) %	0.07 (0.59) %	0.00 (0.00) %	0.00 (0.00) %
Turn	6.68 (7.67) %	14.02 (11.49) %	7.68 (6.96) %	6.77 (6.43) %
Coil	18.22 (4.76) %	23.37 (8.80) %	10.89 (5.02) %	11.42(4.64)%

Table S12. Secondary structure composition over the cumulative trajectory of Amber14SB+TIP3P -based MD of human TRMT10A/TRMT10B/TRMT10C and *drosophila melanogaster* TRMT10C. The average and standard deviation (in parentheses) of each composition across the trajectory are shown.

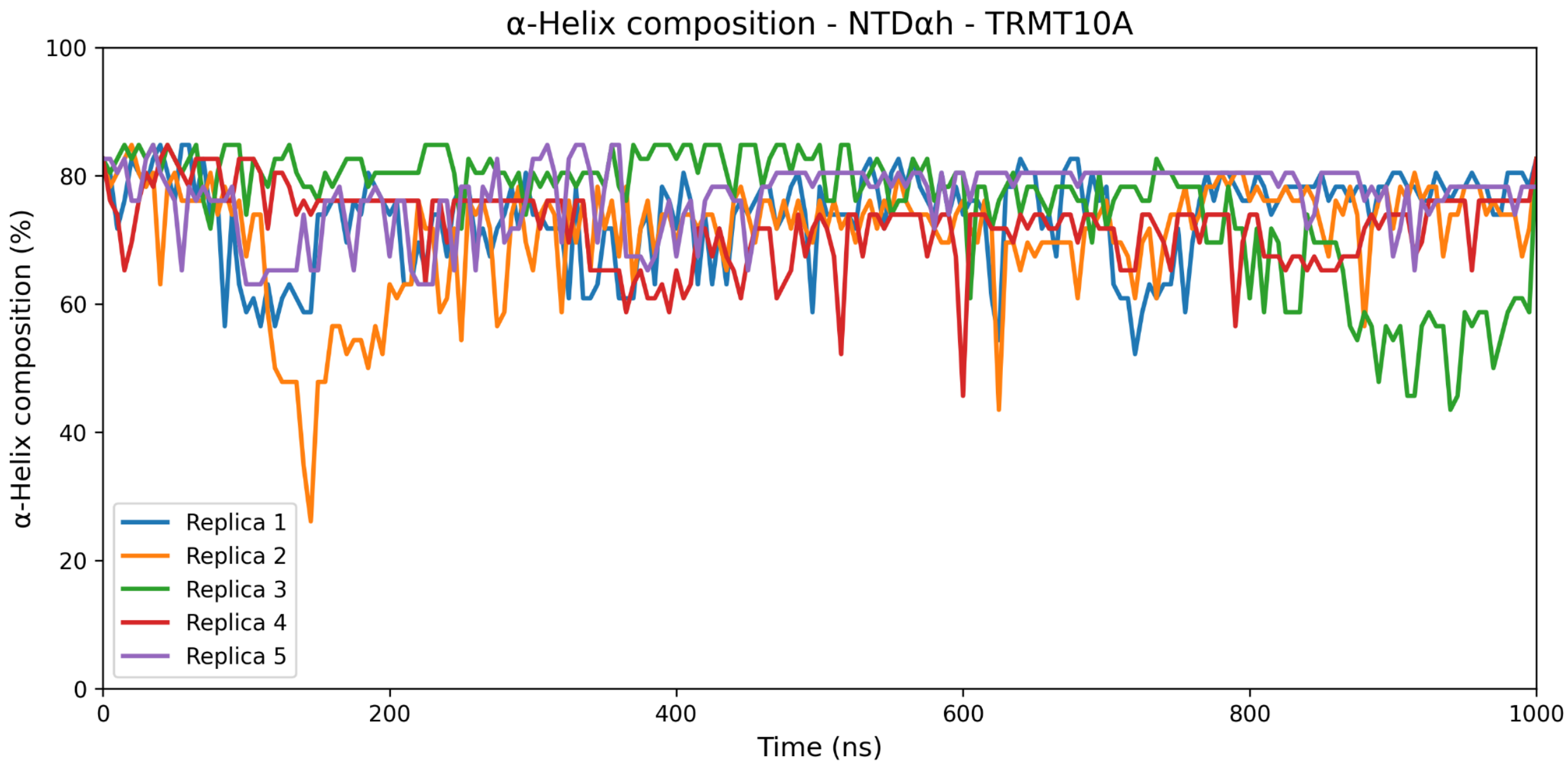

Figure S73. α-helix content of human TRMT10A NTDαh in the Amber14SB+TIP3P-based MD (5 replicas), plotted as a function of simulated time.

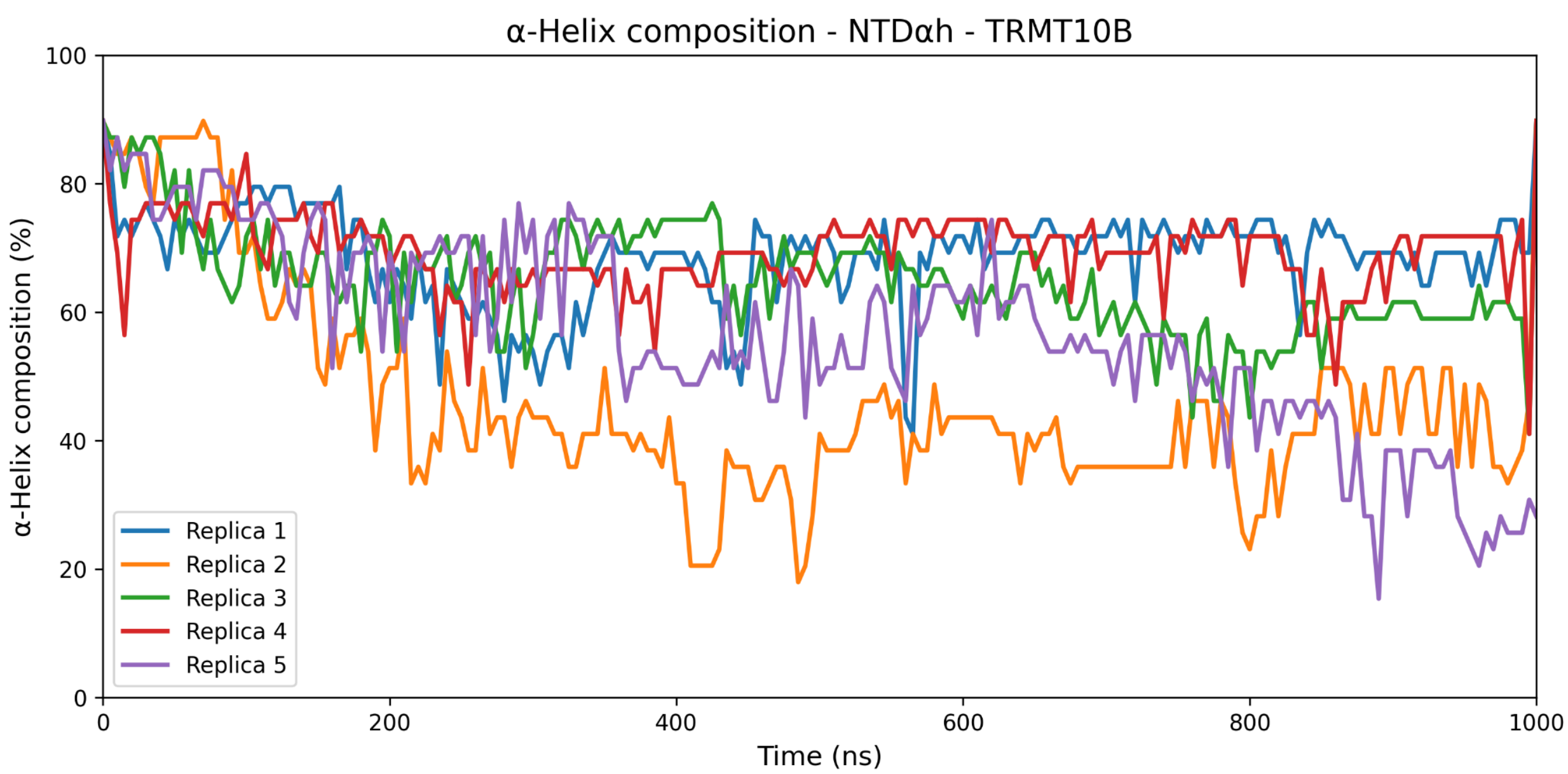

Figure S74. Same as Fig. S73 but for Amber14SB+TIP3P-based MD of human TRMT10B NTDαh.

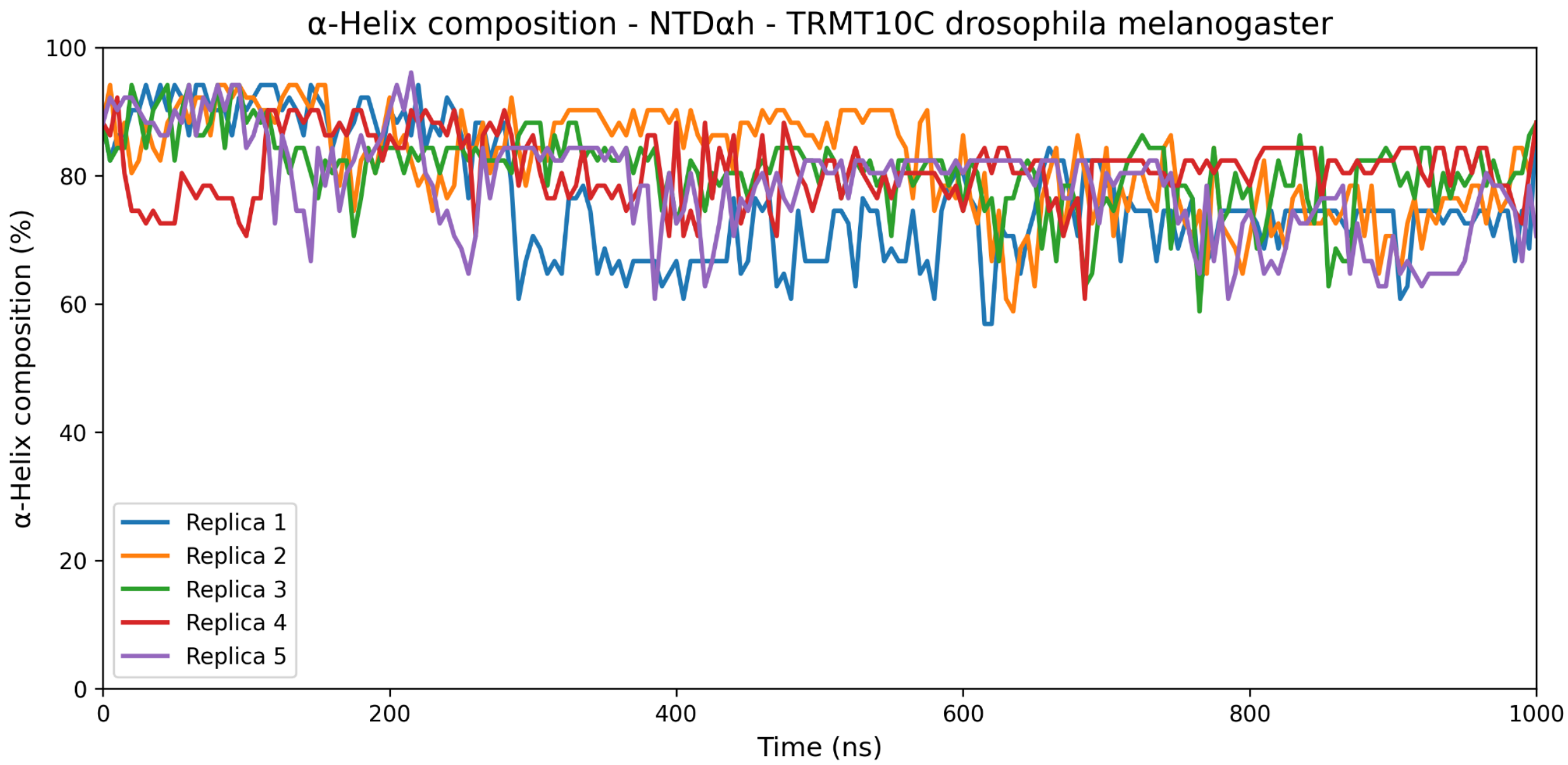

Figure S75. Same as Fig. S73 but for Amber14SB+TIP3P-based MD of *drosophila melanogaster* TRMT10C NTDαh.

6.2. Bending angles

The helices share not too dissimilar bending angle distributions and fluctuations (Figs.S76-S79) from those of **NTDαh**.

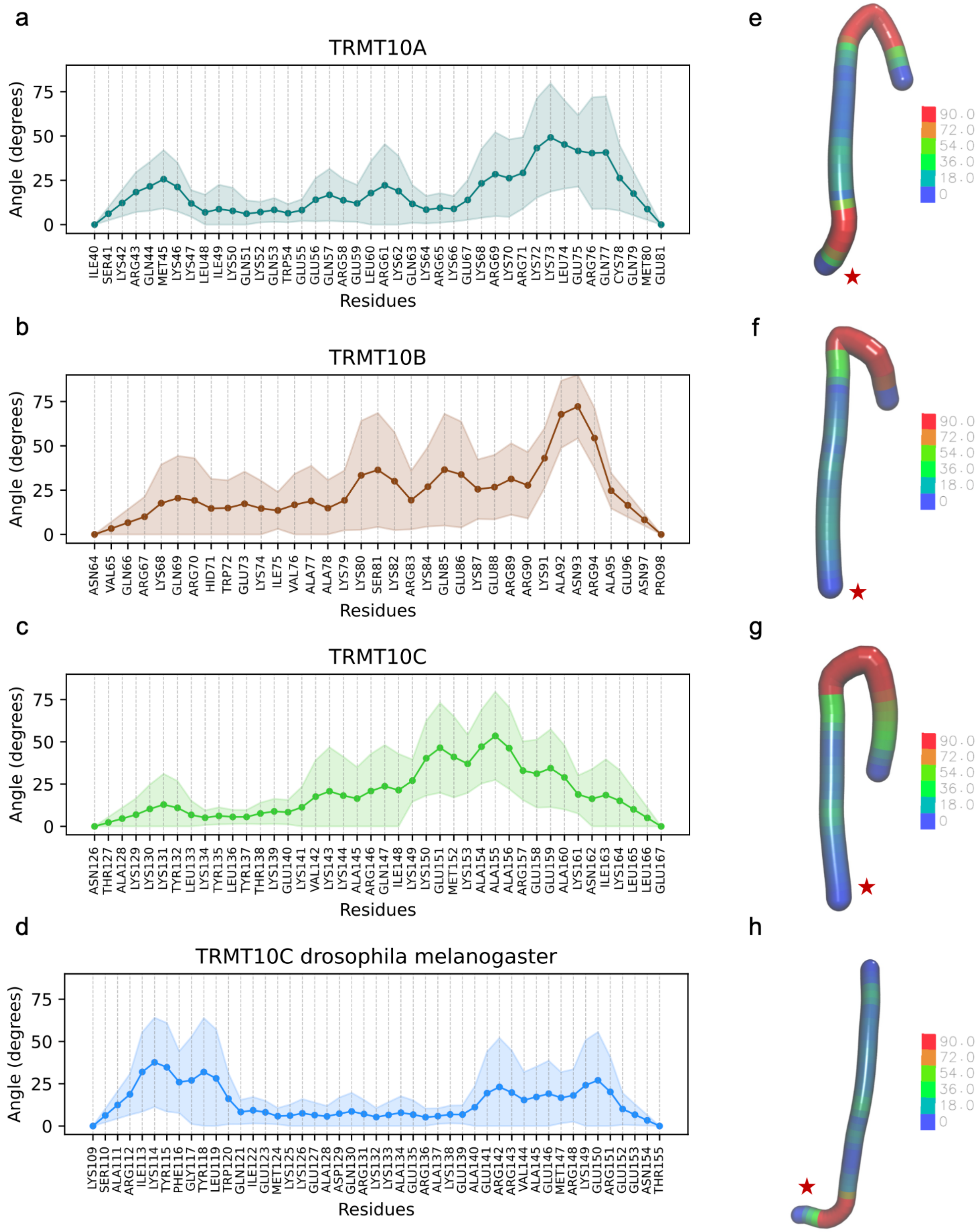
a
TRMT10A
Angle (degrees)
0
25
50
75
ILE40 SER41 LYS42 ARG43 GLN44 MET45 LYS46 LYS47 LEU48 ILE49 LYS50 GLN51 LYS52 GLN53 TRP54 GLU55 GLU56 GLN57 ARG58 GLU59 LEU60 ARG61 LYS62 GLN63 LYS64 ARG65 LYS66 GLU67 LYS68 ARG69 LYS70 ARG71 LYS72 LYS73 LEU74 GLU75 ARG76 GLN77 CYS78 GLN79 MET80 GLU81
Residues
b
TRMT10B
Angle (degrees)
0
25
50
75
ASN64 VAL65 GLN66 ARG67 LYS68 GLN69 ARG70 HID71 TRP72 GLU73 LYS74 ILE75 VAL76 ALA77 ALA78 LYS79 GLN80 SER81 LYS82 ARG83 LYS84 GLN85 GLU86 LYS87 GLU88 ARG89 ARG90 LYS91 ALA92 ASN93 ARG94 ALA95 GLU96 ASN97 PRO98
Residues
c
TRMT10C
Angle (degrees)
0
25
50
75
ASN126 THR127 ALA128 LYS129 LYS130 LYS131 TYR132 LEU133 LYS134 TYR135 LEU136 TYR137 THR138 LYS139 GLU140 LYS141 VAL142 LYS143 LYS144 ALA145 ARG146 GLN147 ILE148 LYS149 LYS150 GLU151 MET152 LYS153 ALA154 ALA155 ALA156 ARG157 GLU158 GLU159 ALA160 LYS161 ASN162 ILE163 LYS164 LEU165 LEU166 GLU167
Residues
d
TRMT10C drosophila melanogaster
Angle (degrees)
0
25
50
75
LYS109 SER110 ALA111 ARG112 ILE113 LYS114 TYR115 PHE116 GLY117 TYR118 LEU119 TRP120 GLN121 ILE122 GLU123 MET124 LYS125 LYS126 GLU127 ALA128 ASP129 GLN130 ARG131 LYS132 LYS133 ALA134 GLU135 ARG136 ALA137 LYS138 GLU139 ALA140 GLU141 ARG142 ARG143 VAL144 ALA145 GLU146 MET147 ARG148 LYS149 GLU150 ARG151 GLU152 GLU153 ASN154 THR155
Residues
e
f
g
h
90.0
72.0
54.0
36.0
18.0
0

Figure S76. Amber14SB+TIP3P -based MD of human TRMT10A/TRMT10B/TRMT10C NTDαh and *drosophila melanogaster* TRMT10C NTDαh helices. **a-d** Average bending angles, along with their standard deviations, along the cumulative trajectories. **e-h** Representative structures of the helices. The red color indicates the areas of highest helix axis angle, green indicates intermediate angle magnitudes and blue indicates angles close to or at 0 degrees. The red star highlights the N-terminus.

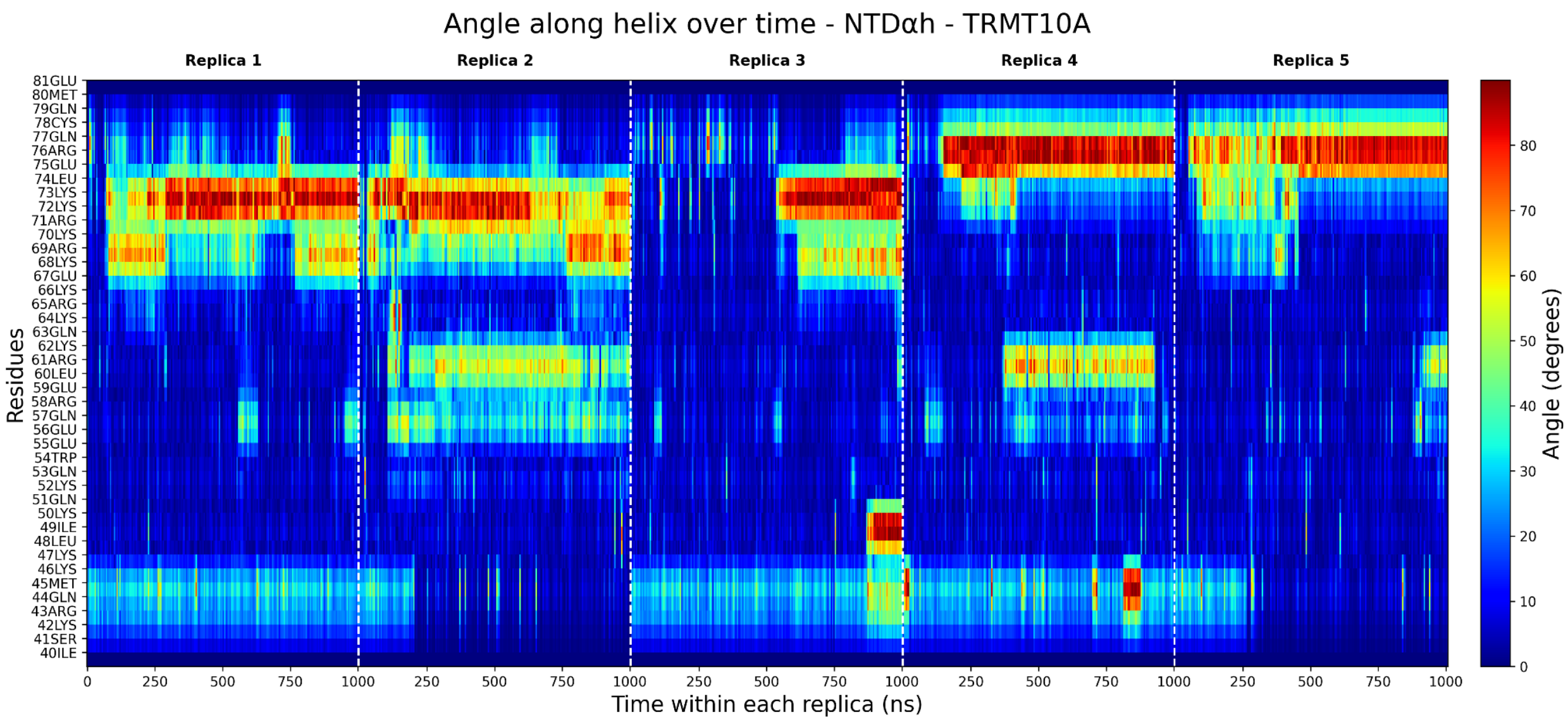

Figure S77. Bending angles for each residue of human TRMT10A NTDαh in the Amber14SB+TIP3P-based MD simulations of 5 replicas, plotted as a function of simulated time.

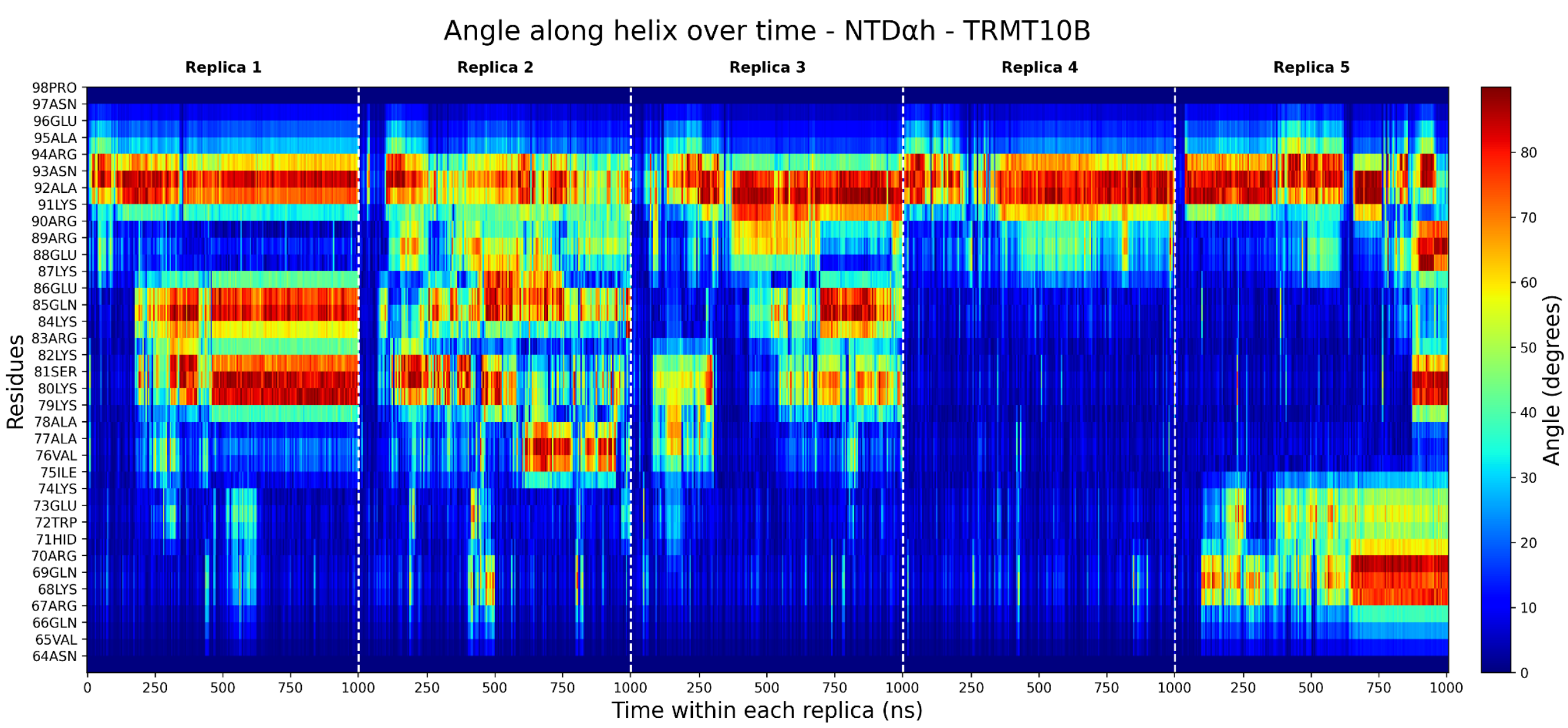

Figure S78. Same as Fig. S77 but for Amber14SB+TIP3P-based MD of human TRMT10B NTDαh.

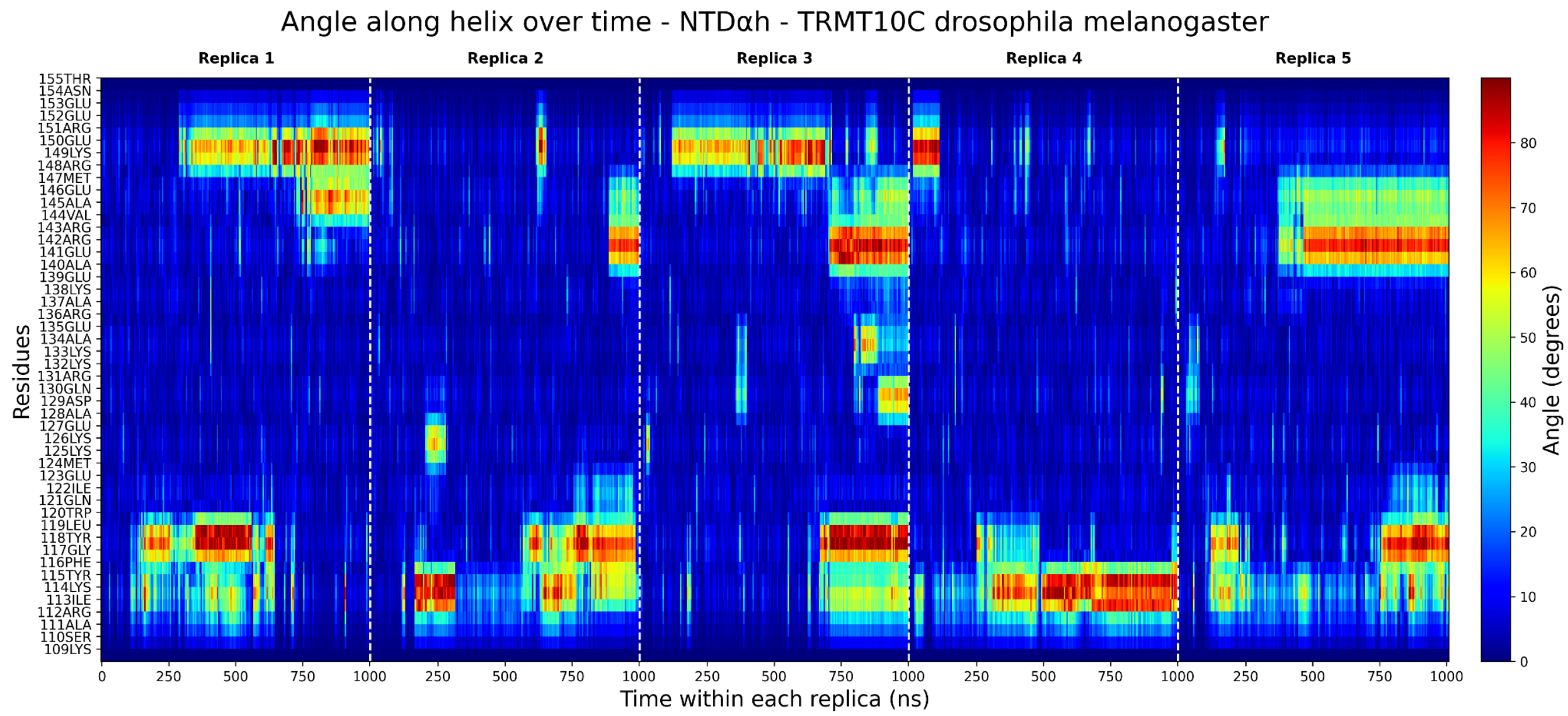

Figure S79. Same as Fig. S77 but for Amber14SB+TIP3P-based MD of *Drosophila melanogaster* TRMT10C NTDαh.

6.3. PAD analysis

In the first two helices (NTDαh of human TRMT10A/TRMT10B) (Figs. S80-S82), the region beyond a few residues from the N-terminal to the middle of the sequence exhibits lower flexibility and the region from the middle of the sequence to the C-terminal has higher flexibility than **NTDαh**. The middle part of *Drosophila melanogaster* TRMT10C NTDαh displays lower flexibility than **NTDαh,** while the termini exhibit larger flexibility (Figs. S80, S83).

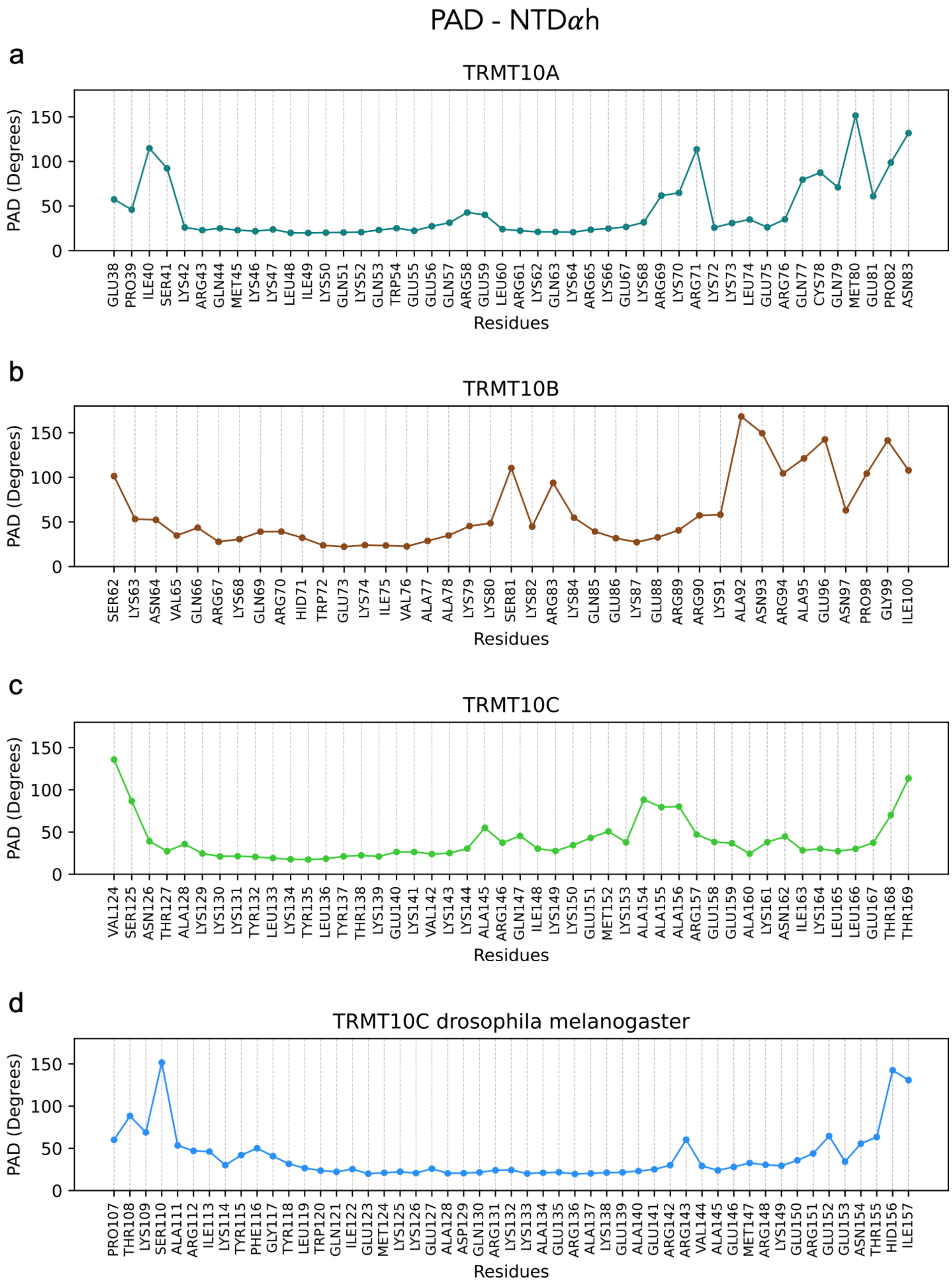
PAD - NTDαh
a
TRMT10A
PAD (Degrees)
0
50
100
150
GLU38 PRO39 ILE40 SER41 LYS42 ARG43 GLN44 MET45 LYS46 LYS47 LEU48 ILE49 LYS50 GLN51 LYS52 GLN53 TRP54 GLU55 GLU56 GLN57 ARG58 GLU59 LEU60 ARG61 LYS62 GLN63 LYS64 ARG65 LYS66 GLU67 LYS68 ARG69 LYS70 ARG71 LYS72 LYS73 LEU74 GLU75 ARG76 GLN77 CYS78 GLN79 MET80 GLU81 PRO82 ASN83
Residues
b
TRMT10B
PAD (Degrees)
0
50
100
150
SER62 LYS63 ASN64 VAL65 GLN66 ARG67 LYS68 GLN69 ARG70 HID71 TRP72 GLU73 LYS74 ILE75 VAL76 ALA77 ALA78 LYS79 LYS80 SER81 LYS82 ARG83 LYS84 GLN85 GLU86 LYS87 GLU88 ARG89 ARG90 LYS91 ALA92 ASN93 ARG94 ALA95 GLU96 ASN97 PRO98 GLY99 ILE100
Residues
c
TRMT10C
PAD (Degrees)
0
50
100
150
VAL124 SER125 ASN126 THR127 ALA128 LYS129 LYS130 LYS131 TYR132 LEU133 LYS134 TYR135 LEU136 TYR137 THR138 LYS139 GLU140 LYS141 VAL142 LYS143 LYS144 ALA145 ARG146 GLN147 ILE148 LYS149 LYS150 GLU151 MET152 LYS153 ALA154 ALA155 ALA156 ARG157 GLU158 GLU159 ALA160 LYS161 ASN162 ILE163 LYS164 LEU165 LEU166 GLU167 THR168 THR169
Residues
d
TRMT10C drosophila melanogaster
PAD (Degrees)
0
50
100
150
PRO107 THR108 LYS109 SER110 ALA111 ARG112 ILE113 LYS114 TYR115 PHE116 GLY117 TYR118 LEU119 TRP120 GLN121 ILE122 GLU123 MET124 LYS125 LYS126 GLU127 ALA128 ASP129 GLN130 ARG131 LYS132 LYS133 ALA134 GLU135 ARG136 ALA137 LYS138 GLU139 ALA140 GLU141 ARG142 ARG143 VAL144 ALA145 GLU146 MET147 ARG148 LYS149 GLU150 ARG151 GLU152 GLU153 ASN154 THR155 HID156 ILE157
Residues

Figure S80. PAD values calculated for each residue from Amber14SB+TIP3P -based MD of human TRMT10A/TRMT10B/TRMT10C and *drosophila melanogaster* TRMT10C NTDαh.

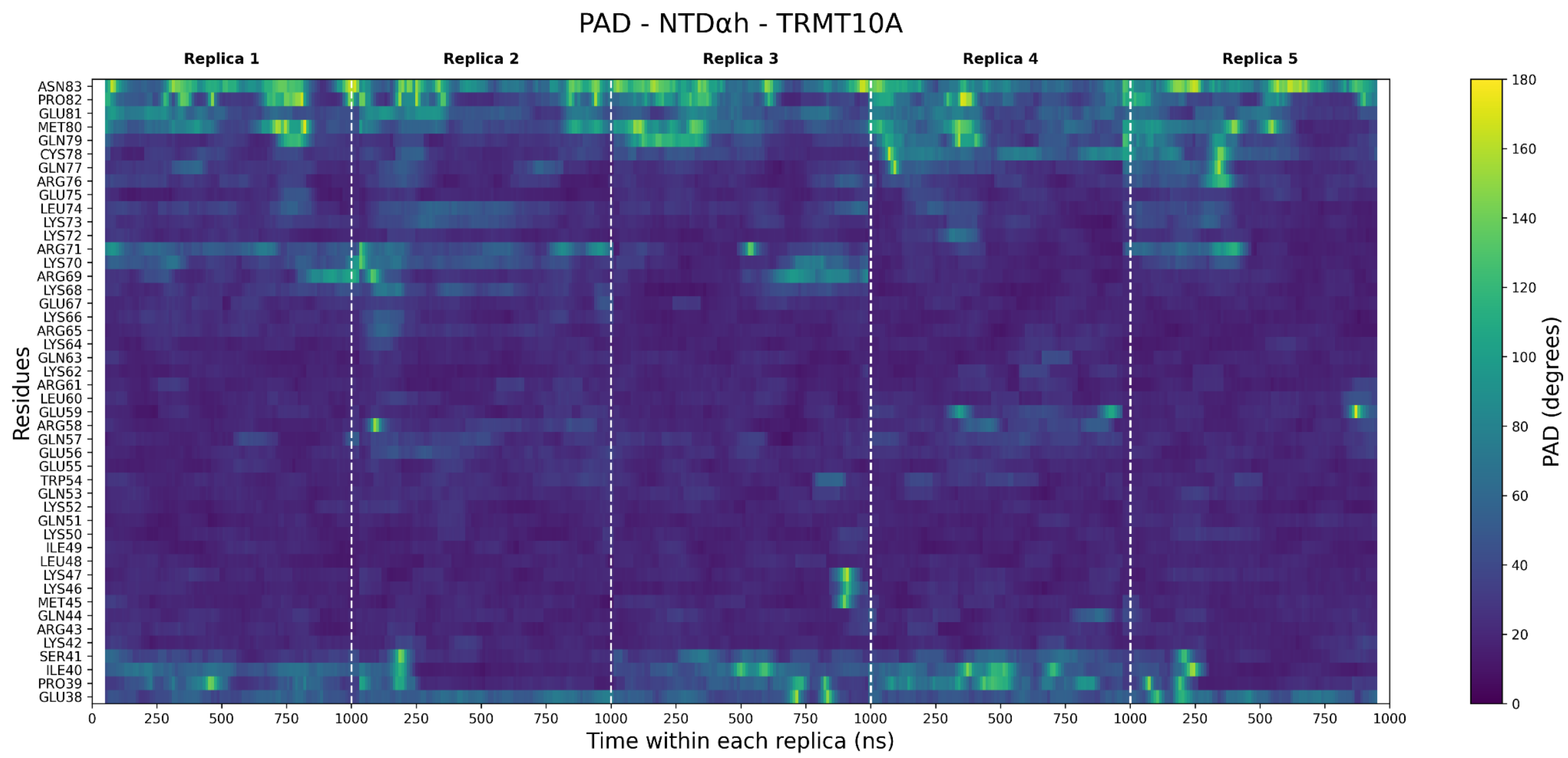

Figure S81. PAD value of each residue in the Amber14SB+TIP3P-based MD of human TRMT10A NTDαh, plotted as a function of simulated time (5 replicas).

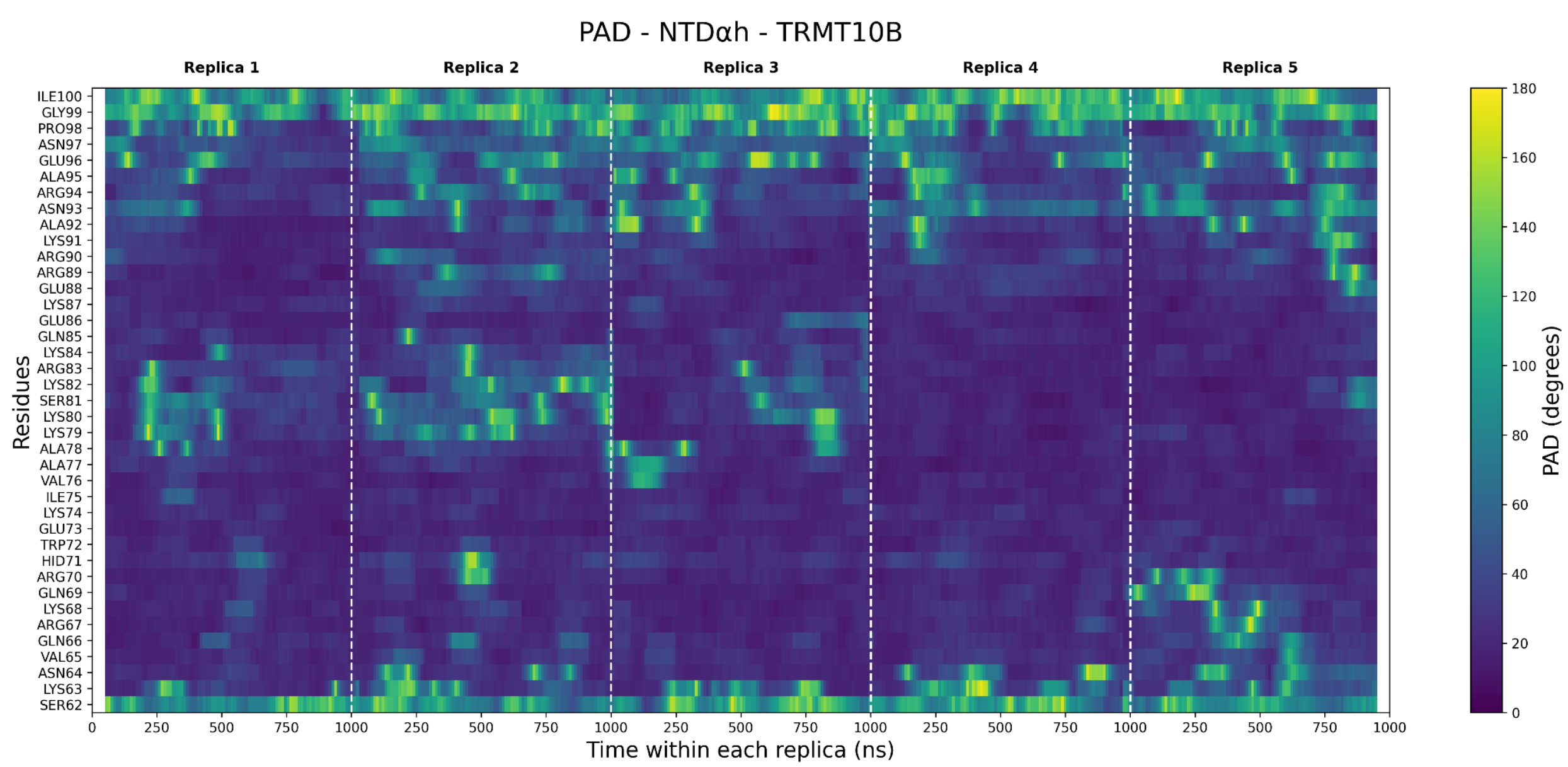

Figure S82. Same as Fig. S81 but for Amber14SB+TIP3P-based MD of human TRMT10B NTDαh.

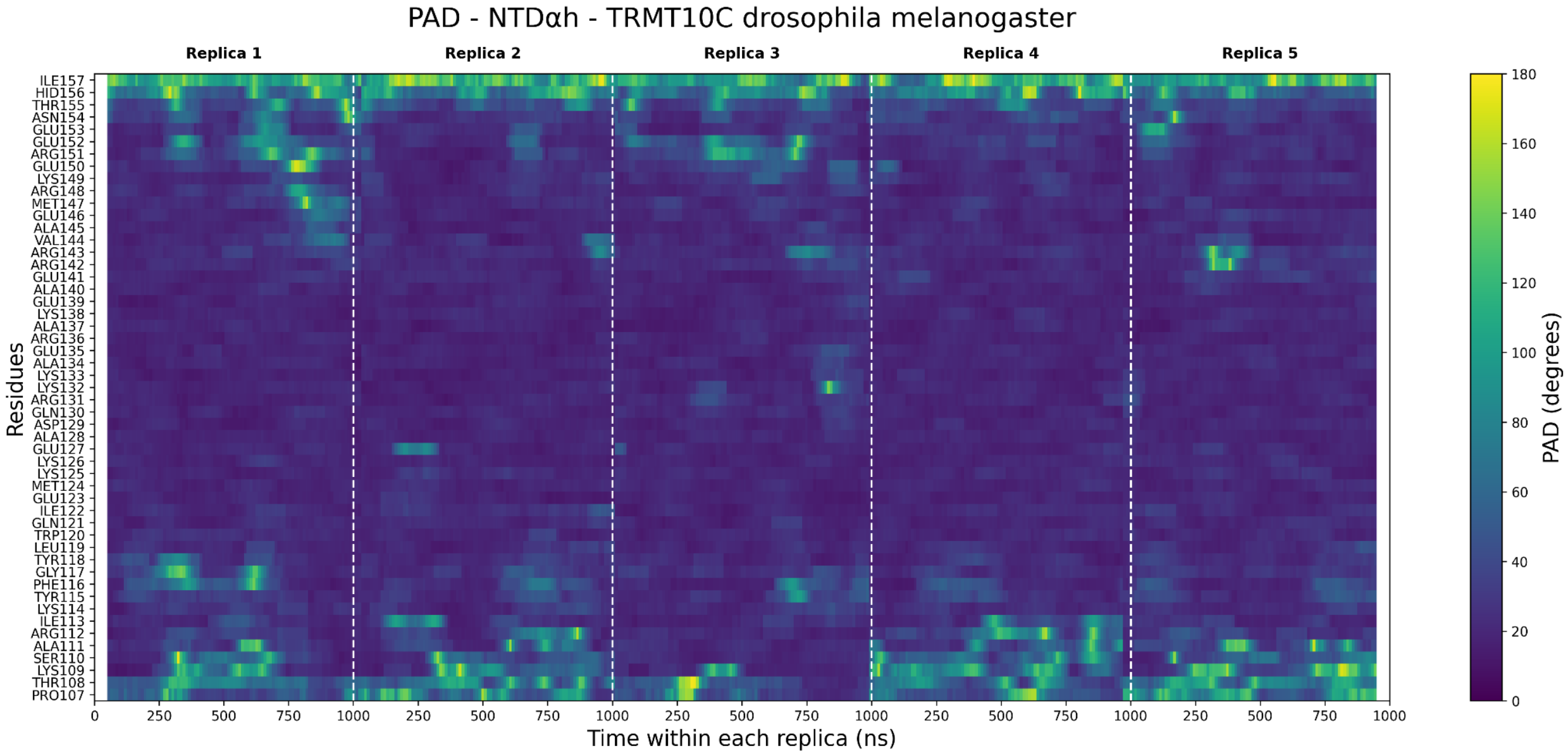

Figure S83. Same as Fig. S81 but for Amber14SB+TIP3P-based MD of *drosophila melanogaster* TRMT10C NTDαh.

6.4. SASA analysis

The SASA values of the three systems (Figs.S84-S87) are quite similar to those of **NTDαh**.

The hydrophobicity features of human TRMT10A/B NTDαh are similar to human TRMT10C **NTDαh**. In particular, the region beyond the first few residues from N-terminal to the middle of the sequence exhibits higher hydrophobicity than the region from the middle of the sequence to the C-terminus (Fig.S88). In *Drosophila melanogaster* TRMT10C NTDαh, the hydrophobicity is quite uniform along the sequence (Fig. S88).

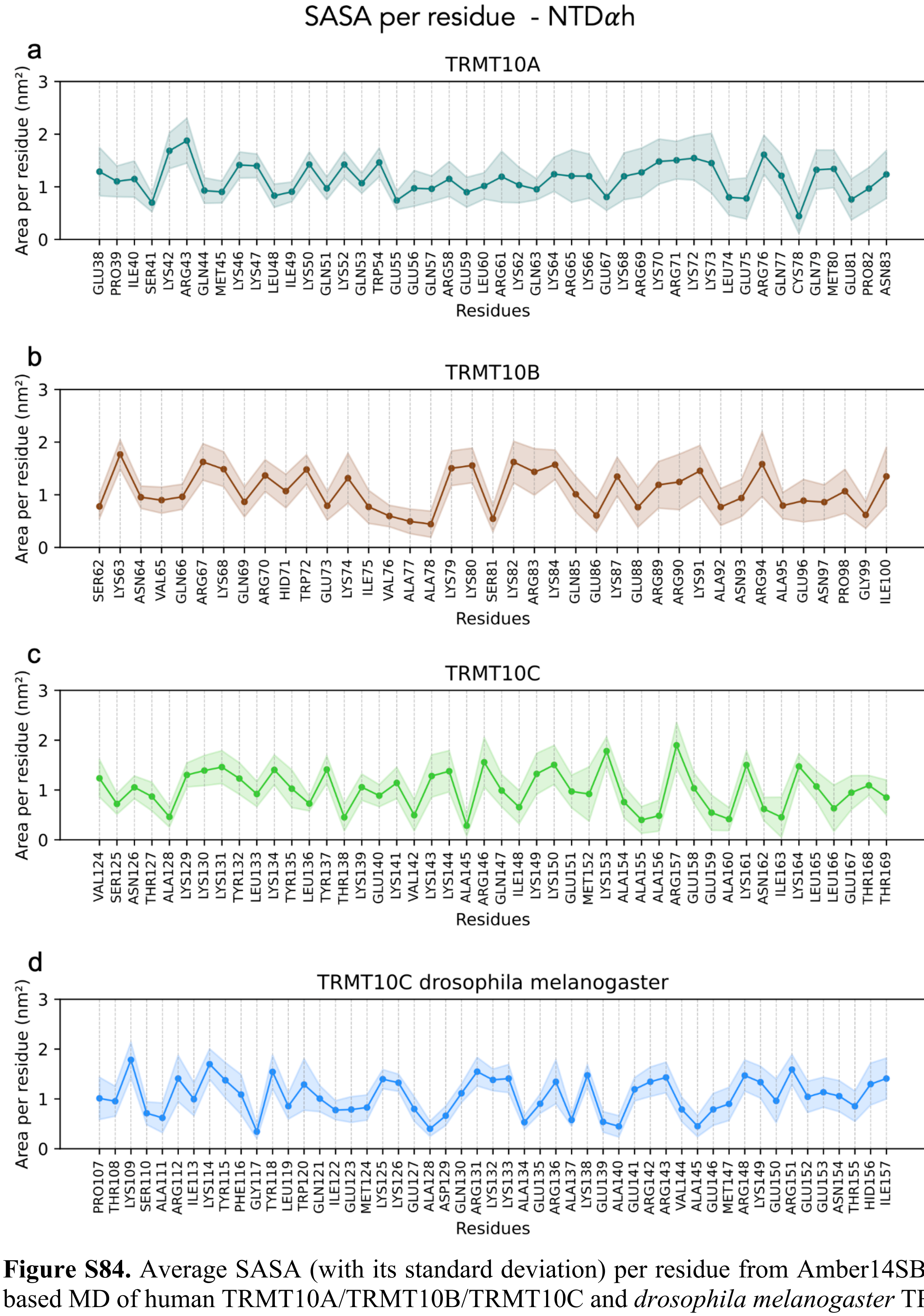

Figure S84. Average SASA (with its standard deviation) per residue from Amber14SB+TIP3P-based MD of human TRMT10A/TRMT10B/TRMT10C and *drosophila melanogaster* TRMT10C NTDαh helices.

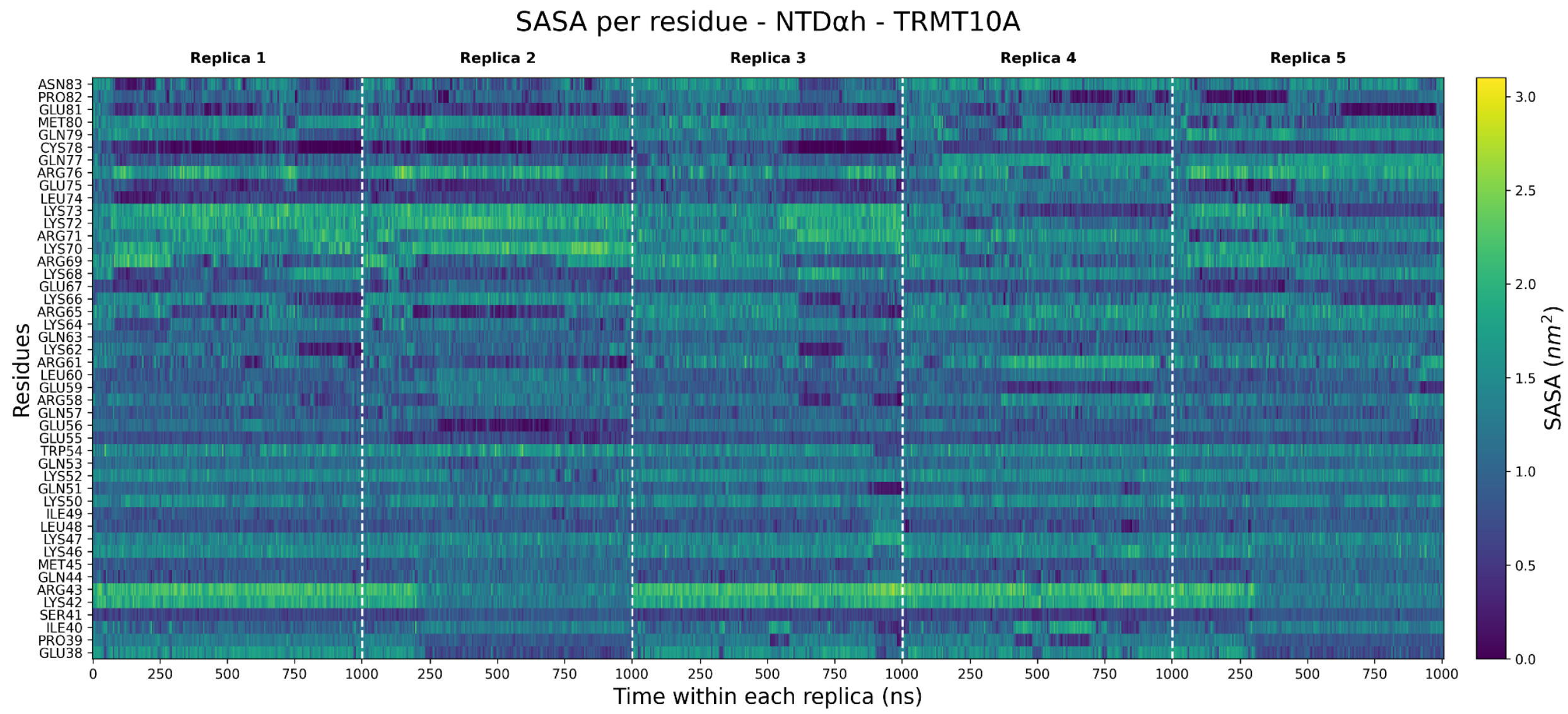

Figure S85. SASA of each residue in the Amber14SB+TIP3P-based MD of human TRMT10A NTDαh (5 replicas) plotted as a function of simulated time.

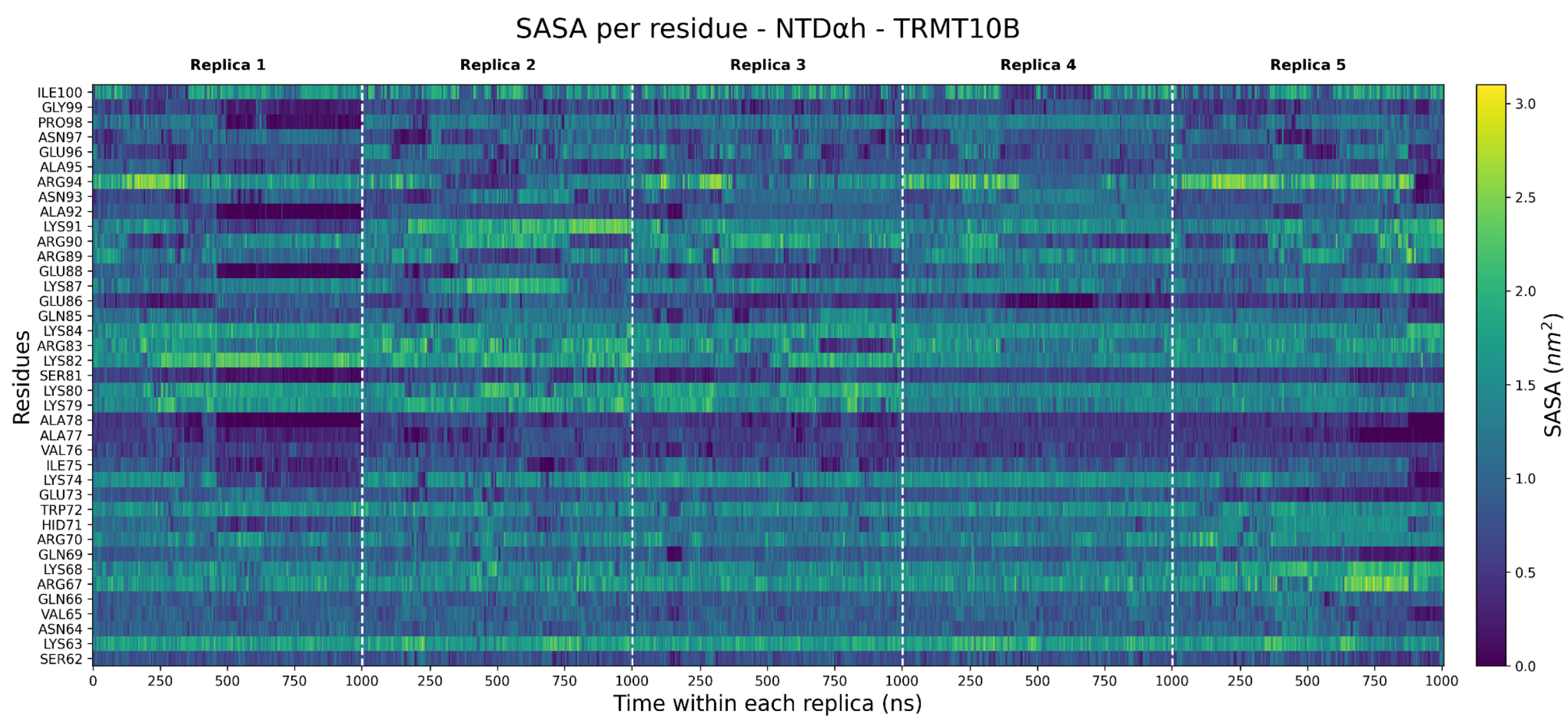

Figure S86. SASA of each residue in the Amber14SB+TIP3P-based MD of human TRMT10B NTDαh.

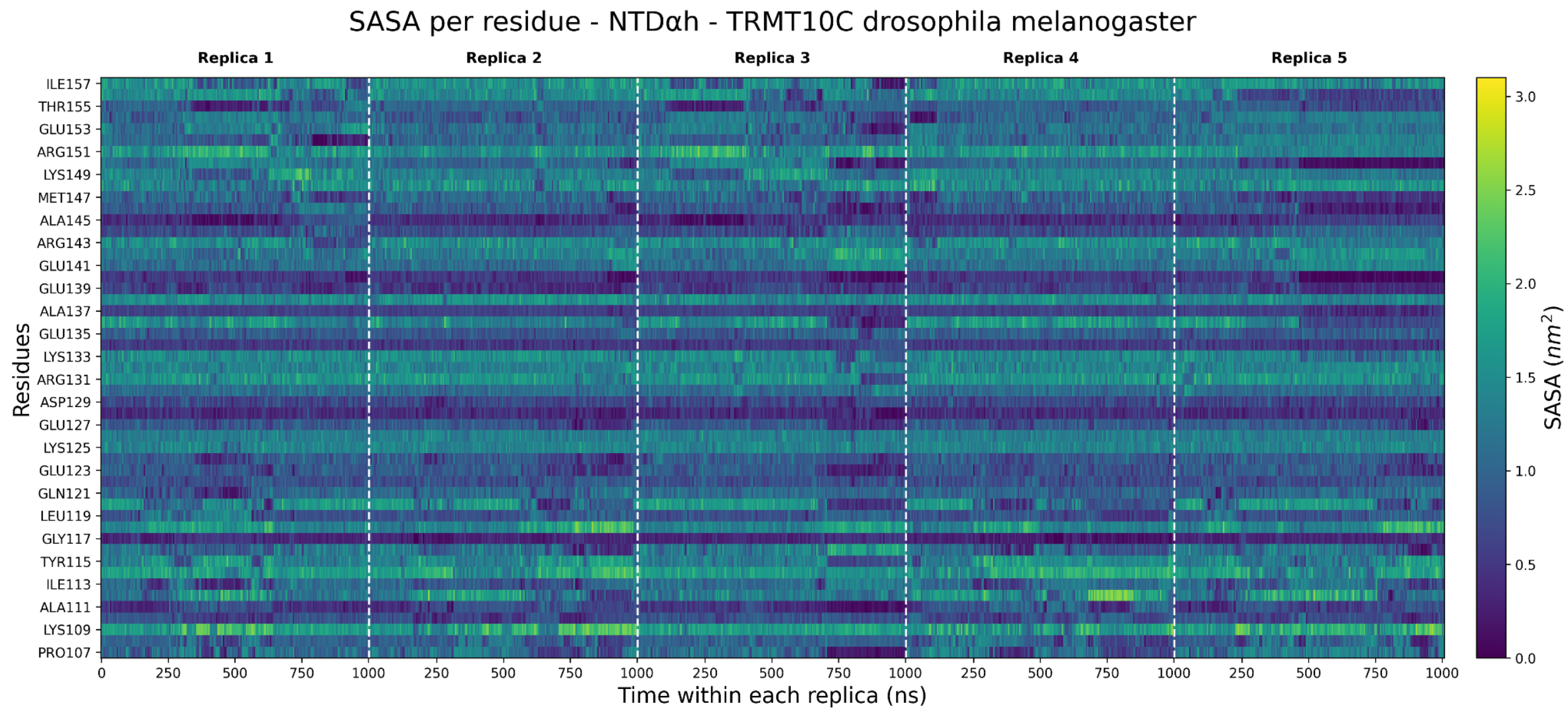

Figure S87. SASA of each residue in the the Amber14SB+TIP3P-based MD of *drosophila melanogaster* TRMT10C NTDαh.

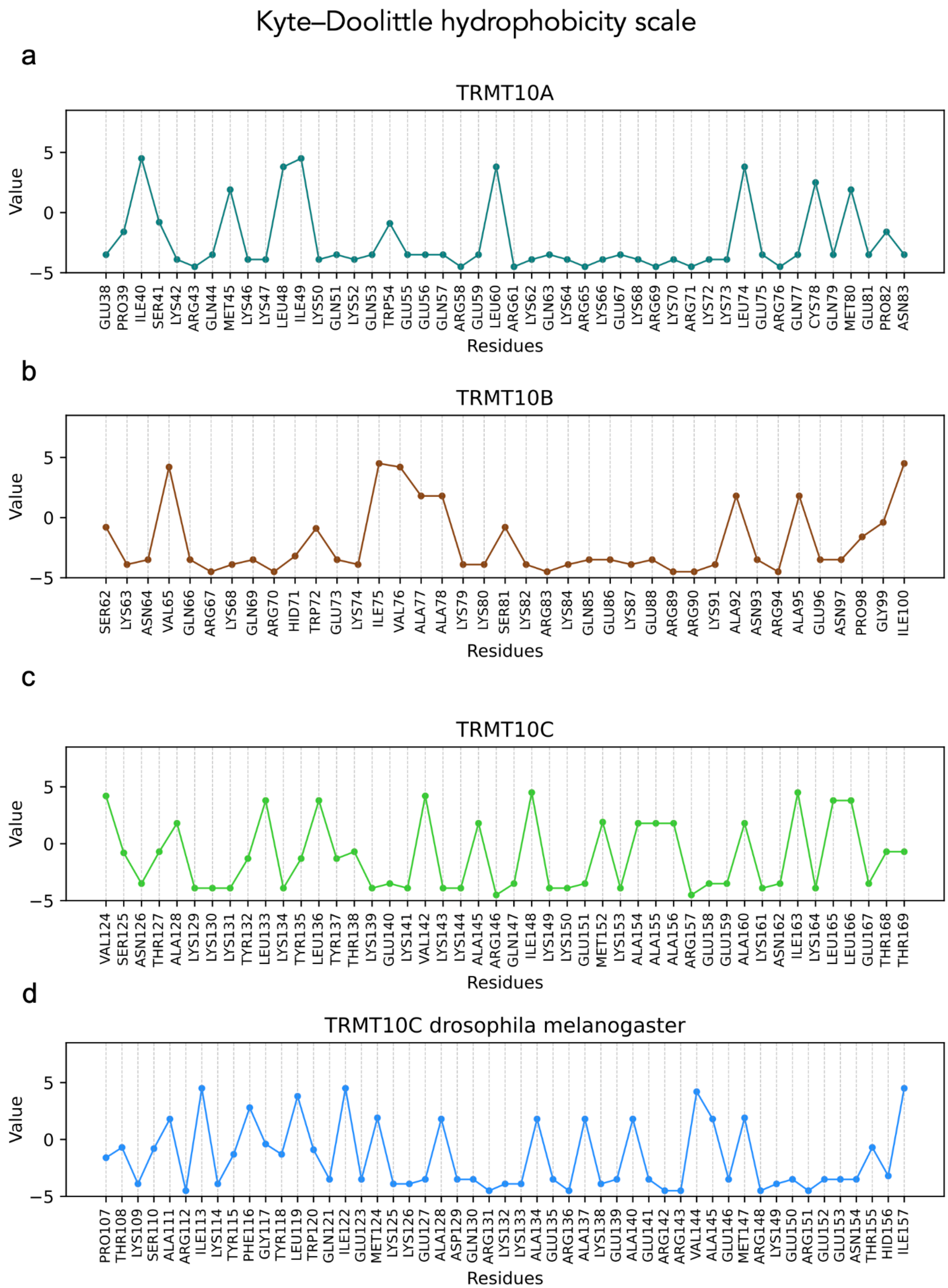

Figure S88. Hydrophobicity of each residue for human TRMT10A/TRMT10B/TRMT10C and *drosophila melanogaster* TRMT10C NTDαh, based on the Kyte-Doolittle scale[8].

6.5. Clustering analysis

The Amber14SB+TIP3P-based MD simulations of human TRMT10A/TRMT10B and *Drosophila melanogaster* TRMT10C NTDαh exhibit few clusters (3-7) accounting for 50-70% of the trajectory (Tabs S13-S15 and Figs S89-S91).

Cluster	Population
1	20.2%
2	14.0%
3	13.1%
4	10.5%
5	8.2%
6	4.3%
7	3.6%
Other (52 clusters)	26.1%

Table S13. Cluster populations from the Amber14SB+TIP3P-based MD of human TRMT10A NTDαh.

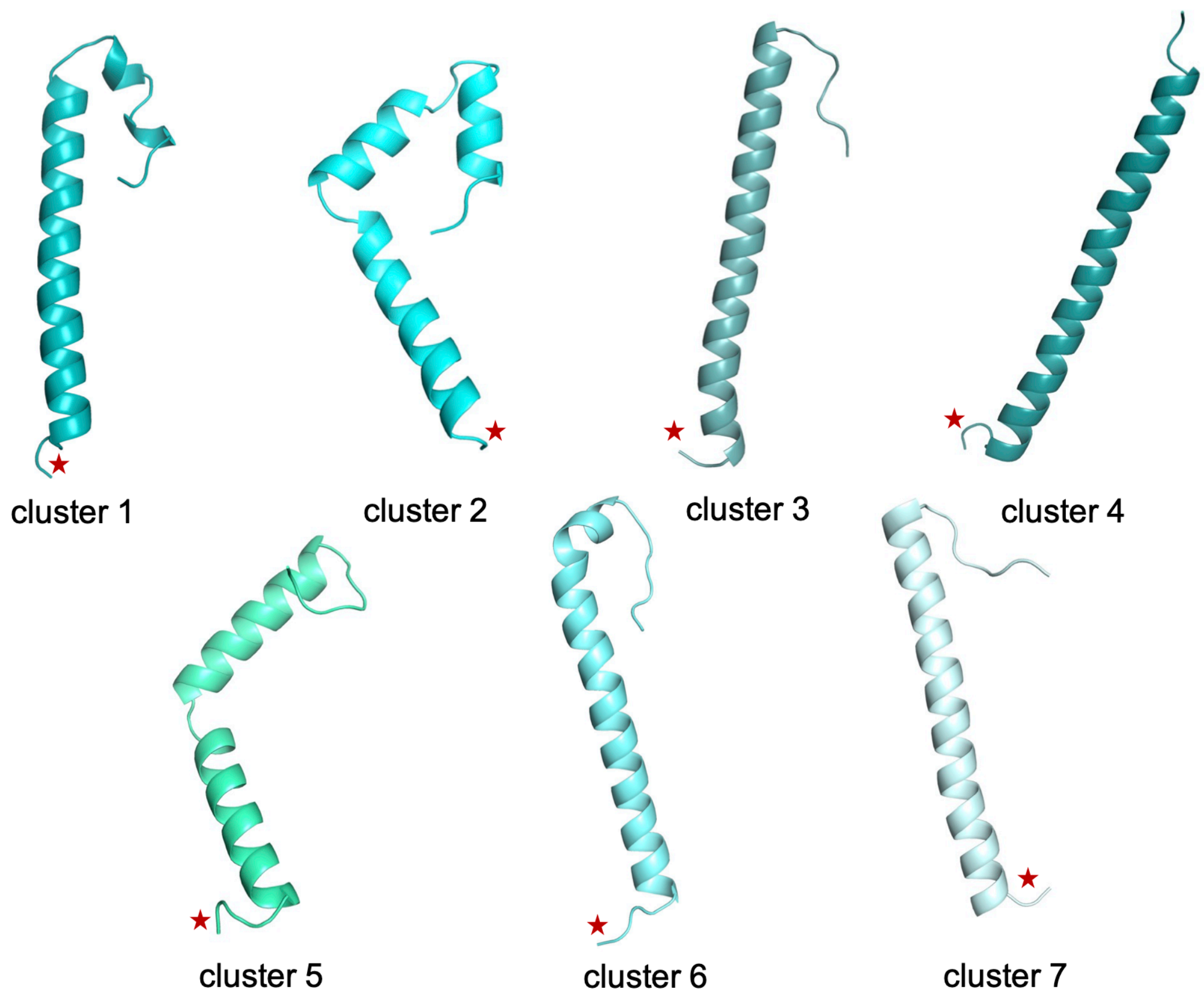

Figure S89. Representative structure of each cluster from the Amber14SB+TIP3P-based MD of human TRMT10A NTDαh. The red star highlights the N-terminus.

Cluster	Population
1	17.1%
2	11.0%
3	6.2%
4	6.2%
5	4.1%
6	3.5%
Other (163 clusters)	51.9%

Table S14. Cluster populations from the Amber14SB+TIP3P-based MD of human TRMT10B NTDαh.

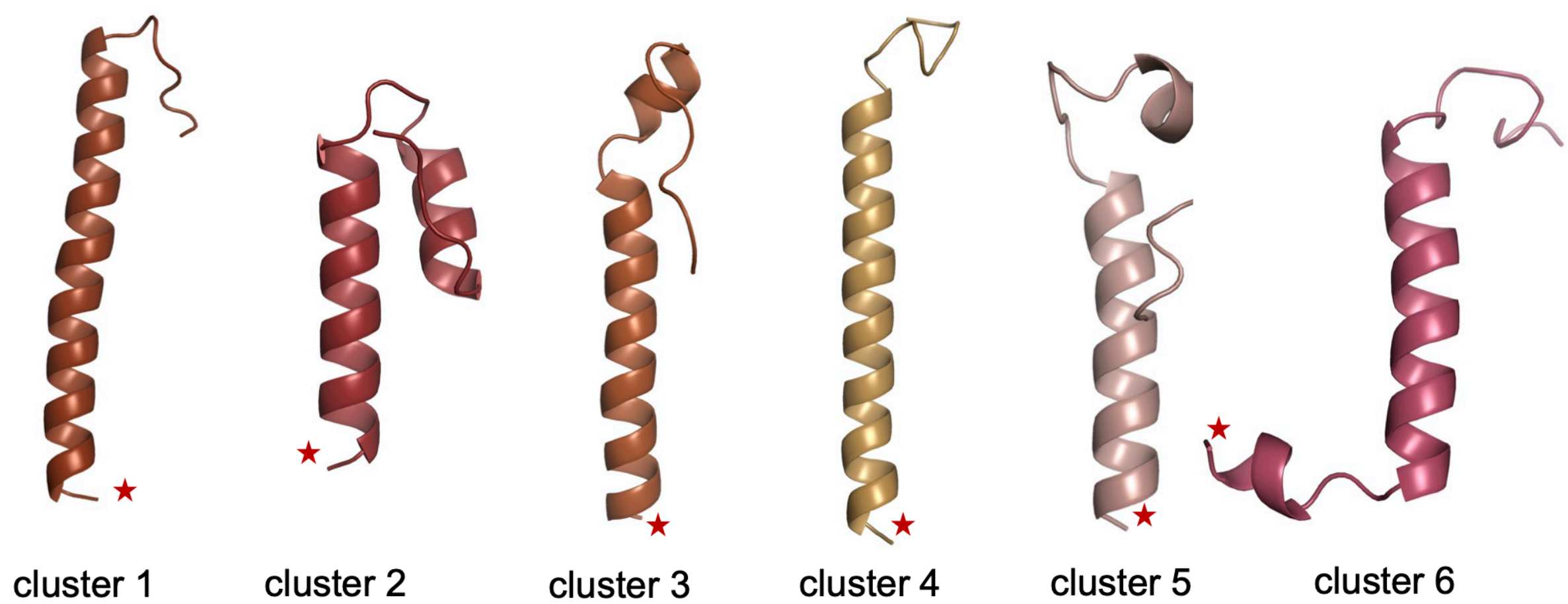

Figure S90. Same as Fig. S89 but for the Amber14SB+TIP3P-based MD of human TRMT10B NTDαh.

Cluster	Population
1	25.3%
2	16.2%
3	7.0%
4	6.1%
5	5.8%
6	4.7%
7	3.9%
Other (54 clusters)	31%

Table S15. Cluster populations from the Amber14SB+TIP3P-based MD of *drosophila melanogaster* TRMT10C NTDαh.

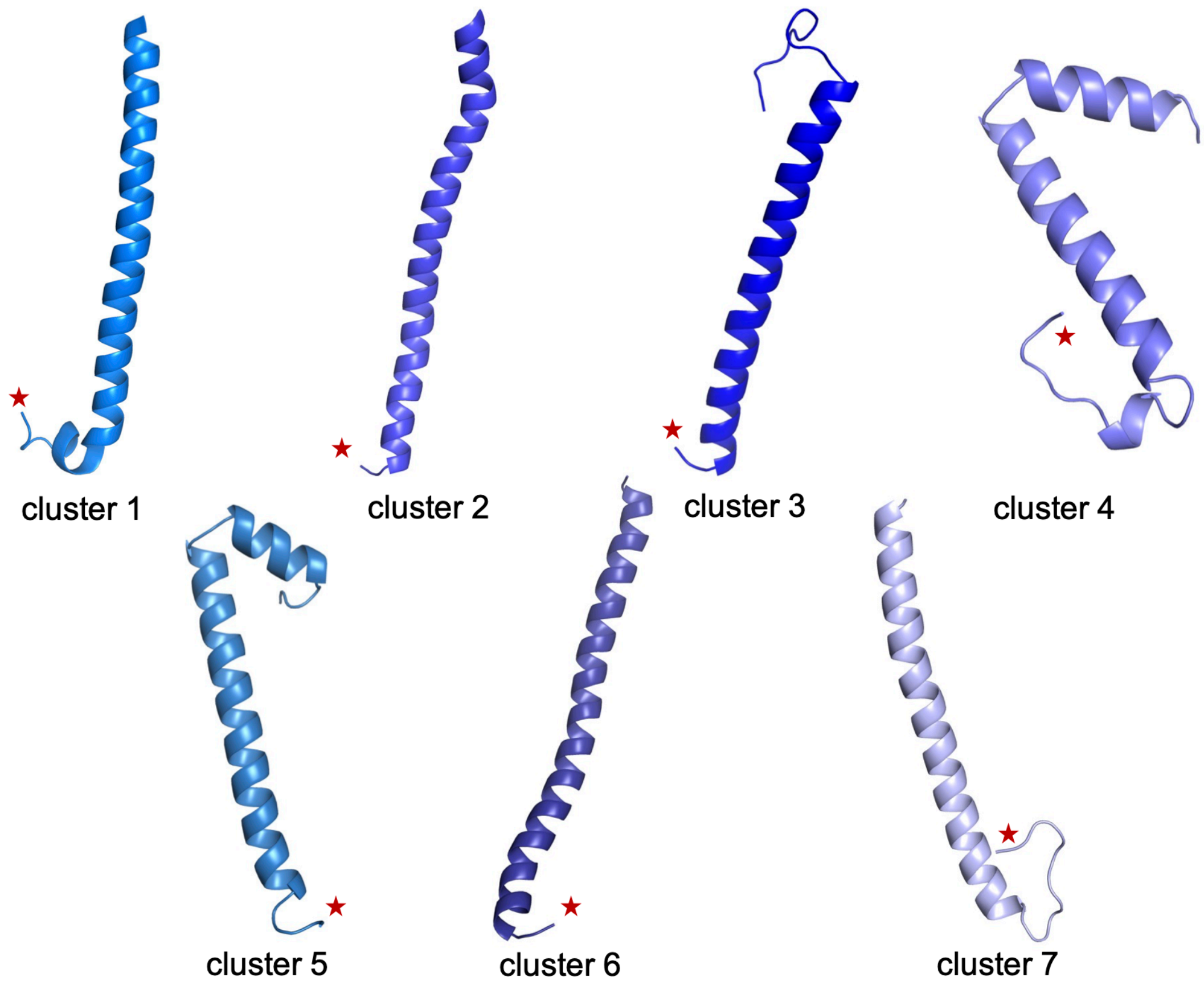

Figure S91. Same as Fig. S89 but for the Amber14SB+TIP3P-based MD of *drosophila melanogaster* TRMT10C NTDαh.

6.6. PCA analysis

In the Amber14SB+TIP3P-based MD of human TRMT10A/TRMT10B and *drosophila melanogaster* TRMT10C NTDαh, the three systems show an energy landscape not too dissimilar to that of **NTDαh**, with 2-3 basins presented (Figs.S92-S97).

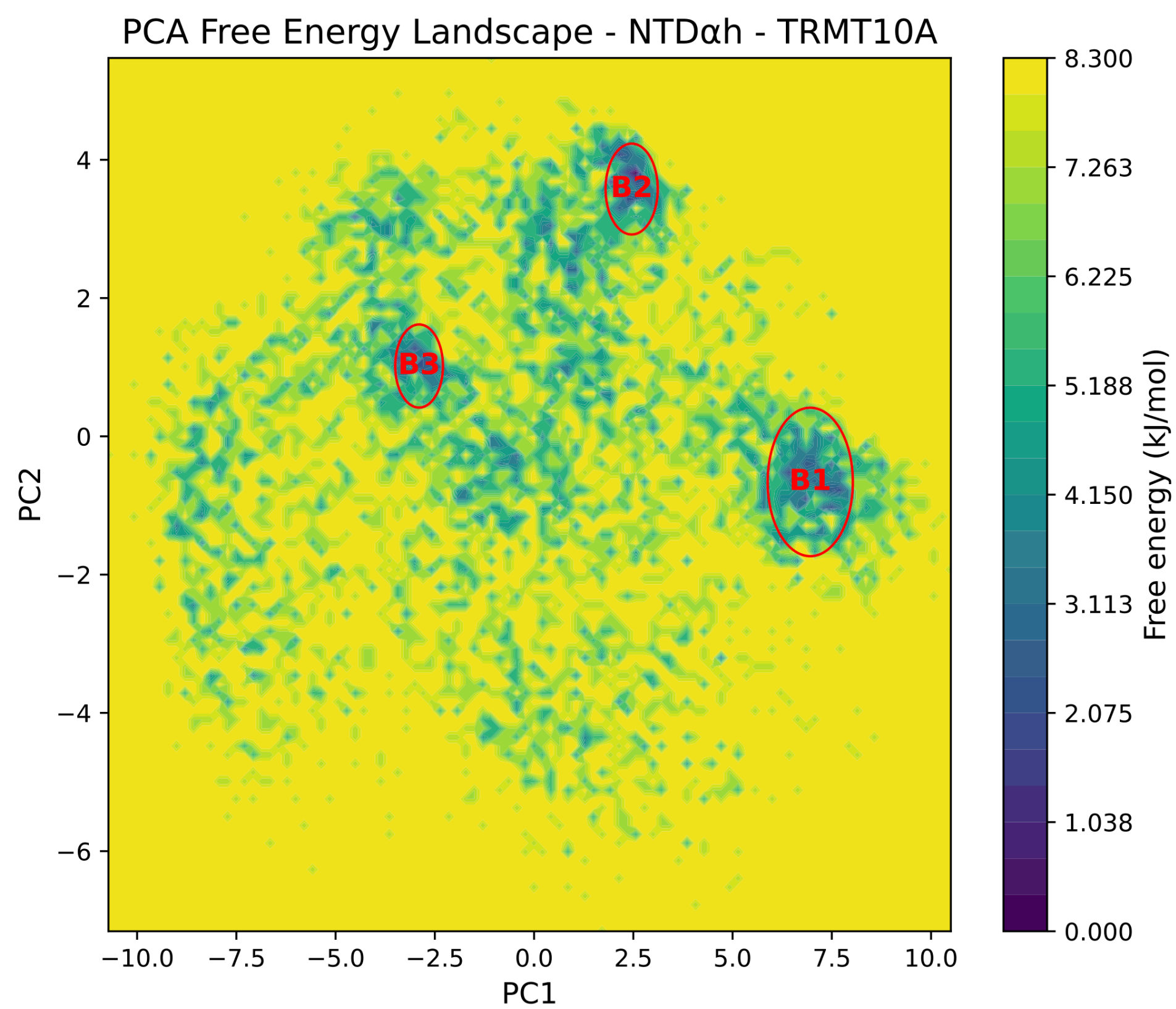

Figure S92. PCA relative free energy landscape from the Amber14SB+TIP3P-based MD of human TRMT10A NTDαh. The minimum is set to zero. The free energy is plotted as a function of the first two principal components (PC1 and PC2). The basins B1-B3 is marked with the red circles.

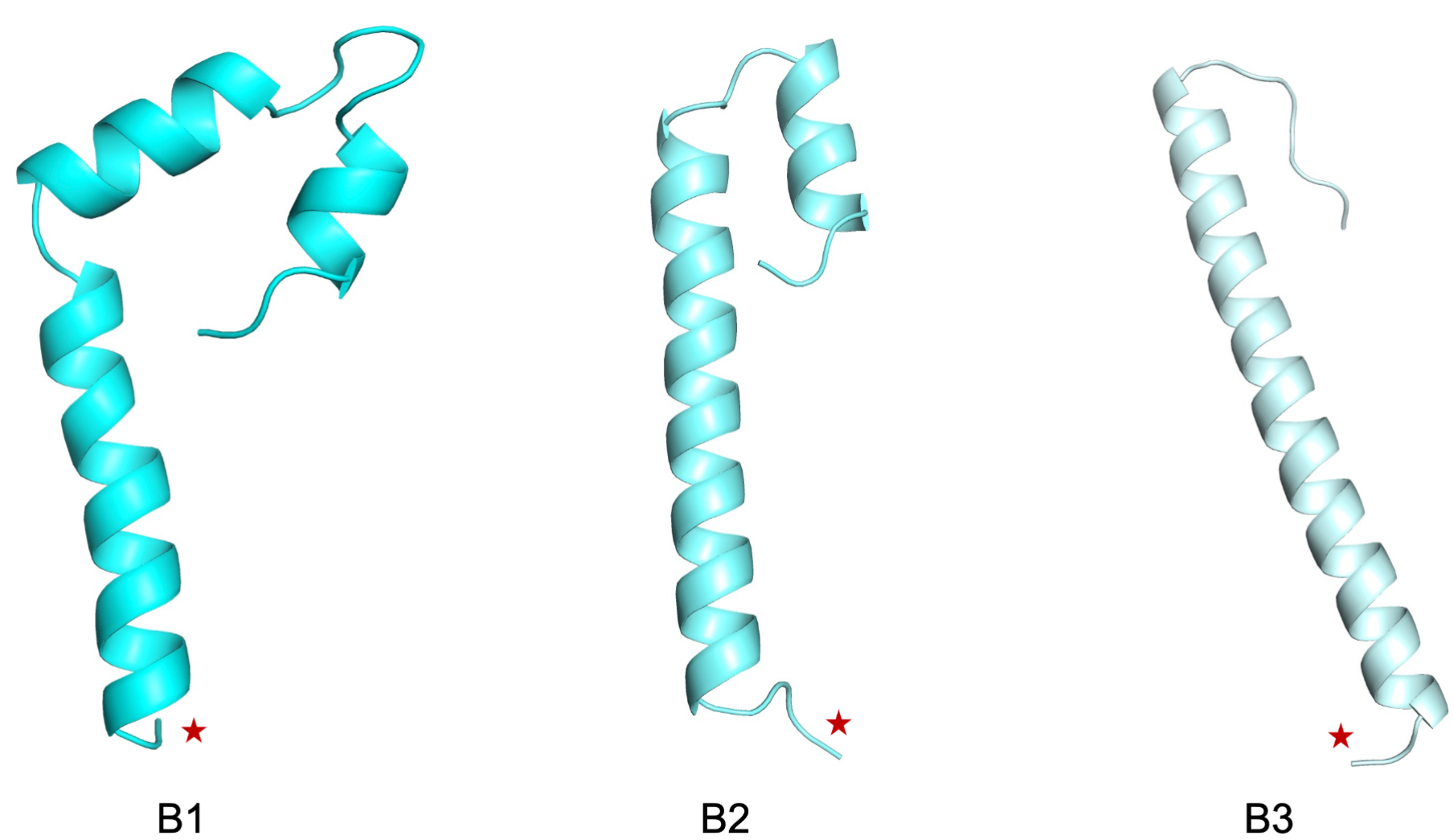

Figure S93. Representative structures from a clustering analysis of the basins in Fig. S92. The red star highlights the N-terminus.

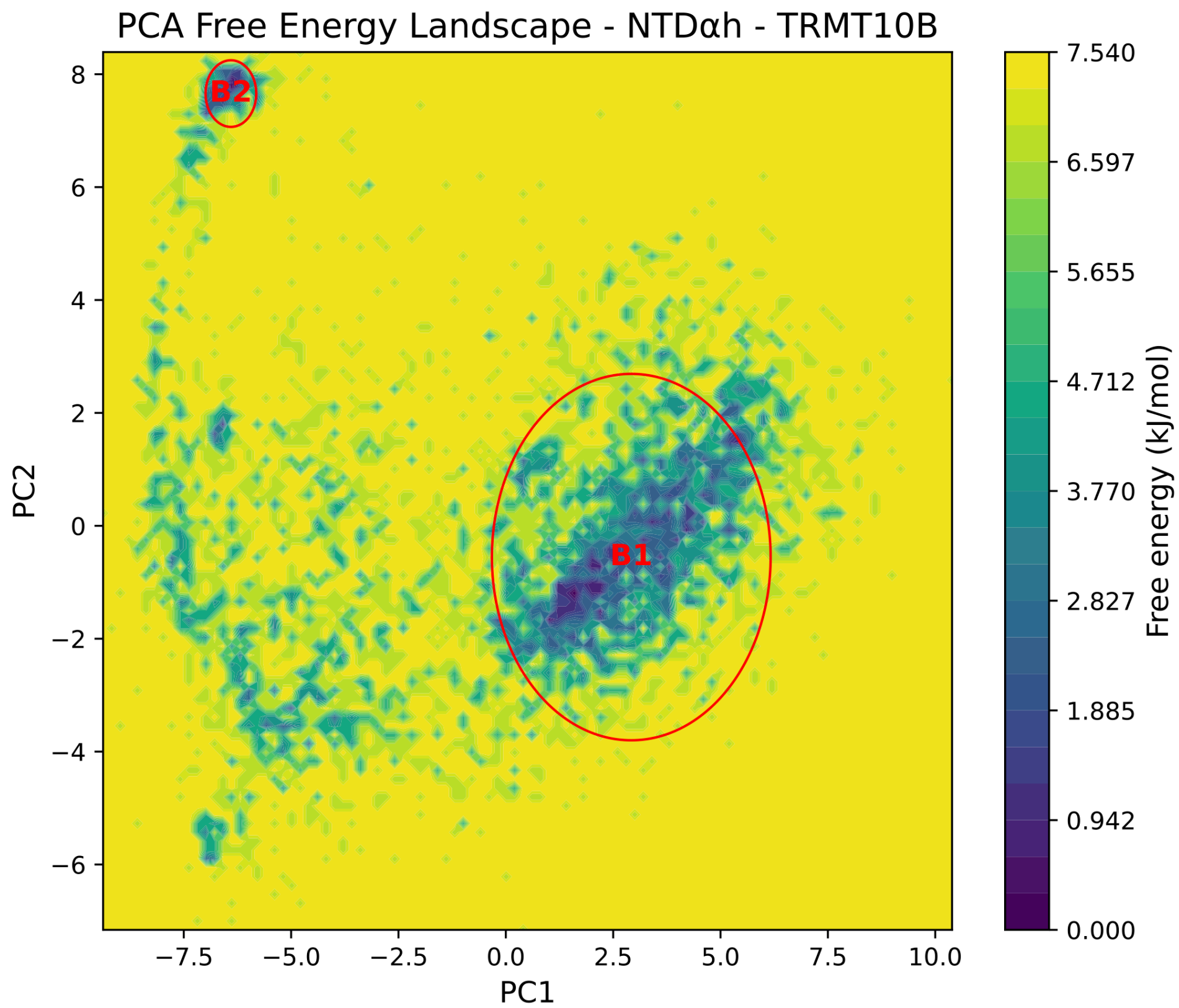

Figure S94. Same as Fig. S92 but for the Amber14SB+TIP3P-based MD of human TRMT10B NTDαh.

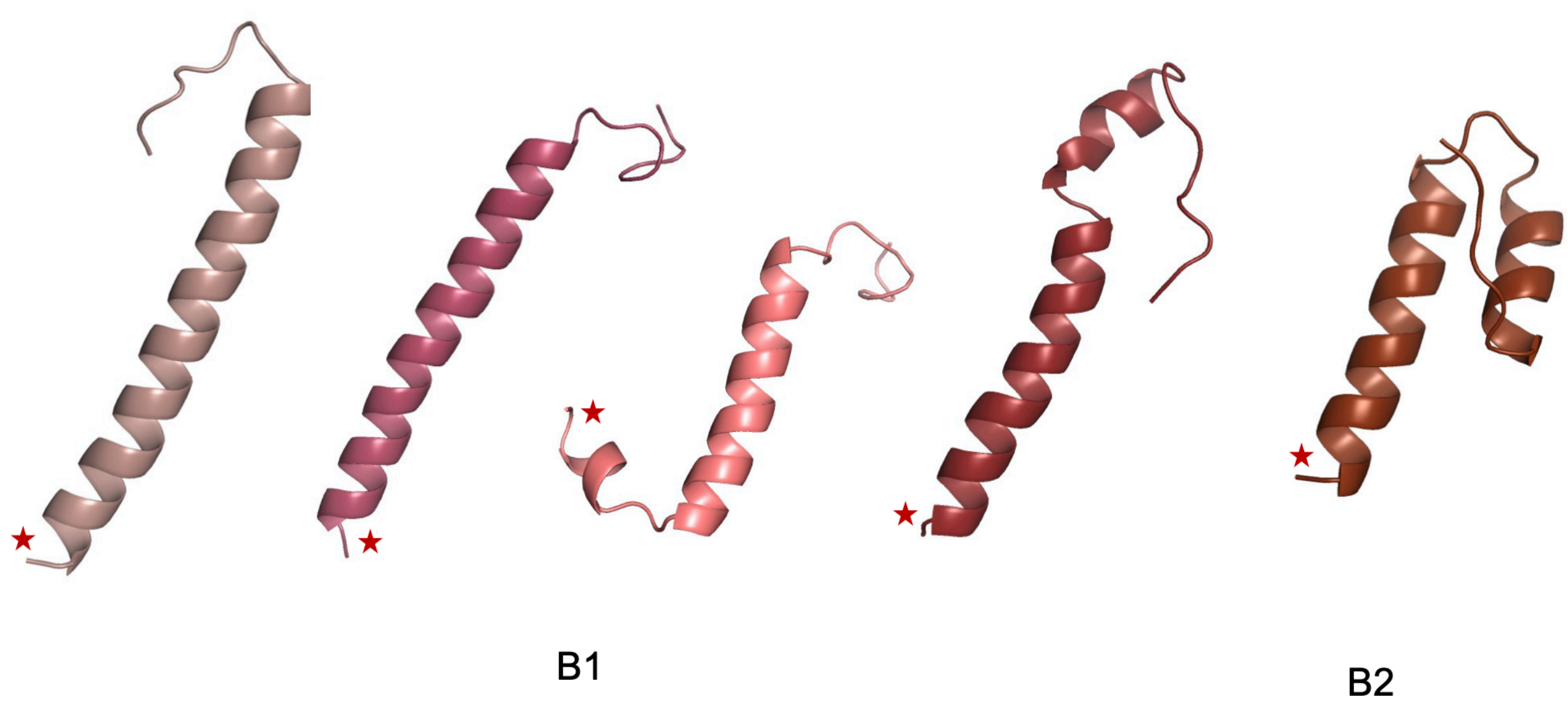

Figure S95. Representative structure from clustering analysis of the basins in Fig. S94. The red star highlights the N-terminus.

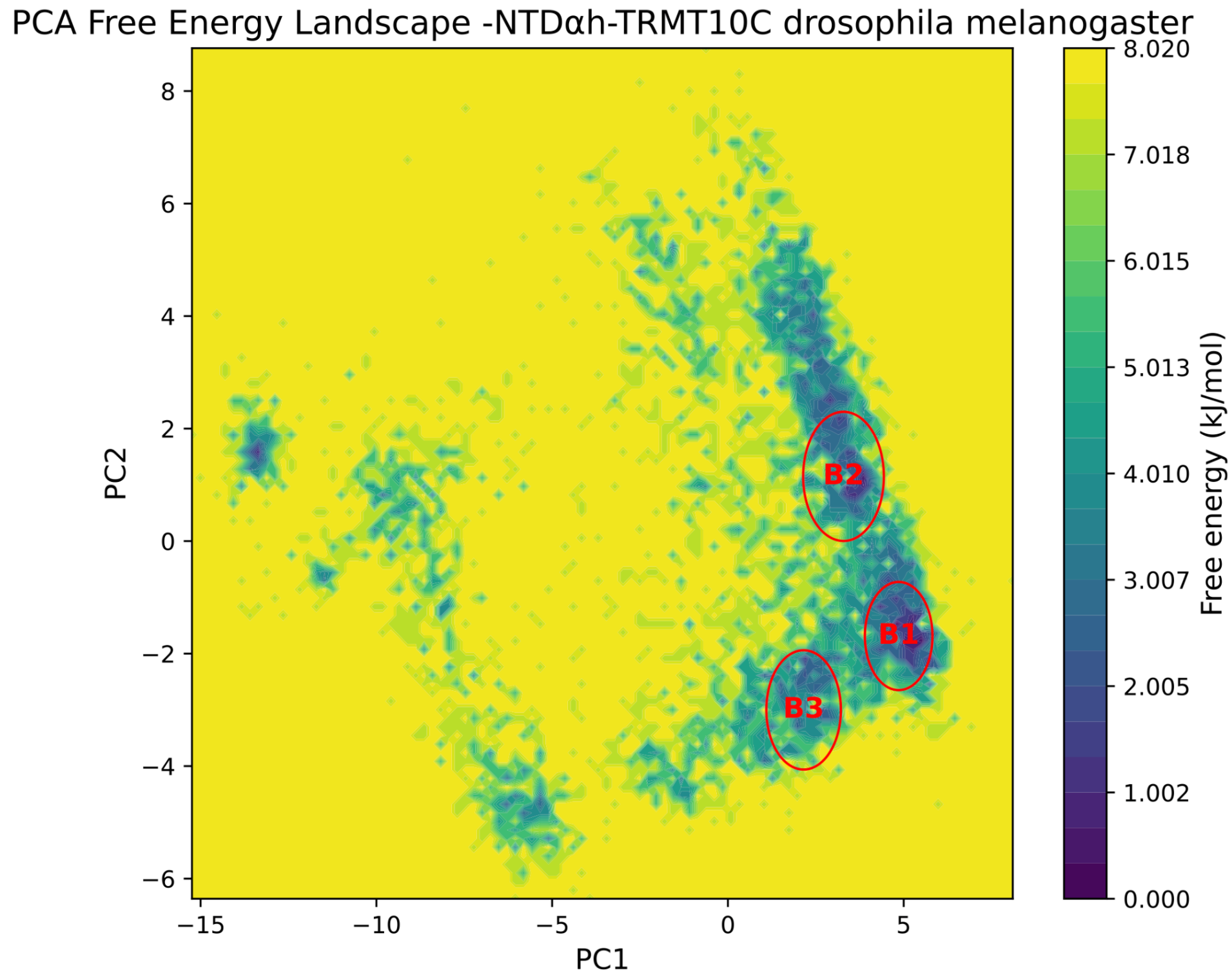

Figure S96. Same as Fig. S92 but for the Amber14SB+TIP3P-based MD of *drosophila melanogaster* TRMT10C NTDαh.

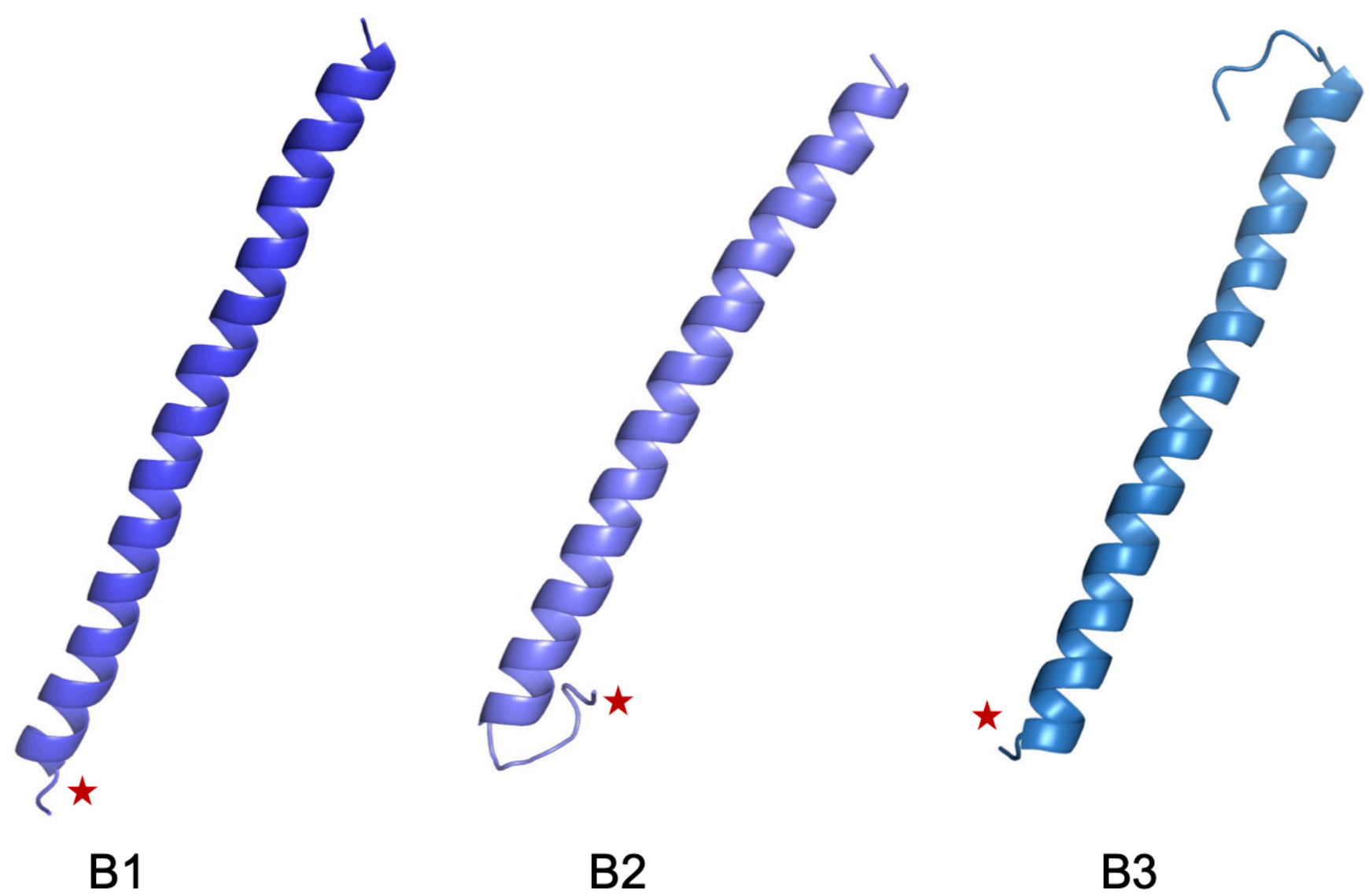

Figure S97. Representative structure from clustering analysis of the basins in Fig. S96. The red star highlights the N-terminus.

7. Salt bridge analysis

This was performed on MD trajectories of all helices using an in-house python script (based on the gmx_saltbridge script from Magnus Bertelsen[10]). The minimum distance between cationic and anionic residues is calculated for each frame of the trajectory. A pair of oppositely charged residues is considered to form a salt bridge if the minimum distance is less than a cutoff (we chose cutoff = 0.4 nm). The salt bridge analysis provides the list of salt bridge interactions with the occupancy rates along with the mean distances. In Tab.S16 to Tab.S23, only pairs with occupancy rates >45% were shown.

All charged residues of the **EK2R1αh** formed intrahelical salt bridge interactions during the MD simulations (with high occupancy rate from 47% to 95%) (Tab.S16). On the other hand, among 19 charged residues of the **NTDαh** (from 126 to 166), only 2 salt bridges were formed between 4 residues in the Amber14SB+TIP3P-based MD (with occupancy rate from 47% to 60%) (Tab.S17). Thus, there are many more salt bridges in **EK2R1αh** than in **NTDαh**.

In the Amber14SB+OPC-based MD, there is no stable salt bridge for **NTDαh** (with all occupancy rates <30%, results not shown), 6 salt-bridges for **EK2R1αh**, (with high occupancy rate from 63% to 87%, Tab.S18). Thus, also in this case, similarly there are more salt bridges in **EK2R1αh** than in **NTDαh**.

In the CHARMM+TIP3P-based MD, one salt bridge was formed in **NTDαh** (with occupancy rate 63%, Tab.S19), while two were formed in **EK2R1αh** (with occupancy rate around 50%) (Tab.S20).

In the Amber14SB+TIP3P-based MD of human TRMT10A and TRMT10B NTDαh, 2 salt bridges were formed (with occupancy rate from 46% to 90%, Tabs S21-S22). In the Amber14SB+TIP3P-based MD of *Drosophila melanogaster* TRMT10C NTDαh, 5 salt bridges were formed (high occupancy rate from 46% to 93%, Tab.S23).

Positive charged residue	Negative charged residue	Occupancy rate (%)	Mean distance (nm)
ARG130	GLU127	95.14	0.281
ARG151	GLU148	94.47	0.281
ARG144	GLU141	94.21	0.281
ARG137	GLU134	91.08	0.281
ARG158	GLU155	90.84	0.282
ARG165	GLU162	89.21	0.283
LYS131	GLU128	62.27	0.286
LYS152	GLU149	60.48	0.287
LYS138	GLU135	57.5	0.288
LYS159	GLU156	57.03	0.286
LYS145	GLU142	56.83	0.287
LYS166	GLU163	56.8	0.286

LYS157	GLU154	51.03	0.288
LYS136	GLU133	49.74	0.288
LYS150	GLU147	49.48	0.289
LYS129	GLU126	49.19	0.287
LYS164	GLU161	48.54	0.289
LYS143	GLU140	46.93	0.29

Table S16. Salt bridges formed during the Amber14SB+TIP3P-based MD of **EK2R1αh.**

Positive charged residue	Negative charged residue	Occupancy rate (%)	Mean distance (nm)
LYS143	GLU140	60.23	0.29
LYS161	GLU158	46.99	0.29

Table S17. Salt bridges formed during the Amber14SB+TIP3P-based MD of **NTDαh.**

Positive charged residue	Negative charged residue	Occupancy rate (%)	Mean distance (nm)
ARG151	GLU148	87.29	0.283
ARG137	GLU134	86.35	0.283
ARG158	GLU155	85.29	0.282
ARG144	GLU141	84.76	0.284
ARG130	GLU127	80.9	0.283
ARG165	GLU162	63.35	0.286

Table S18. Salt bridges formed during the Amber14SB+OPC-based MD of **EK2R1αh.**

Positive charged residue	Negative charged residue	Occupancy rate (%)	Mean distance (nm)
LYS143	GLU140	63.17	0.18

Table S19. Salt bridges formed during the CHARMM+TIP3P-based MD of **NTDαh.**

Positive charged residue	Negative charged residue	Occupancy rate (%)	Mean distance (nm)
LYS131	GLU134	48.89	0.19

LYS138	GLU141	45.57	0.19

Table S20. Salt bridges formed during the CHARMM+TIP3P-based MD of **EK2R1αh**.

Positive charged residue	Negative charged residue	Occupancy rate (%)	Mean distance (nm)
ARG58	GLU55	90.06	0.283
LYS62	GLU59	46.3	0.292

Table S21. Salt bridges formed during the Amber14SB+TIP3P-based MD of human TRMT10A NTDαh.

Positive charged residue	Negative charged residue	Occupancy rate (%)	Mean distance (nm)
ARG89	GLU86	72.72	0,283
ARG70	GLU73	67.41	0,283

Table S22. Salt bridges formed during the Amber14SB+TIP3P-based MD of human TRMT10B NTDαh.

Positive charged residue	Negative charged residue	Occupancy rate (%)	Mean distance (nm)
ARG142	GLU139	93.72	0.281
LYS126	GLU123	65.09	0.286
ARG131	GLU127	64.01	0.296
ARG143	GLU139	53.48	0.309
LYS125	ASP129	46.36	0.281

Table S23. Salt bridges formed during the Amber14SB+TIP3P-based MD of *drosophila melanogaster* TRMT10C NTDαh.

8. Molecular dynamics study of the 21-residue polyalanine peptide

Table S24. Molecular dynamics of the longest polyalanine peptides investigated to date[11]. The force field employed, polyalanine peptide length, terminal groups, length of the simulation and

helical content (%) are reported. Overall helical content was estimated as the area under the per-residue helical fraction curve (using linear interpolation and the trapezoidal rule) divided by the total number of residues.

Force field	Peptide length	Helical content (%)	Length of the MD simulation
AMBER19SB+OPC[6,12]	n=21	~60%	5 µs
AMBER14SB +TIP3P[5,7]	n=21	~35%	5 µs
CHARMM36m+TIP3P[9]	n=21	~45%	5 µs

REFERENCES

(1) Dahl, A. C. E.; Chavent, M.; Sansom, M. S. P. Bendix: Intuitive Helix Geometry Analysis and Abstraction. *Bioinformatics* **2012**, *28* (16), 2193–2194. https://doi.org/10.1093/bioinformatics/bts357
(2) Humphrey, W.; Dalke, A.; Schulten, K. VMD: Visual Molecular Dynamics. *Journal of Molecular Graphics* **1996**, *14* (1), 33–38. https://doi.org/10.1016/0263-7855(96)00018-5
(3) Daura, X.; Gademann, K.; Jaun, B.; Seebach, D.; Van Gunsteren, W. F.; Mark, A. E. Peptide Folding: When Simulation Meets Experiment. *Angew. Chem. Int. Ed.* **1999**, *38* (1–2), 236–240. https://doi.org/10.1002/(SICI)1521-3773(19990115)38:1/2%3C236::AID-ANIE236%3E3.0.CO;2-M
(4) Abraham, M.; Alekseenko, A.; Basov, V.; Bergh, C.; Briand, E.; Brown, A.; Doijade, M.; Fiorin, G.; Fleischmann, S.; Gorelov, S.; Gouaillardet, G.; Grey, A.; Irrgang, M. E.; Jalalypour, F.; Jordan, J.; Kutzner, C.; Lemkul, J. A.; Lundborg, M.; Merz, P.; Miletic, V.; Morozov, D.; Nabet, J.; Pall, S.; Pasquadibisceglie, A.; Pellegrino, M.; Santuz, H.; Schulz, R.; Shugaeva, T.; Shvetsov, A.; Villa, A.; Wingbermuehle, S.; Hess, B.; Lindahl, E. GROMACS 2024.3 Manual. **2024**. https://doi.org/10.5281/zenodo.13457083
(5) Maier, J. A.; Martinez, C.; Kasavajhala, K.; Wickstrom, L.; Hauser, K. E.; Simmerling, C. ff14SB: Improving the Accuracy of Protein Side Chain and Backbone Parameters from ff99SB. *J. Chem. Theory Comput.* **2015**, *11* (8), 3696–3713. https://doi.org/10.1021/acs.jctc.5b00255
(6) Izadi, S.; Anandakrishnan, R.; Onufriev, A. V. Building Water Models: A Different Approach. *J. Phys. Chem. Lett.* **2014**, *5* (21), 3863–3871. https://doi.org/10.1021/jz501780a
(7) Jorgensen, W. L.; Chandrasekhar, J.; Madura, J. D.; Impey, R. W.; Klein, M. L. Comparison of Simple Potential Functions for Simulating Liquid Water. *The Journal of Chemical Physics* **1983**, *79* (2), 926–935. https://doi.org/10.1063/1.445869
(8) Kyte, J.; Doolittle, R. F. A Simple Method for Displaying the Hydropathic Character of a Protein. *Journal of Molecular Biology* **1982**, *157* (1), 105–132. https://doi.org/10.1016/0022-2836(82)90515-0

(9) Huang, J.; Rauscher, S.; Nawrocki, G.; Ran, T.; Feig, M.; De Groot, B. L.; Grubmüller, H.; MacKerell, A. D. CHARMM36m: An Improved Force Field for Folded and Intrinsically Disordered Proteins. *Nat Methods* **2017**, *14* (1), 71–73. https://doi.org/10.1038/nmeth.4067
(10) Gmx_saltbridge. https://github.com/MagnusBertelsen/gmx_saltbridge
(11) Hess, N.; Joseph, J. A. BiomolecularCondensates Dictate the Folding Landscapeof Protein Alpha-Helices. *Journal of the American Chemical Society* **2026**, *148* (34), 36392–36413. https://doi.org/10.1021/jacs.6c01403
(12) Tian, C.; Kasavajhala, K.; Belfon, K. A. A.; Raguette, L.; Huang, H.; Migues, A. N.; Bickel, J.; Wang, Y.; Pincay, J.; Wu, Q.; Simmerling, C. ff19SB: Amino-Acid-Specific Protein Backbone Parameters Trained against Quantum Mechanics Energy Surfaces in Solution. *J. Chem. Theory Comput.* **2020**, *16* (1), 528–552. https://doi.org/10.1021/acs.jctc.9b00591
